# Postoperative outcomes in older patients with postoperative delirium in the UK: SNAP-3, a snapshot observational study

**DOI:** 10.1016/j.bja.2026.01.030

**Published:** 2026-02-26

**Authors:** Helen A. Blake, Claire J. Swarbrick, SNAP-3 collaborators, Karen Williams, Bob Evans, Thomas Poulton, Samuel Nava, Akshay Shah, Peter Martin, Judith S.L. Partridge, Iain K. Moppett

**Affiliations:** 1 University College London, London, UK; 2 University of Nottingham, Nottingham, UK; 3 Royal College of Anaesthetists, London, UK; 4 Royal Devon University Healthcare NHS Foundation Trust, Exeter, UK; 5 Peter MacCallum Cancer Centre, Melbourne, VIC, Australia; 6 University of Melbourne, Melbourne, VIC, Australia; 7 Royal United Hospitals Bath NHS Foundation Trust, Bath, UK; 8 University of Oxford, Oxford, UK; 9 Imperial College Healthcare NHS Trust, London, UK; 10 Guy's and St Thomas' NHS Foundation Trust, London, UK; 11 King’s College Hospital, London, UK; 12 Nottingham University Hospitals NHS Trust, Nottingham, UK

**Keywords:** ageing, delirium, epidemiology, frailty, geriatric medicine, National Health Service, perioperative care, surgery

## Abstract

**Background:**

Delirium occurs in about 6% of older surgical patients and more frequently in those who are older or frail or undergo urgent surgery. It is associated with adverse postoperative outcomes. However, it remains unclear to what extent delirium is the cause of worse outcomes and to what extent it is a marker of other pre-existing vulnerabilities.

**Methods:**

We conducted a prospective observational cohort study of UK patients aged 60 yr and above undergoing surgery (excluding minor procedures) over 5 days in March 2022. Data were collected on patient characteristics and clinical variables, postoperative delirium, length of stay (LOS) in hospital, morbidity, and mortality. Delirium was assessed on postoperative days 3 and 7 using the four A's Test or Confusion Assessment Method for the Intensive Care Unit tools, supplemented by structured notes review. We analysed the impact of delirium on postoperative outcomes using quantile regression and mixed effects logistic regression, with adjustment for confounding informed by directed acyclic graphs and covariate missing data handled using multiple imputation.

**Results:**

Analysis included 7128 patients from 214 hospitals. The incidence of delirium was 6.7% (479/7128). Delirium was associated with longer LOS (increase in median LOS of 5.2 days, effect estimate 95% confidence interval [CI], 3.8–6.5), higher odds of postoperative morbidity (adjusted odds ratio [aOR], 10.2; 95% CI, 7.4–13.9), 120-day mortality (aOR, 1.8; 95% CI, 1.2–2.6), and 1-yr mortality (aOR, 2.0; 95% CI, 1.5–2.7). Sensitivity analysis excluding lower-risk daycase patients found similar results to the main analysis.

**Conclusions:**

Delirium, which is common in older people having surgery, negatively impacts on postoperative outcomes and thus must be included in perioperative shared decision-making with patients considering surgical interventions.

**Editor’s key points**

• Postoperative delirium is common in older surgical patients, and is associated with adverse postoperative outcomes. However, whether delirium is the cause of worse outcomes or a marker of other pre-existing vulnerabilities is unclear.
• This preplanned analysis of data from the third Sprint National Anaesthesia Project (SNAP-3), a prospective study of surgical patients aged ≥60 yr in the UK, analysed the impact of delirium on postoperative length of stay (LOS), morbidity, and mortality at 30 days, 120 days, and 1 yr.
• Of 7128 patients included, the overall incidence of delirium was 6.7%. Delirium was associated with longer LOS, greater risk of postoperative morbidity, 120-day mortality, and 1-yr mortality.
• These findings are important for shared decision-making involving older patients undergoing surgical procedures.

Delirium occurs in ∼6% of older surgical patients, ∼20% of those living with frailty,^1^ and ∼30% of people after hip fracture.^2^^,^^3^ It is distressing for patients, relatives, and staff, even after recovery.^4^^,^^5^ Delirium has consistently been associated with worse outcomes in medical and surgical patients.^6^ The occurrence of delirium is strongly associated with patient and surgical factors including age, urgency of surgery, and frailty.^1^ However, it remains unclear to what extent delirium is the cause of worse outcomes and how much it is a marker of other pre-existing vulnerabilities.

The impact of delirium on postoperative outcomes, accounting for confounding factors, has not been investigated within a large prospective and representative cohort. The third Sprint National Anaesthesia Project (SNAP-3) was a prospective, national study of elective and emergency surgical patients aged ≥60 yr in the UK. The SNAP-3 study collected data on a range of risk factors for adverse postoperative outcomes including a prospective assessment for delirium. The aim of this pre-planned study was to analyse the impact of delirium on postoperative length of stay (LOS), morbidity, and mortality at 30 days, 120 days, and 1 yr.

## Methods

### Data collection and sample

The methods and regulatory approvals for SNAP-3 study have been described.^1^^,^^7^, ^8^, ^9^ In summary, all UK hospitals that deliver adult surgical services were invited to participate in a prospective observational cohort study. SNAP-3 aimed to recruit all patients aged ≥60 yr, undergoing a surgical procedure during five consecutive days (Monday–Friday) in March 2022. It was conducted between waves of the COVID-19 pandemic. SNAP-3 recruited patients undergoing surgery under general, regional, neuraxial, and local anaesthesia, including participants unable to consent to study participation. Patients undergoing very minor procedures, such as cataract surgery, were excluded (Supplementary Table 1 in Swarbrick and colleagues^1^).

Local investigators, including anaesthetists and research nurses, collected patient, medical, surgical, laboratory, risk score, socioeconomic, and frailty data. A central team ensured data quality and completeness, addressing queries with sites. Access to web-based training on Clinical Frailty Scale (CFS) assessment was promoted.^10^ The characteristics of the SNAP-3 cohort have been described.^1^^,^^8^^,^^9^

### Exposure

Postoperative delirium was assessed by local investigators using the four A's Test (4AT) or Confusion Assessment Method for the Intensive Care Unit (CAM-ICU) for patients who were still in hospital on postoperative days 3 and 7.^11^^,^^12^ The investigators were anaesthesia residents and research nurses who were provided with training in the use of the 4AT. The 4AT is explicitly designed as a tool that is reliable without extensive training.

Delirium fluctuates, making it challenging to diagnose, so a structured retrospective notes review to identify *Diagnostic Statistical Manual of Mental Disorders-5* (*DSM-5*) aligned indicators of delirium was conducted for patients staying at least one night in hospital after surgery until discharge or for up to 7 days (whichever occurred first).^13^ This involved identification of terms frequently used in medical documentation to describe phenotypic features of delirium, for example, ‘agitated’, ‘disorientated’, ‘inattentive’, ‘pulling lines out’, ‘drowsy’, etc. Patients discharged on the day of surgery were assumed not to have delirium.

### Outcomes

The primary outcome was postoperative LOS, presented as both the 50th (median) and the 80th percentile. Although no single measure fully captures the success of a surgical management pathway, postoperative LOS encapsulates clinical, organisational, and social factors, all of which are influenced by frailty, multimorbidity, and postoperative complications such as delirium. The median LOS was chosen to reflect the average patient, and the 80th percentile to reflect metrics commonly used in monitoring of healthcare processes. Secondary outcomes were postoperative morbidity and mortality.

Postoperative morbidity was assessed using the PostOperative Morbidity Survey, excluding questions relating to delirium, with specific versions for cardiac surgery and those with hip fracture.^14^, ^15^, ^16^ Patients discharged on the day of surgery were assumed to have no clinically significant postoperative morbidity. Inpatients were followed up on postoperative days 3 and 7.

Postoperative mortality data were obtained from death registrations through the Office for National Statistics in England and Wales, National Records of Scotland (NRS), and local research teams (Northern Ireland). Mortality was assessed at 30 days, 120 days, and 1 yr.

### Covariates

Age was centred (age in years − 72 [median age in whole years]) and scaled (divided by 40 [approximate age range]) to aid model convergence for binary outcomes. A single eight-category nationality/deprivation variable was constructed by combining Index of Multiple Deprivation (IMD) deciles for those participants living within England into quintiles. Northern Ireland, Scotland, and Wales were each treated as a single category and not further divided because of small numbers in those countries. Biological sex assigned at birth, multimorbidity, dementia, malignancy, visual impairments, hearing impairments, assessment at preoperative clinic, and existence of postoperative medical service were recorded as binary variables. Physical status was categorised by ASA physical status from 1 (healthy) to 5 (moribund).^17^ Surgical urgency was categorised using National Confidential Enquiry into Patient Outcome and Death (NCEPOD) 2004 classifications (Emergency, Urgent, Expedited, Elective).^18^ Operative severity was defined by the ordinal AXA procedural codes, where subsequent categories indicate increasing complexity: Minor, Intermediate, Major, Extra Major (XMajor), Complex.^19^^,^^20^

Frailty status was evaluated using the CFS, with frailty defined as CFS ≥5 for descriptive analyses.^21^^,^^22^ In statistical models, frailty was treated as a five-point scale (CFS 1–3, 4, 5, 6, 7–8) for all outcomes except for 30-day mortality, where a three-point scale (CFS 1–3, 4–6, 7–8) was used owing to rare events in some categories. Five participants recorded as CFS 9 (terminally ill) were excluded from all analyses (Supplementary Fig. 1) to minimise risk of disclosure and modelling instability associated with very small groups.

Multimorbidity was defined as the presence of two or more specified comorbidities based on the Charlson Comorbidity Index and a recent Delphi study.^23^, ^24^, ^25^, ^26^ Our list of comorbidities included additional conditions relevant to the perioperative period not captured by the Charlson Comorbidity Index, such as obstructive sleep apnoea and atrial fibrillation (Supplementary Table 2 in previous publication^1^). Data on discharges and mortality were from population-based healthcare administration records from NHS Digital (England), Digital Health and Care Wales, the Office for National Statistics, and NHS National Services Scotland.

### Directed acyclic graphs

We developed directed acyclic graphs to define hypothesised relationships between delirium, outcomes, and related variables (Supplementary Figs 2–4). This ensured transparency in our assumptions and guided covariate selection for adjusted models based on an evidence-driven rationale. Details of this process and our decisions have been reported.^27^

All models adjusted for age, sex, area social deprivation/nation, frailty, multimorbidity, interaction between frailty and multimorbidity, dementia, malignancy, operative severity, surgical urgency, preoperative assessment clinic, and the existence of a postoperative perioperative medicine service within the treating hospital. LOS and mortality models also adjusted for postoperative morbidity. Mortality models also adjusted for visual impairments, hearing impairments, and polypharmacy.

### Statistical analysis

Details of the statistical analysis plan for the SNAP-3 study have been described,^1^^,^^7^, ^8^, ^9^ and the STrengthening the Reporting of OBservational studies in Epidemiology (STROBE) checklist was followed (Supplementary Table 1). All analyses were conducted in R (version 4.3.2; R Foundation for Statistical Computing, Vienna, Austria).^28^ In descriptive analyses, confidence intervals were obtained using bootstrapping.

The primary outcome, LOS, was modelled using quantile regression for the median and 80th percentile, and bootstrapping with 1000 samples to estimate standard errors in the presence of clustering by hospitals in the data. All other outcomes were binary and modelled using mixed effects logistic regression, with a random intercept for hospitals.

### Multiple imputation

Missing exposure and covariate data were handled using multiple imputation with chained equations under the assumption that data are missing at random conditional on all variables in the imputation model. For each of the three outcomes of interest, the imputation models included delirium (the exposure of interest), NHS trust, nation, morbidity, frailty, multimorbidity, sex, age, IMD decile, dementia, malignancy, visual impairment, hearing impairment, operative severity, preoperative assessment clinic, surgical urgency, polypharmacy, the existence of a postoperative perioperative medicine service, and the outcome of interest. Otherwise, the imputation procedures were developed as described,^1^ with details in Supplementary material 1. Twenty imputed datasets were analysed for each outcome; results were pooled using Rubin’s rules. For quantile regression, standard errors obtained from bootstrapping were pooled across imputations.

### Sensitivity analysis

We performed two additional sensitivity analyses. (i) In order to assess the sensitivity of our results to the missing at random assumption underlying multiple imputation, all models were estimated on complete data only. (ii) To investigate whether our estimates were sensitive to the inclusion of a low-risk (daycase) population, multiple imputation analyses were performed after restricting to inpatients only.

## Results

SNAP-3 recruited 7821 older surgical patients from 214 (∼81%) UK hospitals. Because of fluctuations in surgical activity during this period, it is not possible to accurately determine what proportion of eligible patients were included. After withdrawal of patients for reasons including postponement of surgery, not meeting inclusion criteria, and patient wishes, 7128 were included for analysis (Supplementary Fig. 1). The most frequently missing data point was complete assessments of all listed comorbidities (11.7% missing). In all remaining variables, <5% of data were missing (Supplementary Table 2). The mean age of the cohort was 72.8 yr (range: 60-101 yr), 50.9% were male, 35.6% were ASA physical status 3, and 3.7% were ASA physical status 4. Most (69.7%) participants underwent elective surgical procedures.^8^

Incidence of delirium among SNAP-3 participants was 6.7% (479/7128). Notes review identified an additional 48 cases not identified using 4AT or CAM-ICU at postoperative day 3 and 7. Incidence was greater in individuals living with frailty (CFS ≥5) at 20.5% (281/1369) compared with those without frailty at 3.5% (198/5628). Amongst those living with multimorbidity, incidence of delirium was 9.3% (371/3978), whereas in those without multimorbidity, it was 4.8% (92/2325). Delirium was present in 2.7% (134/4922) of those undergoing elective surgery *vs* 16.3% (348/2138) of those undergoing unplanned surgery.

Key characteristics of SNAP-3 participants with and without delirium are shown in Table 1. Supplementary Tables 3–7 provide detailed information on overall cohort characteristics, and on subgroups: aged ≥85 yr, aged <85 yr, living with frailty, not frail, elective, nonelective, and inpatients. Of participants treated as daycases but subsequently admitted, two were reported as having delirium.

**Table 1:** Characteristics of SNAP-3 participants with or without delirium. Percentages have been rounded so do not total 100% exactly. Missing data are omitted from this table but are reported in Supplementary Table 2. Surgical urgency is defined using National Confidential Enquiry into Patient Outcome and Death categorisations.^18^ The values here are percentages unless otherwise stated. Operative severity is defined by AXA procedural codes.^19^ ADLs, activities of daily living; CFS, Clinical Frailty Score; IQR, interquartile range.

Characteristic		All		Delirium		No delirium	
		(*n*=7128)		(*n*=479)		(*n*=6649)	
		*n*	%	*n*	%	*n*	%
Sex (*n*=7055)	Female	3464	49.1	253	52.8	3211	48.8
	Male	3591	50.9	226	47.2	3365	51.2
Age (yr) (*n*=7051)	60–69	2585	36.7	85	17.8	2500	38.0
	70–79	2836	40.2	144	30.1	2692	41.0
	80–89	1400	19.9	171	35.8	1229	18.7
	>90	230	3.3	78	16.3	152	2.3
ASA physical status (*n*=6994)	1	513	7.3	4	0.8	509	7.8
	2	3724	53.2	105	22.0	3619	55.5
	3	2488	35.6	288	60.3	2200	33.8
	4	259	3.7	79	16.5	180	2.8
	5	10	0.1	2	0.4	8	0.1
Surgical urgency (*n*=7055)	Emergency	160	2.3	30	6.3	130	2.0
	Urgent	1060	15.0	215	44.9	845	12.8
	Expedited	914	13.0	100	20.9	814	12.4
	Planned	4921	69.8	134	28	4787	72.8
CFS (*n*=6992)	1	808	11.6	15	3.2	793	12.2
	2	1351	19.3	35	7.4	1316	20.2
	3	2141	30.6	69	14.5	2072	31.8
	4	1328	19.0	79	16.6	1249	19.2
	5	696	10.0	100	21.0	596	9.1
	6	421	6.0	84	17.6	337	5.2
	7	222	3.2	81	17.0	141	2.2
	8	25	0.4	13	2.7	12	0.2
Frailty (CFS ≥5) (*n*=6992)	Frail	1364	19.5	278	58.4	1086	16.7
Dementia (*n*=7056)	Dementia	184	2.6	101	21.1	83	1.3
Multimorbidity (two or more comorbidities) (*n*=6298)	Multimorbid	3974	63.1	368	80.0	3606	61.8

		**Median**	**IQR**	**Median**	**IQR**	**Median**	**IQR**

Number of comorbidities (*n*=6298)		2.0	1.0–3.0	3.0	2.0–4.0	2.0	1.0–3.0

**Fig 1:**
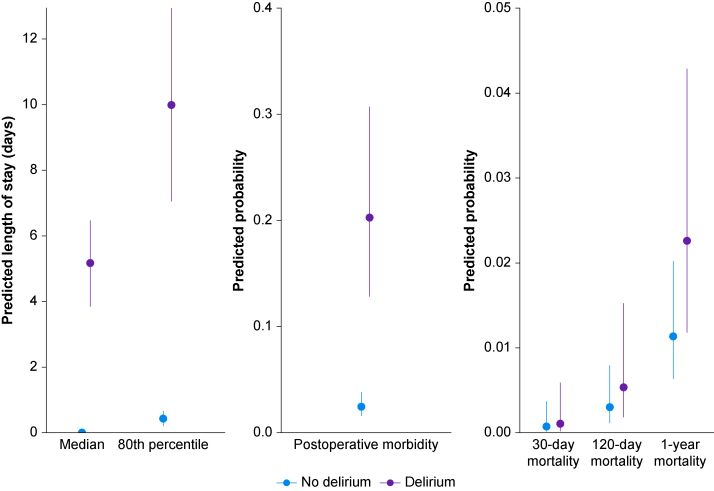
Predicted outcomes by presence of delirium. The reference groups used to calculate predictions vary by outcome (see Methods and Supplementary Table 8 legend for additional details). Note that y-axes scales differ between panels. This figure is supported by Supplementary Table 8.

Figure 1 presents predicted outcomes for participants with and without delirium (supported by Supplementary Table 8). Details follow for each outcome.

### Length of stay

Overall, median LOS was 1.0 day (interquartile range [IQR], 0.0–5.0). Median (IQR) LOS was longer in participants with delirium than those without (12.5 [7.0–22.0] *vs* 1.0 [0.0–4.0] days), with a similar difference for the subset of inpatients (12.0 [7.0–22.0] *vs* 3.0 [1.0–6.0] days). The unadjusted differences in median and 80th percentile LOS were 11.0 days (95% CI, 9.6–12.4) and 21.0 days (95% CI, 17.2–24.8), respectively.

There was strong evidence for an effect of delirium on both the median and the 80th percentile of LOS after adjustment for potential confounding. Delirium is estimated to increase median LOS by 5 days (95% CI, 3.8–6.5 days) and 80th percentile LOS by 10 days (95% CI, 6.6–12.5 days) (Table 2, Supplementary Tables 9 and 10).

**Table 2:** Postoperative outcomes for SNAP-3 participants with and without delirium. Postoperative morbidity includes PostOperative Morbidity Survey-defined morbidity and death, but excludes delirium. Summary statistics are presented as median (IQR), 80th percentile, or number and percentage. Sample sizes differ because of missing outcome data, which were not imputed. Unadjusted and adjusted effect estimates are presented as difference in days (95% CI) or odds ratios (95% CI). See the Methods for additional details. The full regression results are given in Supplementary Tables 9–14. CI, confidence interval; IQR, interquartile range; LOS, length of stay.

	Whole cohort		Delirium		No delirium		Unadjusted association: delirium *vs* no delirium	Adjusted association: delirium *vs* no delirium
Postoperative LOS (days) (*n*=6898)							**Centile difference (95% CI)**	**Centile difference (95% CI)**

Median (IQR)	1.0	(0.0–5.0)	12.5	(7.0–22.0)	1.0	(0.0–4.0)	11.0 (9.6–12.4)	5.2 (3.8–6.5)
LOS: 80th percentile	6.0		26.0		5.0		21.0 (17.2–24.8)	9.6 (6.6–12.5)

	***n***	**%**	***n***	**%**	***n***	**%**	**Odds ratio (95% CI)**	**Odds ratio (95% CI)**

Postoperative morbidity (*n*=7128)	1900	26.7%	416	86.8%	1484	22.3%	23.0 (17.7–30.4)	10.2 (7.4–13.9)
Mortality (30 days) (*n*=7029)	75	1.1%	27	5.7%	48	0.7%	8.1 (5.0–13.1)	1.4 (0.8–2.5)
Mortality (120 days) (*n*=7029)	212	3.0%	69	14.5%	143	2.2%	7.6 (5.6–10.3)	1.8 (1.2–2.6)
Mortality (1 yr) (*n*=7029)	485	6.9%	127	26.7%	358	5.5%	6.3 (5.0–7.9)	2.0 (1.5–2.7)

### Postoperative morbidity

Postoperative morbidity (excluding delirium) within 7 days of surgery occurred in 26.7% (1900/7128) of all participants. Incidence was quadrupled in those experiencing delirium, 86.8% (416/479), *vs* those without, 22.3% (1484/6650). The unadjusted odds ratio for developing postoperative morbidity was 23.0 (95% CI, 17.7–30.4).

After adjustment, delirium was strongly associated with increasing odds of postoperative morbidity. The estimated causal odds ratio for morbidity in those with delirium *vs* those without delirium was 10.2 (95% CI, 7.5–13.9) (Table 2, Supplementary Table 11). The predicted probability of postoperative morbidity for patients in the reference group was 20.3% (95% CI, 12.8–30.7%) in those with delirium *vs* 2.4% (95% CI, 1.6–3.8%) in those without delirium (Fig. 1, Supplementary Table 8).

### 30-day postoperative mortality

Incidence of 30-day mortality was 1.1% (75/7030) for the whole cohort, and was eight times greater in those with delirium (5.7%, 27/476) *vs* (0.7%, 48/6554) in those without. The unadjusted odds ratio for 30-day mortality was 8.1 (95% CI, 5.0–13.1).

After adjustment, delirium was weakly associated with increasing odds of 30-day mortality. The estimated causal odds ratio for 30-day mortality in those with delirium *vs* those without delirium was 1.4 (95% CI, 0.8–2.5), and thus there was no strong evidence from these data for an effect of delirium on 30-day mortality (Table 2, Supplementary Table 12). The predicted probability of postoperative mortality within 30 days for patients in the reference group was 0.1% (95% CI, 0.02–0.6%) in those with delirium *vs* 0.1% (95% CI, 0.02–0.4%) in those without delirium (Fig. 1, Supplementary Table 8).

### 120-day postoperative mortality

Incidence of 120-day mortality was 3.0% (212/7030) for the whole cohort, and was five times greater in those with delirium: 14.5% (69/476) *vs* 2.2% (143/6554) in those without. The unadjusted odds ratio for 120-day mortality was 7.6 (95% CI, 5.6–10.3).

After adjustment, delirium was moderately associated with increasing odds of 120-day mortality. The estimated causal odds ratio for 120-day mortality in those with delirium *vs* those without was 1.8 (95% CI, 1.2–2.6) (Table 2, Supplementary Table 13). The predicted probability of postoperative mortality within 120 days for patients in the reference group was 0.5% (95% CI, 0.2–1.5%) in those with delirium *vs* 0.3% (95% CI, 0.1–0.8%) in those without delirium (Fig. 1, Supplementary Table 8).

### 1-yr postoperative mortality

Incidence of 1-yr mortality was 6.9% (485/7030) for the whole cohort, and was five times greater in those with delirium: 26.7% (127/476) *vs* 5.5% (358/6554) in those without. The unadjusted odds ratio for 1-yr mortality was 6.3 (95% CI, 5.0–7.9).

After adjustment, delirium was moderately associated with increasing odds of 1-yr mortality. The estimated causal odds ratio of 1-yr mortality in those with delirium *vs* those without was 2.0 (95% CI, 1.5–2.7) (Table 2, Supplementary Table 14). The predicted probability of postoperative mortality within 1 yr for patients in the reference group was 2.3% (95% CI, 1.2–4.3%) in those with delirium *vs* 1.1% (95% CI, 0.6–2.0%) in those without delirium (Fig. 1, Supplementary Table 8).

### Sensitivity analyses

Sensitivity analysis restricted to complete records found similar results to the main analysis which used multiple imputation (Supplementary Table 15). Sensitivity analysis restricted to inpatients also found similar results to the main analysis on the full SNAP-3 cohort. Although the effect estimate for postoperative morbidity was reduced compared with the main analysis, there was still strong evidence of an effect of delirium in the inpatient subgroup (Supplementary Table 16).

## Discussion

In a heterogeneous cohort of older surgical patients from the UK, the overall incidence of delirium was 6.7%, with a higher incidence (16.3%) in those undergoing unplanned surgery. This is in keeping with other reports^29^, ^30^, ^31^, ^32^ despite the acknowledged limitations in accurately identifying a clinical condition characterised by a fluctuating course within the research setting. Within SNAP-3 participants, delirium was strongly associated with a prolonged LOS, postoperative morbidity, and mortality (up to 1 yr after surgery). The estimated average causal effects, adjusted for measured confounders, were somewhat weaker but still moderate or strong, with at best modest evidence for an effect of delirium on 30-day mortality.

The univariate relationship between in-hospital delirium and a range of adverse outcomes in both surgical and nonsurgical patients has been described^6^^,^^33^^,^^34^; failure to demonstrate this association would have raised questions about the SNAP-3 cohort data. Less clear is the extent to which delirium is a cause of adverse outcomes as opposed to a marker of a vulnerable surgical patient at risk of adverse outcomes. In the classical experimental paradigm, this might be investigated through a controlled trial. Given that such a trial is practically and ethically impossible, researchers must attempt to disentangle this relationship through observational research. Under certain assumptions, results from observation studies can be interpreted as causal effects. These key assumptions underlying causal inference are conditional exchangeability, positivity, and consistency.^35^ Conditional exchangeability means that, within subgroups defined by confounding variables, if the exposed group had in fact not been exposed, they would have had the same outcomes as the unexposed group and *vice versa*.^35^ Conditional exchangeability holds if our directed acyclic graph is correct and there are no unmeasured confounders. Positivity means that, in every subgroup defined by confounding variables, the probability of being assigned to an exposure group is never zero.^35^ So positivity holds if there is a non-zero probability in every subgroup of patients of having delirium and of not having delirium. Consistency means that there are not multiple versions of the exposure^35^; that is, delirium is the same for all patients who actually have it. Consistency is doubtful if, for example, patients could have more or less severe delirium, or different kinds of delirium with different aetiologies.

To get the best estimates of causal effects using the SNAP-3 data, we endeavoured to clarify hypothesised causal relationships, as illustrated in our directed acyclic graphs,^27^ and have interpreted our effect estimates as causal effects, assuming conditional exchangeability, positivity, and consistency. By being open and explicit about the assumptions we have made in order to interpret our regression results as causal effects (impacts) rather than associations, we welcome future critical assessments of our assumptions, and therefore our causal interpretations.

We undertook a rigorous and robust approach to our adjustment models, using directed acyclic graphs to direct our model choices.^27^ This approach visually depicts our assumptions about the relationships between variables, based on the literature, lived experience of surgery from public and patient representatives, and expert clinical knowledge of practicing geriatricians, surgeons, and anaesthetists. This guided variable selection for our models to deal with potential confounding whilst minimising the risk of over-adjustment (e.g. for variables acting as mediators for the delirium–outcome relationship). We included an interaction term between frailty and multimorbidity in our models, consistent with previous work where we allowed the effect of frailty on outcomes to vary depending on multimorbidity status and *vice versa*.^1^

Delirium is associated with prolonged LOS, likely attributable to the direct effects of delirium, association with other medical complications, and other predictors of longer LOS (age, cognition, frailty). This is an important effect for every healthcare system, regardless of funding model. However, crude estimates which overplay this relationship are unlikely to be helpful. We observed that, after adjustment, the impact of delirium on LOS was reduced from a difference in median LOS of 12.5 days to 5 days. Sensitivity analysis found similar results when restricting to inpatients. Such a difference is clinically relevant, as noted previously,^1^ where we reported that the impact of frailty (CFS 5) *per se* is ∼2 days and 5–7 days for the median and 80th centile of LOS, respectively.

The relationship between postoperative morbidity and delirium is complex. SNAP-3 was not designed to capture the temporal relationship between observation of delirium and postoperative morbidity. We are therefore unable to state with any confidence the direction of this relationship. We argue that better temporal data on when events occurred would be unlikely to further elucidate causation. Various nonexclusive scenarios can be envisaged. For example, a complication occurs (e.g. pneumonia) which is the precipitant of subsequent delirium. Providing pneumonia is diagnosed before delirium that temporal sequence makes sense, but if, as is frequently the case, pneumonia is diagnosed after delirium (perhaps because of appropriate investigation for likely precipitants of delirium,^36^ or because the pneumonia was not initially clinically apparent), the observed sequence of events does not match the presumed biology. More confusingly, pneumonia might be a consequence of (hypoactive) delirium, and so might be diagnosed before or after delirium. These scenarios illustrate the complexity, and near impossibility, of ascribing cause and effect to postoperative complications and delirium in the confounded world of clinical practice. We can be more confident that delirium and postoperative morbidity are strongly associated. From a clinical perspective, this is important. The presence of complications in the surgical patient should trigger vigilance for delirium, and if delirium is identified, causative postoperative complications should be proactively identified and managed.^36^

There was a strong univariate association between delirium and mortality at all measured time points. The relationship was weaker after adjustment and there was no evidence of a relationship for 30-day mortality. This finding is important as our results are based on prospective granular data collection, and predefined directed acyclic graphs to ensure appropriate adjustment for measured confounders. Previous studies in the surgical population have highlighted the strong association between delirium and mortality.^6^ However, systematic reviews suggest that risk of bias due to confounding in these data is high, and that the mortality risk is not present when adequate adjustment is carried out.^34^ We found that the relationship between delirium and mortality was inconsistent over time. Our data suggest that for longer time points, delirium is independently associated with mortality, with an estimated causal odds ratio of around 2. Our finding of no evidence of an association between delirium and 30-day mortality supports the work of Pedemonte and colleagues^37^ in hip fracture where, with a well-adjusted model, the impact of delirium was only observed in later time points. This finding is also in keeping with other literature suggesting that as control for confounding improves, the effect of postoperative delirium on mortality becomes smaller than previously reported.^34^ Additionally, a relatively low event rate in the early postoperative period and the inclusion of delirium as a binary variable not accounting for the severity of delirium may be relevant. Possible aetiological explanations include delirium accelerating or causing cognitive decline, with the role of neurofilament light chain in the brain resulting from delirium a potential causative factor.^38^ Increased surveillance in the early postoperative period may also play a role.

Evidence-based nonpharmacological multicomponent strategies to reduce the incidence of delirium exist.^39^, ^40^, ^41^ Screening for high-risk patients combined with multicomponent interventions is cost-effective, although this is mediated through impacts both within hospital and in the community.^42^ Furthermore, screening for delirium using a brief assessment tool such as 4AT can have additional benefits in identifying unrecognised dementia with relevance to perioperative decision-making and longer-term management.^43^

There are strengths and limitations to the SNAP-3 cohort and analysis. SNAP-3 recruited from almost every UK hospital using prospective data collection. This makes it less susceptible to selection bias by type of hospital, patient characteristics, and surgical specialty, which inevitably exist in non-national studies. However, not every eligible patient would have been recruited, so the possibility of meaningful nonrandom missingness exists. Our primary exposure for this analysis, delirium, is difficult to reliably capture at scale because of fluctuation and missed cases. We mitigated against this with direct observation on two occasions, supplemented with retrospective notes review noting that the resource limitations in SNAP-3 as with other studies will have resulted in false negatives. However, although we will have missed some episodes of delirium, our estimated rates are in line with other studies,^29^, ^30^, ^31^ suggesting that the approach adopted was suitably robust. These inherent and acknowledged issues in identifying a fluctuating condition in the research setting is mirrored in the limitation to clinical identification in usual practice.

Because of the relatively small numbers of delirium episodes identified by methods other than 4AT, we did not attempt to account for the method of assessment in the modelling. It is possible that different approaches to delirium identification might have given quantitatively different results, although we think the differences are likely to be of little clinical significance. In the UK, 4AT is the most commonly used tool in clinical practice,^44^ which supports generalisation of our findings. Despite being a large cohort, analysis at the subgroup level is limited because of small numbers.

Although our results come solely from the UK NHS, we believe they are likely to be generalisable to other developed healthcare settings where populations and perioperative management are similar.

All observational studies are at risk of confounding by measured and unmeasured factors. Most studies reporting the association between delirium and postoperative outcomes make little, if any, adjustment, and those that do find much smaller effects or no evidence of an effect. We developed clinically informed causal models (directed acyclic graphs) to inform statistical adjustment.^27^ Although we believe this to be robust, clinicians and researchers have differing views on what should be in those models. No matter how robust our model, we are unable to adjust for unmeasured confounders, for example, causes of delirium not currently routinely assessed such as cognition, predisposing factors such as alcohol intake, and precipitating factors such as causative medications or pain. The independent impact of delirium might therefore be greater or lesser than our estimates suggest.

There are important clinical, organisational, and research implications of our findings. Notwithstanding its independent association with adverse outcomes, delirium is frequently recalled, and is distressing for patients, families, other patients, and staff.^45^ Delirium is common across all subgroups of older people having surgery, and all clinicians involved in the perioperative pathway have a responsibility to assess elective surgical patients for risk of delirium, factor it into shared decision-making, warn patients of this risk, use mechanisms to reduce the incidence and severity of delirium, and proactively identify and treat delirium if it occurs.^46^^,^^47^ The findings from our parallel survey of perioperative services related to frailty and delirium in the UK and Ireland suggests that this is not consistently being achieved.^44^ As the surgical population ages, healthcare needs to provide evidence-based, cost-effective, and age-attuned services tailored to patient needs.

We have deliberately reported the cross-tabulated data on patient characteristics, clinical characteristics, and outcomes in full in the Supplementary material. These data can provide contemporary estimates of delirium and outcomes in relevant groups to inform future interventional trials or quality improvement. Research at the fundamental science and implementation stages is needed to better understand the underlying pathophysiology of delirium, identification of those at risk and potential pharmacological, physical, and organisational approaches to preventing or mitigating postoperative delirium, together with better cataloguing the longer-term implications. Only when the results of this study and any future interventional work for quality improvement are routinely considered can a true perioperative and patient-centred approach be established.

## Authors’ contributions

Project initiation, guarantor, and grant holder: IKM

Patient-facing document design and patient-public insights: BE, CJS, JSLP, IKM

Ethical and regulatory approvals: CJS, KW

Data collection tools and protocol design: CJS, JSLP, IKM, TP, AS

Study implementation in the UK: CJS, KW, IKM, JSLP

Site and UK-wide activity coordination: KW, CJS

Analysis plan development: IKM, PM, HAB, CJS

Monitoring data collection: CJS

Finalising data collection: SN

Statistical expertise in study design and analysis methods: PM, HAB

Data cleaning: CJS, IKM

Data analysis: CJS, HAB, IKM, PM

Review of final draft and contributions to revisions: all authors

## Funding

Frances and Augustus Newman Foundation and the Royal College of Anaesthetists; National Institute for Health and Care Research ARC North Thames (HAB).

## Declarations of interest

IKM is Chair of the Centre for Research and Improvement at the Royal College of Anaesthetists. IKM, AS, and JP have received grant funding for clinical trials in perioperative care of older people. IKM’s department has received consultancy fees from Astra Zeneca for work unrelated to the submitted work. AS has received honoraria from Pharmacosmos UK outside of the submitted work. The other authors declare that they have no conflicts of interest.
