# Supplementary material for: Postoperative outcomes in older patients with postoperative delirium in the UK: SNAP-3, a snapshot observational study
Source: Br J Anaesth. 2026 Feb 26;136(5):1578–87. doi: 10.1016/j.bja.2026.01.030 (PMC13197927; doi:10.1016/j.bja.2026.01.030)
Supplement: Supplementary file 1 [file mmc1.pdf]

Postoperative outcomes in older patients with postoperative delirium in the UK: SNAP-3, a snapshot observational study

Supplementary table 17: SNAP-3 collaborators

A list of collaborators to be recognised for the input into SNAP-3.

Organisation	Collaborators
**Addenbrookes Hospital**	Alexandre Field
Principal Investigator Svet Petkov	Amit Deshmukh
	Amy Frayling
	Andrea Ortu
	Bogdan Petrisor
	Chamika Abayasinghe
	Charles Noonan
	George Couch
	Harsh Nimaiyar
	Israa Elfouli
	Joanne Outtrim
	Katharina Kohler
	Konul Hajiyeva
	Laura Graham
	Mark Forth
	Mark Gotecha
	Nader Alrefaii
	Poppy Aldam
	Priti Morzaria
	Rachel Ariyanayagam
	Rajan Mehta
	Reece Cordy
	Samudu Wahappulige
	Sandeep Sharma
	Sanika Patil
	Shayan Arshed
	Sonia Boktor
**Aintree Hospital**	Antony Berridge
Principal Investigator Dermot Moloney	Bisanth Batuwitage
	Chris Marsh
	Claire Davis
	Dermot Moloney
	Ellie Quilliam
	Ellie Quillium
	James Cassidy

	James Pratt
	Laura Bridge
	Matthew Palethorpe
	Melanie Harrison
	Michelle Linforth
	Nadim Kozman
	Nicki Russell
	Saskia Van
	Shirley Cooper
**Airedale General Hospital**	Amy Kitching
Principal Investigator Tamsin Gregory	Andrew Pearson
	Chantel Mcparland
	Emma Dooks
	Lisa Armstrong
	Tamsin Gregory
	Patrick Winstanley
**Arrowe Park Hospital**	Abigail Carey
Principal Investigator David Blackman	Billy Holt
	David Blackman
	Callum Hammond
	Florence Wilson
	Julie Grindey
	Kieran Walker
	Lewis Mcintyre
	Molly Wyche
	Natalie Ash
	Sam Freeborn
	Siofra Daly
	Taqua Omer
	Tilemachos Zaimis
**Ashford and St Peters Hospital**	Ana Glennon
Principal investigator Mark Macgregor	Caroline Ellis
	Caroline Pocknall
	Gayatri Saxena
	Isaac John
	Jigneshkumar Patel
	Kashif Jabbar
	Keshnie Govender
	Louise Renouf
	Lynn Drummond
	Margaret Dsouza
	Margaret Walsh
	Marie Buckley
	Mark Macgregor
	Matilda Ravidram
	Meera Nadir

	Rita Pereira
	Seliat Sanusi
	Sophia Khaleeq
	Thomas Hall
**Barnet General Hospital**	Ailidh Lang
Principal Investigator Claire King	Ben Tilley
	Claire King
	Emily Lachmann
	Keerthi Senthil
	Lucy Owen
	Nael Alavi
	Su Ling
**Barnsely Hospital**	Abigail Crewe
Principal Investigator Sunil Chaurasia	Alice Nicholson
	Anna Galvin
	Christopher Harrison
	Emma Stoner
	Jenny Mullan
	Jodie Macdonald
	Lisa Proudfoot
	Natalie Lake
	Nicola Lancaster
	Ruth Nanda
	Sarah Cuts
	Sunil Chaurasia
	Susan Hope
	Wei Low
**Basildon University Hospital**	Angelo Ramos
Principal Investigator Pallavi Marghade	Angukumar Thangamuthu
	Anne Nicholson
	Chanaka Karunaratne
	Claire Mccormick
	Emily Redman
	Gladys Emmanuel
	Jean Byrne
	Joanne Riches
	Jo-anne Cartwright
	Jonaifah Ramirez
	Kai Li
	Kelly Musson
	Kerry Goodsell
	Kriti Vig
	Luke Hounsom
	Lushen Pillay
	Miranda Forsey
	Moroti Abioye

	Muhammed Faheem
	Pallavi Marghade
	Samuel Rowe
	Sofia Alexandra
	Sohail Omar
	Stacey Pepper
	Steph Gumus
	Vivek Sharma
**Bedford Hospital**	Anisha Roopram
Principal Investigator Peter Knowlden	Joshua Mcgillicuddy
	Melchizedek Penacerrada
	Muhammad Haseeb
	Peter Knowlden
	Rachel Lorusso
	Ridwan Sharif
	Ruby Fronda
**Birmingham Heartlands, Good Hope and Solihull Hospitals**	Aaron Klair
Principal Investigator Joyce Yeung	Abdul Hai
	Akhil Chellapuri
	Alexandra Timperley
	Ali Abusbaeh
	Annapoorani Subramani
	Ayesha Khalid
	Ayisha Afzal
	Caitlin Stevens
	Christina Thomas
	Ciara Gibson
	Dan Newport
	Daniel Lenton
	David Brennan
	Deepika Chivukula
	Dineshwaran Rajendran
	Eleanor Reeves
	Emily Butler
	Farhaana Surti
	Faye Moore
	Georgina Kelly
	Girgis Awadalla
	Harry Bhalla
	Hassan Abdullah
	Heather Willis
	Hnin Haymahn Htun
	Hoi Yan
	Joanne Gresty
	Joseph Son-leong

	Joseph Leong-son
	Joyce Yeung
	Juliet Sebastian
	Kiren Parkash
	Komal Bodhke
	Kristina Gallagher
	Maahi Qureshi
	Mable Kurian Chalil
	Mahmoud Khedr
	Manahil Bashir
	Maria Ghani
	Marwel Alyssa Jones
	Mary Bellamy
	Matt Beck
	Matthew Sibley
	Merna Ebrahim
	Mike Seyani
	Mirriam Sangombe
	Mohammed Al-zubayel
	Muhammad Abdulaziz
	Muhammad Ansari
	Muhammad Mujeeb
	Murshid Ali
	Murshid Ali Mohamed Maheen
	Olivia Babaua
	Patience Juru
	Preethi George Pandeth
	Rana Said
	Rebecca Hatchard
	Rehman Crisp
	Rochelle Velho
	Ruth Joslyn
	Safwaan Patel
	Sam Stafford
	Sant Leelamanthep
	Sarang Pathak
	Sharvari Mahajan
	Sharvari Vadeyar
	Sunera Khan
	Suvojit Misra
	Syra Kazmi
	Tammy Bellamy
	Teresa Melody
	Yash Tyagi
**Birmingham Women's Hospital**	Clare Mcpake
Principal Investigator Jane Pilsbury	Faye Andrews

	Jane Pilsbury
	Parminder Chana
	Rosy Dunham
	Samantha Bull
	Kerry Cullis
	Sandeep Kapur
	Lyndon Harkett
	Emma Plunkett
	Tim Molitor
**Blackpool Victoria Hospital**	Alanna Beasley
Principal Investigator Anwar Ulhaq	Amy Barnett
	Andrea Cinconze
	Andrew Donohue
	Aniko Babits
	Anukiran Ravichandran
	Anwar Ulhaq
	Ashleigh Wignall
	Audrey Hellen
	Barbara Lord
	Carol Jeffs
	Deepa Sebastian
	Denise Bennett
	Dzmitry Zabauski
	Emma Ward
	Hannah Walsh
	Jayne Windebank
	Joanna Brown
	John Barrett
	Julia Mason
	Karen Williams
	Katherine Finch
	Kayleigh Mountford
	Kiran Nadiger
	Leonie Benham
	Lisa Elawamy
	Marium Khan
	Melanie Caswell
	Natalie Irvine
	Pinar Sheard
	Robert Davidson
	Samuel Remnant
	Sarah Traynor
	Scott Warden
	Senka Baranovic
	Shamina Hankinson
	Steph Reed

	Stephen Davies
	Stephen Preston
	Steve Wiggans
	Steven Dixon
	Vasanthi Vasudevan
	Victoria Cunliffe
	Zena Bradshaw
**Borders General Hospital**	Chengyuan Zhang
Principal Investigator Stephen Alcorn	Ella Bennett
	Harriet Briggs
	Heather Matthews
	Katie Stewart
	Michael Wild
	Nick Spencer
	Nicola Goldmann
	Peter Carson
	Rachel Harvey
	Stephen Alcorn
	Vanessa MacKenzie
**Bradford Royal Infirmary**	Edward Tam
Principal Investigator Robert Spencer	Estelle Tan
	Louise Akeroyd
	Michael Tattersfield
	Michael Kitchen
	Muhammad Haider
	Robert Spencer
	Robert Palin
	Sangavy Loganathan
	Satish Bharti
	Simon Cousins
	Thomas Knapp
**Bristol Royal Infirmary and St Michaels Hospital**	Amy Ashford
Principal Investigator Hannah Wilson	Andy Bartlett
	Angeliki Kolovou
	Anna Simpson
	Annie Wood
	Becky Woolf
	Chintan Vora
	Denise Webster
	Georgia Efford
	Hannah Wilson
	Henry House
	Josephine Bonnici
	Kathleen Corcoran
	Katie Pass

	Katie Sweet
	Kim Wright
	Mike Peacock
	Neil Choudhuri
	Patrick Liddicoat
	Rachael Meredith
	Richard Cassar White
	Steph Brown
	Suzi Braggins
	Tom Woodland
	Will Gatfield
**Bronglais General Hospital**	Gabor Dudas
Principal Investigator Gabor Dudas	Heather Mcguinness
	Ronda Loosley
	Tanya Sims
**Broomfield Hospital**	Amanda Lyle
Principal Investigator Al Hughes	Anna Beckwith
	Caroline Fox
	Al Hughes
	Christina Williams
	Conor Barrett Nnochiri
	Elizabeth Dawson
	Joanne Wootton
	Karen Cranmer
	Katherine Rao
	Lauren Sach
	Lorraine James
	Lucy Willsher
	Lucy Westcott
	Martina Vitaglione
	Nicola Cutmore
	Nicola Bulleta
	Nikolett Hunyadvari
	Rachel Arnold
	Sharon Reid
	Stacey Cotterell
	Tracey Camburn
	Victoria Apps
	Yvonne Lester
**Buckinghamshire Healthcare Trust**	Abdullah Alkhudhayri
Principal Investigator Tamsin Mcallister & Jeremy Drake	Adam Taylforth
	Adrianna Zembrzycka
	Ajay Sanghvi
	Alice Ngumo
	Anita Cserbane

	Aruna Naire
	Asia Joseph
	Bernard Nyemitei
	Bethan Davies
	Bobbie Sanghera
	Geraldine Hambrook
	Gregory Manning
	Hannah Catton
	James Goetz
	James Winchester
	Jeremy Drake
	Jonathan Blake
	Lucy Godfrey
	Maja Rakic
	Natalie Smith
	Paula Jenkins
	Ruth Penn
	Saba Syed
	Saranya Thurairatnam
	Sonia Mariampillai
	Sophie Jackman
	Tamsin Mcallister
	Victoria Whittaker
**Calderdale and Huddersfield FT**	Aaquid Akram
Principal Investigator Sophie Lawton & Pnt Laloe	Abigail Atkin
	Alice Wheeler
	Andrew Haigh
	Ben Green
	Bethany Spencer-lane
	Eve Braithwaite
	Harriet Watson
	Hedd Carden
	Jack Hogg
	Jason Auguste
	Jessica Morgan
	Jithu Jayan
	Jodie Bellwood
	Matthew Robinson
	Pnt Laloe
	Sam Doyle
	Sophie Lawton
**Charing Cross Hospital**	Ajantheny Naguleswaran
Principal Investigator Kenneth Murray	Ashley Guilliam
	Dharshini Rajasooriyer
	Elaine Viloria
	Patrick Daly

	Samuel Mindel
	Stephanie Ivie
	kenneth Murray
	Vidhya Nagaratnam
**Chelsea and Westminster Hospital**	Alex Schoolmeesters
Principal Investigator Marcela Vizcaychipi	Anan Bomfim
	Carina Bautista
	Eleanor Giles
	Elena Noval
	Emily Futter
	Florison Canlas
	Jaime Carungcong
	Kribashnie Nundlall
	Laura Martins
	Leah Flores
	Marcela Vizcaychipi
	Mari Rose
	Patricia Costa
	Rhian Bull
**Chesterfield Royal Hospital**	Adam Mendelski
Principal Investigator Julie Hui	Amanda Whileman
	Cindy Cart
	Claire Sampson
	Elizabeth Blythe
	Emily Jolly
	Emma Moakes
	Julie Hui
	Katarina Djapic
	Kelly Pritchard
	Lauren Bishop
	Leanne Lowe
	Lesley Stevenson
	Linda Bishop
	Mary Kelly-baxter
	Nicky Ford
	Rachel Gascoyne
	Rheanna Smith
	Roshani Deorukhkar
	Sarah Broadhead
	Stephanie Wright
	Thomas Hughes-gooding
**Colchester Hospital**	Alison Ghosh
Principal Investigator Joanna Simpson	Alison O'kelly
	Anita Immanuel
	Ashley Elden
	Bindiya Shah

	Celine Driscoll
	Devraj Kathwadia
	Edyta Klata
	Emma Williams
	Hazel Yeoh
	Hnin Shwe
	Jennifer Abaddulay
	Joanna Simpson
	Justyna Kapera
	Kali-jade Gunfield
	Katrina Cooke
	Marianne Morgan
	Michelle Dotchin
	Nyasha Nago
	Samuel Rowles
	Sara Scott
	Tracy Abery
**Countess of Chester Hospital**	Alex Moore
Principal Investigator Woei Lin Yap	Chloe Haylett
	Chloe Donaldson
	Conor Steele
	Hannah Mulgrew
	Imogen Watkins
	Kieran Kelly
	Laura Wilson
	Naomi Slater
	Nick Roberts
	Woei Lin Yap
	Yahya Abdullah
**Croydon University Hospital**	Agnes Fong
Principal Investigator Agnes Fong	Alexander Foreman
	Ashok Sundar
	Christopher Cregg
	Darren Caldow
	Jennifer Haugh
	Laura Ashton-Edwards
	Natashia Schneider
	Rana Mallah
	Reena Khade
	Sheun Fang Cheng
	Sonali Mohite
	Sonia Rasoli
	Vasileios Bafitis
**Cumberland Infirmary, Carlisle**	Emma Mawson
Principal Investigator Geetanjali Verma	Geetanjali Verma
	Laura Chapman

	Theresa Cooper
**Darent Valley Hospital**	Asad Zafar
Principal Investigator Mansoor Sange	Bridget Fuller
	Caspar Briault
	Conor Walsh
	Daniel Lake
	Ee Lyn Chan
	Georgia Monantera
	Hoda AbouGhoneim
	Mansoor Sange
	Matthew Smith
	Medappa Kaliyanda
	Naomi Oakley
	Nesma Abdelaziz
	Olumide Olufuwa
	Rajendra Pun
	Sara Yousaf
	Sean Cardoso
	Sean Warburton
	Shamini Sivakumaran
	Sophie Hill
	Sze So
**Darlington Memorial Hospital**	Amanda Cowton
Principal Investigator Victoria Craig	Andrew Shepperson
	Christopher Taylor
	Clare Hutton
	David Ginty
	Raviprakesh Hodigere
	Roshan Sebastian
	Sally Roscoe
	Sanjeev Kumar
	Victoria Craig
	Zachary Bowen-Davies
**Derriford Hospital**	Alice King
Principal Investigator Gavin Werrett	Alina Van-Hien
	Amy Turner
	Andy Savva
	Anna Ratcliffe
	Anneliese Crome
	Chris Gordon
	Elaine Jones
	Emily May
	Emma Bishop
	Fiona Reed
	Gary Minto
	Gavin Werrett

	Gunarathna Perumbadage
	Helen Anderson
	Holly Notman
	Jennifer Moran
	Juleen Fasham
	Julie Alderton
	Karen Friendship
	Kelly Whitehorn
	Lorraine Madziva
	Louise Jose
	Lucy Guile
	Martin Mills
	Memory Mwadeyi
	Natasha Wilmshurst
	Rebecca Allott
	Shivesh Tewari
	Shun Yamanaka
	Tracey Ward
	Will Hare
	William Peagam
**Doncaster Hospital**	Alasdair Strachan
Principal Investigator Alasdair Strachan	Angela Waddingham
	Emily Hall
	Fiona Dunning
	Gemma Rook
	Kerry Dooley
	Lisa Warren
	Rebecca Pugh
	Sarah Farmer
	Thomas Bidmead
	Vikki Cooke
**East Surrey Hospital**	Antony Ratnasingham
Principal Investigator Anthony Ratnasingham	Edward Combes
	Ellen Jessup-dunton
	Emily Watts
	Gail Murphy
	Indhuja Rajkumar
	Julie Houghton
	Leigh-james Spurling
	Lisa Clutterbuck
	Louise Nimako
	Merlin James
	Michele Poole
	Nancy Jones
	Ruth Habibi
	Sallyanne Trotman

	Samantha Weller
	Sarah Davies
	Sophie Beverley
	Sophie Holden
	Valerie Beech
	Yvonne Izzard
**Eastbourne District General Hospital**	Amr Elmosalamy
Principal Investigator Tara Bolton	Charlotte Crossland
	James Hartley
	Janet Sinclair
	Matthew Farrant
	Nicola Deacy
	Paul Jackson
	Penny Boxall
	Ross Holcombe-law
	Sang Yob
	Tara Bolton
	Thomas Kavanagh
	Timothy Faccini
	Toni De Freitas
**Edinburgh Royal Infirmary**	Ajit Singh Obhrai
Principal Investigator Thomas Ballantyne	Anna Te
	Arlena Kuenzel
	Bartlomiej Ordys
	Elizabeth Steel
	Emma Mann
	Flora Mclennan
	Kirsten Reid
	Kwun Chan
	Lisa Dewar
	Michael Robson
	Mysoon Alabdah
	Naomi Hyndman
	Rosanna Seatter
	Rosemary Mudie
	Sarah Scott
	Thomas Ballantyne
**Epsom and St Helier University Hospitals**	Abi Coe
Principal Investigator Martin Akioyame	Adil Hussain
	Agyapong Ansu
	Ahmed Eid
	Alice Hipsey
	Alice Pandaan
	Analyn Alipustain
	Anna Cebula
	Anna Forbes

	Asmaa Hassan
	Beatrix Sari
	Dipak Niroula
	Eva Garcia
	Harriet Asquith
	Irrum Afzal
	Jayadeep Sandhu
	Jenny Hisole
	Joe Joseph
	Kimberly Edgerton
	Krystie Vedat
	Kulasekar Kaliappan
	Laijee Benny
	Luke Parker
	Mahmoud Elhefnawy
	Mahmoud Hassanin
	Martin Akioyame
	Mary Madden
	Maureen Estrada
	Neringa Vilimiene
	Niketa Shukla
	Niketa Shukla
	Noor Ul Islam Syed
	Osatohanmwen Osagie
	Rachel Hayre
	Rosavic Chicano
	Sandhya Anandkumar
	Saurabh Kakkar
	Serah Duro
	Sharad Sinha
	Sonia Andleeb
	Suman Biswas
	Thomas Craven
	Tina Raju
	Uju Roskon-rapu
	Wadzanai Mupaya
**Glangwili General Hospital**	Abdul Rahim Ali Bakhsh
Principal Investigator Peter Havalda	Adam Ryan
	Barnaby Hart
	Becky Icke
	Dylan John
	Jill Williams
	Linda O'brien
	Lucy Hill
	Michael Coulton
	Peter Havalda

	Samantha Coetzee
**Glasgow Royal Infirmary and Stobhill Ambulatory Care Hospital**	Chris Hay
Principal Investigator Malcolm Howell and Sonya Mckinlay	Eilidh Lynch
	Emma Bhatti
	Gemma Scotland
	Khalid Boussouara
	Malcolm Howell
	Marielle Li
	Mykhaylo Shumeyko
	Roshan Saleh
	Ryan Murphy
	Sarah Barton
	Sonya Mckinlay
	Susan Speirs
	Susanne Cathcart
**Glenfield Hospital**	Adela Dobru
Principal Investigator Rahil Mandalia	Aqsa Haq
	Emily Rich
	Frances Tait
	Hayun Lee
	Jaimin Arya
	Joanna Shak
	Jun Tan
	Kathleen Wolff
	Pitchayud Kantachuvesiri
	Rajani Annamaneni
	Rohan Babla
**Gloucestershire Hospital NHS FT**	Alex Christie
Principal Investigator Henry Murdoch	Artemis Prevot
	Benedict Shinner
	Charlotte Bestwick
	Daniel Liu
	Deborah Ward
	Duncan Castle
	Elena Teh
	Ellie Courtney
	Emily Farrow
	Fiona Macrae
	Gemma Gardner
	Gemma Gardener
	Helena Veck
	Henry Murdoch
	Hiromi Uzu
	Isabel Evans

	Jayne Evitts
	Joanne Waldron
	Kayleigh Collins
	Kirsty Benton
	Matthew Townsend
	Maximo Clark
	Ozzie Zaidi
	Pauline Brown
	Reggie Gray
	Rhiannon Tanner
	Sophie Turnton
	Susan O'connell
	Tushar Rakhecha
	Victoria Gaunt
**Great Western Hospital**	Abbie Poole
Principal Investigator Mala Greamspet	Catherine Novis
	Mala Greamspet
	Charlotte Hunt
	Dominic Bashford
	Helen Langton
	Jacinta Ugoji
	James Sharples
	James Brooks
	Joe Stevens
	Laura McAfferty
	Maggie Ryder
	Nicholas Budhram
	Rachel Lyons
	Rebecca Mairs
	Robert Coe
	Tracey Benn
**Guys and St Thomas**	Abegail Salvana
Principal Investigator Kariem El-boghdadly	Alexander Ware
	Andrew Williams
	Andrew Wilson
	Anthi Andrianou
	Azka Afzal
	Bhavini Shah
	Danny Wong
	David Hutchinson
	Eleni Eleni
	Gary Colville
	Gill Arbane
	Jack Jack
	John Paul Mcnally-reilly
	Joseph Hetherington

	Kariem El-boghdadly
	Liana Zucco
	Maame Adesu Poku
	May Rabuya
	Naina Mohan
	Paul Morillon
	Phoebe Scarfield
	Rachel Babic
	Rosalind Rosalind
	Rupert Mason
	Saif Ahmed
	Sanna Khawaja
	Sara Ko
	Sarah Eshelby
	Sherpal Singh
	Sofiane Kouadria
	Thomas Potter
	Thwe Han
**Hammersmith Hospital**	Amitav Philip
Principal Investigator Kenneth Murray	Frances Garrick
	Joshua Singleton
	Peter Zsinko
	Slawomir Jaszczuk
	Uchechi Nwangama
**Harrogate District Hospital**	Abhinav Kant
Principal Investigator Abhinav Kant	Ahmed Aboughazy
	Caroline Bennett
	Karim Elfaham
**Hillingdon Hospital**	Geraldine Landers
Principal Investigator Myra Malik and Robert Crooks	Melinda Holden
	Myra Malik
	Natasha Mahabir
	Robert Crooks
	Stephen John
**Homerton University Hospital**	Amrutha Vishwanathan
Principal Investigator Christian Schwiebert	Christian Schwiebert
	Deepthy Pillai
	Eftychia Sousi
	Eleanor Taylor
	Jagrul Miah
	Jenny Olsson
	Pramitha Chinduluri
	Rose English
	Samuel Naylor
	Saqib Khawaja

	Srinivasan Perumal
	Tay-yibah Mohamed
	Zain Syed
**Hull Royal Infirmary**	Abigail Lau
Principal Investigator Andrew Gratrix	Adam Walker
	Andrew Gratrix
	Barbara Ribeiro
	Christopher Macrow
	Elizabeth Stones
	Emily Frost
	Esme Ward
	Eugene Ndimele
	Hannah Lauder
	Harriet Van
	Hull Research
	Jakub Kazda
	Jennifer Chalmers
	Jonathan Payne
	Jonathan Pyatt
	Jookyung Park
	Karen Winter
	Llucia Cabral-ortega
	Matthew Hines
	Mithilia Govind
	Molly Janowski
	Nicola Staples
	Prakash Subramaniam
	Raquel Costantino-duarte
	Rebecca Ireson
	Stephanie Bailey
	Stephen Mcaleer
	Vicky Martinson
	Llucia Cabral-Ortega
**Ipswich Hospital**	Aleix Ugalde
Principal Investigator Elizabeth Speirs	Carol Buckman
	Cathleen Chabo
	Ceren Driver
	Charlotte Mathur
	Daniel Watkins
	Daniel Waldschutz
	Deborah Beeby
	Elizabeth Speirs
	Genessa Peters
	Georgina Gray
	Jenny Finch
	Laurence Suckling

	Nipun Mundkur
	Rebecca Francis
	Stephanie Bell
	Suchona Hafiz
	Teresa Theobald
	Vanessa Rivers
	Victoria Fernando
**James Paget University Hospital**	Amanda Ayers
Principal Investigator Sudha Garg	Amy Garrod
	Charlotte Kelly
	Christian Alcock
	Christian Hacon
	Daniel Sciberras
	Darylile Guledew
	Donna Griffiths
	Elva Wilhelmsen
	Helen Sutherland
	Julie North
	Kevin Howard
	Lisa Hudig
	Pablov Zamora
	Pablov Zamora
	Sarah Daniels
	Sophie Cook
	Sudha Garg
	Syed Mazhar
	Wendy Harrison
	Zainab Najim
**Kent and Canterbury Hospital**	David Loader
Principal Investigator Srdjane Trajkovic	Gemma Hector
	Madhushika Dayarthne
	Rachel Vernall
	Srdjane Trajkovic
	Ritoo Kapoor
**Kettering General Hospital**	Alistair Thomas
Principal Investigator Satya Jakkampudi	Alvin Lau
	David Jarrold
	Foteini Christodouli
	Gayan Dissanayake
	Haider Zahur
	Julie Sebastian
	Lauren Hunt
	Paul Swift
	Satya Jakkampudi
	Su Vern Lim
	Tony Talhat

**Kings College Hospital, Denmark Hill**	Amy Dukoff-gordon
Principal Investigator Ravi Bhatia	Andy Chu
	Anna Naito
	Bethan Iikponmwosa
	Hamish Baillie
	Jennifer Berg
	Natasha Amaradasa
	Ravi Bhatia
	Sanjoy Bhattacharyya
**Kingston Hospital**	Camilla Paget
Principal Investigator Sarang Puranik	Elizabeth Evans
	Lucy Studd
	Lydia Weiss
	Sarang Puranik
**Lancashire Teaching Hospitals**	Abdelaziz Abdelaziz
Principal Investigator Zara Townley, Arumugam Pitchiah	Ailsa Watt
	Alexandra Williams
	Amanda Alty
	Angela Yan
	Anil Kumar
	Arumugam Pitchiah
	Benjamin Stewart
	Joy Hirst
	Louis Turrell
	Mark Verlander
	Michael Roach
	Nadeem Jamal
	Nadia Paes
	Sandra Sowden
	Shalil Henderson
	Shivani Gulati
	Sourav Mahajan
	Thomas Bradley
	Zara Townley
**Leeds Teaching Hospital NHS Trust**	Aneesha Qadeer
Principal Investigator Simon Howell	Antonio Borrelli
	Beverley Jackson
	Caroline Thomas
	Catherine Moriarty
	Chandan Gupta
	Ian Chadderton
	Jagadish Gourapoura
	Judith Sharp
	Maheeka Rajamuni
	Michelle Naylor

	Mohannad Mohyeldin
	Niranjala Wickramasinghe
	Rosie Wragg
	Samuel Craven
	Samuel Richards
	Sarah Peacock
	Serena Yen
	Simon Howell
**Leicester General Hospital**	Amy Howard
Principal Investigator Rahil Mandalia	Ankit Darolia
	Chris Molloy
	Francesca O'Brien
	Gamal Ibrahim
	Irina Georgieva
	Matt Gawne
	Puspinder Kaur
	Richard Pertwee
	Taha Namik
	Thomas Chad
	Vipul Kaushik
	Yuvraj Kukreja
**Leicester Royal Infirmary**	Anish Khandia
Principal Investigator Rahil Mandalia	Anthony Wyn-hebden
	Beth Leonard
	Busu Zvidzayi
	Cailin Mcevoy
	Charlotte Hall
	Dan Walker
	Dhruti Pandya
	Diane Jackson
	Emily Parker
	Jack Hague
	Jamie Macdonald
	Jen Taylor
	Kirsty Macfarlane
	Lisa Mclelland
	Mohamed Alebsawy
	Mohamed Shahbudin
	Nathan Ware
	Paras Patel
	Prematie Andreou
	Rahil Mandalia
	Rebecca Cole
	Sharon Tam
	Sobia Jahan
	Syed Hussain

	Wahaballah Abdallah
	Zaid Ahmad
**Leighton Hospital**	Beata Lawecka
Principal Investigator Helen Burton	Claire Gabriel
	Deborah Maren
	Emily Lear
	Helen Burton
	Hewakilpitige Ranaweera
	Juraj Hajnik
	Katherine Pagett
	Laura Ellerton
	Przemyslaw Lawecki
	Rajanbabu Nivethana
	Sanjeewa Ranaweera
	Sheron Clarke
	Victoria Williams
	William Gray
**Lincoln County Hospital**	Arion Pepas
Principal Investigator Manish Kakkar	Crystal Yick
	Frances Wilson-Morkeh
	Kieran Poland
	Lydia Pegman
	Mark Chen
	Rosanna Baker-Wilding
	Sarah Farrington
	Shawn Miranda
	Manish Kakkar
	Nicole Sarens
	Shivan Kanani
**Lister Hospital**	Anna Price
Principal Investigator Pietro Ferranti	Carina Cruz
	Christopher Eyeington
	Darren Smith
	Gloria Sikapite
	James Noble Johnston
	Joy Derigay
	Martin Ebon
	Pietro Ferranti
	Rhos Gabriel
	Sachin Navarange
	Saniyah Shaikh
	Sheena Lim
**Liverpool Womens Hospital**	Adam Mcconville
Principal Investigator Grainne Garvey	Amy Beasley
	Amy Hughes
	Ashoke Shah

	Asim Khan
	Edward Staunton
	Grainne Garvey
	Helen Mcnamara
	John Osakue
	Katrina Reily
	Oliver Henry
**Manchester Royal Infirmary (MRI)**	Adel Hutchinson
Principal Investigator Hannah Greenlee	Alistair Duncan
	Amarjeet Patil
	Anila Sukumaran
	Deborah Paripoorani
	Dominique Jones
	Hannah Greenlee
	Holly Moxon
	Iain Venables
	Lauren Edmunds
	Melanie Barker
	Rahul Norawat
	Rajit Khosla
	Richard Clark
	Shazra Reeza
	Sujesh Bansal
**Medway Maritime Hospital**	Ahmed Rashwan
Principal Investigator Keith Lankester	Aimee Williams
	Andreas Sotirieau
	Gayzel Vallejera
	Jennifer May
	Jodie Wright
	Keith Lankester
	Laura Kemp
	Linda Ofori
	Lisa Parker
	Lucy Connolly
	Mary Everett
	Mohammed Aktar Rezaur Rahman
	Petros Akin-nibosun
	Rebecca Collins
	Sabita Pokharel
	Sheena Cheung
	Sheldon Ferron
	Sian Wilson
	Sureswarasarma Jagatheepan
	Thomas Bolland
	Tom Hatton
	Vandana Rajesh

**Manchester University NHS FT (except MRI)**	Afeefa Rasheeth
Principal Investigator Sujesh Bansal	Alexander Scott
	Sujesh Bansal
	Alistair Sawyerr
	Amr Shalaby
	Andy Fairclough
	Angela Chrisopoulou
	Charlotte Taylor
	Daniel Conway
	Despoina Toraki
	Dhirendra Allen
	Dominque Jones
	Hannah Bennett
	Heather Mcmullen
	Helen Michael
	Helen T-michael
	Jane Shaw
	Jees Porinch
	Jo-ann Midgley
	Joanne Rothwell
	Jossy Kayappurathu
	Kailash Bhatia
	Karan Kanal
	Karen Connolly
	Lisa Cooper
	Lok Heng Arosa Lin
	Lorna Oshea
	Neeraj Bhardwaj
	Nicholas Gould
	Nisha Jayanandan
	Nowfal Rahman
	O'baird Haider
	Olga Colaco
	Oliver Hill
	Peter Alexander
	Preetha Mathew
	Rose Jama
	Sarah Beresford
	Sarah Yousif
	Sharon Baxter-dore
	Sheetal Crasta
	Sofia Fioumi
	Stefania Stewart
	Steph Harrison
	Susan Ferguson
	Thomas Morris

	Tracey Hodgkiss
**Mid Yorkshire Hospital NHS Trust**	Alexandra Metcalfe
Principal Investigator Brendan Sloan	Amit Dalvi
	Amy Longhurst
	Amy Major
	Brendan Sloan
	Chuyan Yu
	Elizabeth Denis
	John Norris
	Lauren Tye
	Lucy Gurr
	Martin Sylvester
	Nathan Sloane
	Nicholas Wroe
	Rusha Saha
	Sarah Buckley
	Sharwend Supermanian
	Susannah Thomas
	Theo Perkins
**Milton Keynes University Hospital**	Andrew Jones
Principal Investigator Richard Stewart	Anne Rose
	Arun Mukkavilli
	Cheryl Padila-harris
	Diane Scaletta
	Eva Howard
	Francesca Teasdale
	Jeannette Smith
	Jimmy John
	Louise Mew
	Louise Moran
	Mirajini Manoharan
	Mustafa Majeed
	Paramdeep Jandu
	Rahim Esmail
	Ramiro D'abrantes
	Rashmi Rebello
	Rhys Smith
	Richard Stewart
	Sara-beth Sutherland
	Shalini Patel
	Sue George
	Veronica Edgell
**Moorfields Eye Hospital**	Floju Chin
Principal Investigator Louisa Pavlakovic	Louisa Pavlakovic
	Mariepi Manolis
	Sejung Park

	Stefanos Ioannidis
**Morriston Hospital**	Abish Kodakkat
Principal Investigator Shilpa Rawat	Alex Wilson-evans
	Caroline Davies
	Christine Range
	Craig Sell
	Debra Evans
	Doaa Awadallah
	Fahad Salim
	Fatima Lahloub
	Helen Williamson
	James Ainsworth
	James Bowen
	Jenny Travers
	Kristian Dye
	Lee Gauntlet
	Marie Williams
	Mark Baker
	Mohammed Hassan
	Mostafa Elsayed
	Richard Hughes
	Sharon Storton
	Shilpa Rawat
	Sophie Jones
	Sophie Tate
	Tim Green
**Musgrove Park Hospital**	Anna Tennant
Principal Investigator Rebecca Purnell	Ashly Thomas
	Charmaine Shovelton
	Gemma Chilcott
	Kate James
	Rebecca Purnell
	Sharon Bates
	Wayne Battishill
**National Hospital for Neurology and Neurosurgery**	Archana Depala
Principal Investigator Eleanor Carter	Eleanor Carter
	Astri Luoma
	Emily Awana
	Emma-Jane Smith
	Megan Griffiths
	Michelle Lam
	Robert John
	Thomas O'Dell
	Zainab Hussein

**Newcastle upon Tyne Hospitals NHS Foundation Trust**	Arankumar Sivasubramaniam
Principal Investigator David Saunders	Arathi Radhakrishnan
	Arti Gulati
	Ben Brown
	David Saunders
	Emma Grace Lewis
	Fatima Simoes
	Grace Lewis
	Hazem Altriagy
	Helen Doherty
	James Savage
	Janaki Pearson
	Jonathan Dinsmore
	Lauren Butler
	Leigh Dunn
	Luzgie Gavina
	Munira Zogaib
	Paula Gomez
	Rikzing Bhutta
	Shin Chia
	Sophie Ingham
	Suzanne Oneill
	Suzy O'Neill
	Tess Wilkinson
	Thomas Wooten
	Tom Wootten
	Wirginia Bada
	Zahid Khan
**Newham Hospital**	Aleksandra Laguna
Principal Investigator Bavesh Gohil	Bhavesh Gohil
	Cherry Jain
	Esme Ingram
	Harkiran Sagoo
	Himani Murdeshwar
	Joseph Gafton
	Kathy Lok-Yu Man
	Labbeka Begum
	Maryam Chaudhry
	Niraj Barot
	Rebecca Carroll
	Sameh Shafek
	Serena Chanoch
	Swati Bansal
	Tim Westwood
	Zohreh Abdi

**NHS Tayside**	Alison Mcculloch
Principal Investigator Sharon Hilton-Christie	Callum Taylor
	Chloe Chang
	Christopher Perman
	David Johnstone
	Duncan Hargreaves
	Eilidh Gillen
	Fiona Burns
	Jennifer Lockhart
	Joanna Tait
	Joaquim Desousa
	Jonathan Miller
	Kirsty Morrison
	Lewis Macleod
	Nicholas Record
	Paul Martin
	Paul Wasik
	Rachel Philips
	Ross Hendry
	Samantha Dean
	Sarah Bugeja
	Sean Sproule
	Sharon Hilton-Christie
	Simon Parkin
	Tim Smith
	Victoria Richmond
**Norfolk and Norwich University Hospital**	Akshita Daga
Principal Investigator Melanie Maxwell	Alex Yusaf
	Carla White
	Caroline Reavley
	Carolyn Dales
	Daniel Teszka
	David Brooks
	Dominic Linden
	Ewa Prusak
	Gemma Maryan
	Harriette Beard
	Hazem Kamel
	James Long
	Jonathan Dearden
	Karan Verma
	Kate Tabrett
	Laura Hobbs
	Meghan Jones
	Melanie Maxwell
	Nancy Wang

	Nicky Ueckermann
	Peter Locke
	Sebastian Locke
**North Middlesex University Hospital**	Bhamini Tharamalingam
Principal Investigator Hemantha Handapangoda	Dinesh Vidanagamage
	Farham Rasheed
	Godknows Mashaire
	Hemantha Handapangoda
	Jane Benedict
	Kugan Xavier
	Rebecca Hull
	Rizana Ghafoor
**University Hospital of North Tees**	Alison Chilvers
Principal Investigator David Pritchard	Carol Adams
	David Pritchard
	Deborah Wilson
	Elaine Siddle
	Elizabeth Parkes
	Emma Connell
	Fe Hernandez
	Gala Stevanovic
	Helen Wardle
	Jasmine Wilkinson
	Liz Baker
	Lorna Shepherd
	Sarah Purvis
**North Tyneside General Hospital**	Angela Dawson
Principal Investigator Adrian Taylor	Chris Yates
	Gemma Mccafferty
	Adrian Taylor
	Hayley Mckie
	Jessica Bell
	Stacey Short
**North West Anglia NHS FT**	Ben Straughan
Principal Investigator Shiny Sivanandan and Sivaprakash Vaitheeswaran	Chiamaka Oladipo
	Chloe Eddings
	Claire Chisenga
	Cristina Constantin
	Eleanor Smith
	Eleonora Gkigkelou
	Elizabeth Clayton
	Helen Bowyer
	Islam Hamed
	Janki Bhayani
	John Frazer

	Karen Scholes
	Kate Fitzpatrick
	Krishma Adatia
	Lauren Eadie
	Loredana Sescu
	Lucy Dunn
	Michelle Bone
	Mukur Ghosh
	Nicola Parker
	Ping Coutts
	Raquel Calcada
	Roberta De
	Sarah Tester
	Shiny Sivanandan
	Sivaprakash Vaitheeswaran
	Susan O'sullivan
	Sushma Ojha
	Susie Osullivan
	Terri-Anne Baker
	Vikas Saxena
	Zarah Brown
**Northampton General Hospital**	Alexander Nottingham
Principal Investigator Prashant Kakodkar	Amaryl Jones
	Andrea Kempa
	Catherine Wilde
	Claire Woolhouse
	Dave Bella
	Ethelwolda Goyena
	Flora Gallamoza
	Jake Pile
	Jane Ocallaghan
	Kate Smith
	Kathryn Hall
	Lorraine Campey
	Lucy Dudgeon
	Lynne Stockham
	Malgorzata Polnik
	Maxine Foo
	Paula Oconnell
	Prashant Kakodkar
	Rachael Hitchcock
	Rachel Tighe
	Shesly Jose
**Nottingham University Hospitals NHS Trust**	Ahmed Elwakil
Principal Investigator David Hewson	Alice Williams
	Amy Mitchell

	Andrew Orsi
	Babar Riaz
	Basma Adiel
	Craig Smith
	David Hewson
	Hannah Dudhill
	Henry Corner
	Jennifer Bowen
	Kelly Williams
	Maryam Umar
	Shameek Datta
	Shannon Boardman
	Shannon Gawley
	Siti Abd Hadi
	Thomas Horne
	Viresh Patel
	Vitul Manhas
	Ying Chean Haw
**Orpington Hospital**	Ahmed Elfaioumy
Principal Investigator Bavesh Gohil	Michael Ayres
	Osokoya Babatunde
**Oxford University Hospitals NHS Trust**	Akshay Shah
Principal Investigator James Day	Alex Filby
	Eyad Abdeljawad
	Georgina Wilson
	Grace Readion
	James Day
	Jean Wilson
	Joy Edwards
	Karen Clark
	Kin Lam
	Luke Holdsworth
	Mukunthakrishnan Lingeswaran
	Neil Davidson
	Nissy George
	Peter Chater-lea
	Sally Beer
	Soyamol Mathew
	Susan Johnston
	Victoria Green
	Vishaka Kerner
**Pilgrim Hospital**	Bryony Saint
Principal Investigator Lisa Sharp	Khaled Ahmed
	Kimberley Netherton
	Kinga Szymiczek
	Lisa Sharp

	Tianzhe Wong
	Trish Tsuro
**Poole General Hospital**	Charlotte Humphrey
Principal Investigator Henrik Reschreiter	Charolotte Barclay
	Claire Osey
	Emma Langridge
	Henrik Reschreiter
	Judith Dube
	Maxine Ashton
	Megan Woolcock
	Patrick Covernton
	Patrick Coverton
	Rebecca Miln
	Yasmin De'Ath
**Portsmouth Hospital NHS FT (Queen Alexandra Hospital)**	Ahmed Abdelhadi
Principal Investigator Renee Ford	Ayesha Shajpal
	Barnaby Jafkins
	Charlotte Bellis
	Connor James
	Daniel Growcott
	Guy Slabbert
	Heidi See
	Hermione Tolliday
	James Collis
	James Connor
	James Gray
	Karen Hudson
	Katherine Pavel
	Mahesh Chandrashekaraiah
	Megan Adams
	Mina Narouz
	Natasha Hughes
	Neha Hasija
	Nicola Mundy
	Nina Szarazova
	Noel Kithakye
	Nosheen Younas
	Rebecca Smart
	Renee Ford
	Shiv Vohra
	Snigdha Seksaria
	Thomas Mankelow
	Timothy Prescott
	Yuen Kang
**Prince Charles Hospital**	Alysha Hancock

Principal Investigator Omar Pemberton	Bibi Khan
	Hatem Elsharawih
	Israel Okwor
	Michael Gibbons
	Najia Hasan
	Nick Gill
	Omar Pemberton
	Ravishankar Punuloou
**Prince Philip Hospital**	Barnaby Hart
Principal Investigator Peter Havalda	Charlotte Jones
	Emma Perkins
	Joanne Connell
	Kathryn Powell
	Konara Dharmarathna
	Laura Micusan
	Linda Brien
	Michael Martin
	Peter Havalda
	Richard Timoney
	Robert Cassidy
	Tracy Lewis
	Yvonna Plesnikova
**Princess Royal University Hospital (PRUH)**	Ahmed Elfaioumy
Principal Investigator Karthick Duraisamy	Ananya Mandal
	Caitlin Spooner
	Clare Finney
	Clare Donegan
	Emma Clarey
	Hayley Kaye
	Humza Yusuf
	Karthick Duraisamy
	Liam Botterill
	Mikaela Theocharidou
	Mohamed Afifi
	Nayer Guirguis
	Nicola Griffiths
	Osokoya Bosokoya
	Pranav Pershad
	Ranganathan Srinivasan
	Ravi Bhatia
	Sophie Rabas
	Sruthi Ravichandran
	Sylvia Martin
**Queen Elizabeth Hospital Gateshead**	Adam Cookson
Principal Investigator Joanne Knight	Emma Allen
	Fiona Sim

	Frederick Hett
	Helen Wild
	Ingvild Helgesen
	James Mcpherson
	Jenny Ritzema
	Joanne Knight
	Julie James
	Nikhil Tambe
	Rachel Lucas
	Stephanie Berry
	Usama Butt
**Queen Elizabeth Hospital, Birmingham**	Ali Usman
Principal Investigator Mansoor Bangash	Anandh Balu
	Anna Musgrave
	Anne Lim
	Chiemezie Orji
	David Desai
	Despoina Terzi
	Hoi Yan Wong
	James Baker
	Jamie Thompson
	Jessica Mernagh
	Jigneshbhai Patel
	Julia Blackburn
	Kavaldeep Jabbal
	Liam Roberts
	Mansoor Bangash
	Menanta Van Velze
	Mohyman El Habishi
	Muneeba Ahmed
	Neelesh Mohan
	Neil Tiwari
	Rupal Swami
	Zikrullah Kalim
**Queen Elizabeth Hospital, Lewisham and Greenwich**	Allison Mascagni
Principal Investigator Danielle Factor	Charlotte Braithwaite-shirley
	Chris Holt
	Daniel Henderson
	Danielle Factor
	Jonase Mutetwa
	Neisha Rhule
	Pradnya Vadnere
	Rachel Williams
	Samia Pilgrim
	Shirley Braithwaite

**Queen Elizabeth Queen Mother Hospital**	Dushyant Sharma
Principal Investigator Sanjay Agrawal	Eva Beranova
	Gabriella Tutt
	Hazel Ramos
	Liam Austin
	Sanjay Agrawal
	Sharon Turney
	Tracy Hazelton
**Queen Elizabeth Hospital, Kings Lynn**	Charlotte Kingsley
Principal Investigator Holy Sira	Harriet Mark
	Holly Sira
	Jessica Murley
	Ping Chen
	Shrestha Sinha
**Queen Victoria Hospital**	Cassandra Honeywell
Principal Investigator Fiona Ramsden	Catherine Bounds
	Christopher Ward
	Fiona Ramsden
	Gail Pottinger
	Julian Giles
	Megan Thomas
	Tom Hansen
	Tracey Shewan
**Queens Hospital Burton**	Agha Isguzar
Principal Investigator Manab Haldar	Amara Masood
	Bis Das
	Caroline Dickens
	Debasis Pradhan
	Emily Blurton
	Gillian Bell
	Louise Wilcox
	Manab Haldar
	Precious Basvi
	Sam Besant
	Sunday Ekaiidem
	Sunita Gurung
**Queens Hospital Romford**	Abishek Chitnis
Principal Investigator Madeep Phull	Daisy Riddle
	Heidi Chandler
	Louis Peakall
	Mandeep Phull
	Oliver Mckinney
	Suraj Shah
**Raigmore Hospital**	Alex Reid
Principal Investigator Mario Fernandes	Alys Wei
	Andrew Richardson

	Ben Marshall
	Frances Hines
	Heather Turnbull
	Kat Murray
	Laura Mcilhatton
	Mario Fernandes
	Mark Hannen
	Rebecca Trimble
**Robert Jones & Agnes Hunt Hospital**	Barbara Linklater-jones
Principal Investigator Melanie Bloor	Charlotte Perkins
	Claire Nicholas
	Claire Wright
	Jayne Edwards
	Johanna Wales
	Julie Steen
	Melanie Bloor
	Sara Owen
	Sarah Clamp
	Teresa Jones
	Tessa Rowlands
	Theresa Garratt
**Rotherham General Hospital**	Andy Mitchell
Principal Investigator Elinor Cromarty	Becky Hawes
	Cheryl Graham
	Elinor Cromarty
	Jake Mccormick
	Lianne Sellors
	Louise Weatherley
	Morwenna Read
	Namitha Jayaprabhu
	Natalie Chan
	Nick Hobbs
	Prasan Kadaramandalgi
	Rachael Faulkner
	Rachel Walker
	Rick Harrold
	Sarah Ingram
**Royal Alexandra Hospital and Vale of Leven**	Alasdair Turnbull
Principal Investigator Michael Brett	Alice Solerod
	Alistair May
	Colin Hutchison
	David Ure
	Fenella Barlow-pay
	Gary Paul
	Iain MacTier
	Louise Clark

	Michael Brett
	Michael Kerr
	Paul Beggs
	Paul Mcconnell
	Rebecca Vere
	Shashi Timalapur
	Timothy Gray
**Royal Berkshire NHS Foundation Trust**	Amelia Robinson
Principal Investigator Richard Barnes	Camilla Jackson
	Cecille Cadampog
	Dan Kent
	Dave Golding
	Killian Donovan
	Kinza Emmanuel
	Kulpdeep Nijjar
	Odhran Keating
	Parminder Bhuie
	Poppy Sellwood
	Richard Barnes
	Sabi Rai
**Royal Blackburn Hospital**	Andrew Lancaster
Principal Investigator Anuradha Kurvey	Anuradha Kurvey
	Beverley Hammond
	Georgina Sutcliffe
	Jillian Fitchett
	Sanjiv Sharma
**Royal Bolton Hopsital**	Aashish Koirala
Principal Investigator Peter Sandbach	Helen Dixon
	Antonia Peilober-Richardson
	Chris Dale
	Elfateh Ibrahim
	Patricia Hodgson
	Peter Sandbach
	Priyash Verma
	Rachel Fletcher
	Raphael Holmes
**Royal Bournemouth Hospital**	Amy Gribble
Principal Investigator James Walker	Debbie Branney
	Faith Beecham
	Heather Tiller
	James Walker
	Lindsay Rogers
	Nina Barratt
	Sally Pitts
	Sarah Savage
**Royal Cornwall Hospital**	Benita Adams

Principal Investigator Claire Preedy	Cara Campbell
	Charlotte Barker-kirby
	Claire Preedy
	Daniel Phillips
	Eden Leaper
	Elliot Edmund
	Eve Fletcher
	Evelina Russell
	Jack Williams
	Jemima Henstridge-blows
	Octavia Smith
	Ollie Ryan
	Peter Thomas
	Riyea Akhtar
	Selina Roy
	Shane Roy
	Suzanne Dean
	Virginija Vilkelyte
**Royal Derby Hospital**	Alison Fletcher
Principal Investigator Nagendra Prasad	Carly Mcdonald
	Catherine Addleton
	Charlotte Downes
	Coral Smith
	Corinne Paxton
	David Daly
	Emily Mignott
	Fiona Scothern
	Jeanette Allison
	Julie Edmonds
	Katie Large
	Lisa Mayles
	Liz Nadin
	Maggie Langley
	Melanie Hayman
	Mercy Korley
	Nagendra Prasad
	Samia Hussain
	Sarah Miller
	Timothy Streets
	Zita Ibatuliniene
**Royal Devon and Exeter Hospital**	Aaron Lavin
Principal Investigator Helen Gilfillan	Chris Gillett
	Ellie Nelson
	Francis Bonomaully
	Gemma Clark
	Helen Gilfillan

	James Grant
	Jo Wilson
	Katie Flower
	Kevin Windsor
	Linda Park
	Lisa Jones
	Martha Belete
	Megan Purchall
	Peggy Fooks
	Pei Jean Ong
	Rebecca Dyar
	Sophie Ashman
	Tania Nightingale
	Tom Hewitt
	Vanessa Chiappa
	Will Foers
	Will Spencer
	Zach Jeffery
	Zahid Gilitwala
**Royal Gwent, the Grange and Nevill Hall Hospitals**	Alison Hare
Principal Investigator Jake Hartford-beynon	Anna Roberts
	Anushka Sierarine
	Charlotte Dunn
	David Agombar
	Hannah Hoskins
	Jake Hartford-beynon
	Kota Kumar
	Maxine Nash
	Sammy Sharif
	Simran Kooner
	Swyn Lewis
	Zoe Bennetton
**Royal Hampshire County Hospital**	Emma Norman
Principal Investigator Kathleen Hempenstall	Jordan Dennis
	Kathleen Hempenstall
	Rachael Brooks
	Sarah Davidson
**Royal Lancaster Infirmary**	Andrew Prior
Principal Investigator Corinne Rimmer	Aziza Aini
	Corinne Rimmer
	Craig Marshall
	Helen Spickett
	Hilary Thatcher
	Jack Dalziel
	Jayne Craig

	Julie Le
	Jyothis Manalayil
	Karen Burns
	Nurul El-Ruslan
	Steve Peters
	Sushma Paccha
	Therese Kelly
	Wael Abdelrhman
**Royal Liverpool and Broadgreen University Hospitals NHS**	Amanda Wood
Principal Investigator Richad Ramsaran	Andre Simons
	Christy Ord
	Claire Hennigan
	Emilia Spodniewska
	Fran Westwel
	Francesco Ferraro
	Georgia Williams
	Hannah Davis
	Hefin Llewellyn
	Hema Thomas
	Ibrahim Abdelkhalek
	Jenny Kirkpatrick
	Katelyn Aitchison
	Kera Hainey
	Laura Cureton
	Lauren Greer
	Peter Harding
	Rasmeet Kainth
	Richard Ramsaran
	Sophie Holder
**Royal National Orthopaedic Hospital**	Amit Patel
Principal Investigator Rachel Baumber	Antony Finny
	Charlotte Pratt
	Esther Hanison
	Finny Antony
	Fiona Fitzgerald
	Jackline Nkhoma
	Nana Okine
	Nnebuife Oji
	Rachel Baumber
	Trusha Halai
**Royal Surrey County Hospital**	Donna Sanga
Principal Investigator James Mckinlay	James Mckinlay
	Jerik Verula
	Natalia Michalak
	Nicholas Maskell

	Paula Carvelli
**Royal United Hospital, Bath**	Abigail Mann
Principal Investigator Lesley Jordan	Annete Moreton
	Catherine Bressington
	Charlotte Ekblad
	Frances Parry
	Gabrielle Evans
	Jennifer Pullen
	John Wright
	Joyce Katebe
	Katherine Lloyd-jones
	Laura Evans
	Lesley Jordan
	Lidia Ramos
	Lucy Howie
	Melody Rich
	Natalie Gaskell
	Rachel Awan
	Rachel Beer
	Ronan Hanratty
	Sarah Burnard
	Sarah Hierons
	Sophia Muschnik
	Tobin Osicki
	Tom Cloke
	Tonia Clark
	Wendy Duberry
**Royal Victoria Hospital, Belfast**	Adam Glass
Principal Investigator Kerry Featherstone	Adam Lowe
	Amy O'donnell
	Anastasia Mcbride
	Blayne Mccann
	Catherine Poots
	Claire Mccaul
	Emma Gardiner
	Emma Sweeney
	Hannah Cooper
	Helen Mcgourty
	Kerry Featherstone
	Matthew Mcguckin
	Ruth Mccrystal
**Royal Wolverhampton Trust**	Alex Villaplaza
Principal Investigator Asha Ramkumar	Amardeep Kulkarni
	Antonella Meraglia
	Asha Ramkumar
	Beenish Bashir

	Benedict Williams
	Chakravarthy Tutika
	Deepak Ravindran
	Emma Jay
	Iqra Jangda
	James Haddock
	Jyothi Avula
	Kanika Daga
	Katie Betts
	Kesavan Dhamodaran
	Lewis Davies
	Mohamed Shariff
	Nick Ledlie
	Parvathy Nair
	Philip Thomas
	Prabjoyt Kler
	Puja Sharma
	Roma Kalaria
	Ross Evans
	Stephen Norris
	Sumant Shanbhag
**Russells Hall Hospital**	Anser Ali
Principal Investigator Anser Ali	Lesley Jones
	Sarah Stavert
	Stacey Forsey
	Thomas Wallbridge
	Tony Anthony
	Vishal Amin
**Salford Royal Hospital**	Alice Harvey
Principal Investigator Manjunatha Patel	Bethan Charles
	Danielle Walker
	Diane Lomas
	Elena Aperti
	Helen Cristensen
	Jesse Oliver
	Kris Sivarajan
	Manjunatha Patel
	Melanie Taylor
	Sheryl Bell-rhone
	Vicky Thomas
**Salisbury District Hospital**	Abby Rand
Principal Investigator Xantha Holmwood	Hayley Savage
	Oliver King
	Xantha Holmwood
**Scarborough and Bridlington Hospital**	Alexander Knighton
Principal Investigator Bejamin Chandler	Alexander Polding

	Alison Turnbull
	Anna Waine
	Benjamin Chandler
	James Sangma
	Janine Mallinson
	Jordan Toohie
	Kate Quigley
	Katie Howard
	Kerry Elliott
	Laith Alsaket
	Laura Barman
	Rachael Harrison
	Tania Neale
**Sherwood Forest Hospitals (Kings Mill Hospital)**	Andra Baghiu
Principal Investigator Srinivas Magham	Camelia Goodwin
	Cheryl Heeley
	Donna Sowter
	Helen Shirt
	Jill Kirk
	Kaytie Bennett
	Leah Holloway
	Lynne Allsop
	Mandy Gill
	Nigel Thorpe
	Philip Buckley
	Rachel Johnson
	Sarah Turner
	Srinivas Magham
	Stephanie Pike
	Susan Smith
	Vaisakh Viswanathan
	Wayne Lovegrove
**Shrewsbury and Telford Hospital NHS Trust**	David Stuckey
Principal Investigator Paul Jones	Louise Ting
	Paul Jones
	Richard Watson
**Southampton General Hospital**	Aldo Bibnamini
Principal Investigator Karen Salmon	Alice Baker
	Alix Bird
	Anna Foster
	Aurore Gerrish
	Belinda Roberts
	Clare Bolger
	Daniela Georgieva
	David Baker

	Diana Mondo
	Edisa Xhani
	Elisabeth Jarman
	Erika Kovacs
	Hannah Wardall
	Helena Eagles
	Imogene Hedges
	Jonathan Biss
	Karen Salmon
	Kerry Thorpe
	Kim Golder
	Luke Bracegirdle
	Maria Baggott
	Matthew Morris
	Michael Carter
	Norma Diaper
	Owen Gregory
	Rachel Burnish
	Rachel Schranz
	Sandra Bartolomeu-pires
	Stephanie Kirby
	Susan Jackson
	Thomas Purvis
**Southend University Hospital**	Aneta Oborska
Principal Investigator Aneta Oborska	Bridgett Masunda
	Henna Sattar
	James Jegard
	Mohamed Eshmandi
	Naima Khalk
	Nigara Atayeva
	Prisca Gondo
	Sunil Shah
	Swapna Kunhunny
**Southern Health & Social Care Trust**	Aidan Cullen
Principal Investigator Michael Jones and Laura McLoughlin	Alexandrina Todd
	Alison Blair
	Anastasia Solomou
	Andrew Dunbar
	Barry Mcconville
	Catherine Yarr
	Claire Steenson
	Declan Love
	Denise Mcfarland
	Duncan King
	Emma Doherty

	Erik Lichnovsky
	Garwei Ho
	Grainne Mckendry
	Jacek Sobocinski
	Jack Carmichael
	James Crockett
	Joanne Wylie
	Laura Mcloughlin
	Laura Somerville
	Manvi Singhal
	Michael Jones
	Michael Magee
	Mohammed Zayan
	Niranjana Mohan
	Patricia McCaffrey
	Peter Mcclung
	Peter Merjavy
	Rebecca Rooney
	Ruby Crothers
	Ruth Thornbury
	Ruth Thonbury
	Samantha Leung
	Shane Donnelly
	Tim Bennett
**Southmead Hospital, Bristol**	Benjamin Hillam
Principal Investigator Sarah Martindale	Chris Thorne
	Hannah Matthews
	James Marshall
	James Matthams
	Jerome Condry
	Kate Bell
	Kerry Smith
	Lydia Osborne
	Ottilie Lloyd-thomas
	Peter Sykes
	Richard Mason
	Sam Scholes
	Sarah Dolling
	Sarah Martindale
**Southport and Ormskirk NHS Trust**	Abdul Alim Khan
Principal Investigator Abdul Alim Khan	Afeez Abderahman
	Alveena Bilal
	Amanda Adigwe
	Anna Morris
	Belal Yasin
	Bethany Preece

	Christopher Goddard
	Claire Corless
	Himashi Nawimana
	Patricia Jenkins
	Rebecca Seddon
	Rohith Nayak
**St Johns Hospital**	Andrew Goddard
Principal Investigator Andrew Goddard	Bridget Podmore
	Michael Kriger
	Paul Purvis
	Rebecca Lovett
**St Marys Hospital, Imperial**	Ajantheny Naguleswaran
Principal Investigator Kenneth Murray	Dharshini Rajasooriyer
	Drew Harding
	Emily Russell
	Jonathan Dunne
	Lloyd Nunag
	Sulaimaan Haq
	Tizzy Abraham
**St Richards Hospital**	Denise Szabo
Principal Investigator Emily Dana	Emily Dana
	Emma Finlay
	Erikka Siddall
	Esther Towner
	Grace Williams
	Hannah Atkinson
	Sharon Floyd
	Sophie Clarke
	Thomas Thompson
	Yolanda Baird
	Yvette Thirlwall
**Stepping Hill Hospital**	Ali Nawaz
Principal Investigator Petya Chalakova	Bishal Gautam
	Hywel Garrard
	Karen Cheung
	Leigh Wilson
	Malathi Jabanathan
	Nicolas Short
	Petya Chalakova
	Rebecca Mills
	Rohan Colaco
	Thomas Moore
	Ujala Khwaja
**Sunderland Royal Hospital**	Akhil Lakhani
Principal Investigator Sean Cope	Alexandria Cropp
	Claire Agius

	David Swetman
	Edward Halvey
	Emma Worth
	Faisal Shiekh
	Hannah Fairclough
	Henry Tancred-holmes
	Iain Dryburgh
	Jack Shepard
	James Durrand
	Jennifer Hooper
	Katie Burke
	Lindsey Woods
	Matthew Bickerton
	Natalie Hickling
	Nicholas Killips
	Robert Hessell
	Sabrina Kapur
	Sarah Dawson
	Sean Cope
	Sophie Curtis
	Tom Ballance
	Tom Collis
**The Christie Hospital**	Adam Bulinski
Principal Investigator Jaya Nariani	Anhthony Murphy
	Jaya Nariani
	Parisa Cutting
	Roman Mary-genetu
	Suzanne Allibone
**The James Cook University Hospital**	Abigail List
Principal Investigator Charlotte Anderson	Charlotte Anderson
	Dean Wilkinson
	Harry Heaton
	Kerry Colling
	Leanne Wakes
	Nicola Powley
	Tracy Ruddick
**The Princess Alexandra Hospital**	Bibi Badal
Principal Investigator Rajamani Seturaman	Dinesh Das
	Huw Griffiths
	Joanne Finn
	Karen Ixer
	Michelle Grove
	Nikki White
	Patricia Nabayego
	Rajamani Seturaman
	Sophie Harris

**The Royal Glamorgan Hospital**	Alice O'donnell
Principal Investigator Neeta Taylor	Ameerah Azmil
	Amy Nixon
	Bethan Gibson
	Catherine Archer
	Catherine Farrelly
	Ceri Lynch
	Faye Bond
	Iain Mclure
	Kathryn Lloyd-thomas
	Martin Cole
	Nathan Anderson
	Neeta Taylor
	Nick Preston
	Richard Roberts
	Robert Jones
	Tracey Thomas-wood
**The Royal London Hospital**	Aaroh Dubey
Principal Investigator Ashley Parker	Alison Li
	Anna Yang
	Asher Knight
	Ashley Parker
	Ben Thorp
	Divya Harshan
	Fatimah Bme
	Hannah Nugent
	Heather Fuller
	Ioannis Kapsokalyuas
	Jennifer Overend
	Karen Collins
	May Webb
	Mina Arsan
	Mira Razzaque
	Nicky Lau
	Philip Devendra
	Xiaoyan Yang
**The Whittington Hospital**	Amy Findlay
Principal Investigator Denise Lim	Denise Lim
	Emma Jenkins
	Floju Chin
	Kayleigh Gilbert
	Lauren Booker
	Lucy Blair
	Nicola Brown
**Torbay Hospital**	Angie Foulds
Principal Investigator Johannes Retief	Gregory Warren

	Helen Williams
	Johannes Retief
	Kieran Miller
	Laura Helley
	Pauline Aspa
**Tunbridge Wells Hospital**	Daniel Gorman
Principal Investigator Hilary Taylor	Emily Phiri
	Fabio Fernandes
	Gavin Fossey
	Heather Callaghan
	Hilary Taylor
	Indrowtree Sookun
	Jen Assimakopoulos
	Miriam Davey
	Rebecca Seaman
	Vicky Earl
**University College Hospital**	Atokwame Ocansey
Principal Investigator Sohail Bampoe	Chimverly Diaz
	Denise Wyndham
	Ernesto Bettini
	Gladys Martir
	Maya Sussman
	Sohail Bampoe
**University Hospital Ayr**	Amadeusz Ziarkowski
Principal Investigator Philip Hamilton	Cameron Taylor
	Danielle Gilmour
	Debbie Callaghan
	Fiona Elliott
	Mark Wilson
	Philip Hamilton
	Pritam Mohanty
	Sharon Meehan
	Stephen Wood
	Toni Mcintosh
**University Hospital Coventry**	Adam Boulton
Principal Investigator Carol Bradbury	Anne Scase
	Carl Groves
	Carol Bradbury
	Charlotte Bullock
	Giles Coverdale
	Jennifer Morrish
	Katharine Reeves
	Katie Ramm
	Kay Mak
	Michelle Aukland
	Nalini Sethia

	Sarah Delahunt
	Stephen Pearson
	Susanne Anver
	Tom Radcliffe-law
	Tom Trouton
	Tom Wilson
**University Hospital Crosshouse**	Ali Meikle
Principal Investigator Andrew Clark	Amy Clark
	Andrew Clark
	Cara Hughes
	Colin Pow
	Conor Nichol
	David Finn
	Hugh Neill
	Jacqueline Mccarthy
	Jamie Weir
	Jane Collie
	Kathryn Mcaleer
	Ker Wei
	Laura Macdonnell
	Mark Andonovic
	Monika Doshi
	Nick Brown
	Phil Jacobs
	Rob Bonar
	Ross Junkin
	Ruth Blackett
	Sam Norman
	Sarjit Singh
**University Hospital Hairmyres**	Alfie Lloyd
Principal Investigator Jonathan Edgar	Arthur Norton
	Chloe Macdonald
	Claire Young
	Dairshini Sithambaram
	Emma Lee
	Euan Murdoch
	Faith Dalgaty
	Fiona Walker
	Jonathan Edgar
	Laura Meney
	Leigh Hamilton
	Louise Jamieson
	Lynn Valentine
	Michael Airlie
	Ross Dryden
**University Hospital Lewisham**	Anghard Langdon

Principal Investigator	Cara Mclean
	Elizabeth Herbert
	Fay Riley
	Hamish Jackson
	Harpreet Gill
	Josephine Priest
	Kimberley Rhodes
	Manju Agarwal
	Rebecca Mitchell
	Richard Crowson
**University Hospital Monklands**	Gail Fleming
Principal Investigator Samuel Maguire	Michael Rodger
	Samuel Maguire
	Tracy Baird
**University Hospital of North Durham**	Abigail Sachs
Principal Investigator David Hamilton	Ami Wilkinson
	Andrea Kay
	Arun Varghese
	Chloe Gilchrist
	Christopher Pennington
	David Hamilton
	Jacob Mcdermott
	Jamie Greenwood
	Jonathan Dennis
	Joseph Haynes
	Khalid Zahir
	Lauren Ferguson
	Mark Birt
	Mary Leese
	Melanie Kent
	Noreen Kingston
	Peter Standen
	Stefanie Hobson
	Vicki Atkinson
	Victoria Allinson
**University Hospital Wales**	Amanda Skingle
Principal Investigator Margaret Coakley	Anna Gilfedder
	Anna Pisarcyk
	Ben Sharif
	Brigitte Baxter
	Charlotte Eglinton
	Claire Bunce
	David Manson
	Deborah Mann
	Eais Mehmood
	Farzad Saadat

	Gail Williams
	George Pitchers
	Gina Allen
	Graham Picton
	Hannah Saitch
	Harris Beca
	Hawys Evans
	Hywel Evans
	Ifan Patchell
	Jana Anandarajah
	Jennie Williams
	Jo Krawczyk
	Joseph Edwards
	Josh Patch
	Julia Parnell
	Jyothi Srinivas
	Kerry Paradowski
	Laura Gray
	Luca Galvani
	Margaret Coakley
	Martin Grigg
	Matt Short
	Matthew Forester
	Natalya Acres
	Nia Humphry
	Nicola Ball
	Nicola Jardine
	Oluwafisayo Olabisi
	Pete Rogers
	Phil Molloy
	Rachel Skinner
	Russel Townsend
	Sam Tyrell
	Samuel Bird
	Sara Turley
	Shaun Oram
	Sonal Lodhi
	Sophie Foreman
	Sophie Pooley
	Tom Roberts
	Zarah Paris
**University Hospitals Sussex NHS FT (Royal Sussex County Hospital, Princess Royal Hospital, Haywards Heath, Sussex Orthopaedic Treatment Centre)**	Afonso Sequeira
Principal Investigator Stuart White	Alison Porges

	Caroline Humphreys
	Denise Skinner
	Gabriella Salvi
	Jane Gaylard
	Justyna Nowak
	Kanmani Lakshmikantha
	Kate May
	Keely Stewart
	Mel Smith
	Melissa Thorburn
	Miles Seavill
	Patrick Haye
	Patrick Hayes
	Stuart White
	Usman Jamil
	Valentina Toska
	Zdenka Cipinova
**Victoria Hospital, Kirkcaldy**	Ally Rocke
Principal Investigator Katie Hunter	Cara Mccahill
	Emma Scahill
	Jessica Mccleery
	Katie Hunter
	Mandy Couser
	Olivia Groom
	Patricia Cochrane
	Sarah Galloway
	Susan Fowler
**Warwick Hospital**	Ben Wilkinson
Principal Investigator Emert White	Charlie Hudson
	Chloe Thomson
	Claire Baldwin
	Emert White
	Hannah Wolfenden
	Hooi Shin
	Ifra Zahoor
	Jonathan Finnity
	Parag Shastri
	Rakesh Khunti
**Watford General Hospital**	Barnaby Glover
Principal Investigator Nidhi Gautam	Camilla Zorloni
	Chiara Ellis
	David Mccretton
	Dharshana Ranasinghe
	Elvira Hoxha
	Georgia Perkins
	Harish Ningegowda

	Jackie Evans
	Jules Kho
	Mary James
	Mehul Raithatha
	Melanie Claridge
	Naila Zahoor
	Nidhi Gautam
	Priyanka Moon
	Rajesh Shankar
	Rebekah Mostyn
	Rosie Cortaville
	Sarah Loftus
	Saul Sundayi
	Sukanya Khan
	Xiaobei Zhao
**West Cumberland Hospital**	Elena Grani
Principal Investigator Elena Grani	Hannah Craig
	Melanie Scott-richardson
	Rosemary Harper
	Una Poultney
	Wendy Armstrong
**West Middlesex University Hospital**	Amir Majid
Principal Investigator Mhairi Jhugursing	Amrinder Sayan
	Daniel Stubbins
	Farrah Akkouch
	Filomena Liccardo
	Harriet Wilson
	Jonathan Fox
	Louise Cloney
	Marie-louise Svensson
	Mhairi Jhugursing
	Mary O'brien
	Priyakam Chowdhury
	Roya Movahedi
	Wazir Salamut
**Western General Hospital**	Alistair Coleman
Principal Investigator Louise Peach	Elspeth Paterson
	Emily Kidd
	Faisal Jafar
	Fiona Auld
	Iain Slessor
	Julie Baruah-young
	Louise Peach
	Mala Greamspet
	Nina Park
	Rebecca Gormley

	Teodora Filipescu
	Akila Visvanathan
	Alison Smith
	Chetan Pataki
	Debra Chatterton
	Edel Robbins
	Harvey Dymond
	Katrina Stallard
	Lindy Murray
	Robert Grecian
	Susan Wilkinson
**William Harvey Hospital**	Angela Moon
Principal Investigator Kim Jemmett	Angela Munteanu
	Emma Ingall
	Heather Weston
	James Rand
	Jeffrey Tsang
	John Cockcroft
	Kim Jemmett
	Max Rigaudy
	Prathiban Kumar
	Reanne Solly
	Sam Mcferran
	Sarah Stirrup
	Victoria Clarke
**Withybush General Hospital**	Amir Mekael
Principal Investigator Sunita Agarwal	Deshan Wickramaarachchi
	Edward Todd
	Jolene Brooks
	Lara Sabry
	Mary O'regan
	Michelle Edwards
	Prasanth Ganesan
	Sharmila Ajaratnam
	Sunita Agarwal
**Worcestershire Acute Hospitals NHS Trust (Worcester Royal Hospital, The Alexandra Hospital and Kidderminster Treatment Centre)**	Aly-khan Makhani
Principal Investigator Emily Johnson	Antonia Stone
	Cindy Persad
	Emily Johnson
	Hannah Puddy
	Jack Lee
	Joseph Swani
	Mike Eager
	Mukunder Patel

	Omolola Afelumo
	Prakash Vadukul
	Sarah Clayton
	Shahid Khan
	Shailendra Krishniah
	Suhas Hebri
	Vatsala Padmanabhan
	Vinayak Nirmalan
	Zuzana Hutkova
**Worthing Hospital**	Amy Davis
Principal Investigator Patrick Thorburn	Ben Mcallister
	Bjorn Birk
	Carmen Laue
	Carrie Ridley
	Charlotte Quick
	Chloe Hoskins
	Chris Redburn
	Dan Puntis
	Hannah Shimmin
	Heather Fox
	Jamie Gibson
	Jimmy Siu
	Joanna Dearden
	Jordi Margalef
	Kat Ganly
	Katrine Thorup
	Kirsten King
	Layla Brookfield
	Linda Folkes
	Lucy Brennan
	Marek Perera
	Mark Burgess
	Masseh Yakubi
	Mohamam Selim
	Neil Botting
	Nicholas Weston-smith
	Nicole Martins
	Oliver Harvey
	Patrick Thorburn
	Piyush Varma
	Raquel Gomez-marcos
	Sam Goodhand
	Samira Green
	Sarah Driver
	Sarah House
	Shane Weinmann

	Todd Leckie
	Vivienne Cannons
	Will Southall
**Wrexham Maelor Hospital**	Ahmed Salem
Principal Investigator Anna Williams	Amelia Devine
	Anna Williams
	Andrew Lonsdale
	Chaw Nandar
	Chijioke Orji
	Chris Littler
	Daphne Gunness
	Emma Mcivor
	Gillian Bennett
	Hope Worthington
	Jane Stockport
	Josh Exley
	Luke Williams
	Mohit Sethi
	Myles Roach
	Oliver Smith
	Philip Metcalf
	Rachel Bradley
	Robert Jesty
	Romy Peterson
	Sara Murray
	Thomas Abberton
	Will Simpson
**Wrightington Hospital and Royal Albert Edward Infirmary**	Anne Evans
Principal Investigator Anthony Short	Anthony Short
	Caroline Tierney
	Claire Williams
	Daivd Wilcock
	Emma Robinson
	George Metias
	Joshua Cooper
	Katherine Dowdall
	Lauren Brown
	Rebecca Smith
	Sarah Liderth
	Thomas Bedwell
	Tracey Taylor
	Valerie Parkinson
**Yeovil District Hospital**	Aashkaben Shah
Principal Investigator Agnieszka Kubiszpudelko	Agnieszka Kubiszpudelko
	Alice Quayle

	Alison Lewis
	Ashraf Ahmed
	Ayman Gouda
	Jess Perry
	Kate Beesley
	Linda Howard
	Matthew Hillier
	Munnaza Irfan
	Nigel Beer
	Rachel Wood
	Ramez Aziz
	Sarah Board
	Thomas Bennett
	Tressy Pitt-kerby
**York Hospital**	Andrew Chamberlain
Principal Investigator Andrew Chamberlain	Claire Brookes
	Daniel Greenwood
	Ellis Bramall
	Harriet Pearson
	Heidi Redfearn
	Julie Anderson
	Kate Howard
	Louise Martin
	Nicola Marshall
	Paul Brittain
	Radu Chirvasuta
	Sally Gilroy
	Stephy Jose
	Zoe Cinquina
	Zoe Scott
**Ysbyty Glan Clwyd Hospital**	Alice O'docherty
Principal Investigator Venkat Sundaram	Amy Gullis
	Andras Safranko
	Angela Pye
	Annette Bolger
	Hossam Abdelmotaal
	Hossam Helmy
	Laurence Baker
	Llinos Davies
	Nathan Littley
	Rachael Farnell
	Rachel Manley
	Venkat Sundaram
	Victoria Garvey
	Yehya Slim
**Ysbyty Gwynedd Hospital**	Bryn Ellis

Principal Investigator Chrisopher Bailey	Christopher Bailey
	Donna Ward
	Ellen Knights
	Jasmine Peh
	Jeannie Bishop
	Lisa Roberts
	Wendy Scrase
**Unknown**	Emma Perkins
	Ahmed Abdelhadi
	Lynn Abel
	Suzanne Allibone
	Emad Al-Washash
	Alpha Anthony
	Beenish Bashir
	Hannah Baytree
	Karen Beaumont
	Austin Begbey
	Sophie Berry
	Emily Bevan
	Parminder Bhomra
	Jonathan Blake
	Neil Botting
	Charlotte Brathwaite-Shirley
	Layla Brookfield
	Julie Chadwick
	Kwun Chan
	Karen Cloherty
	Jon Clouston
	John Cockcroft
	Shirley Cocks
	Joanne Connell
	Maria Corretge
	Alexa Cox
	Benjamin Cracknell
	Thomas Craven
	Kavit Dasari
	Elizabeth Denman
	Anna Devlin
	Kate Dillon
	Joe Edwards
	Abdalla Eisazwi
	Kamal El-Badawi
	Zenira Elbasheer
	Sofia Fiouni
	Rebecca Flanagan
	Marian Flynn-Batham

	Linda Folkes
	Aidan Fullbrook
	Gillian Garden
	Ben Goodman
	Jonathan Goodship
	Miguel Guerrero
	Ramanan Gukathasan
	Emma Gunter
	Dashiell Hall
	Rachel Hallam
	Henry Harcourt
	Matt Henwood
	Natasha Hughes
	Mohamed Imam
	Dianne Jackson
	Zachary Jeffery
	Ancy John
	Lijo Joy
	Prasanth Kandepalli
	Matthew Kearney
	Heather Kelley
	Stephanie Kirby
	Edward Knights
	Louis Koizia
	Chethana Kossinnage
	Kartik Kota
	Scott Latham
	Anna Leslie
	Chiwen Lin
	Kat Lloyd Jones
	Annabel Lloyd-Thomas
	Ruairidh Mackay
	Rose Mackonochie
	Asif Mahmood
	Ben McAllister
	Chris McKee
	Emma McKenna
	Alex Metcalfe
	Angelo Milioto
	Raksha Mistry
	Selma Mohammed
	Mirela Mukaj
	Anthony Murphy
	Sophia Muschik
	Priya Nagaraj
	Vinayak Nirmalan

	Raymond Njafuh
	Ahmed Osman
	Sarah Packer
	Diran Padiachy
	Emily Pallister
	YeeWee Pang
	Amy Parekh
	Na Hyun Park
	Sophie Patterson
	George Pitchers
	Barbara Pryzsyz
	Paul Purves
	Umang Qazi
	Samyuktha Raj
	Tom Ratcliffe-Law
	Tom Reevell
	Morag Renton
	Kat Rhead
	Maxime Rigaudy
	Natalie Rodden
	Hannah Saitch
	Mark Sandford
	Selena Sehgal
	Imran Shareiff
	Jasmin Shearer
	Rachel Shipsides
	Michaela Sibsey
	Rhona Sinclair
	Sophia Strong-Sheldrake
	Jeevan Subramaniam
	Neena Suchdev
	Seb Tanner
	Emma Tanton
	Chris Tattersall
	Jacqui Taylor
	Anna Te Water Naude
	Philip Thomas
	Alex Touze
	Tor Tuckey
	Sara Turley
	Chidimma Ugonabo
	Yuvashree Venkatesan
	Lagath Wanigabadu
	Christopher Ward
	Annamaria Wilce
	Caroline Wrey Brown

	Roger Yau
	Nur Zalkapli
