# Supplementary material for: Postoperative outcomes in older patients with postoperative delirium in the UK: SNAP-3, a snapshot observational study
Source: Br J Anaesth. 2026 Feb 26;136(5):1578–87. doi: 10.1016/j.bja.2026.01.030 (PMC13197927; doi:10.1016/j.bja.2026.01.030)
Supplement: Supplementary file 2 [file mmc2.docx]

# Supplementary material for:

# Postoperative outcomes in older patients with postoperative delirium in the UK: SNAP-3, a snapshot observational study

## Authors

- Helen A Blake ^1^, [helen.blake@ucl.ac.uk](mailto:helen.blake@ucl.ac.uk) *corresponding author
- Claire J Swarbrick ^2,3,4^
- SNAP-3 collaborators (supplementary table 17)
- Karen Williams^3^
- Bob Evans^3^
- Thomas Poulton^1, 5, 6^
- Samuel Nava^7^
- Akshay Shah^8, 9^
- Peter Martin^1^
- Judith SL Partridge^10, 11^
- Iain K Moppett^2, 3, 12^, [iain.moppett@nottingham.ac.uk](mailto:iain.moppett@nottingham.ac.uk) @iainmoppett (BlueSky)

1. University College London, London, UK
2. University of Nottingham, Nottingham, UK
3. Royal College of Anaesthetists, London, UK
4. Royal Devon University Healthcare NHS Foundation Trust, Exeter, UK
5. Peter MacCallum Cancer Centre, Melbourne, Australia
6. University of Melbourne, Australia
7. Royal United Hospitals Bath NHS Foundation Trust, Bath, UK
8. University of Oxford, Oxford, UK
9. Imperial College Healthcare NHS Trust, London, UK
10. Guy's and St Thomas' NHS Foundation Trust, London, UK
11. King’s College Hospital, London, UK
12. Nottingham University Hospitals NHS Trust, Nottingham, UK

Table of Contents

[Supplementary tables 4](#_Toc211620416)

[Supplementary table 1: STROBE checklist 4](#_Toc211620417)

[Supplementary table 2: Prevalence of missing data in SNAP-3 7](#_Toc211620418)

[Demographics: whole cohort 8](#_Toc211620419)

[Supplementary table 3: Demographics of SNAP-3 cohort, with and without delirium 8](#_Toc211620420)

[Clinical characteristics: whole cohort 13](#_Toc211620421)

[Supplementary table 4: Clinical characteristics of SNAP-3 cohort, with and without delirium 13](#_Toc211620422)

[Demographics: subgroups 16](#_Toc211620423)

[Supplementary table 5A: Demographics of participants aged ≥85 in the SNAP-3 cohort, with and without delirium 16](#_Toc211620424)

[Supplementary table 5B: Demographics of participants aged <85 in the SNAP-3 cohort, with and without delirium 22](#_Toc211620425)

[Supplementary table 5C: Demographics of participants living with frailty in the SNAP-3 cohort, with and without delirium 28](#_Toc211620426)

[Supplementary table 5D: Demographics of participants not frail in the SNAP-3 cohort, with and without delirium 34](#_Toc211620427)

[Supplementary table 5E: Demographics of participants having elective surgery in the SNAP-3 cohort, with and without delirium 40](#_Toc211620428)

[Supplementary table 5F: Demographics of participants having non-elective surgery in the SNAP-3 cohort, with and without delirium 46](#_Toc211620429)

[Supplementary table 5G: Demographics of participants having inpatient surgery in the SNAP-3 cohort, with and without delirium 52](#_Toc211620430)

[Clinical characteristics: subgroups 57](#_Toc211620431)

[Supplementary table 6A: Clinical characteristics of participants aged ≥85 in the SNAP-3 cohort, with and without delirium 58](#_Toc211620432)

[Supplementary table 6B: Clinical characteristics of participants aged <85 in the SNAP-3 cohort, with and without delirium 61](#_Toc211620433)

[Supplementary table 6C: Clinical characteristics of participants living with frailty in the SNAP-3 cohort, with and without delirium 64](#_Toc211620434)

[Supplementary table 6D: Clinical characteristics of participants not frail in the SNAP-3 cohort, with and without delirium 67](#_Toc211620435)

[Supplementary table 6E: Clinical characteristics of participants having elective surgery in the SNAP-3 cohort, with and without delirium 70](#_Toc211620436)

[Supplementary table 6F: Clinical characteristics of participants having non-elective surgery in the SNAP-3 cohort, with and without delirium 73](#_Toc211620437)

[Supplementary table 6G: Clinical characteristics of participants having inpatient surgery in the SNAP-3 cohort, with and without delirium 76](#_Toc211620438)

[Outcomes: subgroups 79](#_Toc211620439)

[Supplementary table 7A: Outcomes of participants aged ≥85 in the SNAP-3 cohort, with and without delirium 79](#_Toc211620440)

[Supplementary table 7B: Outcomes of participants aged <85 in the SNAP-3 cohort, with and without delirium 80](#_Toc211620441)

[Supplementary table 7C: Outcomes of participants living with frailty in the SNAP-3 cohort, with and without delirium 80](#_Toc211620442)

[Supplementary table 7D: Outcomes of participants not frail in the SNAP-3 cohort, with and without delirium 80](#_Toc211620443)

[Supplementary table 7E: Outcomes of participants having elective surgery in the SNAP-3 cohort, with and without delirium 81](#_Toc211620444)

[Supplementary table 7F: Outcomes of participants having non-elective surgery in the SNAP-3 cohort, with and without delirium 82](#_Toc211620445)

[Supplementary table 7G: Outcomes of participants having inpatient surgery in the SNAP-3 cohort, with and without delirium 82](#_Toc211620446)

[Supplementary results: main analysis 83](#_Toc211620447)

[Supplementary table 8: The impact of delirium on predicted outcomes, using adjusted models and multiple imputation via chained equations 83](#_Toc211620448)

[Supplementary table 9: Adjusted effect estimates from quantile regression with delirium as main exposure and median length of stay as outcome 84](#_Toc211620449)

[Supplementary table 10: Adjusted effect estimates from quantile regression with delirium as main exposure and 80^th^ percentile length of stay as outcome 86](#_Toc211620450)

[Supplementary table 11: Adjusted odds ratios from mixed effects logistic regression with delirium as main exposure and postoperative morbidity as outcome 88](#_Toc211620451)

[Supplementary table 12: Adjusted odds ratios from mixed effects logistic regression with delirium as main exposure and 30-day mortality as outcome 90](#_Toc211620452)

[Supplementary table 13: Adjusted odds ratios from mixed effects logistic regression with delirium as main exposure and 120-day mortality as outcome 92](#_Toc211620453)

[Supplementary table 14: Adjusted odds ratios from mixed effects logistic regression with delirium as main exposure and one-year mortality as outcome 94](#_Toc211620454)

[Supplementary results: sensitivity analyses 96](#_Toc211620455)

[Supplementary table 15: Effect estimates comparing the effect of postoperative delirium within 7 days versus no delirium, complete cases only. 96](#_Toc211620456)

[Supplementary table 16: Effect estimates of comparing the effect of postoperative delirium within 7 days versus no delirium, inpatient cases only. 96](#_Toc211620457)

[SNAP-3 Collaborators 96](#_Toc211620458)

[Supplementary table 17: SNAP-3 collaborators (in separate file) 96](#_Toc211620459)

[Supplementary figures 97](#_Toc211620460)

[Supplementary figure 1: participant flow diagram 97](#_Toc211620461)

[Supplementary figure 2: directed acyclic graph for the relationship between delirium with postoperative length of stay 98](#_Toc211620462)

[Supplementary figure 3: directed acyclic graph for the relationship between delirium with postoperative morbidity 99](#_Toc211620463)

[Supplementary figure 4: directed acyclic graph for the relationship between delirium with postoperative mortality 100](#_Toc211620464)

### Supplementary tables

#### Supplementary table 1: STROBE checklist

STROBE Statement—Checklist of items that should be included in reports of ***cohort studies***

|  | Item No | Recommendation | SNAP-3 |
| --- | --- | --- | --- |
| **Title and abstract** | 1 | (*a*) Indicate the study’s design with a commonly used term in the title or the abstract | Title page |
|  |  | (*b*) Provide in the abstract an informative and balanced summary of what was done and what was found | Abstract |
| Introduction | | |  |
| Background/rationale | 2 | Explain the scientific background and rationale for the investigation being reported | Introduction |
| Objectives | 3 | State specific objectives, including any prespecified hypotheses | Introduction |
| Methods | | |  |
| Study design | 4 | Present key elements of study design early in the paper | Methods and previously published protocol |
| Setting | 5 | Describe the setting, locations, and relevant dates, including periods of recruitment, exposure, follow-up, and data collection | Methods – first paragraph |
| Participants | 6 | (*a*) Give the eligibility criteria, and the sources and methods of selection of participants. Describe methods of follow-up | Supplementary information (table S1) previously published protocol |
|  |  | (*b*) For matched studies, give matching criteria and number of exposed and unexposed | NA |
| Variables | 7 | Clearly define all outcomes, exposures, predictors, potential confounders, and effect modifiers. Give diagnostic criteria, if applicable | Methods section plus previously published protocol |
| Data sources/ measurement | 8* | For each variable of interest, give sources of data and details of methods of assessment (measurement). Describe comparability of assessment methods if there is more than one group | Methods section plus previously published protocol |
| Bias | 9 | Describe any efforts to address potential sources of bias | Methods section plus previously published protocol. Causal diagrams (DAGs) in methods section, Figure S2 |
| Study size | 10 | Explain how the study size was arrived at | Methods section plus previously published protocol |
| Quantitative variables | 11 | Explain how quantitative variables were handled in the analyses. If applicable, describe which groupings were chosen and why | Methods section plus previously published protocol) |
| Statistical methods | 12 | (*a*) Describe all statistical methods, including those used to control for confounding | Methods section plus previously published protocol |
|  |  | (*b*) Describe any methods used to examine subgroups and interactions | NA |
|  |  | (*c*) Explain how missing data were addressed | Methods section, table S4 |
|  |  | (*d*) If applicable, explain how loss to follow-up was addressed | Methods section plus previously published protocol |
|  |  | (*e*) Describe any sensitivity analyses | NA |
| Results | | |  |
| Participants | 13* | (a) Report numbers of individuals at each stage of study—eg numbers potentially eligible, examined for eligibility, confirmed eligible, included in the study, completing follow-up, and analysed | Results and figure S1 |
|  |  | (b) Give reasons for non-participation at each stage | Results and figure S1 |
|  |  | (c) Consider use of a flow diagram | Figure S1 |
| Descriptive data | 14* | (a) Give characteristics of study participants (eg demographic, clinical, social) and information on exposures and potential confounders | Table 1, supplementary tables in full detail. Previously published SNAP3 papers |
|  |  | (b) Indicate number of participants with missing data for each variable of interest | Table 1, supplementary tables in full detail. Previously published SNAP3 papers |
|  |  | (c) Summarise follow-up time (eg, average and total amount) | Explicit in outcomes (defined times) |
| Outcome data | 15* | Report numbers of outcome events or summary measures over time | Table 2, supplementary tables in full detail. |
| Main results | 16 | (*a*) Give unadjusted estimates and, if applicable, confounder-adjusted estimates and their precision (eg, 95% confidence interval). Make clear which confounders were adjusted for and why they were included | Results, table 2, supplementary outcomes tables S10-16 |
|  |  | (*b*) Report category boundaries when continuous variables were categorized | Table 1 and 2, methods |
|  |  | (*c*) If relevant, consider translating estimates of relative risk into absolute risk for a meaningful time period | NA |
| Other analyses | 17 | Report other analyses done—eg analyses of subgroups and interactions, and sensitivity analyses | Results and supplementary information S17 & 18 |
| Discussion | | |  |
| Key results | 18 | Summarise key results with reference to study objectives | Discussion |
| Limitations | 19 | Discuss limitations of the study, taking into account sources of potential bias or imprecision. Discuss both direction and magnitude of any potential bias | Discussion – middle section |
| Interpretation | 20 | Give a cautious overall interpretation of results considering objectives, limitations, multiplicity of analyses, results from similar studies, and other relevant evidence | Discussion final section |
| Generalisability | 21 | Discuss the generalisability (external validity) of the study results | Discussion |
| Other information | | |  |
| Funding | 22 | Give the source of funding and the role of the funders for the present study and, if applicable, for the original study on which the present article is based | Details provided after main body of paper |

*Give information separately for exposed and unexposed groups.

**Note:** An Explanation and Elaboration article discusses each checklist item and gives methodological background and published examples of transparent reporting. The STROBE checklist is best used in conjunction with this article (freely available on the Web sites of PLoS Medicine at http://www.plosmedicine.org/, Annals of Internal Medicine at http://www.annals.org/, and Epidemiology at http://www.epidem.com/). Information on the STROBE Initiative is available at <http://www.strobe-statement.org>.

#### Supplementary table 2: Prevalence of missing data in SNAP-3

| **Variable of interest** | **Missing data (%)** |
| --- | --- |
|  |  |
| Number of comorbidities | 831 (11.7%) |
| Preoperative assessment clinic | 340 (4.8%) |
| Reported Edmonton Frailty Scale | 304 (4.3%) |
| ADLs | 288 (4.0%) |
| Postoperative length of stay (outcome) | 230 (3.2%) |
| IMD deciles | 206 (2.9%) |
| Anaesthetic technique | 202 (2.8%) |
| Surgical specialty | 176 (2.5%) |
| Surgical Outcome Risk Tool (SORT) predicted morbidity | 174 (2.4%) |
| Operative severity | 171 (2.4%) |
| Surgical procedure | 165 (2.3%) |
| Preoperative clinic | 162 (2.3%) |
| Polypharmacy | 141 (2.0%) |
| CFS | 137 (1.9%) |
| ASA | 135 (1.9%) |
| BMI | 127 (1.8%) |
| Postoperative mortality (outcome) | 99 (1.4%) |
| Surgical Outcome Risk Tool (SORT) predicted mortality | 88 (1.2%) |
| Education | 80 (1.1%) |
| Age | 78 (1.1%) |
| Ethnicity | 78 (1.1%) |
| Sex assigned at birth | 74 (1.0%) |
| Urgency | 74 (1.0%) |
| Hearing impairment | 73 (1.0%) |
| Visual impairment | 73 (1.0%) |
| Dementia | 73 (1.0%) |
| Postoperative morbidity (outcome) | 0 (0.0%) |

Notes: Number of participants (of 7128 total) with missing data for each variable of interest. The methodology of this study was to assume that a participant did not have delirium or morbidity if they were day case patients. If a participant was admitted overnight, they were recorded as not having delirium or morbidity if the clinical tools and/or notes reviews didn’t detect them. ASA: American Society of Anesthesiologists physical status; CFS: Clinical Frailty Scale; ADLs: activities of daily living; IMD: Index of Multiple Deprivation deciles (IMD10 , least deprived); BMI: Body Mass Index (kg.m^-2^).

### Demographics: whole cohort

Percentages have been rounded so may not total 100% exactly. Missing data are omitted from this table but reported in Supplementary table 4. Surgical urgency is defined using NCEPOD categorisations. The values here are proportions with 95% confidence intervals (95% CI) unless stated. 95% CI are calculated using the percentile method and 2000 bootstraps. Surgical urgency is described using the NCEPOD criteria.^1^ ASA: American Society of Anesthesiologists physical status; CFS: Clinical Frailty Scale; ADLs: activities of daily living; IMD: Index of Multiple Deprivation deciles (IMD10 , least deprived); Operative severity is defined by AXA procedural codes; AHP: allied health professional (e.g. physiotherapist); MDT: multidisciplinary team; PACU: Post-anaesthesia care unit;

#### Supplementary table 3: Demographics of SNAP-3 cohort, with and without delirium

|  |  | **All** | | **Delirium** | | **No delirium** | |
| --- | --- | --- | --- | --- | --- | --- | --- |
| **Characteristic** |  | **Summary** | **Number** | **Summary** | **Number** | **Summary** | **Number** |
| Consent |  |  |  |  |  |  |  |
|  | Capacity | 96.6 (96.2-97) % | 6885/7128 | 70.8 (66.8-74.9) % | 339/479 | 98.5 (98.2-98.7) % | 6547/6650 |
|  | Consultee | 3.4 (3-3.8) % | 243/7128 | 29.2 (25.1-33.4) % | 140/479 | 1.5 (1.2-1.8) % | 103/6650 |
| Surgical specialty |  |  |  |  |  |  |  |
|  | Bariatric | 0.1 (0.1-0.2) % | 10/6959 | NA | NA | 0.2 (0.1-0.3) % | 10/6483 |
|  | Breast | 5.5 (5-6.1) % | 386/6959 | 0.2 (0-0.6) % | 1/476 | 5.9 (5.4-6.5) % | 385/6483 |
|  | Cardiology | 0 (0-0.1) % | 2/6959 | NA | NA | 0 (0-0.1) % | 2/6483 |
|  | Colorectal | 12.2 (11.5-13) % | 850/6959 | 9.5 (6.9-12.2) % | 45/476 | 12.4 (11.6-13.3) % | 805/6483 |
|  | Ear, nose & throat | 3.7 (3.3-4.2) % | 259/6959 | 0.4 (0-1.1) % | 2/476 | 4 (3.5-4.5) % | 257/6483 |
|  | Endocrine | 0.9 (0.7-1.1) % | 61/6959 | 0.2 (0-0.6) % | 1/476 | 0.9 (0.7-1.2) % | 60/6483 |
|  | Endoscopic | 0.4 (0.2-0.5) % | 25/6959 | 0.2 (0-0.6) % | 1/476 | 0.4 (0.2-0.5) % | 24/6483 |
|  | Gynaecology | 4.8 (4.4-5.4) % | 337/6959 | 0.8 (0.2-1.7) % | 4/476 | 5.1 (4.6-5.6) % | 333/6483 |
|  | Hepato-pancreato-biliary | 0.6 (0.4-0.8) % | 43/6959 | 1.9 (0.8-3.2) % | 9/476 | 0.5 (0.4-0.7) % | 34/6483 |
|  | Interventional radiology | 1.1 (0.9-1.4) % | 78/6959 | 1.9 (0.8-3.2) % | 9/476 | 1.1 (0.8-1.3) % | 69/6483 |
|  | Maxillo-facial and dental | 1.3 (1.1-1.6) % | 93/6959 | 1.1 (0.2-2.1) % | 5/476 | 1.4 (1.1-1.6) % | 88/6483 |
|  | Neurosurgery | 0.9 (0.7-1.2) % | 66/6959 | 2.5 (1.3-4) % | 12/476 | 0.8 (0.6-1) % | 54/6483 |
|  | Ophthalmic | 1.2 (0.9-1.5) % | 83/6959 | NA | NA | 1.3 (1-1.6) % | 83/6483 |
|  | Orthopaedics | 29.7 (28.7-30.8) % | 2070/6959 | 56.5 (52.1-61.1) % | 269/476 | 27.8 (26.7-28.9) % | 1801/6483 |
|  | Plastics | 3.8 (3.4-4.3) % | 266/6959 | 2.3 (1.1-3.8) % | 11/476 | 3.9 (3.5-4.4) % | 255/6483 |
|  | Spine | 2.1 (1.7-2.4) % | 143/6959 | 2.5 (1.3-4) % | 12/476 | 2 (1.7-2.4) % | 131/6483 |
|  | Thoracic | 4.2 (3.7-4.6) % | 290/6959 | 8.2 (5.9-10.7) % | 39/476 | 3.9 (3.4-4.3) % | 251/6483 |
|  | Transplant | 0 (0-0.1) % | 3/6959 | 0.2 (0-0.6) % | 1/476 | 0 (0-0.1) % | 2/6483 |
|  | Upper gastrointestinal | 5.3 (4.8-5.8) % | 368/6959 | 4.2 (2.5-6.1) % | 20/476 | 5.4 (4.8-6) % | 348/6483 |
|  | Urology | 19.2 (18.3-20.1) % | 1334/6959 | 5 (3.2-7.1) % | 24/476 | 20.2 (19.3-21.2) % | 1310/6483 |
|  | Vascular | 2.7 (2.3-3) % | 186/6959 | 2.3 (1.1-3.8) % | 11/476 | 2.7 (2.3-3.1) % | 175/6483 |
|  | Other | 0 (0-0.1) % | 2/6959 | NA | NA | 0 (0-0.1) % | 2/6483 |
|  | Unknown | 0.1 (0-0.1) % | 4/6959 | NA | NA | 0.1 (0-0.1) % | 4/6483 |
| Source of admission |  |  |  |  |  |  |  |
|  | Own home | 95.1 (94.6-95.6) % | 6705/7051 | 82 (78.7-85.6) % | 393/479 | 96 (95.6-96.5) % | 6312/6572 |
|  | Sheltered housing | 1.4 (1.1-1.7) % | 100/7051 | 1.9 (0.8-3.1) % | 9/479 | 1.4 (1.1-1.7) % | 91/6572 |
|  | Residential or nursing home | 1.5 (1.3-1.8) % | 109/7051 | 12.1 (9.2-15.2) % | 58/479 | 0.8 (0.6-1) % | 51/6572 |
|  | Rehabilitation facility | 0.1 (0-0.2) % | 8/7051 | 0.6 (0-1.5) % | 3/479 | 0.1 (0-0.2) % | 5/6572 |
|  | Another secondary care hospital | 0.6 (0.5-0.8) % | 44/7051 | 2.1 (0.8-3.5) % | 10/479 | 0.5 (0.3-0.7) % | 34/6572 |
|  | Other | 0.9 (0.7-1.1) % | 62/7051 | 1.3 (0.4-2.3) % | 6/479 | 0.9 (0.6-1.1) % | 56/6572 |
|  | Unknown | 0.3 (0.2-0.5) % | 23/7051 | NA | NA | 0.3 (0.2-0.5) % | 23/6572 |
| Independence |  |  |  |  |  |  |  |
|  | Independent | 72.9 (71.8-73.9) % | 4989/6842 | 38.8 (34.4-43.5) % | 165/425 | 75.2 (74.2-76.2) % | 4824/6417 |
|  | Assistance with instrumental ADLs | 23.8 (22.9-24.8) % | 1631/6842 | 46.6 (41.9-51.5) % | 198/425 | 22.3 (21.3-23.4) % | 1433/6417 |
|  | Assistance with basic ADLs | 3.2 (2.8-3.7) % | 222/6842 | 14.6 (11.5-17.9) % | 62/425 | 2.5 (2.1-2.9) % | 160/6417 |
| Ethnicity |  |  |  |  |  |  |  |
|  | Asian | 2 (1.6-2.3) % | 138/6973 | 1.9 (0.8-3.2) % | 9/472 | 2 (1.7-2.3) % | 129/6501 |
|  | Black | 1.4 (1.2-1.7) % | 100/6973 | 1.5 (0.4-2.8) % | 7/472 | 1.4 (1.1-1.7) % | 93/6501 |
|  | Mixed | 0.5 (0.4-0.7) % | 36/6973 | 0.2 (0-0.6) % | 1/472 | 0.5 (0.4-0.7) % | 35/6501 |
|  | White | 96.1 (95.6-96.5) % | 6699/6973 | 96.4 (94.5-97.9) % | 455/472 | 96 (95.6-96.5) % | 6244/6501 |
| Education |  |  |  |  |  |  |  |
|  | Degree level | 22.7 (21.8-23.7) % | 1600/7049 | 15.7 (12.6-19) % | 75/478 | 23.2 (22.1-24.2) % | 1525/6571 |
|  | A levels / NVQ 3 | 12.1 (11.3-12.9) % | 854/7049 | 9.6 (7.1-12.3) % | 46/478 | 12.3 (11.5-13.1) % | 808/6571 |
|  | Apprenticeship | 6.6 (6.1-7.2) % | 468/7049 | 6.5 (4.4-8.8) % | 31/478 | 6.7 (6.1-7.3) % | 437/6571 |
|  | GCSEs / NVQ 2 | 14.2 (13.4-15.1) % | 1003/7049 | 11.1 (8.4-14) % | 53/478 | 14.5 (13.6-15.3) % | 950/6571 |
|  | O level / NVQ 1 | 11.7 (10.9-12.5) % | 825/7049 | 10.5 (7.7-13.4) % | 50/478 | 11.8 (11-12.6) % | 775/6571 |
|  | No formal qualifications | 22.7 (21.7-23.6) % | 1598/7049 | 27 (23.2-31) % | 129/478 | 22.4 (21.3-23.3) % | 1469/6571 |
|  | Education not recorded | 9.9 (9.3-10.6) % | 701/7049 | 19.7 (16.3-23.2) % | 94/478 | 9.2 (8.5-9.9) % | 607/6571 |
| IMD deciles |  |  |  |  |  |  |  |
|  | 1 (most deprived) | 6.8 (6.2-7.5) % | 469/6878 | 8.5 (6.1-11.1) % | 39/458 | 6.7 (6-7.3) % | 430/6420 |
|  | 2 | 7.6 (7-8.2) % | 520/6878 | 7.2 (4.8-9.6) % | 33/458 | 7.6 (7-8.2) % | 487/6420 |
|  | 3 | 8.2 (7.6-8.9) % | 566/6878 | 7.9 (5.5-10.5) % | 36/458 | 8.3 (7.6-8.9) % | 530/6420 |
|  | 4 | 8.9 (8.2-9.6) % | 612/6878 | 9.4 (6.8-12.2) % | 43/458 | 8.9 (8.1-9.6) % | 569/6420 |
|  | 5 | 9.9 (9.3-10.7) % | 684/6878 | 9 (6.3-11.8) % | 41/458 | 10 (9.3-10.7) % | 643/6420 |
|  | 6 | 12.1 (11.3-12.8) % | 829/6878 | 13.1 (10-16.4) % | 60/458 | 12 (11.2-12.8) % | 769/6420 |
|  | 7 | 11.8 (11-12.6) % | 811/6878 | 12 (9.2-15.1) % | 55/458 | 11.8 (11-12.6) % | 756/6420 |
|  | 8 | 11.4 (10.7-12.2) % | 784/6878 | 12.2 (9.4-15.3) % | 56/458 | 11.3 (10.6-12.1) % | 728/6420 |
|  | 9 | 12.4 (11.7-13.2) % | 855/6878 | 11.8 (9-14.8) % | 54/458 | 12.5 (11.7-13.3) % | 801/6420 |
|  | 10 (least deprived) | 10.9 (10.1-11.6) % | 748/6878 | 9 (6.3-11.6) % | 41/458 | 11 (10.3-11.8) % | 707/6420 |
| Operative severity |  |  |  |  |  |  |  |
|  | Minor | 6.2 (5.6-6.8) % | 432/6958 | 2.1 (0.8-3.6) % | 10/476 | 6.5 (5.9-7.1) % | 422/6482 |
|  | Intermediate | 25.6 (24.6-26.7) % | 1784/6958 | 8.8 (6.3-11.3) % | 42/476 | 26.9 (25.8-28) % | 1742/6482 |
|  | Major | 30.9 (29.7-32) % | 2147/6958 | 34.7 (30.5-39.1) % | 165/476 | 30.6 (29.4-31.7) % | 1982/6482 |
|  | X Major | 25 (24-26) % | 1737/6958 | 35.1 (30.9-39.3) % | 167/476 | 24.2 (23.1-25.3) % | 1570/6482 |
|  | Complex | 12.3 (11.5-13) % | 854/6958 | 19.3 (16-22.9) % | 92/476 | 11.8 (11-12.5) % | 762/6482 |
|  | Unknown | 0.1 (0-0.1) % | 4/6958 | NA | NA | 0.1 (0-0.1) % | 4/6482 |
| Preop assessment |  |  |  |  |  |  |  |
|  | Nurse or AHP led clinic | 35.4 (34.2-36.5) % | 2400/6789 | 15.5 (12.1-18.9) % | 69/445 | 36.7 (35.6-37.9) % | 2331/6344 |
|  | Anaesthetist led clinic | 7.2 (6.6-7.8) % | 490/6789 | 5.2 (3.1-7.4) % | 23/445 | 7.4 (6.7-8) % | 467/6344 |
|  | Anaesthetist and geriatrician led clinics | 0.2 (0.1-0.3) % | 14/6789 | 0.2 (0-0.7) % | 1/445 | 0.2 (0.1-0.3) % | 13/6344 |
|  | Geriatrician led or MDT clinic | 0.9 (0.7-1.2) % | 64/6789 | 1.1 (0.2-2.2) % | 5/445 | 0.9 (0.7-1.2) % | 59/6344 |
|  | Physician (non geriatrician) led clinic | 1.1 (0.9-1.4) % | 76/6789 | 0.9 (0.2-1.8) % | 4/445 | 1.1 (0.9-1.4) % | 72/6344 |
|  | On the day assessment | 55.2 (54-56.4) % | 3745/6789 | 77.1 (73-80.7) % | 343/445 | 53.6 (52.4-54.8) % | 3402/6344 |
| Postop destination |  |  |  |  |  |  |  |
|  | Ward (level 0 or 1 care) | 86 (85.1-86.7) % | 6054/7043 | 73.4 (69.5-77.2) % | 351/478 | 86.9 (86.1-87.7) % | 5703/6565 |
|  | Unplanned admission to PACU or equivalent (level 1.5 care) | 0.5 (0.3-0.7) % | 35/7043 | 1 (0.2-2.1) % | 5/478 | 0.5 (0.3-0.6) % | 30/6565 |
|  | Planned admission to PACU or equivalent (level 1.5 care) | 5.9 (5.4-6.5) % | 418/7043 | 5.4 (3.6-7.5) % | 26/478 | 6 (5.4-6.5) % | 392/6565 |
|  | Unplanned admission to PACU or equivalent (level 2/3 care) | 0.2 (0.1-0.3) % | 13/7043 | 1 (0.2-2.1) % | 5/478 | 0.1 (0-0.2) % | 8/6565 |
|  | Planned admission to PACU or equivalent (level 2/3 care) | 1.4 (1.2-1.7) % | 101/7043 | 2.7 (1.5-4.2) % | 13/478 | 1.3 (1.1-1.6) % | 88/6565 |
|  | Unplanned critical care admission (level 2 or 3 care) | 0.7 (0.5-0.9) % | 51/7043 | 3.3 (1.9-5) % | 16/478 | 0.5 (0.4-0.7) % | 35/6565 |
|  | Planned critical care admission (level 2 or 3 care) | 5 (4.5-5.6) % | 355/7043 | 13 (10-16.1) % | 62/478 | 4.5 (4-5) % | 293/6565 |
| Urinary catheter |  |  |  |  |  |  |  |
|  | No catheter | 63.1 (62-64.2) % | 4442/7044 | 36.1 (31.7-40.5) % | 173/479 | 65 (63.9-66.2) % | 4269/6565 |
|  | Long-term/pre-admission catheter | 2.1 (1.8-2.4) % | 148/7044 | 5.6 (3.8-7.7) % | 27/479 | 1.8 (1.5-2.2) % | 121/6565 |
|  | Electively catheterised pre/intra-op | 30.8 (29.9-31.9) % | 2173/7044 | 53 (48.6-57.6) % | 254/479 | 29.2 (28.1-30.4) % | 1919/6565 |
|  | Catheterised post-op | 2 (1.7-2.3) % | 140/7044 | 3.8 (2.1-5.6) % | 18/479 | 1.9 (1.5-2.2) % | 122/6565 |

### Clinical characteristics: whole cohort

Percentages have been rounded so may not total 100% exactly. Missing data are omitted from this table but reported in Supplementary table 4. Surgical urgency is defined using NCEPOD categorisations. The values here are proportions with 95% confidence intervals (95% CI) unless stated. 95% CI are calculated using the percentile method and 2000 bootstraps. SORT: Surgical Outcome Risk Tool; BMI: Body Mass Index (kg.m^-2^); COPD: Chronic obstructive pulmonary disease; WCC: white cell (leucocyte) count; eGFR: estimated glomerular filtration rate

#### Supplementary table 4: Clinical characteristics of SNAP-3 cohort, with and without delirium

|  |  | **All** | | **Delirium** | | **No delirium** | |
| --- | --- | --- | --- | --- | --- | --- | --- |
| **Characteristic** |  | **Summary** | **Number** | **Summary** | **Number** | **Summary** | **Number** |
| Number of comorbidities | Total comorbidities | 2 (1-3) | 6298 | 3 (2-4) | 460 | 2 (1-3) | 5838 |
| SORT predicted morbidity (%) | Predicted morbidity | 22.7 (22.3-23.1) | 6955 | 31.7 (30-33.4) | 475 | 22.1 (21.7-22.5) | 6480 |
| SORT predicted mortality (%) | Predicted mortality | 1.9 (1.8-2) | 7041 | 6.3 (5.6-7.1) | 478 | 1.5 (1.5-1.6) | 6563 |
| Polypharmacy |  | 47 (45.8-48.1) % | 3282/6988 | 65.5 (61-69.5) % | 309/472 | 45.6 (44.4-46.8) % | 2973/6516 |
| BMI category |  |  |  |  |  |  |  |
|  | Underweight | 1.9 (1.6-2.3) % | 136/6995 | 6.6 (4.5-8.9) % | 31/471 | 1.6 (1.3-1.9) % | 105/6524 |
|  | Healthy weight | 29.9 (28.8-31) % | 2094/6995 | 43.7 (39.3-48.2) % | 206/471 | 28.9 (27.9-30) % | 1888/6524 |
|  | Overweight | 36 (34.8-37.1) % | 2515/6995 | 29.5 (25.5-33.8) % | 139/471 | 36.4 (35.3-37.6) % | 2376/6524 |
|  | Obese class 1 | 28.6 (27.6-29.7) % | 1999/6995 | 18.9 (15.5-22.5) % | 89/471 | 29.3 (28.2-30.4) % | 1910/6524 |
|  | Obese class >=2 | 3.6 (3.2-4) % | 251/6995 | 1.3 (0.4-2.3) % | 6/471 | 3.8 (3.3-4.2) % | 245/6524 |
| Hearing impairment |  | 8.5 (7.8-9.1) % | 597/7056 | 13.8 (10.6-16.9) % | 66/479 | 8.1 (7.4-8.7) % | 531/6577 |
| Visual impairment |  | 2.6 (2.3-3) % | 186/7056 | 6.5 (4.4-8.8) % | 31/479 | 2.4 (2-2.7) % | 155/6577 |
| Dementia |  | 2.6 (2.2-3) % | 184/7056 | 21.1 (17.3-24.8) % | 101/479 | 1.3 (1-1.5) % | 83/6577 |
| Myocardial infarction |  | 9.7 (9-10.4) % | 682/7056 | 12.1 (9.2-15) % | 58/479 | 9.5 (8.8-10.2) % | 624/6577 |
| Heart failure |  | 5 (4.5-5.5) % | 352/7056 | 10.9 (8.1-13.8) % | 52/479 | 4.6 (4.1-5.1) % | 300/6577 |
| Atrial fibrillation |  | 11 (10.2-11.7) % | 775/7056 | 23 (19.4-26.7) % | 110/479 | 10.1 (9.4-10.8) % | 665/6577 |
| Valvular heart disease |  | 4.5 (4-5) % | 315/7056 | 8.4 (5.8-11.1) % | 40/479 | 4.2 (3.7-4.6) % | 275/6577 |
| Hypertension |  | 48.7 (47.5-49.9) % | 3438/7056 | 54.9 (50.3-59.3) % | 263/479 | 48.3 (47.1-49.5) % | 3175/6577 |
| Peripheral vascular disease |  | 4.6 (4.1-5.1) % | 326/7056 | 6.7 (4.6-9) % | 32/479 | 4.5 (4-5) % | 294/6577 |
| COPD |  | 9.5 (8.8-10.2) % | 673/7056 | 11.9 (9-14.8) % | 57/479 | 9.4 (8.7-10.1) % | 616/6577 |
| Chronic lung disease |  | 8.7 (8.1-9.4) % | 616/7056 | 9 (6.5-11.5) % | 43/479 | 8.7 (8-9.4) % | 573/6577 |
| OSA |  | 3 (2.6-3.5) % | 215/7056 | 1 (0.2-2.1) % | 5/479 | 3.2 (2.8-3.6) % | 210/6577 |
| Stroke |  | 8 (7.4-8.7) % | 568/7056 | 15.4 (12.1-18.8) % | 74/479 | 7.5 (6.9-8.2) % | 494/6577 |
| Hemiplegia |  | 0.8 (0.6-1) % | 53/7056 | 2.9 (1.7-4.6) % | 14/479 | 0.6 (0.4-0.8) % | 39/6577 |
| Anxiety / depression |  | 9.9 (9.2-10.6) % | 699/7056 | 15 (12.1-18.2) % | 72/479 | 9.5 (8.9-10.2) % | 627/6577 |
| Parkinson's disease |  | 0.9 (0.7-1.1) % | 65/7056 | 2.5 (1.3-4) % | 12/479 | 0.8 (0.6-1) % | 53/6577 |
| Diabetes |  |  |  |  |  |  |  |
|  | Present | 16.3 (15.5-17.3) % | 1153/7056 | 21.5 (18.2-25.3) % | 103/479 | 16 (15-16.8) % | 1050/6577 |
|  | Diabetes without chronic complications | 70.9 (68.3-73.5) % | 817/1152 | 64.1 (54.4-73.8) % | 66/103 | 71.6 (68.8-74.5) % | 751/1049 |
|  | Diabetes with chronic complications | 29.1 (26.5-31.7) % | 335/1152 | 35.9 (27.2-45.6) % | 37/103 | 28.4 (25.6-31.3) % | 298/1049 |
| Chronic kidney disease |  | 9.9 (9.2-10.6) % | 697/7056 | 16.3 (13.2-19.6) % | 78/479 | 9.4 (8.7-10.1) % | 619/6577 |
| Benign prostatic hyperplasia |  | 6.9 (6.3-7.5) % | 488/7056 | 5.2 (3.3-7.3) % | 25/479 | 7 (6.4-7.7) % | 463/6577 |
| Liver disease |  |  |  |  |  |  |  |
|  | Present | 1.7 (1.4-2) % | 122/7056 | 1.7 (0.6-2.9) % | 8/479 | 1.7 (1.4-2.1) % | 114/6577 |
|  | Mild liver disease | 1.5 (1.2-1.8) % | 107/7129 | 1.5 (0.4-2.7) % | 7/479 | 1.5 (1.2-1.8) % | 100/6650 |
|  | Moderate or severe liver disease | 0.2 (0.1-0.3) % | 13/7129 | 0.2 (0-0.6) % | 1/479 | 0.2 (0.1-0.3) % | 12/6650 |
| Peptic ulcer disease |  | 3.5 (3-3.9) % | 244/7056 | 3.8 (2.1-5.6) % | 18/479 | 3.4 (3-3.9) % | 226/6577 |
| Lymphoma |  | 1 (0.8-1.2) % | 69/7056 | 1.5 (0.4-2.5) % | 7/479 | 0.9 (0.7-1.2) % | 62/6577 |
| Leukaemia |  | 0.6 (0.4-0.8) % | 41/7056 | 0.6 (0-1.5) % | 3/479 | 0.6 (0.4-0.8) % | 38/6577 |
| Connective tissue disorders |  | 4.8 (4.3-5.3) % | 340/7056 | 3.3 (1.7-5) % | 16/479 | 4.9 (4.4-5.4) % | 324/6577 |
| Osteoarthritis |  | 29.2 (28.2-30.3) % | 2063/7056 | 29.2 (25.3-33.4) % | 140/479 | 29.2 (28.2-30.4) % | 1923/6577 |
| Malignancy |  |  |  |  |  |  |  |
|  | Present | 21.1 (20.1-22) % | 1487/7056 | 17.1 (14-20.5) % | 82/479 | 21.4 (20.4-22.3) % | 1405/6577 |
|  | Malignancy without metastasis | 17.6 (16.8-18.5) % | 1243/7056 | 13.4 (10.6-16.5) % | 64/479 | 17.9 (17-18.9) % | 1179/6577 |
|  | Malignancy with metastasis | 3.5 (3.1-4) % | 250/7056 | 3.8 (2.3-5.4) % | 18/479 | 3.5 (3.1-4) % | 232/6577 |
|  | Malignancy diagnosis ≤ 5 years | 16.4 (15.5-17.3) % | 1157/7056 | 12.7 (9.8-15.9) % | 61/479 | 16.7 (15.8-17.6) % | 1096/6577 |
|  | Malignancy diagnosis > 5 years | 4.9 (4.4-5.4) % | 344/7056 | 4.8 (2.9-6.9) % | 23/479 | 4.9 (4.4-5.4) % | 321/6577 |
| Haematology |  |  |  |  |  |  |  |
|  | Haemoglobin (g/l) | 132 (131.6-132.5) | 6387 | 122.6 (120.7-124.5) | 469 | 132.8 (132.4-133.3) | 5918 |
|  | WCC (x10^9/L) | 8.2 (8.1-8.3) | 6387 | 10.2 (9.8-10.7) | 469 | 8 (7.9-8.1) | 5918 |
|  | Neutrophil count (x10^9/L) | 5.5 (5.4-5.6) | 6348 | 7.8 (7.4-8.2) | 465 | 5.3 (5.3-5.4) | 5883 |
| Biochemistry |  |  |  |  |  |  |  |
|  | eGFR (ml/min) | 75 (61-88) | 6358 | 69.1 (55-84) | 460 | 75 (61-88) | 5898 |
|  | Sodium (mmol/l) | 139 (137-141) | 6420 | 138 (136-141) | 468 | 139 (137-141) | 5952 |
|  | Potassium (mmol/l) | 4.4 (4.4-4.4) | 6390 | 4.3 (4.2-4.3) | 465 | 4.4 (4.4-4.4) | 5925 |
|  | Creatinine (micromol/l) | 77 (64-94) | 6411 | 79 (62-99) | 468 | 77 (64-94) | 5943 |

### Demographics: subgroups

Percentages have been rounded so may not total 100% exactly. Missing data are omitted from this table but reported in Supplementary table 4. Surgical urgency is defined using NCEPOD categorisations. The values here are proportions with 95% confidence intervals (95% CI) unless stated. 95% CI are calculated using the percentile method and 2000 bootstraps. Surgical urgency is described using the NCEPOD criteria.^1^ ASA: American Society of Anesthesiologists physical status; CFS: Clinical Frailty Scale; ADLs: activities of daily living; IMD: Index of Multiple Deprivation deciles (IMD10 , least deprived); Operative severity is defined by AXA procedural codes; AHP: allied health professional (e.g. physiotherapist); MDT: multidisciplinary team; PACU: Post-anaesthesia care unit;

#### Supplementary table 5A: Demographics of participants aged ≥85 in the SNAP-3 cohort, with and without delirium

|  |  | **All** | | **Delirium** | | **No delirium** | |
| --- | --- | --- | --- | --- | --- | --- | --- |
| **Characteristic** |  | **Summary** | **Number** | **Summary** | **Number** | **Summary** | **Number** |
| Sex |  |  |  |  |  |  |  |
|  | Female | 53.4 (49.8-56.9) % | 398/745 | 66.3 (58.9-73.6) % | 108/163 | 49.8 (45.9-54) % | 290/582 |
|  | Male | 46.6 (42.8-50.1) % | 347/745 | 33.7 (26.4-41.1) % | 55/163 | 50.2 (45.9-54.1) % | 292/582 |
| Age |  |  |  |  |  |  |  |
|  | Age (years) | 88 (3.5) | 745 | 89.4 (4.1) | 163 | 87.6 (3.2) | 582 |
|  | 80-89 | 69.1 (65.6-72.5) % | 515/745 | 52.1 (44.2-59.5) % | 85/163 | 73.9 (70.4-77.5) % | 430/582 |
|  | >90 | 30.9 (27.4-34.2) % | 230/745 | 47.9 (40.5-55.8) % | 78/163 | 26.1 (22.5-29.6) % | 152/582 |
| ASA |  |  |  |  |  |  |  |
|  | 1 | 0.9 (0.3-1.6) % | 7/737 | NA | NA | 1.2 (0.3-2.3) % | 7/574 |
|  | 2 | 31.3 (28-34.7) % | 231/737 | 12.9 (8-18.4) % | 21/163 | 36.6 (32.8-40.4) % | 210/574 |
|  | 3 | 56.9 (53.2-60.2) % | 419/737 | 64.4 (57.1-71.2) % | 105/163 | 54.7 (50.7-58.9) % | 314/574 |
|  | 4 | 10.4 (8.3-12.8) % | 77/737 | 22.1 (16-28.2) % | 36/163 | 7.1 (5.1-9.2) % | 41/574 |
|  | 5 | 0.4 (0-0.9) % | 3/737 | 0.6 (0-1.8) % | 1/163 | 0.3 (0-0.9) % | 2/574 |
| Frailty (CFS≥5) | Frail | 49.2 (45.7-53) % | 363/738 | 76.5 (70.4-83.3) % | 124/162 | 41.5 (37.3-45.3) % | 239/576 |
| Clinical Frailty Scale |  |  |  |  |  |  |  |
|  | 1 | 2.2 (1.2-3.4) % | 16/738 | 0.6 (0-1.9) % | 1/162 | 2.6 (1.4-4) % | 15/576 |
|  | 2 | 7 (5.3-8.9) % | 52/738 | 3.1 (0.6-5.6) % | 5/162 | 8.2 (5.9-10.6) % | 47/576 |
|  | 3 | 17.3 (14.6-20.2) % | 128/738 | 5.6 (2.5-9.3) % | 9/162 | 20.7 (17.4-23.8) % | 119/576 |
|  | 4 | 24.3 (21.1-27.2) % | 179/738 | 14.2 (9.3-19.8) % | 23/162 | 27.1 (23.4-30.9) % | 156/576 |
|  | 5 | 22.4 (19.2-25.3) % | 165/738 | 25.9 (19.1-32.7) % | 42/162 | 21.4 (18.1-24.8) % | 123/576 |
|  | 6 | 14.5 (12.1-17.1) % | 107/738 | 22.8 (16.7-29.6) % | 37/162 | 12.2 (9.5-14.9) % | 70/576 |
|  | 7 | 10.8 (8.5-13.1) % | 80/738 | 23.5 (16.7-29.6) % | 38/162 | 7.3 (5.2-9.4) % | 42/576 |
|  | 8 | 1.5 (0.7-2.4) % | 11/738 | 4.3 (1.2-7.4) % | 7/162 | 0.7 (0.2-1.4) % | 4/576 |
| Multimorbidity (≥2 comorbidities) | Multimorbid | 80.5 (77.5-83.6) % | 559/694 | 86.7 (81-91.8) % | 137/158 | 78.7 (75.2-82.3) % | 422/536 |
| Number of comorbidities | Number of comorbidities | 3 (2-4) | 694 | 3 (2-4) | 158 | 3 (2-4) | 536 |
| Dementia | NA | 13.6 (11.3-16) % | 101/745 | 37.4 (30.1-45.4) % | 61/163 | 6.9 (4.8-8.9) % | 40/582 |
| Consent |  |  |  |  |  |  |  |
|  | Capacity | 83.9 (81.2-86.6) % | 625/745 | 52.1 (44.8-59.5) % | 85/163 | 92.8 (90.7-94.8) % | 540/582 |
|  | Consultee | 16.1 (13.7-18.8) % | 120/745 | 47.9 (39.9-55.2) % | 78/163 | 7.2 (5.2-9.5) % | 42/582 |
| Surgical specialty |  |  |  |  |  |  |  |
|  | Breast | 2.7 (1.6-3.9) % | 20/740 | NA | NA | 3.5 (2.1-5) % | 20/578 |
|  | Colorectal | 11.2 (9.1-13.5) % | 83/740 | 6.2 (2.5-9.9) % | 10/162 | 12.6 (10-15.4) % | 73/578 |
|  | Ear, nose & throat | 1.2 (0.5-2) % | 9/740 | NA | NA | 1.6 (0.7-2.6) % | 9/578 |
|  | Endoscopic | 0.1 (0-0.4) % | 1/740 | 0.6 (0-1.9) % | 1/162 | NA | NA |
|  | Gynaecology | 2 (1.1-3.1) % | 15/740 | NA | NA | 2.6 (1.4-4) % | 15/578 |
|  | Hepato-pancreato-biliary | 0.3 (0-0.7) % | 2/740 | 0.6 (0-1.9) % | 1/162 | 0.2 (0-0.5) % | 1/578 |
|  | Interventional radiology | 0.9 (0.3-1.8) % | 7/740 | 0.6 (0-1.9) % | 1/162 | 1 (0.3-1.9) % | 6/578 |
|  | Maxillo-facial and dental | 1.2 (0.5-2) % | 9/740 | 0.6 (0-2.5) % | 1/162 | 1.4 (0.5-2.4) % | 8/578 |
|  | Neurosurgery | 0.5 (0.1-1.1) % | 4/740 | 0.6 (0-1.9) % | 1/162 | 0.5 (0-1.2) % | 3/578 |
|  | Ophthalmic | 0.5 (0.1-1.1) % | 4/740 | NA | NA | 0.7 (0.2-1.4) % | 4/578 |
|  | Orthopaedics | 48.2 (44.6-51.8) % | 357/740 | 78.4 (72.2-84.6) % | 127/162 | 39.8 (35.8-43.8) % | 230/578 |
|  | Plastics | 5.1 (3.6-6.8) % | 38/740 | 3.1 (0.6-6.2) % | 5/162 | 5.7 (4-7.8) % | 33/578 |
|  | Spine | 0.7 (0.1-1.4) % | 5/740 | NA | NA | 0.9 (0.2-1.7) % | 5/578 |
|  | Thoracic | 2.4 (1.5-3.6) % | 18/740 | 1.9 (0-4.3) % | 3/162 | 2.6 (1.4-4) % | 15/578 |
|  | Upper gastrointestinal | 1.8 (0.8-2.7) % | 13/740 | 3.1 (0.6-6.2) % | 5/162 | 1.4 (0.5-2.4) % | 8/578 |
|  | Urology | 19.3 (16.5-22) % | 143/740 | 3.1 (0.6-6.2) % | 5/162 | 23.9 (20.6-27.5) % | 138/578 |
|  | Vascular | 1.6 (0.8-2.6) % | 12/740 | 1.2 (0-3.1) % | 2/162 | 1.7 (0.7-2.9) % | 10/578 |
| Surgical urgency |  |  |  |  |  |  |  |
|  | Emergency | 4.7 (3.2-6.3) % | 35/745 | 8.6 (4.3-12.9) % | 14/163 | 3.6 (2.2-5.2) % | 21/582 |
|  | Urgent | 35.6 (32.1-38.9) % | 265/745 | 61.3 (53.4-68.7) % | 100/163 | 28.4 (24.7-32.1) % | 165/582 |
|  | Expedited | 14.6 (12.3-17.2) % | 109/745 | 16.6 (11-22.1) % | 27/163 | 14.1 (11.3-17) % | 82/582 |
|  | Planned | 45.1 (41.6-48.6) % | 336/745 | 13.5 (8-19) % | 22/163 | 54 (50-57.7) % | 314/582 |
| Source of admission |  |  |  |  |  |  |  |
|  | Own home | 85.8 (83.4-88.3) % | 639/745 | 69.9 (63.2-77.3) % | 114/163 | 90.2 (87.6-92.6) % | 525/582 |
|  | Sheltered housing | 3.2 (2-4.6) % | 24/745 | 2.5 (0.6-4.9) % | 4/163 | 3.4 (2.1-5) % | 20/582 |
|  | Residential or nursing home | 7.9 (6-9.9) % | 59/745 | 21.5 (15.3-28.2) % | 35/163 | 4.1 (2.6-5.8) % | 24/582 |
|  | Rehabilitation facility | 0.5 (0.1-1.1) % | 4/745 | 1.8 (0-4.3) % | 3/163 | 0.2 (0-0.5) % | 1/582 |
|  | Another secondary care hospital | 0.5 (0.1-1.1) % | 4/745 | 1.2 (0-3.1) % | 2/163 | 0.3 (0-0.9) % | 2/582 |
|  | Other | 1.7 (0.8-2.8) % | 13/745 | 3.1 (0.6-5.5) % | 5/163 | 1.4 (0.5-2.4) % | 8/582 |
|  | Unknown | 0.3 (0-0.7) % | 2/745 | NA | NA | 0.3 (0-0.9) % | 2/582 |
| Independence |  |  |  |  |  |  |  |
|  | Independent | 42.1 (38.4-45.9) % | 289/687 | 18.2 (12.1-25) % | 24/132 | 47.7 (43.4-52.1) % | 265/555 |
|  | Assistance with instrumental ADLs | 49.8 (46-53.6) % | 342/687 | 61.4 (52.3-69.7) % | 81/132 | 47 (42.7-51) % | 261/555 |
|  | Assistance with basic ADLs | 8.2 (6.1-10.3) % | 56/687 | 20.5 (13.6-27.3) % | 27/132 | 5.2 (3.4-7.2) % | 29/555 |
| Ethnicity |  |  |  |  |  |  |  |
|  | Asian | 0.3 (0-0.7) % | 2/743 | 0.6 (0-1.8) % | 1/163 | 0.2 (0-0.5) % | 1/580 |
|  | Black | 0.8 (0.3-1.5) % | 6/743 | 0.6 (0-1.8) % | 1/163 | 0.9 (0.2-1.7) % | 5/580 |
|  | Mixed | 0.1 (0-0.4) % | 1/743 | NA | NA | 0.2 (0-0.5) % | 1/580 |
|  | White | 98.8 (98-99.5) % | 734/743 | 98.8 (96.9-100) % | 161/163 | 98.8 (97.8-99.7) % | 573/580 |
| Education |  |  |  |  |  |  |  |
|  | Degree level | 14.2 (11.8-16.7) % | 106/744 | 11.7 (7.4-17.3) % | 19/162 | 14.9 (12-18) % | 87/582 |
|  | A levels / NVQ 3 | 9.5 (7.4-11.7) % | 71/744 | 9.9 (5.6-14.8) % | 16/162 | 9.5 (7.2-12) % | 55/582 |
|  | Apprenticeship | 6.9 (5.1-8.7) % | 51/744 | 4.3 (1.2-7.4) % | 7/162 | 7.6 (5.5-9.8) % | 44/582 |
|  | GCSEs / NVQ 2 | 11.3 (9-13.6) % | 84/744 | 7.4 (3.7-11.7) % | 12/162 | 12.4 (9.8-15.1) % | 72/582 |
|  | O level / NVQ 1 | 9.8 (7.8-12) % | 73/744 | 8 (4.3-12.3) % | 13/162 | 10.3 (7.9-12.9) % | 60/582 |
|  | No formal qualifications | 33.3 (30.1-36.7) % | 248/744 | 32.7 (25.9-40.1) % | 53/162 | 33.5 (29.9-37.3) % | 195/582 |
|  | Education not recorded | 14.9 (12.5-17.3) % | 111/744 | 25.9 (19.1-32.7) % | 42/162 | 11.9 (9.3-14.6) % | 69/582 |
| IMD deciles |  |  |  |  |  |  |  |
|  | 1 (most deprived) | 4.8 (3.3-6.5) % | 35/725 | 6.5 (2.6-10.4) % | 10/154 | 4.4 (2.8-6.1) % | 25/571 |
|  | 2 | 5.9 (4.3-7.7) % | 43/725 | 7.1 (3.2-11.7) % | 11/154 | 5.6 (3.9-7.7) % | 32/571 |
|  | 3 | 7.4 (5.5-9.4) % | 54/725 | 7.1 (3.2-11) % | 11/154 | 7.5 (5.6-9.8) % | 43/571 |
|  | 4 | 7.9 (5.9-9.9) % | 57/725 | 8.4 (4.5-13) % | 13/154 | 7.7 (5.6-10) % | 44/571 |
|  | 5 | 8.8 (6.8-10.9) % | 64/725 | 9.7 (5.2-14.9) % | 15/154 | 8.6 (6.3-11) % | 49/571 |
|  | 6 | 12.7 (10.3-15.2) % | 92/725 | 13.6 (8.4-19.5) % | 21/154 | 12.4 (9.8-15.2) % | 71/571 |
|  | 7 | 13 (10.6-15.6) % | 94/725 | 13.6 (8.4-19.5) % | 21/154 | 12.8 (10.2-15.6) % | 73/571 |
|  | 8 | 12.4 (9.9-14.9) % | 90/725 | 11 (6.5-16.2) % | 17/154 | 12.8 (10.2-15.6) % | 73/571 |
|  | 9 | 13.8 (11.4-16.4) % | 100/725 | 12.3 (7.1-18.2) % | 19/154 | 14.2 (11.2-17.2) % | 81/571 |
|  | 10 (least deprived) | 13.2 (10.9-15.7) % | 96/725 | 10.4 (5.8-15.6) % | 16/154 | 14 (11-17) % | 80/571 |
| Operative severity |  |  |  |  |  |  |  |
|  | Minor | 5.5 (3.9-7.3) % | 41/740 | 2.5 (0.6-4.9) % | 4/162 | 6.4 (4.7-8.5) % | 37/578 |
|  | Intermediate | 20.4 (17.6-23.1) % | 151/740 | 9.3 (4.9-13.6) % | 15/162 | 23.5 (20.2-26.8) % | 136/578 |
|  | Major | 37.4 (33.9-41.1) % | 277/740 | 41.4 (34-49.4) % | 67/162 | 36.3 (32.4-40.3) % | 210/578 |
|  | X Major | 30.1 (26.9-33.4) % | 223/740 | 40.7 (32.7-48.1) % | 66/162 | 27.2 (23.5-30.8) % | 157/578 |
|  | Complex | 6.5 (4.9-8.2) % | 48/740 | 6.2 (2.5-10.5) % | 10/162 | 6.6 (4.5-8.7) % | 38/578 |
| Preop assessment |  |  |  |  |  |  |  |
|  | Nurse or AHP led clinic | 22.6 (19.6-25.7) % | 159/705 | 9.3 (4.7-14) % | 14/150 | 26.1 (22.3-29.7) % | 145/555 |
|  | Anaesthetist led clinic | 5.1 (3.5-6.7) % | 36/705 | 2 (0-4.7) % | 3/150 | 5.9 (4.1-8.1) % | 33/555 |
|  | Anaesthetist and geriatrician led clinics | 0.1 (0-0.4) % | 1/705 | 0.7 (0-2) % | 1/150 | NA | NA |
|  | Geriatrician led or MDT clinic | 1 (0.3-1.8) % | 7/705 | NA | NA | 1.3 (0.4-2.2) % | 7/555 |
|  | Physician (non geriatrician) led clinic | 1.4 (0.6-2.4) % | 10/705 | 1.3 (0-3.3) % | 2/150 | 1.4 (0.5-2.5) % | 8/555 |
|  | On the day assessment | 69.8 (66.5-73) % | 492/705 | 86.7 (81.3-92) % | 130/150 | 65.2 (61.4-69) % | 362/555 |
| Postop destination |  |  |  |  |  |  |  |
|  | Ward (level 0 or 1 care) | 87.5 (84.9-89.8) % | 651/744 | 85.3 (79.8-90.8) % | 139/163 | 88.1 (85.2-90.5) % | 512/581 |
|  | Unplanned admission to PACU or equivalent (level 1.5 care) | 0.8 (0.3-1.5) % | 6/744 | 1.2 (0-3.1) % | 2/163 | 0.7 (0.2-1.4) % | 4/581 |
|  | Planned admission to PACU or equivalent (level 1.5 care) | 5.6 (4-7.4) % | 42/744 | 3.1 (0.6-6.1) % | 5/163 | 6.4 (4.5-8.4) % | 37/581 |
|  | Unplanned admission to PACU or equivalent (level 2/3 care) | 0.4 (0-0.9) % | 3/744 | 0.6 (0-1.8) % | 1/163 | 0.3 (0-0.9) % | 2/581 |
|  | Planned admission to PACU or equivalent (level 2/3 care) | 1.2 (0.4-2.2) % | 9/744 | 1.2 (0-3.1) % | 2/163 | 1.2 (0.3-2.2) % | 7/581 |
|  | Unplanned critical care admission (level 2 or 3 care) | 0.9 (0.3-1.7) % | 7/744 | 3.1 (0.6-6.1) % | 5/163 | 0.3 (0-0.9) % | 2/581 |
|  | Planned critical care admission (level 2 or 3 care) | 3.2 (2-4.6) % | 24/744 | 5.5 (2.5-9.2) % | 9/163 | 2.6 (1.4-4) % | 15/581 |
| Urinary catheter |  |  |  |  |  |  |  |
|  | No catheter | 49.2 (45.7-52.8) % | 366/744 | 41.7 (33.7-49.1) % | 68/163 | 51.3 (47-55.2) % | 298/581 |
|  | Long-term/pre-admission catheter | 4.3 (2.8-5.8) % | 32/744 | 5.5 (2.5-9.2) % | 9/163 | 4 (2.4-5.7) % | 23/581 |
|  | Electively catheterised pre/intra-op | 41.4 (37.8-44.9) % | 308/744 | 47.9 (39.9-55.8) % | 78/163 | 39.6 (35.5-43.7) % | 230/581 |
|  | Catheterised post-op | 2.7 (1.6-3.9) % | 20/744 | 3.1 (0.6-6.1) % | 5/163 | 2.6 (1.4-4.1) % | 15/581 |

#### Supplementary table 5B: Demographics of participants aged <85 in the SNAP-3 cohort, with and without delirium

|  |  | **All** | | **Delirium** | | **No delirium** | |
| --- | --- | --- | --- | --- | --- | --- | --- |
| **Characteristic** |  | **Summary** | **Number** | **Summary** | **Number** | **Summary** | **Number** |
| Sex |  |  |  |  |  |  |  |
|  | Female | 48.6 (47.4-49.8) % | 3063/6305 | 46 (40.6-51.4) % | 145/315 | 48.7 (47.4-50) % | 2918/5990 |
|  | Male | 51.4 (50.2-52.7) % | 3242/6305 | 54 (48.3-60) % | 170/315 | 51.3 (50-52.5) % | 3072/5990 |
| Age |  |  |  |  |  |  |  |
|  | Age (years) | 71 (6.6) | 6306 | 73.8 (6.5) | 315 | 70.9 (6.3) | 5991 |
|  | 60-69 | 41 (39.8-42.2) % | 2585/6306 | 27 (21.9-32.1) % | 85/315 | 41.7 (40.4-43) % | 2500/5991 |
|  | 70-79 | 45 (43.8-46.2) % | 2836/6306 | 45.7 (40-51.4) % | 144/315 | 44.9 (43.7-46.2) % | 2692/5991 |
|  | 80-89 | 14 (13.2-14.9) % | 885/6306 | 27.3 (22.5-32.4) % | 86/315 | 13.3 (12.5-14.2) % | 799/5991 |
| ASA |  |  |  |  |  |  |  |
|  | 1 | 8.1 (7.4-8.8) % | 506/6252 | 1.3 (0.3-2.5) % | 4/314 | 8.5 (7.8-9.2) % | 502/5938 |
|  | 2 | 55.8 (54.5-57) % | 3490/6252 | 26.8 (22-31.5) % | 84/314 | 57.4 (56.1-58.6) % | 3406/5938 |
|  | 3 | 33.1 (31.9-34.3) % | 2068/6252 | 58.3 (52.5-64) % | 183/314 | 31.7 (30.5-33) % | 1885/5938 |
|  | 4 | 2.9 (2.5-3.3) % | 181/6252 | 13.4 (9.9-17.2) % | 42/314 | 2.3 (2-2.7) % | 139/5938 |
|  | 5 | 0.1 (0-0.2) % | 7/6252 | 0.3 (0-1) % | 1/314 | 0.1 (0-0.2) % | 6/5938 |
| Frailty (CFS≥5) | Frail | 16 (15.1-16.9) % | 1000/6249 | 48.9 (43.5-54.3) % | 153/313 | 14.3 (13.4-15.2) % | 847/5936 |
| Clinical Frailty Scale |  |  |  |  |  |  |  |
|  | 1 | 12.7 (11.9-13.5) % | 792/6249 | 4.5 (2.2-7) % | 14/313 | 13.1 (12.3-14) % | 778/5936 |
|  | 2 | 20.8 (19.8-21.8) % | 1298/6249 | 9.6 (6.4-13.1) % | 30/313 | 21.4 (20.3-22.4) % | 1268/5936 |
|  | 3 | 32.2 (31-33.3) % | 2012/6249 | 19.2 (15-23.6) % | 60/313 | 32.9 (31.7-34.1) % | 1952/5936 |
|  | 4 | 18.4 (17.4-19.3) % | 1147/6249 | 17.9 (13.7-22.4) % | 56/313 | 18.4 (17.4-19.3) % | 1091/5936 |
|  | 5 | 8.5 (7.8-9.2) % | 530/6249 | 18.2 (14.1-22.4) % | 57/313 | 8 (7.3-8.6) % | 473/5936 |
|  | 6 | 5 (4.5-5.6) % | 314/6249 | 15 (11.2-19.2) % | 47/313 | 4.5 (4-5) % | 267/5936 |
|  | 7 | 2.3 (1.9-2.6) % | 142/6249 | 13.7 (9.9-17.6) % | 43/313 | 1.7 (1.3-2) % | 99/5936 |
|  | 8 | 0.2 (0.1-0.3) % | 14/6249 | 1.9 (0.6-3.5) % | 6/313 | 0.1 (0.1-0.2) % | 8/5936 |
| Multimorbidity (≥2 comorbidities) | Multimorbid | 60.9 (59.6-62.2) % | 3413/5601 | 76.5 (71.5-81.1) % | 231/302 | 60 (58.7-61.4) % | 3182/5299 |
| Number of comorbidities | Number of comorbidities | 2 (1-3) | 5601 | 3 (2-4) | 302 | 2 (1-3) | 5299 |
| Dementia | NA | 1.3 (1-1.6) % | 83/6306 | 12.7 (9.2-16.5) % | 40/315 | 0.7 (0.5-1) % | 43/5991 |
| Consent |  |  |  |  |  |  |  |
|  | Capacity | 98.1 (97.8-98.5) % | 6189/6306 | 80.3 (76.2-84.8) % | 253/315 | 99.1 (98.8-99.3) % | 5936/5991 |
|  | Consultee | 1.9 (1.5-2.2) % | 117/6306 | 19.7 (15.2-24.1) % | 62/315 | 0.9 (0.7-1.2) % | 55/5991 |
| Surgical specialty |  |  |  |  |  |  |  |
|  | Bariatric | 0.2 (0.1-0.3) % | 10/6214 | NA | NA | 0.2 (0.1-0.3) % | 10/5901 |
|  | Breast | 5.9 (5.3-6.5) % | 365/6214 | 0.3 (0-1) % | 1/313 | 6.2 (5.6-6.8) % | 364/5901 |
|  | Cardiology | 0 (0-0.1) % | 2/6214 | NA | NA | 0 (0-0.1) % | 2/5901 |
|  | Colorectal | 12.3 (11.5-13.2) % | 767/6214 | 11.2 (7.7-14.7) % | 35/313 | 12.4 (11.5-13.2) % | 732/5901 |
|  | Ear, nose & throat | 4 (3.5-4.5) % | 250/6214 | 0.6 (0-1.6) % | 2/313 | 4.2 (3.7-4.7) % | 248/5901 |
|  | Endocrine | 1 (0.7-1.2) % | 61/6214 | 0.3 (0-1) % | 1/313 | 1 (0.8-1.3) % | 60/5901 |
|  | Endoscopic | 0.4 (0.2-0.5) % | 24/6214 | NA | NA | 0.4 (0.3-0.6) % | 24/5901 |
|  | Gynaecology | 5.2 (4.6-5.7) % | 321/6214 | 1.3 (0.3-2.6) % | 4/313 | 5.4 (4.8-6) % | 317/5901 |
|  | Hepato-pancreato-biliary | 0.7 (0.5-0.9) % | 41/6214 | 2.6 (1-4.5) % | 8/313 | 0.6 (0.4-0.8) % | 33/5901 |
|  | Interventional radiology | 1.1 (0.9-1.4) % | 71/6214 | 2.6 (1-4.5) % | 8/313 | 1.1 (0.8-1.3) % | 63/5901 |
|  | Maxillo-facial and dental | 1.4 (1.1-1.6) % | 84/6214 | 1.3 (0.3-2.6) % | 4/313 | 1.4 (1.1-1.7) % | 80/5901 |
|  | Neurosurgery | 1 (0.8-1.3) % | 62/6214 | 3.5 (1.6-5.8) % | 11/313 | 0.9 (0.6-1.1) % | 51/5901 |
|  | Ophthalmic | 1.3 (1-1.6) % | 79/6214 | NA | NA | 1.3 (1.1-1.7) % | 79/5901 |
|  | Orthopaedics | 27.5 (26.5-28.6) % | 1711/6214 | 45 (39.9-50.8) % | 141/313 | 26.6 (25.5-27.7) % | 1570/5901 |
|  | Plastics | 3.7 (3.2-4.2) % | 228/6214 | 1.9 (0.6-3.5) % | 6/313 | 3.8 (3.3-4.3) % | 222/5901 |
|  | Spine | 2.2 (1.9-2.6) % | 138/6214 | 3.8 (1.9-6.1) % | 12/313 | 2.1 (1.8-2.5) % | 126/5901 |
|  | Thoracic | 4.4 (3.9-4.9) % | 272/6214 | 11.5 (8-15) % | 36/313 | 4 (3.5-4.5) % | 236/5901 |
|  | Transplant | 0 (0-0.1) % | 3/6214 | 0.3 (0-1) % | 1/313 | 0 (0-0.1) % | 2/5901 |
|  | Upper gastrointestinal | 5.7 (5.1-6.3) % | 355/6214 | 4.8 (2.6-7.3) % | 15/313 | 5.8 (5.2-6.4) % | 340/5901 |
|  | Urology | 19.2 (18.2-20.2) % | 1190/6214 | 6.1 (3.5-8.9) % | 19/313 | 19.8 (18.8-20.8) % | 1171/5901 |
|  | Vascular | 2.8 (2.4-3.2) % | 174/6214 | 2.9 (1.3-4.8) % | 9/313 | 2.8 (2.4-3.2) % | 165/5901 |
|  | Other | 0 (0-0.1) % | 2/6214 | NA | NA | 0 (0-0.1) % | 2/5901 |
|  | Unknown | 0.1 (0-0.1) % | 4/6214 | NA | NA | 0.1 (0-0.1) % | 4/5901 |
| Surgical urgency |  |  |  |  |  |  |  |
|  | Emergency | 2 (1.6-2.3) % | 124/6305 | 4.8 (2.5-7.3) % | 15/315 | 1.8 (1.5-2.2) % | 109/5990 |
|  | Urgent | 12.6 (11.8-13.4) % | 794/6305 | 36.5 (31.1-41.9) % | 115/315 | 11.3 (10.5-12.2) % | 679/5990 |
|  | Expedited | 12.8 (11.9-13.6) % | 804/6305 | 23.2 (18.4-27.9) % | 73/315 | 12.2 (11.4-13.1) % | 731/5990 |
|  | Planned | 72.7 (71.6-73.8) % | 4583/6305 | 35.6 (30.5-41) % | 112/315 | 74.6 (73.5-75.7) % | 4471/5990 |
| Source of admission |  |  |  |  |  |  |  |
|  | Own home | 96.2 (95.7-96.7) % | 6062/6301 | 88.3 (84.4-91.4) % | 278/315 | 96.6 (96.2-97.1) % | 5784/5986 |
|  | Sheltered housing | 1.2 (0.9-1.5) % | 75/6301 | 1.6 (0.3-3.2) % | 5/315 | 1.2 (0.9-1.4) % | 70/5986 |
|  | Residential or nursing home | 0.8 (0.6-1) % | 50/6301 | 7.3 (4.4-10.2) % | 23/315 | 0.5 (0.3-0.6) % | 27/5986 |
|  | Rehabilitation facility | 0.1 (0-0.1) % | 4/6301 | NA | NA | 0.1 (0-0.1) % | 4/5986 |
|  | Another secondary care hospital | 0.6 (0.4-0.9) % | 40/6301 | 2.5 (1-4.4) % | 8/315 | 0.5 (0.4-0.7) % | 32/5986 |
|  | Other | 0.8 (0.6-1) % | 49/6301 | 0.3 (0-1) % | 1/315 | 0.8 (0.6-1) % | 48/5986 |
|  | Unknown | 0.3 (0.2-0.5) % | 21/6301 | NA | NA | 0.4 (0.2-0.5) % | 21/5986 |
| Independence |  |  |  |  |  |  |  |
|  | Independent | 76.4 (75.3-77.4) % | 4696/6150 | 48.3 (42.5-53.8) % | 141/292 | 77.8 (76.7-78.9) % | 4555/5858 |
|  | Assistance with instrumental ADLs | 20.9 (19.9-22) % | 1288/6150 | 39.7 (33.9-45.5) % | 116/292 | 20 (19-21) % | 1172/5858 |
|  | Assistance with basic ADLs | 2.7 (2.3-3.1) % | 166/6150 | 12 (8.2-15.8) % | 35/292 | 2.2 (1.9-2.6) % | 131/5858 |
| Ethnicity |  |  |  |  |  |  |  |
|  | Asian | 2.2 (1.8-2.6) % | 136/6225 | 2.6 (1-4.5) % | 8/308 | 2.2 (1.8-2.5) % | 128/5917 |
|  | Black | 1.5 (1.2-1.8) % | 94/6225 | 1.9 (0.6-3.6) % | 6/308 | 1.5 (1.2-1.8) % | 88/5917 |
|  | Mixed | 0.6 (0.4-0.8) % | 35/6225 | 0.3 (0-1) % | 1/308 | 0.6 (0.4-0.8) % | 34/5917 |
|  | White | 95.7 (95.2-96.2) % | 5960/6225 | 95.1 (92.5-97.4) % | 293/308 | 95.8 (95.3-96.3) % | 5667/5917 |
| Education |  |  |  |  |  |  |  |
|  | Degree level | 23.7 (22.7-24.8) % | 1493/6300 | 17.8 (13.7-22.2) % | 56/315 | 24 (23-25) % | 1437/5985 |
|  | A levels / NVQ 3 | 12.4 (11.6-13.2) % | 783/6300 | 9.5 (6.3-13) % | 30/315 | 12.6 (11.8-13.4) % | 753/5985 |
|  | Apprenticeship | 6.6 (6-7.3) % | 416/6300 | 7.3 (4.4-10.5) % | 23/315 | 6.6 (5.9-7.2) % | 393/5985 |
|  | GCSEs / NVQ 2 | 14.6 (13.7-15.5) % | 918/6300 | 13 (9.2-17.1) % | 41/315 | 14.7 (13.8-15.6) % | 877/5985 |
|  | O level / NVQ 1 | 11.9 (11.1-12.7) % | 751/6300 | 11.7 (8.3-15.2) % | 37/315 | 11.9 (11.1-12.8) % | 714/5985 |
|  | No formal qualifications | 21.4 (20.4-22.4) % | 1350/6300 | 24.1 (19.4-28.9) % | 76/315 | 21.3 (20.3-22.3) % | 1274/5985 |
|  | Education not recorded | 9.3 (8.6-10.1) % | 589/6300 | 16.5 (12.4-20.6) % | 52/315 | 9 (8.2-9.7) % | 537/5985 |
| IMD deciles |  |  |  |  |  |  |  |
|  | 1 (most deprived) | 7 (6.4-7.7) % | 433/6148 | 9.5 (6.6-12.8) % | 29/304 | 6.9 (6.3-7.6) % | 404/5844 |
|  | 2 | 7.8 (7.1-8.4) % | 477/6148 | 7.2 (4.6-10.2) % | 22/304 | 7.8 (7.1-8.5) % | 455/5844 |
|  | 3 | 8.3 (7.6-9) % | 512/6148 | 8.2 (5.3-11.2) % | 25/304 | 8.3 (7.6-9) % | 487/5844 |
|  | 4 | 9 (8.3-9.7) % | 554/6148 | 9.9 (6.6-13.2) % | 30/304 | 9 (8.2-9.7) % | 524/5844 |
|  | 5 | 10.1 (9.4-10.8) % | 620/6148 | 8.6 (5.6-11.8) % | 26/304 | 10.2 (9.4-11) % | 594/5844 |
|  | 6 | 12 (11.2-12.8) % | 737/6148 | 12.8 (9.2-16.8) % | 39/304 | 11.9 (11.1-12.8) % | 698/5844 |
|  | 7 | 11.7 (10.9-12.4) % | 717/6148 | 11.2 (7.9-14.8) % | 34/304 | 11.7 (10.8-12.5) % | 683/5844 |
|  | 8 | 11.3 (10.5-12.1) % | 693/6148 | 12.8 (9.2-17.1) % | 39/304 | 11.2 (10.4-12) % | 654/5844 |
|  | 9 | 12.3 (11.5-13.1) % | 754/6148 | 11.5 (8.2-15.1) % | 35/304 | 12.3 (11.5-13.2) % | 719/5844 |
|  | 10 (least deprived) | 10.6 (9.9-11.4) % | 651/6148 | 8.2 (5.3-11.2) % | 25/304 | 10.7 (9.9-11.5) % | 626/5844 |
| Operative severity |  |  |  |  |  |  |  |
|  | Minor | 6.3 (5.7-6.9) % | 391/6213 | 1.9 (0.6-3.5) % | 6/313 | 6.5 (5.9-7.2) % | 385/5900 |
|  | Intermediate | 26.3 (25.2-27.4) % | 1633/6213 | 8.6 (5.8-11.8) % | 27/313 | 27.2 (26.1-28.4) % | 1606/5900 |
|  | Major | 30 (28.9-31.2) % | 1865/6213 | 31 (26.2-36.1) % | 97/313 | 30 (28.8-31.1) % | 1768/5900 |
|  | X Major | 24.4 (23.3-25.5) % | 1514/6213 | 32.3 (27.2-37.4) % | 101/313 | 23.9 (22.9-25.1) % | 1413/5900 |
|  | Complex | 13 (12.1-13.8) % | 806/6213 | 26.2 (21.1-31.3) % | 82/313 | 12.3 (11.5-13.1) % | 724/5900 |
|  | Unknown | 0.1 (0-0.1) % | 4/6213 | NA | NA | 0.1 (0-0.1) % | 4/5900 |
| Preop assessment |  |  |  |  |  |  |  |
|  | Nurse or AHP led clinic | 36.8 (35.7-38.1) % | 2239/6079 | 18.7 (14.3-23.1) % | 55/294 | 37.8 (36.5-39) % | 2184/5785 |
|  | Anaesthetist led clinic | 7.5 (6.8-8.1) % | 454/6079 | 6.8 (4.1-9.9) % | 20/294 | 7.5 (6.8-8.2) % | 434/5785 |
|  | Anaesthetist and geriatrician led clinics | 0.2 (0.1-0.3) % | 13/6079 | NA | NA | 0.2 (0.1-0.3) % | 13/5785 |
|  | Geriatrician led or MDT clinic | 0.9 (0.7-1.2) % | 57/6079 | 1.7 (0.3-3.4) % | 5/294 | 0.9 (0.7-1.1) % | 52/5785 |
|  | Physician (non geriatrician) led clinic | 1.1 (0.8-1.3) % | 66/6079 | 0.7 (0-1.7) % | 2/294 | 1.1 (0.9-1.4) % | 64/5785 |
|  | On the day assessment | 53.5 (52.2-54.7) % | 3250/6079 | 72.1 (67-77.2) % | 212/294 | 52.5 (51.3-53.8) % | 3038/5785 |
| Postop destination |  |  |  |  |  |  |  |
|  | Ward (level 0 or 1 care) | 85.8 (84.9-86.6) % | 5398/6294 | 67.2 (62.1-72.3) % | 211/314 | 86.7 (85.9-87.6) % | 5187/5980 |
|  | Unplanned admission to PACU or equivalent (level 1.5 care) | 0.5 (0.3-0.6) % | 29/6294 | 1 (0-2.2) % | 3/314 | 0.4 (0.3-0.6) % | 26/5980 |
|  | Planned admission to PACU or equivalent (level 1.5 care) | 6 (5.4-6.6) % | 376/6294 | 6.7 (4.1-9.6) % | 21/314 | 5.9 (5.4-6.5) % | 355/5980 |
|  | Unplanned admission to PACU or equivalent (level 2/3 care) | 0.2 (0.1-0.3) % | 10/6294 | 1.3 (0.3-2.5) % | 4/314 | 0.1 (0-0.2) % | 6/5980 |
|  | Planned admission to PACU or equivalent (level 2/3 care) | 1.5 (1.2-1.8) % | 92/6294 | 3.5 (1.6-5.7) % | 11/314 | 1.4 (1.1-1.7) % | 81/5980 |
|  | Unplanned critical care admission (level 2 or 3 care) | 0.7 (0.5-0.9) % | 44/6294 | 3.5 (1.6-5.7) % | 11/314 | 0.6 (0.4-0.8) % | 33/5980 |
|  | Planned critical care admission (level 2 or 3 care) | 5.3 (4.7-5.8) % | 331/6294 | 16.9 (13.1-21) % | 53/314 | 4.6 (4.1-5.2) % | 278/5980 |
| Urinary catheter |  |  |  |  |  |  |  |
|  | No catheter | 64.7 (63.5-65.9) % | 4075/6295 | 33.3 (27.9-38.7) % | 105/315 | 66.4 (65.2-67.6) % | 3970/5980 |
|  | Long-term/pre-admission catheter | 1.8 (1.5-2.2) % | 116/6295 | 5.7 (3.2-8.6) % | 18/315 | 1.6 (1.3-2) % | 98/5980 |
|  | Electively catheterised pre/intra-op | 29.6 (28.4-30.6) % | 1861/6295 | 55.6 (50.2-61) % | 175/315 | 28.2 (27.1-29.3) % | 1686/5980 |
|  | Catheterised post-op | 1.9 (1.6-2.3) % | 120/6295 | 4.1 (1.9-6.3) % | 13/315 | 1.8 (1.5-2.1) % | 107/5980 |

#### Supplementary table 5C: Demographics of participants living with frailty in the SNAP-3 cohort, with and without delirium

|  |  | **All** | | **Delirium** | | **No delirium** | |
| --- | --- | --- | --- | --- | --- | --- | --- |
| **Characteristic** |  | **Summary** | **Number** | **Summary** | **Number** | **Summary** | **Number** |
| Sex |  |  |  |  |  |  |  |
|  | Female | 57.8 (55.3-60.5) % | 789/1364 | 62.6 (56.5-68.3) % | 174/278 | 56.6 (53.7-59.7) % | 615/1086 |
|  | Male | 42.2 (39.6-44.8) % | 575/1364 | 37.4 (31.7-43.2) % | 104/278 | 43.4 (40.5-46.2) % | 471/1086 |
| Age |  |  |  |  |  |  |  |
|  | Age (years) | 77 (9.6) | 1363 | 81.8 (9.5) | 277 | 75.8 (9.3) | 1086 |
|  | 60-69 | 25.2 (23-27.6) % | 344/1363 | 12.6 (8.7-17) % | 35/277 | 28.5 (25.8-31) % | 309/1086 |
|  | 70-79 | 30.6 (28.1-33.1) % | 417/1363 | 20.9 (15.9-25.6) % | 58/277 | 33.1 (30.3-35.8) % | 359/1086 |
|  | 80-89 | 32.8 (30.4-35.2) % | 447/1363 | 41.5 (36.1-47.3) % | 115/277 | 30.6 (27.8-33.3) % | 332/1086 |
|  | >90 | 11.4 (9.8-13) % | 155/1363 | 24.9 (19.9-30.3) % | 69/277 | 7.9 (6.3-9.6) % | 86/1086 |
| ASA |  |  |  |  |  |  |  |
|  | 1 | 0.5 (0.1-1) % | 7/1354 | NA | NA | 0.7 (0.2-1.1) % | 7/1076 |
|  | 2 | 20.2 (17.9-22.4) % | 274/1354 | 8.6 (5.4-12.2) % | 24/278 | 23.2 (20.7-25.8) % | 250/1076 |
|  | 3 | 65.9 (63.4-68.3) % | 892/1354 | 65.8 (60.4-71.2) % | 183/278 | 65.9 (63.2-68.8) % | 709/1076 |
|  | 4 | 13.4 (11.6-15.2) % | 181/1354 | 25.5 (20.9-30.9) % | 71/278 | 10.2 (8.6-12.1) % | 110/1076 |
| Frailty (CFS≥5) | Frail | 100 (NA-NA) % | 1364/1364 | 100 (NA-NA) % | 278/278 | 100 (NA-NA) % | 1086/1086 |
| Clinical Frailty Scale |  |  |  |  |  |  |  |
|  | 5 | 51 (48.2-53.9) % | 696/1364 | 36 (30.6-41.7) % | 100/278 | 54.9 (51.9-57.8) % | 596/1086 |
|  | 6 | 30.9 (28.2-33.3) % | 421/1364 | 30.2 (24.8-35.6) % | 84/278 | 31 (28.4-33.8) % | 337/1086 |
|  | 7 | 16.3 (14.3-18.3) % | 222/1364 | 29.1 (23.7-34.9) % | 81/278 | 13 (11-15) % | 141/1086 |
|  | 8 | 1.8 (1.1-2.6) % | 25/1364 | 4.7 (2.5-7.2) % | 13/278 | 1.1 (0.6-1.7) % | 12/1086 |
| Multimorbidity (≥2 comorbidities) | Multimorbid | 85.9 (83.9-87.9) % | 1075/1251 | 88 (83.9-91.8) % | 235/267 | 85.4 (83.2-87.5) % | 840/984 |
| Number of comorbidities | Number of comorbidities | 3 (2-4) | 1251 | 3 (2-4) | 267 | 3 (2-4) | 984 |
| Dementia |  | 12.2 (10.6-14) % | 166/1364 | 36 (30.6-41.7) % | 100/278 | 6.1 (4.8-7.6) % | 66/1086 |
| Consent |  |  |  |  |  |  |  |
|  | Capacity | 85 (83-86.9) % | 1159/1364 | 55.8 (50-61.2) % | 155/278 | 92.4 (90.9-94) % | 1004/1086 |
|  | Consultee | 15 (13.1-16.9) % | 205/1364 | 44.2 (38.1-50) % | 123/278 | 7.6 (6-9.1) % | 82/1086 |
| Surgical specialty |  |  |  |  |  |  |  |
|  | Bariatric | 0.1 (0-0.2) % | 1/1349 | NA | NA | 0.1 (0-0.3) % | 1/1071 |
|  | Breast | 2.2 (1.5-3) % | 30/1349 | NA | NA | 2.8 (1.9-3.8) % | 30/1071 |
|  | Colorectal | 8.2 (6.7-9.7) % | 111/1349 | 6.1 (3.6-9) % | 17/278 | 8.8 (7.1-10.5) % | 94/1071 |
|  | Ear, nose & throat | 1.8 (1.1-2.5) % | 24/1349 | 0.4 (0-1.1) % | 1/278 | 2.1 (1.3-3.1) % | 23/1071 |
|  | Endocrine | 0.4 (0.1-0.7) % | 5/1349 | NA | NA | 0.5 (0.1-0.9) % | 5/1071 |
|  | Endoscopic | 0.6 (0.2-1) % | 8/1349 | 0.4 (0-1.1) % | 1/278 | 0.7 (0.2-1.1) % | 7/1071 |
|  | Gynaecology | 3.4 (2.4-4.4) % | 46/1349 | NA | NA | 4.3 (3.1-5.5) % | 46/1071 |
|  | Hepato-pancreato-biliary | 0.1 (0-0.4) % | 2/1349 | 0.7 (0-1.8) % | 2/278 | NA | NA |
|  | Interventional radiology | 1.6 (1-2.3) % | 22/1349 | 3.2 (1.1-5.4) % | 9/278 | 1.2 (0.7-1.9) % | 13/1071 |
|  | Maxillo-facial and dental | 0.8 (0.4-1.3) % | 11/1349 | 0.4 (0-1.1) % | 1/278 | 0.9 (0.4-1.6) % | 10/1071 |
|  | Neurosurgery | 1.3 (0.7-2) % | 18/1349 | 2.2 (0.7-4) % | 6/278 | 1.1 (0.6-1.8) % | 12/1071 |
|  | Ophthalmic | 1 (0.5-1.6) % | 14/1349 | NA | NA | 1.3 (0.7-2.1) % | 14/1071 |
|  | Orthopaedics | 46.6 (43.8-49.1) % | 628/1349 | 71.2 (65.8-76.6) % | 198/278 | 40.1 (37.2-43.1) % | 430/1071 |
|  | Plastics | 4.9 (3.8-6.1) % | 66/1349 | 2.2 (0.7-4) % | 6/278 | 5.6 (4.2-7) % | 60/1071 |
|  | Spine | 3.3 (2.4-4.4) % | 45/1349 | 2.2 (0.7-4) % | 6/278 | 3.6 (2.5-4.9) % | 39/1071 |
|  | Thoracic | 2 (1.3-2.7) % | 27/1349 | 1.8 (0.4-3.6) % | 5/278 | 2.1 (1.2-2.9) % | 22/1071 |
|  | Transplant | 0.1 (0-0.2) % | 1/1349 | NA | NA | 0.1 (0-0.3) % | 1/1071 |
|  | Upper gastrointestinal | 2.6 (1.8-3.4) % | 35/1349 | 3.2 (1.4-5.4) % | 9/278 | 2.4 (1.6-3.4) % | 26/1071 |
|  | Urology | 14.9 (13-16.8) % | 201/1349 | 4.7 (2.5-7.2) % | 13/278 | 17.6 (15.4-19.8) % | 188/1071 |
|  | Vascular | 4 (3-5) % | 54/1349 | 1.4 (0.4-2.9) % | 4/278 | 4.7 (3.5-6) % | 50/1071 |
| Surgical urgency |  |  |  |  |  |  |  |
|  | Emergency | 3.4 (2.4-4.4) % | 46/1364 | 5.4 (2.9-8.3) % | 15/278 | 2.9 (1.8-4) % | 31/1086 |
|  | Urgent | 29.5 (27.1-32) % | 402/1364 | 56.1 (50-61.9) % | 156/278 | 22.7 (20.1-25.1) % | 246/1086 |
|  | Expedited | 15.8 (13.9-17.7) % | 216/1364 | 19.4 (14.7-24.1) % | 54/278 | 14.9 (12.8-17) % | 162/1086 |
|  | Planned | 51.3 (48.5-54.1) % | 700/1364 | 19.1 (14.7-23.7) % | 53/278 | 59.6 (56.6-62.5) % | 647/1086 |
| Source of admission |  |  |  |  |  |  |  |
|  | Own home | 86.9 (85.1-88.6) % | 1185/1364 | 73.4 (68-78.8) % | 204/278 | 90.3 (88.7-92) % | 981/1086 |
|  | Sheltered housing | 3.7 (2.8-4.8) % | 51/1364 | 1.8 (0.4-3.6) % | 5/278 | 4.2 (3-5.5) % | 46/1086 |
|  | Residential or nursing home | 7 (5.6-8.4) % | 95/1364 | 20.9 (16.2-26.3) % | 58/278 | 3.4 (2.4-4.5) % | 37/1086 |
|  | Rehabilitation facility | 0.5 (0.1-0.9) % | 7/1364 | 1.1 (0-2.5) % | 3/278 | 0.4 (0.1-0.7) % | 4/1086 |
|  | Another secondary care hospital | 0.5 (0.1-0.9) % | 7/1364 | 1.1 (0-2.5) % | 3/278 | 0.4 (0.1-0.7) % | 4/1086 |
|  | Other | 1.2 (0.6-1.8) % | 16/1364 | 1.8 (0.4-3.6) % | 5/278 | 1 (0.5-1.7) % | 11/1086 |
|  | Unknown | 0.2 (0-0.5) % | 3/1364 | NA | NA | 0.3 (0-0.6) % | 3/1086 |
| Independence |  |  |  |  |  |  |  |
|  | Independent | 10.6 (9-12.4) % | 135/1272 | 8.6 (5.2-12.5) % | 20/232 | 11.1 (9.3-13) % | 115/1040 |
|  | Assistance with instrumental ADLs | 74.1 (71.6-76.3) % | 942/1272 | 66.4 (60.3-72.4) % | 154/232 | 75.8 (73-78.4) % | 788/1040 |
|  | Assistance with basic ADLs | 15.3 (13.4-17.3) % | 195/1272 | 25 (19.4-30.6) % | 58/232 | 13.2 (11.2-15.3) % | 137/1040 |
| Ethnicity |  |  |  |  |  |  |  |
|  | Asian | 2.4 (1.6-3.2) % | 32/1350 | 1.8 (0.4-3.6) % | 5/276 | 2.5 (1.6-3.4) % | 27/1074 |
|  | Black | 1.9 (1.2-2.7) % | 26/1350 | 2.2 (0.7-4) % | 6/276 | 1.9 (1.1-2.7) % | 20/1074 |
|  | Mixed | 0.7 (0.3-1.3) % | 10/1350 | 0.4 (0-1.1) % | 1/276 | 0.8 (0.4-1.4) % | 9/1074 |
|  | White | 95 (93.8-96.1) % | 1282/1350 | 95.7 (93.1-97.8) % | 264/276 | 94.8 (93.4-96.1) % | 1018/1074 |
| Education |  |  |  |  |  |  |  |
|  | Degree level | 13.4 (11.6-15.1) % | 182/1363 | 10.8 (7.2-14.4) % | 30/277 | 14 (12-16.2) % | 152/1086 |
|  | A levels / NVQ 3 | 10.1 (8.5-11.7) % | 138/1363 | 9.7 (6.5-13.4) % | 27/277 | 10.2 (8.5-12.1) % | 111/1086 |
|  | Apprenticeship | 6.3 (5.1-7.6) % | 86/1363 | 6.1 (3.6-9) % | 17/277 | 6.4 (5.1-7.8) % | 69/1086 |
|  | GCSEs / NVQ 2 | 11.1 (9.4-12.8) % | 151/1363 | 6.5 (4-9.4) % | 18/277 | 12.2 (10.4-14.3) % | 133/1086 |
|  | O level / NVQ 1 | 12 (10.3-13.8) % | 164/1363 | 9 (5.8-12.6) % | 25/277 | 12.8 (10.9-14.8) % | 139/1086 |
|  | No formal qualifications | 33 (30.6-35.6) % | 450/1363 | 33.2 (27.8-39) % | 92/277 | 33 (30.2-35.8) % | 358/1086 |
|  | Education not recorded | 14.1 (12.3-16) % | 192/1363 | 24.5 (19.5-29.6) % | 68/277 | 11.4 (9.6-13.4) % | 124/1086 |
| IMD deciles |  |  |  |  |  |  |  |
|  | 1 (most deprived) | 10.8 (9.2-12.6) % | 144/1329 | 8.7 (5.7-12.2) % | 23/263 | 11.4 (9.5-13.3) % | 121/1066 |
|  | 2 | 10.9 (9.2-12.5) % | 145/1329 | 8 (4.9-11.4) % | 21/263 | 11.6 (9.7-13.6) % | 124/1066 |
|  | 3 | 10.8 (9.1-12.4) % | 143/1329 | 8.7 (5.3-12.2) % | 23/263 | 11.3 (9.5-13.2) % | 120/1066 |
|  | 4 | 9.6 (8.1-11.1) % | 127/1329 | 10.6 (6.8-14.4) % | 28/263 | 9.3 (7.5-11) % | 99/1066 |
|  | 5 | 9.1 (7.6-10.7) % | 121/1329 | 9.9 (6.1-13.7) % | 26/263 | 8.9 (7.2-10.7) % | 95/1066 |
|  | 6 | 11.5 (9.9-13.2) % | 153/1329 | 11 (7.6-14.8) % | 29/263 | 11.6 (9.8-13.7) % | 124/1066 |
|  | 7 | 10.5 (8.8-12.1) % | 139/1329 | 11.8 (8-16) % | 31/263 | 10.1 (8.3-12.1) % | 108/1066 |
|  | 8 | 9.3 (7.8-10.8) % | 123/1329 | 11 (7.6-15.2) % | 29/263 | 8.8 (7.1-10.5) % | 94/1066 |
|  | 9 | 10.5 (8.9-12.2) % | 139/1329 | 12.5 (8.7-16.7) % | 33/263 | 9.9 (8.2-11.7) % | 106/1066 |
|  | 10 (least deprived) | 7.1 (5.8-8.5) % | 95/1329 | 7.6 (4.6-11) % | 20/263 | 7 (5.5-8.6) % | 75/1066 |
| Operative severity |  |  |  |  |  |  |  |
|  | Minor | 5 (3.9-6.2) % | 67/1349 | 1.1 (0-2.5) % | 3/278 | 6 (4.6-7.5) % | 64/1071 |
|  | Intermediate | 21.6 (19.5-23.8) % | 292/1349 | 10.4 (7.2-14) % | 29/278 | 24.6 (21.9-27.1) % | 263/1071 |
|  | Major | 33.4 (31-36) % | 451/1349 | 42.4 (36.7-48.2) % | 118/278 | 31.1 (28.3-33.9) % | 333/1071 |
|  | X Major | 30 (27.7-32.5) % | 405/1349 | 37.1 (31.3-42.4) % | 103/278 | 28.2 (25.6-30.8) % | 302/1071 |
|  | Complex | 9.9 (8.5-11.6) % | 134/1349 | 9 (5.8-12.2) % | 25/278 | 10.2 (8.4-12.1) % | 109/1071 |
| Preop assessment |  |  |  |  |  |  |  |
|  | Nurse or AHP led clinic | 26.2 (23.9-28.5) % | 337/1286 | 11 (7.5-15) % | 28/254 | 29.9 (27.3-32.8) % | 309/1032 |
|  | Anaesthetist led clinic | 7.5 (6.1-8.9) % | 96/1286 | 2.8 (0.8-4.7) % | 7/254 | 8.6 (7-10.4) % | 89/1032 |
|  | Anaesthetist and geriatrician led clinics | 0.3 (0.1-0.6) % | 4/1286 | 0.4 (0-1.2) % | 1/254 | 0.3 (0-0.7) % | 3/1032 |
|  | Geriatrician led or MDT clinic | 0.6 (0.2-1.1) % | 8/1286 | NA | NA | 0.8 (0.3-1.4) % | 8/1032 |
|  | Physician (non geriatrician) led clinic | 1 (0.5-1.6) % | 13/1286 | 0.8 (0-2) % | 2/254 | 1.1 (0.5-1.7) % | 11/1032 |
|  | On the day assessment | 64.4 (61.8-67) % | 828/1286 | 85 (80.3-89) % | 216/254 | 59.3 (56.2-62.3) % | 612/1032 |
| Postop destination |  |  |  |  |  |  |  |
|  | Ward (level 0 or 1 care) | 87.5 (85.7-89.3) % | 1191/1361 | 83.8 (79.4-88.1) % | 232/277 | 88.5 (86.6-90.4) % | 959/1084 |
|  | Unplanned admission to PACU or equivalent (level 1.5 care) | 0.7 (0.3-1.2) % | 10/1361 | 0.7 (0-1.8) % | 2/277 | 0.7 (0.3-1.3) % | 8/1084 |
|  | Planned admission to PACU or equivalent (level 1.5 care) | 4.6 (3.5-5.7) % | 62/1361 | 4.7 (2.5-7.2) % | 13/277 | 4.5 (3.3-5.7) % | 49/1084 |
|  | Unplanned admission to PACU or equivalent (level 2/3 care) | 0.5 (0.1-1) % | 7/1361 | 1.4 (0.4-2.9) % | 4/277 | 0.3 (0-0.6) % | 3/1084 |
|  | Planned admission to PACU or equivalent (level 2/3 care) | 1.6 (1-2.3) % | 22/1361 | 1.8 (0.4-3.6) % | 5/277 | 1.6 (0.8-2.4) % | 17/1084 |
|  | Unplanned critical care admission (level 2 or 3 care) | 1.1 (0.6-1.6) % | 15/1361 | 2.9 (1.1-5.1) % | 8/277 | 0.6 (0.3-1.1) % | 7/1084 |
|  | Planned critical care admission (level 2 or 3 care) | 3.8 (2.9-4.8) % | 52/1361 | 4.7 (2.5-7.2) % | 13/277 | 3.6 (2.6-4.7) % | 39/1084 |
| Urinary catheter |  |  |  |  |  |  |  |
|  | No catheter | 56.3 (53.7-59) % | 767/1362 | 41.7 (36-47.5) % | 116/278 | 60.1 (57.1-63) % | 651/1084 |
|  | Long-term/pre-admission catheter | 5.1 (4-6.4) % | 70/1362 | 6.5 (3.6-9.4) % | 18/278 | 4.8 (3.6-6.1) % | 52/1084 |
|  | Electively catheterised pre/intra-op | 33.9 (31.5-36.3) % | 462/1362 | 47.5 (41.4-53.2) % | 132/278 | 30.4 (27.6-33.2) % | 330/1084 |
|  | Catheterised post-op | 2.3 (1.5-3.2) % | 32/1362 | 3.2 (1.4-5.4) % | 9/278 | 2.1 (1.3-3) % | 23/1084 |

#### Supplementary table 5D: Demographics of participants not frail in the SNAP-3 cohort, with and without delirium

|  |  | **All** | | **Delirium** | | **No delirium** | |
| --- | --- | --- | --- | --- | --- | --- | --- |
| **Characteristic** |  | **Summary** | **Number** | **Summary** | **Number** | **Summary** | **Number** |
| Sex |  |  |  |  |  |  |  |
|  | Female | 46.9 (45.7-48.2) % | 2642/5628 | 38.9 (32.3-45.5) % | 77/198 | 47.2 (45.9-48.6) % | 2565/5430 |
|  | Male | 53.1 (51.8-54.3) % | 2986/5628 | 61.1 (54.5-68.2) % | 121/198 | 52.8 (51.5-54.1) % | 2865/5430 |
| Age |  |  |  |  |  |  |  |
|  | Age (years) | 71.8 (7.4) | 5624 | 75.5 (8.5) | 198 | 71.7 (7.4) | 5426 |
|  | 60-69 | 39.4 (38.1-40.6) % | 2217/5624 | 25.3 (19.2-31.3) % | 50/198 | 39.9 (38.6-41.2) % | 2167/5426 |
|  | 70-79 | 42.5 (41.3-43.8) % | 2392/5624 | 42.9 (36.4-50) % | 85/198 | 42.5 (41.2-43.8) % | 2307/5426 |
|  | 80-89 | 16.7 (15.7-17.7) % | 940/5624 | 27.3 (21.2-33.3) % | 54/198 | 16.3 (15.3-17.3) % | 886/5426 |
|  | >90 | 1.3 (1-1.6) % | 75/5624 | 4.5 (2-7.6) % | 9/198 | 1.2 (0.9-1.5) % | 66/5426 |
| ASA |  |  |  |  |  |  |  |
|  | 1 | 8.9 (8.2-9.7) % | 499/5578 | 2 (0.5-4.1) % | 4/197 | 9.2 (8.4-10) % | 495/5381 |
|  | 2 | 61.2 (60-62.5) % | 3416/5578 | 40.6 (34-47.2) % | 80/197 | 62 (60.7-63.3) % | 3336/5381 |
|  | 3 | 28.3 (27.1-29.4) % | 1577/5578 | 52.3 (45.7-59.4) % | 103/197 | 27.4 (26.2-28.5) % | 1474/5381 |
|  | 4 | 1.4 (1.1-1.7) % | 76/5578 | 4.1 (1.5-7.1) % | 8/197 | 1.3 (1-1.6) % | 68/5381 |
|  | 5 | 0.2 (0.1-0.3) % | 10/5578 | 1 (0-2.5) % | 2/197 | 0.1 (0.1-0.3) % | 8/5381 |
| Clinical Frailty Score |  |  |  |  |  |  |  |
|  | 1 | 14.4 (13.4-15.3) % | 808/5628 | 7.6 (4-11.6) % | 15/198 | 14.6 (13.7-15.5) % | 793/5430 |
|  | 2 | 24 (22.9-25.2) % | 1351/5628 | 17.7 (12.6-23.2) % | 35/198 | 24.2 (23.1-25.4) % | 1316/5430 |
|  | 3 | 38 (36.8-39.3) % | 2141/5628 | 34.8 (28.3-41.4) % | 69/198 | 38.2 (36.8-39.5) % | 2072/5430 |
|  | 4 | 23.6 (22.5-24.7) % | 1328/5628 | 39.9 (33.3-46.5) % | 79/198 | 23 (21.9-24.1) % | 1249/5430 |
| Multimorbidity (≥2 comorbidities) | Multimorbid | 57.4 (56-58.7) % | 2863/4992 | 68.9 (62.6-75.8) % | 131/190 | 56.9 (55.5-58.3) % | 2732/4802 |
| Number of comorbidities | Number of comorbidities | 2 (1-3) | 4992 | 2 (1-3) | 190 | 2 (1-3) | 4802 |
| Dementia |  | 0.3 (0.2-0.4) % | 17/5628 | 0.5 (0-1.5) % | 1/198 | 0.3 (0.1-0.5) % | 16/5430 |
| Consent |  |  |  |  |  |  |  |
|  | Capacity | 99.5 (99.3-99.7) % | 5600/5628 | 91.9 (87.9-95.5) % | 182/198 | 99.8 (99.7-99.9) % | 5418/5430 |
|  | Consultee | 0.5 (0.3-0.7) % | 28/5628 | 8.1 (4.5-12.1) % | 16/198 | 0.2 (0.1-0.3) % | 12/5430 |
| Surgical specialty |  |  |  |  |  |  |  |
|  | Bariatric | 0.2 (0.1-0.3) % | 9/5547 | NA | NA | 0.2 (0.1-0.3) % | 9/5352 |
|  | Breast | 6.4 (5.7-7) % | 353/5547 | 0.5 (0-1.5) % | 1/195 | 6.6 (5.9-7.2) % | 352/5352 |
|  | Cardiology | 0 (0-0.1) % | 2/5547 | NA | NA | 0 (0-0.1) % | 2/5352 |
|  | Colorectal | 13.2 (12.3-14.1) % | 732/5547 | 13.8 (9.2-19) % | 27/195 | 13.2 (12.3-14.1) % | 705/5352 |
|  | Ear, nose & throat | 4.2 (3.7-4.7) % | 231/5547 | 0.5 (0-1.5) % | 1/195 | 4.3 (3.8-4.8) % | 230/5352 |
|  | Endocrine | 1 (0.7-1.2) % | 55/5547 | 0.5 (0-1.5) % | 1/195 | 1 (0.7-1.3) % | 54/5352 |
|  | Endoscopic | 0.3 (0.2-0.5) % | 17/5547 | NA | NA | 0.3 (0.2-0.5) % | 17/5352 |
|  | Gynaecology | 5.2 (4.7-5.8) % | 289/5547 | 2.1 (0.5-4.1) % | 4/195 | 5.3 (4.7-5.9) % | 285/5352 |
|  | Hepato-pancreato-biliary | 0.7 (0.5-1) % | 41/5547 | 3.6 (1-6.7) % | 7/195 | 0.6 (0.4-0.9) % | 34/5352 |
|  | Interventional radiology | 1 (0.7-1.3) % | 56/5547 | NA | NA | 1 (0.8-1.3) % | 56/5352 |
|  | Maxillo-facial and dental | 1.5 (1.2-1.8) % | 82/5547 | 2.1 (0.5-4.1) % | 4/195 | 1.5 (1.1-1.8) % | 78/5352 |
|  | Neurosurgery | 0.9 (0.6-1.1) % | 48/5547 | 3.1 (1-5.6) % | 6/195 | 0.8 (0.6-1) % | 42/5352 |
|  | Ophthalmic | 1.2 (1-1.5) % | 68/5547 | NA | NA | 1.3 (1-1.6) % | 68/5352 |
|  | Orthopaedics | 25.7 (24.6-26.9) % | 1426/5547 | 35.4 (28.7-42.1) % | 69/195 | 25.4 (24.2-26.6) % | 1357/5352 |
|  | Plastics | 3.6 (3.1-4.1) % | 198/5547 | 2.6 (0.5-5.1) % | 5/195 | 3.6 (3.1-4.1) % | 193/5352 |
|  | Spine | 1.7 (1.4-2.1) % | 97/5547 | 3.1 (1-5.6) % | 6/195 | 1.7 (1.4-2.1) % | 91/5352 |
|  | Thoracic | 4.7 (4.1-5.3) % | 259/5547 | 17.4 (12.3-22.6) % | 34/195 | 4.2 (3.6-4.7) % | 225/5352 |
|  | Transplant | 0 (0-0.1) % | 2/5547 | 0.5 (0-1.5) % | 1/195 | 0 (0-0.1) % | 1/5352 |
|  | Upper gastrointestinal | 5.9 (5.3-6.6) % | 328/5547 | 5.6 (2.6-9.2) % | 11/195 | 5.9 (5.3-6.5) % | 317/5352 |
|  | Urology | 20.2 (19.2-21.2) % | 1119/5547 | 5.6 (2.6-9.2) % | 11/195 | 20.7 (19.6-21.8) % | 1108/5352 |
|  | Vascular | 2.3 (1.9-2.7) % | 129/5547 | 3.6 (1-6.7) % | 7/195 | 2.3 (1.9-2.7) % | 122/5352 |
|  | Other | 0 (0-0.1) % | 2/5547 | NA | NA | 0 (0-0.1) % | 2/5352 |
|  | Unknown | 0.1 (0-0.1) % | 4/5547 | NA | NA | 0.1 (0-0.1) % | 4/5352 |
| Surgical urgency |  |  |  |  |  |  |  |
|  | Emergency | 2 (1.6-2.4) % | 112/5627 | 7.6 (4-11.6) % | 15/198 | 1.8 (1.5-2.1) % | 97/5429 |
|  | Urgent | 11.6 (10.8-12.5) % | 654/5627 | 29.8 (23.7-36.4) % | 59/198 | 11 (10.1-11.8) % | 595/5429 |
|  | Expedited | 12.3 (11.4-13.2) % | 692/5627 | 22.2 (16.7-27.8) % | 44/198 | 11.9 (11.1-12.8) % | 648/5429 |
|  | Planned | 74.1 (73-75.3) % | 4169/5627 | 40.4 (33.8-47.5) % | 80/198 | 75.3 (74.2-76.5) % | 4089/5429 |
| Source of admission |  |  |  |  |  |  |  |
|  | Own home | 97.2 (96.8-97.7) % | 5468/5623 | 94.4 (90.9-97.5) % | 187/198 | 97.3 (96.9-97.8) % | 5281/5425 |
|  | Sheltered housing | 0.9 (0.6-1.1) % | 49/5623 | 2 (0.5-4) % | 4/198 | 0.8 (0.6-1.1) % | 45/5425 |
|  | Residential or nursing home | 0.2 (0.1-0.4) % | 13/5623 | NA | NA | 0.2 (0.1-0.4) % | 13/5425 |
|  | Another secondary care hospital | 0.6 (0.4-0.9) % | 35/5623 | 3 (1-5.6) % | 6/198 | 0.5 (0.4-0.8) % | 29/5425 |
|  | Other | 0.8 (0.6-1.1) % | 46/5623 | 0.5 (0-1.5) % | 1/198 | 0.8 (0.6-1.1) % | 45/5425 |
|  | Unknown | 0.2 (0.1-0.3) % | 12/5623 | NA | NA | 0.2 (0.1-0.4) % | 12/5425 |
| Independence |  |  |  |  |  |  |  |
|  | Independent | 87.2 (86.3-88.1) % | 4813/5519 | 75.4 (69.1-81.2) % | 144/191 | 87.6 (86.7-88.5) % | 4669/5328 |
|  | Assistance with instrumental ADLs | 12.3 (11.4-13.2) % | 680/5519 | 23 (17.3-29.3) % | 44/191 | 11.9 (11.1-12.8) % | 636/5328 |
|  | Assistance with basic ADLs | 0.5 (0.3-0.7) % | 26/5519 | 1.6 (0-3.7) % | 3/191 | 0.4 (0.3-0.6) % | 23/5328 |
| Ethnicity |  |  |  |  |  |  |  |
|  | Asian | 1.9 (1.5-2.2) % | 104/5563 | 2.1 (0.5-4.1) % | 4/193 | 1.9 (1.5-2.2) % | 100/5370 |
|  | Black | 1.3 (1-1.6) % | 72/5563 | 0.5 (0-1.6) % | 1/193 | 1.3 (1-1.6) % | 71/5370 |
|  | Mixed | 0.5 (0.3-0.7) % | 26/5563 | NA | NA | 0.5 (0.3-0.7) % | 26/5370 |
|  | White | 96.4 (95.8-96.9) % | 5361/5563 | 97.4 (94.8-99.5) % | 188/193 | 96.3 (95.8-96.8) % | 5173/5370 |
| Education |  |  |  |  |  |  |  |
|  | Degree level | 25.1 (24-26.3) % | 1412/5622 | 22.7 (17.2-28.3) % | 45/198 | 25.2 (24.1-26.4) % | 1367/5424 |
|  | A levels / NVQ 3 | 12.6 (11.8-13.5) % | 711/5622 | 9.1 (5.1-13.1) % | 18/198 | 12.8 (11.9-13.7) % | 693/5424 |
|  | Apprenticeship | 6.8 (6.1-7.5) % | 382/5622 | 7.1 (3.5-10.6) % | 14/198 | 6.8 (6.1-7.5) % | 368/5424 |
|  | GCSEs / NVQ 2 | 15.1 (14.2-16.1) % | 851/5622 | 17.7 (12.6-23.2) % | 35/198 | 15 (14-16) % | 816/5424 |
|  | O level / NVQ 1 | 11.7 (10.8-12.5) % | 657/5622 | 12.6 (8.1-17.7) % | 25/198 | 11.7 (10.8-12.6) % | 632/5424 |
|  | No formal qualifications | 20.3 (19.3-21.3) % | 1144/5622 | 18.2 (12.6-23.7) % | 36/198 | 20.4 (19.4-21.5) % | 1108/5424 |
|  | Education not recorded | 8.3 (7.6-9) % | 465/5622 | 12.6 (8.1-17.2) % | 25/198 | 8.1 (7.4-8.8) % | 440/5424 |
| IMD deciles |  |  |  |  |  |  |  |
|  | 1 (most deprived) | 5.8 (5.2-6.4) % | 319/5486 | 8.3 (4.7-12.4) % | 16/193 | 5.7 (5.1-6.4) % | 303/5293 |
|  | 2 | 6.7 (6.1-7.4) % | 369/5486 | 6.2 (3.1-9.8) % | 12/193 | 6.7 (6.1-7.4) % | 357/5293 |
|  | 3 | 7.7 (7-8.4) % | 420/5486 | 6.7 (3.1-10.4) % | 13/193 | 7.7 (7-8.4) % | 407/5293 |
|  | 4 | 8.8 (8-9.5) % | 482/5486 | 7.8 (4.1-11.9) % | 15/193 | 8.8 (8.1-9.6) % | 467/5293 |
|  | 5 | 10.1 (9.3-10.9) % | 553/5486 | 7.8 (4.1-11.9) % | 15/193 | 10.2 (9.4-11) % | 538/5293 |
|  | 6 | 12.2 (11.4-13.1) % | 671/5486 | 16.1 (10.9-21.8) % | 31/193 | 12.1 (11.2-13) % | 640/5293 |
|  | 7 | 12.1 (11.2-13) % | 665/5486 | 12.4 (8.3-17.1) % | 24/193 | 12.1 (11.2-13) % | 641/5293 |
|  | 8 | 11.9 (11-12.7) % | 651/5486 | 13.5 (8.8-18.7) % | 26/193 | 11.8 (11-12.6) % | 625/5293 |
|  | 9 | 12.9 (12-13.9) % | 708/5486 | 10.4 (6.2-15) % | 20/193 | 13 (12.1-13.9) % | 688/5293 |
|  | 10 (least deprived) | 11.8 (10.9-12.6) % | 648/5486 | 10.9 (6.7-15) % | 21/193 | 11.8 (11-12.7) % | 627/5293 |
| Operative severity |  |  |  |  |  |  |  |
|  | Minor | 6.5 (5.8-7.1) % | 359/5546 | 3.6 (1-6.2) % | 7/195 | 6.6 (5.9-7.3) % | 352/5351 |
|  | Intermediate | 26.6 (25.6-27.8) % | 1478/5546 | 6.7 (3.6-10.3) % | 13/195 | 27.4 (26.2-28.6) % | 1465/5351 |
|  | Major | 30.2 (29-31.5) % | 1677/5546 | 24.1 (17.9-30.3) % | 47/195 | 30.5 (29.2-31.7) % | 1630/5351 |
|  | X Major | 23.8 (22.7-25) % | 1320/5546 | 31.8 (25.1-39) % | 62/195 | 23.5 (22.4-24.6) % | 1258/5351 |
|  | Complex | 12.8 (11.9-13.6) % | 708/5546 | 33.8 (27.2-41) % | 66/195 | 12 (11.1-12.9) % | 642/5351 |
|  | Unknown | 0.1 (0-0.1) % | 4/5546 | NA | NA | 0.1 (0-0.1) % | 4/5351 |
| Preop assessment |  |  |  |  |  |  |  |
|  | Nurse or AHP led clinic | 37.5 (36.3-38.8) % | 2043/5444 | 21.3 (15.4-27.7) % | 40/188 | 38.1 (36.8-39.4) % | 2003/5256 |
|  | Anaesthetist led clinic | 7.2 (6.5-7.9) % | 392/5444 | 8.5 (4.8-12.8) % | 16/188 | 7.2 (6.5-7.9) % | 376/5256 |
|  | Anaesthetist and geriatrician led clinics | 0.2 (0.1-0.3) % | 9/5444 | NA | NA | 0.2 (0.1-0.3) % | 9/5256 |
|  | Geriatrician led or MDT clinic | 1 (0.7-1.2) % | 52/5444 | 2.7 (0.5-5.3) % | 5/188 | 0.9 (0.6-1.1) % | 47/5256 |
|  | Physician (non geriatrician) led clinic | 1.1 (0.8-1.4) % | 59/5444 | 1.1 (0-2.7) % | 2/188 | 1.1 (0.8-1.4) % | 57/5256 |
|  | On the day assessment | 53.1 (51.7-54.4) % | 2889/5444 | 66.5 (60.1-72.9) % | 125/188 | 52.6 (51.3-54) % | 2764/5256 |
| Postop destination |  |  |  |  |  |  |  |
|  | Ward (level 0 or 1 care) | 85.6 (84.7-86.5) % | 4808/5618 | 58.6 (52-65.2) % | 116/198 | 86.6 (85.6-87.5) % | 4692/5420 |
|  | Unplanned admission to PACU or equivalent (level 1.5 care) | 0.4 (0.3-0.6) % | 25/5618 | 1.5 (0-3.5) % | 3/198 | 0.4 (0.2-0.6) % | 22/5420 |
|  | Planned admission to PACU or equivalent (level 1.5 care) | 6.3 (5.7-7) % | 354/5618 | 6.6 (3-10.1) % | 13/198 | 6.3 (5.6-6.9) % | 341/5420 |
|  | Unplanned admission to PACU or equivalent (level 2/3 care) | 0.1 (0-0.2) % | 6/5618 | 0.5 (0-1.5) % | 1/198 | 0.1 (0-0.2) % | 5/5420 |
|  | Planned admission to PACU or equivalent (level 2/3 care) | 1.4 (1.1-1.7) % | 78/5618 | 4 (1.5-7.1) % | 8/198 | 1.3 (1-1.6) % | 70/5420 |
|  | Unplanned critical care admission (level 2 or 3 care) | 0.6 (0.4-0.9) % | 36/5618 | 4 (1.5-7.1) % | 8/198 | 0.5 (0.3-0.7) % | 28/5420 |
|  | Planned critical care admission (level 2 or 3 care) | 5.3 (4.7-5.9) % | 298/5618 | 24.7 (18.7-31.3) % | 49/198 | 4.6 (4-5.2) % | 249/5420 |
| Urinary catheter |  |  |  |  |  |  |  |
|  | No catheter | 64.7 (63.5-66) % | 3637/5618 | 28.8 (22.7-34.8) % | 57/198 | 66.1 (64.8-67.4) % | 3580/5420 |
|  | Long-term/pre-admission catheter | 1.4 (1.1-1.7) % | 77/5618 | 4 (1.5-7.1) % | 8/198 | 1.3 (1-1.6) % | 69/5420 |
|  | Electively catheterised pre/intra-op | 30.1 (28.9-31.3) % | 1689/5618 | 60.6 (54-67.2) % | 120/198 | 28.9 (27.8-30.2) % | 1569/5420 |
|  | Catheterised post-op | 1.9 (1.6-2.3) % | 108/5618 | 4.5 (2-7.6) % | 9/198 | 1.8 (1.5-2.2) % | 99/5420 |

#### Supplementary table 5E: Demographics of participants having elective surgery in the SNAP-3 cohort, with and without delirium

|  |  | **All** | | **Delirium** | | **No delirium** | |
| --- | --- | --- | --- | --- | --- | --- | --- |
| **Characteristic** |  | **Summary** | **Number** | **Summary** | **Number** | **Summary** | **Number** |
| Sex |  |  |  |  |  |  |  |
|  | Female | 47.6 (46.1-49) % | 2340/4920 | 40.3 (32.1-47.8) % | 54/134 | 47.8 (46.4-49.1) % | 2286/4786 |
|  | Male | 52.4 (51.1-53.9) % | 2580/4920 | 59.7 (50.8-67.9) % | 80/134 | 52.2 (50.8-53.7) % | 2500/4786 |
| Age |  |  |  |  |  |  |  |
|  | Age (years) | 71.9 (7.3) | 4919 | 75.4 (7.6) | 134 | 71.8 (7.3) | 4785 |
|  | 60-69 | 39 (37.7-40.5) % | 1920/4919 | 24.6 (17.2-32.1) % | 33/134 | 39.4 (38.1-40.8) % | 1887/4785 |
|  | 70-79 | 42.3 (40.9-43.8) % | 2083/4919 | 43.3 (35.1-51.5) % | 58/134 | 42.3 (40.9-43.7) % | 2025/4785 |
|  | 80-89 | 17.3 (16.3-18.5) % | 853/4919 | 27.6 (20.2-35.8) % | 37/134 | 17.1 (16-18.2) % | 816/4785 |
|  | >90 | 1.3 (1-1.6) % | 63/4919 | 4.5 (1.5-8.2) % | 6/134 | 1.2 (0.9-1.5) % | 57/4785 |
| ASA |  |  |  |  |  |  |  |
|  | 1 | 8.1 (7.4-8.9) % | 395/4871 | 3 (0.8-6) % | 4/133 | 8.3 (7.5-9.1) % | 391/4738 |
|  | 2 | 58.6 (57.2-60) % | 2855/4871 | 33.8 (25.6-42.1) % | 45/133 | 59.3 (57.8-60.7) % | 2810/4738 |
|  | 3 | 31.5 (30.2-32.8) % | 1533/4871 | 54.9 (46.6-63.9) % | 73/133 | 30.8 (29.5-32.1) % | 1460/4738 |
|  | 4 | 1.7 (1.3-2.1) % | 82/4871 | 8.3 (3.8-13.5) % | 11/133 | 1.5 (1.2-1.9) % | 71/4738 |
|  | 5 | 0.1 (0-0.2) % | 6/4871 | NA | NA | 0.1 (0-0.2) % | 6/4738 |
| Frailty (CFS≥5) | Frail | 14.4 (13.4-15.4) % | 700/4869 | 39.8 (31.6-48.1) % | 53/133 | 13.7 (12.7-14.6) % | 647/4736 |
| Clinical Frailty Scale |  |  |  |  |  |  |  |
|  | 1 | 12.4 (11.5-13.3) % | 603/4869 | 5.3 (1.5-9.8) % | 7/133 | 12.6 (11.6-13.5) % | 596/4736 |
|  | 2 | 20.7 (19.5-21.8) % | 1008/4869 | 12.8 (7.5-18.8) % | 17/133 | 20.9 (19.8-22.1) % | 991/4736 |
|  | 3 | 33 (31.7-34.4) % | 1609/4869 | 23.3 (16.5-30.8) % | 31/133 | 33.3 (32-34.6) % | 1578/4736 |
|  | 4 | 19.5 (18.4-20.6) % | 949/4869 | 18.8 (12.8-26.3) % | 25/133 | 19.5 (18.4-20.7) % | 924/4736 |
|  | 5 | 8.6 (7.8-9.4) % | 418/4869 | 21.8 (15-28.6) % | 29/133 | 8.2 (7.4-9) % | 389/4736 |
|  | 6 | 4.3 (3.7-4.9) % | 210/4869 | 10.5 (6-15.8) % | 14/133 | 4.1 (3.6-4.7) % | 196/4736 |
|  | 7 | 1.4 (1-1.7) % | 67/4869 | 6.8 (3-11.3) % | 9/133 | 1.2 (0.9-1.5) % | 58/4736 |
|  | 8 | 0.1 (0-0.2) % | 5/4869 | 0.8 (0-2.3) % | 1/133 | 0.1 (0-0.2) % | 4/4736 |
|  | 9 |  |  |  |  |  |  |
| Multimorbidity (≥2 comorbidities) | Multimorbid | 61.1 (59.7-62.5) % | 2650/4338 | 74.2 (66.4-82) % | 95/128 | 60.7 (59.2-62.2) % | 2555/4210 |
| Number of comorbidities | Number of comorbidities | 2 (1-3) | 4338 | 2.5 (1-4) | 128 | 2 (1-3) | 4210 |
| Dementia |  | 1 (0.8-1.3) % | 51/4921 | 6.7 (3-11.2) % | 9/134 | 0.9 (0.6-1.1) % | 42/4787 |
| Consent |  |  |  |  |  |  |  |
|  | Capacity | 99.2 (98.9-99.5) % | 4882/4921 | 92.5 (88.1-96.3) % | 124/134 | 99.4 (99.2-99.6) % | 4758/4787 |
|  | Consultee | 0.8 (0.6-1.1) % | 39/4921 | 7.5 (3.7-11.9) % | 10/134 | 0.6 (0.4-0.8) % | 29/4787 |
| Surgical specialty |  |  |  |  |  |  |  |
|  | Bariatric | 0.2 (0.1-0.3) % | 8/4851 | NA | NA | 0.2 (0.1-0.3) % | 8/4717 |
|  | Breast | 6.1 (5.4-6.8) % | 294/4851 | 0.7 (0-2.2) % | 1/134 | 6.2 (5.5-6.9) % | 293/4717 |
|  | Cardiology | 0 (0-0.1) % | 2/4851 | NA | NA | 0 (0-0.1) % | 2/4717 |
|  | Colorectal | 12.8 (11.9-13.7) % | 622/4851 | 14.9 (9-21.6) % | 20/134 | 12.8 (11.8-13.8) % | 602/4717 |
|  | Ear, nose & throat | 4.4 (3.8-5) % | 212/4851 | 1.5 (0-3.7) % | 2/134 | 4.5 (3.9-5) % | 210/4717 |
|  | Endocrine | 1.2 (0.9-1.5) % | 56/4851 | 0.7 (0-2.2) % | 1/134 | 1.2 (0.9-1.5) % | 55/4717 |
|  | Endoscopic | 0.4 (0.2-0.6) % | 19/4851 | 0.7 (0-2.2) % | 1/134 | 0.4 (0.2-0.6) % | 18/4717 |
|  | Gynaecology | 6 (5.3-6.6) % | 289/4851 | 2.2 (0-5.2) % | 3/134 | 6.1 (5.4-6.7) % | 286/4717 |
|  | Hepato-pancreato-biliary | 0.7 (0.5-0.9) % | 33/4851 | 3.7 (0.7-7.5) % | 5/134 | 0.6 (0.4-0.8) % | 28/4717 |
|  | Interventional radiology | 0.8 (0.5-1.1) % | 39/4851 | 1.5 (0-3.7) % | 2/134 | 0.8 (0.6-1.1) % | 37/4717 |
|  | Maxillo-facial and dental | 1.5 (1.2-1.8) % | 72/4851 | 2.2 (0-5.2) % | 3/134 | 1.5 (1.1-1.8) % | 69/4717 |
|  | Neurosurgery | 0.7 (0.5-0.9) % | 33/4851 | 1.5 (0-3.7) % | 2/134 | 0.7 (0.4-0.9) % | 31/4717 |
|  | Ophthalmic | 1.5 (1.1-1.8) % | 71/4851 | NA | NA | 1.5 (1.2-1.9) % | 71/4717 |
|  | Orthopaedics | 24 (22.8-25.2) % | 1164/4851 | 31.3 (23.9-39.6) % | 42/134 | 23.8 (22.6-25) % | 1122/4717 |
|  | Plastics | 3.2 (2.7-3.6) % | 154/4851 | NA | NA | 3.3 (2.8-3.8) % | 154/4717 |
|  | Spine | 2.3 (1.9-2.8) % | 113/4851 | 4.5 (1.5-8.2) % | 6/134 | 2.3 (1.9-2.7) % | 107/4717 |
|  | Thoracic | 3.6 (3.1-4.1) % | 174/4851 | 17.2 (11.2-23.9) % | 23/134 | 3.2 (2.7-3.7) % | 151/4717 |
|  | Transplant | 0 (0-0.1) % | 1/4851 | NA | NA | 0 (0-0.1) % | 1/4717 |
|  | Upper gastrointestinal | 5.4 (4.8-6.1) % | 263/4851 | 3 (0.7-6) % | 4/134 | 5.5 (4.8-6.1) % | 259/4717 |
|  | Urology | 22.8 (21.6-24) % | 1107/4851 | 9.7 (5.2-14.9) % | 13/134 | 23.2 (22-24.4) % | 1094/4717 |
|  | Vascular | 2.5 (2-2.9) % | 120/4851 | 4.5 (1.5-8.2) % | 6/134 | 2.4 (2-2.9) % | 114/4717 |
|  | Other | 0 (0-0.1) % | 2/4851 | NA | NA | 0 (0-0.1) % | 2/4717 |
|  | Unknown | 0.1 (0-0.1) % | 3/4851 | NA | NA | 0.1 (0-0.1) % | 3/4717 |
|  | Planned | 100 (NA-NA) % | 4921/4921 | 100 (NA-NA) % | 134/134 | 100 (NA-NA) % | 4787/4787 |
| Source of admission |  |  |  |  |  |  |  |
|  | Own home | 97.2 (96.7-97.6) % | 4780/4918 | 93.3 (88.8-97) % | 125/134 | 97.3 (96.9-97.7) % | 4655/4784 |
|  | Sheltered housing | 1.1 (0.8-1.4) % | 55/4918 | 3 (0.7-6) % | 4/134 | 1.1 (0.8-1.4) % | 51/4784 |
|  | Residential or nursing home | 0.4 (0.3-0.7) % | 22/4918 | 1.5 (0-3.7) % | 2/134 | 0.4 (0.3-0.6) % | 20/4784 |
|  | Rehabilitation facility | 0.1 (0-0.1) % | 3/4918 | NA | NA | 0.1 (0-0.1) % | 3/4784 |
|  | Another secondary care hospital | 0.1 (0-0.2) % | 4/4918 | 1.5 (0-3.7) % | 2/134 | 0 (0-0.1) % | 2/4784 |
|  | Other | 0.7 (0.5-1) % | 35/4918 | 0.7 (0-2.2) % | 1/134 | 0.7 (0.5-1) % | 34/4784 |
|  | Unknown | 0.4 (0.2-0.6) % | 19/4918 | NA | NA | 0.4 (0.2-0.6) % | 19/4784 |
| Independence |  |  |  |  |  |  |  |
|  | Independent | 77.3 (76-78.4) % | 3738/4838 | 55.7 (47.3-64.9) % | 73/131 | 77.9 (76.7-79) % | 3665/4707 |
|  | Assistance with instrumental ADLs | 20.8 (19.7-21.9) % | 1006/4838 | 36.6 (29-45) % | 48/131 | 20.4 (19.2-21.5) % | 958/4707 |
|  | Assistance with basic ADLs | 1.9 (1.6-2.3) % | 94/4838 | 7.6 (3.8-12.2) % | 10/131 | 1.8 (1.4-2.2) % | 84/4707 |
| Ethnicity |  |  |  |  |  |  |  |
|  | Asian | 2.1 (1.7-2.5) % | 101/4858 | 2.3 (0-5.3) % | 3/133 | 2.1 (1.7-2.5) % | 98/4725 |
|  | Black | 1.5 (1.2-1.9) % | 74/4858 | 3 (0.8-6) % | 4/133 | 1.5 (1.1-1.8) % | 70/4725 |
|  | Mixed | 0.6 (0.4-0.8) % | 27/4858 | NA | NA | 0.6 (0.4-0.8) % | 27/4725 |
|  | White | 95.8 (95.3-96.4) % | 4656/4858 | 94.7 (91-97.7) % | 126/133 | 95.9 (95.3-96.4) % | 4530/4725 |
| Education |  |  |  |  |  |  |  |
|  | Degree level | 23.5 (22.3-24.7) % | 1153/4916 | 15.7 (9.7-22.4) % | 21/134 | 23.7 (22.5-24.9) % | 1132/4782 |
|  | A levels / NVQ 3 | 12.7 (11.8-13.6) % | 624/4916 | 11.2 (6-17.2) % | 15/134 | 12.7 (11.8-13.7) % | 609/4782 |
|  | Apprenticeship | 6.5 (5.9-7.3) % | 321/4916 | 6 (2.2-10.4) % | 8/134 | 6.5 (5.8-7.2) % | 313/4782 |
|  | GCSEs / NVQ 2 | 14.5 (13.5-15.4) % | 711/4916 | 16.4 (10.4-22.4) % | 22/134 | 14.4 (13.4-15.4) % | 689/4782 |
|  | O level / NVQ 1 | 11.7 (10.8-12.6) % | 573/4916 | 16.4 (10.4-22.4) % | 22/134 | 11.5 (10.7-12.5) % | 551/4782 |
|  | No formal qualifications | 22.1 (20.9-23.3) % | 1085/4916 | 23.9 (16.4-31.3) % | 32/134 | 22 (20.8-23.2) % | 1053/4782 |
|  | Education not recorded | 9.1 (8.4-9.9) % | 449/4916 | 10.4 (5.2-15.7) % | 14/134 | 9.1 (8.3-9.9) % | 435/4782 |
| IMD deciles |  |  |  |  |  |  |  |
|  | 1 (most deprived) | 6.5 (5.8-7.2) % | 314/4802 | 6.2 (2.3-10.9) % | 8/128 | 6.5 (5.9-7.3) % | 306/4674 |
|  | 2 | 7.3 (6.6-8) % | 351/4802 | 7 (3.1-11.7) % | 9/128 | 7.3 (6.5-8.1) % | 342/4674 |
|  | 3 | 8.3 (7.5-9.1) % | 398/4802 | 7.8 (3.1-13.3) % | 10/128 | 8.3 (7.5-9.1) % | 388/4674 |
|  | 4 | 8.5 (7.7-9.3) % | 409/4802 | 9.4 (4.7-14.1) % | 12/128 | 8.5 (7.7-9.3) % | 397/4674 |
|  | 5 | 9.7 (8.9-10.6) % | 466/4802 | 4.7 (1.6-8.6) % | 6/128 | 9.8 (9-10.7) % | 460/4674 |
|  | 6 | 11.6 (10.8-12.6) % | 559/4802 | 15.6 (10.2-21.9) % | 20/128 | 11.5 (10.6-12.4) % | 539/4674 |
|  | 7 | 11.8 (10.9-12.7) % | 566/4802 | 10.2 (5.5-15.6) % | 13/128 | 11.8 (11-12.8) % | 553/4674 |
|  | 8 | 12.4 (11.5-13.3) % | 594/4802 | 16.4 (10.2-23.4) % | 21/128 | 12.3 (11.3-13.2) % | 573/4674 |
|  | 9 | 12.6 (11.6-13.5) % | 603/4802 | 11.7 (6.2-18) % | 15/128 | 12.6 (11.6-13.5) % | 588/4674 |
|  | 10 (least deprived) | 11.3 (10.3-12.2) % | 542/4802 | 10.9 (6.2-16.4) % | 14/128 | 11.3 (10.4-12.2) % | 528/4674 |
| Operative severity |  |  |  |  |  |  |  |
|  | Minor | 6.5 (5.8-7.1) % | 313/4850 | 1.5 (0-3.7) % | 2/134 | 6.6 (5.9-7.3) % | 311/4716 |
|  | Intermediate | 27.5 (26.3-28.8) % | 1336/4850 | 5.2 (2.2-9) % | 7/134 | 28.2 (26.8-29.4) % | 1329/4716 |
|  | Major | 27.9 (26.7-29.2) % | 1355/4850 | 17.2 (11.2-23.9) % | 23/134 | 28.2 (26.9-29.6) % | 1332/4716 |
|  | X Major | 25.7 (24.5-26.9) % | 1248/4850 | 37.3 (29.1-45.5) % | 50/134 | 25.4 (24.2-26.7) % | 1198/4716 |
|  | Complex | 12.3 (11.3-13.1) % | 595/4850 | 38.8 (30.6-47.7) % | 52/134 | 11.5 (10.6-12.4) % | 543/4716 |
|  | Unknown | 0.1 (0-0.1) % | 3/4850 | NA | NA | 0.1 (0-0.1) % | 3/4716 |
| Preop assessment |  |  |  |  |  |  |  |
|  | Nurse or AHP led clinic | 44.9 (43.6-46.4) % | 2164/4819 | 45.7 (37.2-54.3) % | 59/129 | 44.9 (43.5-46.3) % | 2105/4690 |
|  | Anaesthetist led clinic | 8.7 (7.9-9.5) % | 418/4819 | 12.4 (7-17.8) % | 16/129 | 8.6 (7.8-9.4) % | 402/4690 |
|  | Anaesthetist and geriatrician led clinics | 0.2 (0.1-0.4) % | 11/4819 | 0.8 (0-2.3) % | 1/129 | 0.2 (0.1-0.4) % | 10/4690 |
|  | Geriatrician led or MDT clinic | 1.1 (0.8-1.4) % | 51/4819 | 3.1 (0.8-6.2) % | 4/129 | 1 (0.7-1.3) % | 47/4690 |
|  | Physician (non geriatrician) led clinic | 1.3 (1-1.6) % | 63/4819 | 1.6 (0-3.9) % | 2/129 | 1.3 (1-1.6) % | 61/4690 |
|  | On the day assessment | 43.8 (42.4-45.2) % | 2112/4819 | 36.4 (27.9-45) % | 47/129 | 44 (42.5-45.5) % | 2065/4690 |
| Postop destination |  |  |  |  |  |  |  |
|  | Ward (level 0 or 1 care) | 87.1 (86.1-88) % | 4277/4913 | 55.2 (46.3-63.4) % | 74/134 | 87.9 (87-88.8) % | 4203/4779 |
|  | Unplanned admission to PACU or equivalent (level 1.5 care) | 0.5 (0.3-0.7) % | 23/4913 | 1.5 (0-3.7) % | 2/134 | 0.4 (0.3-0.6) % | 21/4779 |
|  | Planned admission to PACU or equivalent (level 1.5 care) | 6.1 (5.5-6.8) % | 301/4913 | 11.9 (6.7-17.9) % | 16/134 | 6 (5.3-6.7) % | 285/4779 |
|  | Unplanned admission to PACU or equivalent (level 2/3 care) | 0.1 (0-0.2) % | 5/4913 | NA | NA | 0.1 (0-0.2) % | 5/4779 |
|  | Planned admission to PACU or equivalent (level 2/3 care) | 1.6 (1.2-2) % | 78/4913 | 5.2 (1.5-9) % | 7/134 | 1.5 (1.2-1.8) % | 71/4779 |
|  | Unplanned critical care admission (level 2 or 3 care) | 0.4 (0.3-0.6) % | 22/4913 | 4.5 (1.5-8.2) % | 6/134 | 0.3 (0.2-0.5) % | 16/4779 |
|  | Planned critical care admission (level 2 or 3 care) | 3.9 (3.4-4.5) % | 194/4913 | 21.6 (14.9-29.1) % | 29/134 | 3.5 (2.9-4) % | 165/4779 |
| Urinary catheter |  |  |  |  |  |  |  |
|  | No catheter | 66.9 (65.6-68.2) % | 3288/4913 | 29.1 (21.6-36.6) % | 39/134 | 68 (66.6-69.3) % | 3249/4779 |
|  | Long-term/pre-admission catheter | 1.7 (1.3-2.1) % | 82/4913 | 3 (0.7-6) % | 4/134 | 1.6 (1.3-2) % | 78/4779 |
|  | Electively catheterised pre/intra-op | 27.7 (26.5-29) % | 1363/4913 | 60.4 (52.2-68.7) % | 81/134 | 26.8 (25.5-28.1) % | 1282/4779 |
|  | Catheterised post-op | 1.9 (1.5-2.3) % | 92/4913 | 6.7 (3-11.2) % | 9/134 | 1.7 (1.4-2.1) % | 83/4779 |

#### Supplementary table 5F: Demographics of participants having non-elective surgery in the SNAP-3 cohort, with and without delirium

|  |  | **All** | | **Delirium** | | **No delirium** | |
| --- | --- | --- | --- | --- | --- | --- | --- |
| **Characteristic** |  | **Summary** | **Number** | **Summary** | **Number** | **Summary** | **Number** |
| Sex |  |  |  |  |  |  |  |
|  | Female | 52.7 (50.6-54.7) % | 1124/2134 | 57.7 (52.5-62.9) % | 199/345 | 51.7 (49.4-54) % | 925/1789 |
|  | Male | 47.3 (45.1-49.4) % | 1010/2134 | 42.3 (37.1-47.2) % | 146/345 | 48.3 (46-50.7) % | 864/1789 |
| Age |  |  |  |  |  |  |  |
|  | Age (years) | 75 (9.2) | 2131 | 80.6 (9.6) | 344 | 73.9 (8.7) | 1787 |
|  | 60-69 | 31.2 (29.2-33.2) % | 665/2131 | 15.1 (11.3-18.9) % | 52/344 | 34.3 (32-36.6) % | 613/1787 |
|  | 70-79 | 35.3 (33.2-37.3) % | 752/2131 | 25 (20.3-29.9) % | 86/344 | 37.3 (35-39.6) % | 666/1787 |
|  | 80-89 | 25.7 (23.8-27.5) % | 547/2131 | 39 (33.7-43.9) % | 134/344 | 23.1 (21.2-25.1) % | 413/1787 |
|  | >90 | 7.8 (6.7-9) % | 167/2131 | 20.9 (16.6-25.3) % | 72/344 | 5.3 (4.3-6.4) % | 95/1787 |
| ASA |  |  |  |  |  |  |  |
|  | 1 | 5.6 (4.6-6.5) % | 118/2123 | NA | NA | 6.6 (5.6-7.8) % | 118/1778 |
|  | 2 | 40.9 (38.8-43.1) % | 869/2123 | 17.4 (13.6-21.4) % | 60/345 | 45.5 (43.3-47.9) % | 809/1778 |
|  | 3 | 45 (42.8-47.2) % | 955/2123 | 62.3 (57.1-67.5) % | 215/345 | 41.6 (39.4-44) % | 740/1778 |
|  | 4 | 8.3 (7.2-9.6) % | 177/2123 | 19.7 (15.4-23.8) % | 68/345 | 6.1 (5-7.3) % | 109/1778 |
|  | 5 | 0.2 (0-0.4) % | 4/2123 | 0.6 (0-1.4) % | 2/345 | 0.1 (0-0.3) % | 2/1778 |
| Frailty (CFS≥5) | Frail | 31.3 (29.4-33.2) % | 664/2122 | 65.6 (60.6-70.8) % | 225/343 | 24.7 (22.7-26.8) % | 439/1779 |
| Clinical Frailty Scale |  |  |  |  |  |  |  |
|  | 1 | 9.7 (8.3-11) % | 205/2122 | 2.3 (0.9-4.1) % | 8/343 | 11.1 (9.7-12.5) % | 197/1779 |
|  | 2 | 16.2 (14.5-17.7) % | 343/2122 | 5.2 (2.9-7.6) % | 18/343 | 18.3 (16.6-20.1) % | 325/1779 |
|  | 3 | 25 (23.1-26.9) % | 531/2122 | 11.1 (7.6-14.6) % | 38/343 | 27.7 (25.6-29.8) % | 493/1779 |
|  | 4 | 17.9 (16.3-19.5) % | 379/2122 | 15.7 (12-19.5) % | 54/343 | 18.3 (16.5-20.1) % | 325/1779 |
|  | 5 | 13.1 (11.7-14.6) % | 278/2122 | 20.7 (16.6-24.8) % | 71/343 | 11.6 (10.2-13.1) % | 207/1779 |
|  | 6 | 9.9 (8.7-11.2) % | 211/2122 | 20.4 (16.3-24.8) % | 70/343 | 7.9 (6.7-9.2) % | 141/1779 |
|  | 7 | 7.3 (6.2-8.5) % | 155/2122 | 21 (16.6-25.4) % | 72/343 | 4.7 (3.7-5.7) % | 83/1779 |
|  | 8 | 0.9 (0.6-1.4) % | 20/2122 | 3.5 (1.7-5.5) % | 12/343 | 0.4 (0.2-0.8) % | 8/1779 |
|  | 9 |  |  |  |  |  |  |
| Multimorbidity (≥2 comorbidities) | Multimorbid | 67.6 (65.4-69.6) % | 1324/1960 | 82.2 (78-86.1) % | 273/332 | 64.6 (62.3-67) % | 1051/1628 |
| Number of comorbidities | Number of comorbidities | 2 (1-4) | 1960 | 3 (2-4) | 332 | 2 (1-3) | 1628 |
| Dementia | NA | 6.2 (5.2-7.3) % | 133/2134 | 26.7 (22-31) % | 92/345 | 2.3 (1.6-3) % | 41/1789 |
| Consent |  |  |  |  |  |  |  |
|  | Capacity | 90.7 (89.5-91.9) % | 1936/2134 | 62.3 (57.1-67.5) % | 215/345 | 96.2 (95.3-97.1) % | 1721/1789 |
|  | Consultee | 9.3 (8-10.5) % | 198/2134 | 37.7 (32.8-42.6) % | 130/345 | 3.8 (2.9-4.7) % | 68/1789 |
| Surgical specialty |  |  |  |  |  |  |  |
|  | Bariatric | 0.1 (0-0.2) % | 2/2108 | NA | NA | 0.1 (0-0.3) % | 2/1766 |
|  | Breast | 4.4 (3.6-5.3) % | 92/2108 | NA | NA | 5.2 (4.2-6.2) % | 92/1766 |
|  | Colorectal | 10.8 (9.5-12.2) % | 228/2108 | 7.3 (4.7-10.2) % | 25/342 | 11.5 (10-13) % | 203/1766 |
|  | Ear, nose & throat | 2.2 (1.7-2.9) % | 47/2108 | NA | NA | 2.7 (1.9-3.5) % | 47/1766 |
|  | Endocrine | 0.2 (0-0.5) % | 5/2108 | NA | NA | 0.3 (0.1-0.6) % | 5/1766 |
|  | Endoscopic | 0.3 (0.1-0.5) % | 6/2108 | NA | NA | 0.3 (0.1-0.6) % | 6/1766 |
|  | Gynaecology | 2.3 (1.7-2.9) % | 48/2108 | 0.3 (0-0.9) % | 1/342 | 2.7 (1.9-3.5) % | 47/1766 |
|  | Hepato-pancreato-biliary | 0.5 (0.2-0.8) % | 10/2108 | 1.2 (0.3-2.3) % | 4/342 | 0.3 (0.1-0.6) % | 6/1766 |
|  | Interventional radiology | 1.9 (1.3-2.5) % | 39/2108 | 2 (0.6-3.5) % | 7/342 | 1.8 (1.2-2.5) % | 32/1766 |
|  | Maxillo-facial and dental | 1 (0.6-1.4) % | 21/2108 | 0.6 (0-1.5) % | 2/342 | 1.1 (0.6-1.6) % | 19/1766 |
|  | Neurosurgery | 1.6 (1-2.1) % | 33/2108 | 2.9 (1.2-5) % | 10/342 | 1.3 (0.8-1.9) % | 23/1766 |
|  | Ophthalmic | 0.6 (0.3-0.9) % | 12/2108 | NA | NA | 0.7 (0.3-1.1) % | 12/1766 |
|  | Orthopaedics | 43 (40.7-45.1) % | 906/2108 | 66.4 (61.4-71.3) % | 227/342 | 38.4 (36.2-40.7) % | 679/1766 |
|  | Plastics | 5.3 (4.3-6.3) % | 112/2108 | 3.2 (1.5-5.3) % | 11/342 | 5.7 (4.6-6.8) % | 101/1766 |
|  | Spine | 1.4 (0.9-1.9) % | 30/2108 | 1.8 (0.6-3.5) % | 6/342 | 1.4 (0.8-1.9) % | 24/1766 |
|  | Thoracic | 5.5 (4.5-6.5) % | 116/2108 | 4.7 (2.6-7) % | 16/342 | 5.7 (4.6-6.8) % | 100/1766 |
|  | Transplant | 0.1 (0-0.2) % | 2/2108 | 0.3 (0-0.9) % | 1/342 | 0.1 (0-0.2) % | 1/1766 |
|  | Upper gastrointestinal | 5 (4.1-5.9) % | 105/2108 | 4.7 (2.6-7) % | 16/342 | 5 (4-6.1) % | 89/1766 |
|  | Urology | 10.8 (9.5-12) % | 227/2108 | 3.2 (1.5-5.3) % | 11/342 | 12.2 (10.7-13.8) % | 216/1766 |
|  | Vascular | 3.1 (2.4-3.9) % | 66/2108 | 1.5 (0.3-2.9) % | 5/342 | 3.5 (2.6-4.3) % | 61/1766 |
|  | Unknown | 0 (0-0.1) % | 1/2108 | NA | NA | 0.1 (0-0.2) % | 1/1766 |
|  | Emergency | 7.5 (6.4-8.7) % | 160/2134 | 8.7 (5.8-11.6) % | 30/345 | 7.3 (6.1-8.4) % | 130/1789 |
|  | Urgent | 49.7 (47.6-51.9) % | 1060/2134 | 62.3 (57.1-67.2) % | 215/345 | 47.2 (44.9-49.5) % | 845/1789 |
|  | Expedited | 42.8 (40.6-44.9) % | 914/2134 | 29 (24.1-33.9) % | 100/345 | 45.5 (43.3-47.8) % | 814/1789 |
| Source of admission |  |  |  |  |  |  |  |
|  | Own home | 90.2 (89-91.4) % | 1925/2133 | 77.7 (73.3-82) % | 268/345 | 92.7 (91.4-93.8) % | 1657/1788 |
|  | Sheltered housing | 2.1 (1.5-2.8) % | 45/2133 | 1.4 (0.3-2.9) % | 5/345 | 2.2 (1.6-2.9) % | 40/1788 |
|  | Residential or nursing home | 4.1 (3.2-4.9) % | 87/2133 | 16.2 (12.5-20.3) % | 56/345 | 1.7 (1.1-2.3) % | 31/1788 |
|  | Rehabilitation facility | 0.2 (0-0.5) % | 5/2133 | 0.9 (0-2) % | 3/345 | 0.1 (0-0.3) % | 2/1788 |
|  | Another secondary care hospital | 1.9 (1.4-2.4) % | 40/2133 | 2.3 (0.9-4.1) % | 8/345 | 1.8 (1.2-2.4) % | 32/1788 |
|  | Other | 1.3 (0.8-1.8) % | 27/2133 | 1.4 (0.3-2.9) % | 5/345 | 1.2 (0.8-1.8) % | 22/1788 |
|  | Unknown | 0.2 (0-0.4) % | 4/2133 | NA | NA | 0.2 (0.1-0.4) % | 4/1788 |
| Independence |  |  |  |  |  |  |  |
|  | Independent | 62.4 (60.1-64.6) % | 1251/2004 | 31.3 (26.2-36.7) % | 92/294 | 67.8 (65.6-70.1) % | 1159/1710 |
|  | Assistance with instrumental ADLs | 31.2 (29.1-33.3) % | 625/2004 | 51 (45.6-56.5) % | 150/294 | 27.8 (25.7-29.8) % | 475/1710 |
|  | Assistance with basic ADLs | 6.4 (5.3-7.5) % | 128/2004 | 17.7 (13.3-22.1) % | 52/294 | 4.4 (3.5-5.5) % | 76/1710 |
| Ethnicity |  |  |  |  |  |  |  |
|  | Asian | 1.8 (1.2-2.3) % | 37/2114 | 1.8 (0.6-3.2) % | 6/339 | 1.7 (1.2-2.4) % | 31/1775 |
|  | Black | 1.2 (0.8-1.7) % | 26/2114 | 0.9 (0-2.1) % | 3/339 | 1.3 (0.8-1.9) % | 23/1775 |
|  | Mixed | 0.4 (0.2-0.7) % | 9/2114 | 0.3 (0-0.9) % | 1/339 | 0.5 (0.2-0.8) % | 8/1775 |
|  | White | 96.6 (95.8-97.4) % | 2042/2114 | 97.1 (95.3-98.8) % | 329/339 | 96.5 (95.7-97.4) % | 1713/1775 |
| Education |  |  |  |  |  |  |  |
|  | Degree level | 20.9 (19.2-22.8) % | 446/2132 | 15.7 (11.9-19.5) % | 54/344 | 21.9 (20-23.9) % | 392/1788 |
|  | A levels / NVQ 3 | 10.8 (9.6-12.2) % | 230/2132 | 9 (6.4-12.2) % | 31/344 | 11.1 (9.7-12.5) % | 199/1788 |
|  | Apprenticeship | 6.9 (5.9-8) % | 147/2132 | 6.7 (4.1-9.3) % | 23/344 | 6.9 (5.8-8.1) % | 124/1788 |
|  | GCSEs / NVQ 2 | 13.7 (12.3-15.1) % | 292/2132 | 9 (6.1-12.2) % | 31/344 | 14.6 (13-16.2) % | 261/1788 |
|  | O level / NVQ 1 | 11.8 (10.5-13.3) % | 252/2132 | 8.1 (5.2-11) % | 28/344 | 12.5 (11-14.1) % | 224/1788 |
|  | No formal qualifications | 24.1 (22.3-25.8) % | 513/2132 | 28.2 (23.5-33.4) % | 97/344 | 23.3 (21.3-25.3) % | 416/1788 |
|  | Education not recorded | 11.8 (10.5-13.3) % | 252/2132 | 23.3 (18.6-27.9) % | 80/344 | 9.6 (8.2-11) % | 172/1788 |
| IMD deciles |  |  |  |  |  |  |  |
|  | 1 (most deprived) | 7.4 (6.4-8.6) % | 154/2072 | 9.4 (6.4-12.7) % | 31/330 | 7.1 (5.9-8.3) % | 123/1742 |
|  | 2 | 8.2 (6.9-9.4) % | 169/2072 | 7.3 (4.5-10.3) % | 24/330 | 8.3 (7.1-9.6) % | 145/1742 |
|  | 3 | 8.1 (6.9-9.3) % | 168/2072 | 7.9 (5.2-10.9) % | 26/330 | 8.2 (7-9.4) % | 142/1742 |
|  | 4 | 9.8 (8.4-11.1) % | 203/2072 | 9.4 (6.4-12.7) % | 31/330 | 9.9 (8.5-11.4) % | 172/1742 |
|  | 5 | 10.5 (9.1-11.7) % | 217/2072 | 10.6 (7.6-14.2) % | 35/330 | 10.4 (9-11.9) % | 182/1742 |
|  | 6 | 13 (11.6-14.4) % | 270/2072 | 12.1 (8.8-15.8) % | 40/330 | 13.2 (11.7-14.8) % | 230/1742 |
|  | 7 | 11.8 (10.5-13.2) % | 245/2072 | 12.7 (9.1-16.4) % | 42/330 | 11.7 (10.2-13.3) % | 203/1742 |
|  | 8 | 9.1 (7.9-10.4) % | 189/2072 | 10.6 (7.3-14.2) % | 35/330 | 8.8 (7.6-10.2) % | 154/1742 |
|  | 9 | 12.2 (10.8-13.6) % | 252/2072 | 11.8 (8.5-15.5) % | 39/330 | 12.2 (10.7-13.8) % | 213/1742 |
|  | 10 (least deprived) | 9.9 (8.6-11.2) % | 205/2072 | 8.2 (5.2-11.2) % | 27/330 | 10.2 (8.8-11.6) % | 178/1742 |
| Operative severity |  |  |  |  |  |  |  |
|  | Minor | 5.6 (4.7-6.7) % | 119/2108 | 2.3 (0.9-4.1) % | 8/342 | 6.3 (5.2-7.4) % | 111/1766 |
|  | Intermediate | 21.3 (19.4-23.1) % | 448/2108 | 10.2 (7-13.7) % | 35/342 | 23.4 (21.5-25.3) % | 413/1766 |
|  | Major | 37.6 (35.5-39.7) % | 792/2108 | 41.5 (36-47.1) % | 142/342 | 36.8 (34.5-39) % | 650/1766 |
|  | X Major | 23.2 (21.4-25) % | 489/2108 | 34.2 (29.2-39.2) % | 117/342 | 21.1 (19.2-23) % | 372/1766 |
|  | Complex | 12.3 (10.9-13.7) % | 259/2108 | 11.7 (8.5-15.2) % | 40/342 | 12.4 (10.8-13.9) % | 219/1766 |
|  | Unknown | 0 (0-0.1) % | 1/2108 | NA | NA | 0.1 (0-0.2) % | 1/1766 |
| Preop assessment |  |  |  |  |  |  |  |
|  | Nurse or AHP led clinic | 12 (10.6-13.5) % | 236/1970 | 3.2 (1.6-5.1) % | 10/316 | 13.7 (12-15.3) % | 226/1654 |
|  | Anaesthetist led clinic | 3.7 (2.8-4.5) % | 72/1970 | 2.2 (0.9-4.1) % | 7/316 | 3.9 (3-4.9) % | 65/1654 |
|  | Anaesthetist and geriatrician led clinics | 0.2 (0-0.4) % | 3/1970 | NA | NA | 0.2 (0-0.4) % | 3/1654 |
|  | Geriatrician led or MDT clinic | 0.7 (0.3-1) % | 13/1970 | 0.3 (0-0.9) % | 1/316 | 0.7 (0.4-1.1) % | 12/1654 |
|  | Physician (non geriatrician) led clinic | 0.7 (0.3-1.1) % | 13/1970 | 0.6 (0-1.6) % | 2/316 | 0.7 (0.3-1.1) % | 11/1654 |
|  | On the day assessment | 82.9 (81.1-84.5) % | 1633/1970 | 93.7 (90.8-96.2) % | 296/316 | 80.8 (78.8-82.8) % | 1337/1654 |
| Postop destination |  |  |  |  |  |  |  |
|  | Ward (level 0 or 1 care) | 83.4 (81.8-85) % | 1776/2129 | 80.5 (76.5-84.6) % | 277/344 | 84 (82.3-85.7) % | 1499/1785 |
|  | Unplanned admission to PACU or equivalent (level 1.5 care) | 0.6 (0.3-0.9) % | 12/2129 | 0.9 (0-2) % | 3/344 | 0.5 (0.2-0.9) % | 9/1785 |
|  | Planned admission to PACU or equivalent (level 1.5 care) | 5.5 (4.6-6.5) % | 117/2129 | 2.9 (1.2-4.7) % | 10/344 | 6 (4.9-7.1) % | 107/1785 |
|  | Unplanned admission to PACU or equivalent (level 2/3 care) | 0.4 (0.1-0.7) % | 8/2129 | 1.5 (0.3-2.9) % | 5/344 | 0.2 (0-0.4) % | 3/1785 |
|  | Planned admission to PACU or equivalent (level 2/3 care) | 1.1 (0.7-1.6) % | 23/2129 | 1.7 (0.6-3.2) % | 6/344 | 1 (0.6-1.4) % | 17/1785 |
|  | Unplanned critical care admission (level 2 or 3 care) | 1.4 (0.9-1.9) % | 29/2129 | 2.9 (1.5-4.9) % | 10/344 | 1.1 (0.6-1.6) % | 19/1785 |
|  | Planned critical care admission (level 2 or 3 care) | 7.6 (6.4-8.7) % | 161/2129 | 9.6 (6.7-12.8) % | 33/344 | 7.2 (6-8.3) % | 128/1785 |
| Urinary catheter |  |  |  |  |  |  |  |
|  | No catheter | 54.1 (52-56.3) % | 1153/2130 | 38.8 (33.6-44.1) % | 134/345 | 57.1 (54.7-59.4) % | 1019/1785 |
|  | Long-term/pre-admission catheter | 3.1 (2.4-3.9) % | 66/2130 | 6.7 (4.1-9.3) % | 23/345 | 2.4 (1.7-3.1) % | 43/1785 |
|  | Electively catheterised pre/intra-op | 38 (35.9-40) % | 810/2130 | 50.1 (44.9-55.4) % | 173/345 | 35.7 (33.4-37.9) % | 637/1785 |
|  | Catheterised post-op | 2.3 (1.6-2.9) % | 48/2130 | 2.6 (0.9-4.6) % | 9/345 | 2.2 (1.6-2.9) % | 39/1785 |

#### Supplementary table 5G: Demographics of participants having inpatient surgery in the SNAP-3 cohort, with and without delirium

|  |  | **All** | | **Delirium** | | **All delirium** | |
| --- | --- | --- | --- | --- | --- | --- | --- |
| **Characteristic** |  | **Summary** | **Number** | **Summary** | **Number** | **Summary** | **Number** |
| Sex |  |  |  |  |  |  |  |
|  | Female | 48.5 (47-50) % | 2256/4649 | 52.8 (48.2-57.2) % | 252/477 | 48 (46.5-49.5) % | 2004/4172 |
|  | Male | 51.5 (50-53) % | 2393/4649 | 47.2 (42.6-51.4) % | 225/477 | 52 (50.5-53.4) % | 2168/4172 |
| Age |  |  |  |  |  |  |  |
|  | Age (years) | 73.8 (8.4) | 4646 | 79.1 (9.4) | 476 | 73.2 (7.8) | 4170 |
|  | 60-69 | 32.4 (31.1-33.8) % | 1506/4646 | 17.9 (14.5-21.4) % | 85/476 | 34.1 (32.6-35.5) % | 1421/4170 |
|  | 70-79 | 40.8 (39.4-42.2) % | 1895/4646 | 30 (26.1-34.2) % | 143/476 | 42 (40.4-43.5) % | 1752/4170 |
|  | 80-89 | 22.4 (21.2-23.6) % | 1039/4646 | 35.7 (31.3-39.9) % | 170/476 | 20.8 (19.6-22.1) % | 869/4170 |
|  | >90 | 4.4 (3.9-5) % | 206/4646 | 16.4 (13.2-19.7) % | 78/476 | 3.1 (2.6-3.6) % | 128/4170 |
| ASA |  |  |  |  |  |  |  |
|  | 1 | 5.3 (4.7-5.9) % | 243/4613 | 0.8 (0.2-1.7) % | 4/476 | 5.8 (5.1-6.5) % | 239/4137 |
|  | 2 | 47.9 (46.4-49.3) % | 2209/4613 | 21.8 (18.3-25.6) % | 104/476 | 50.9 (49.4-52.5) % | 2105/4137 |
|  | 3 | 41.6 (40.1-43) % | 1919/4613 | 60.3 (55.9-64.7) % | 287/476 | 39.4 (38-41) % | 1632/4137 |
|  | 4 | 5.1 (4.4-5.7) % | 234/4613 | 16.6 (13.4-20) % | 79/476 | 3.7 (3.2-4.4) % | 155/4137 |
|  | 5 | 0.2 (0.1-0.3) % | 8/4613 | 0.4 (0-1.1) % | 2/476 | 0.1 (0-0.3) % | 6/4137 |
| Frailty (CFS≥5) | Frail | 24.1 (22.8-25.4) % | 1112/4613 | 58.4 (54-62.9) % | 277/474 | 20.2 (18.9-21.3) % | 835/4139 |
| Clinical Frailty Scale |  |  |  |  |  |  |  |
|  | 1 | 9.1 (8.3-10) % | 419/4613 | 3.2 (1.7-4.9) % | 15/474 | 9.8 (8.9-10.7) % | 404/4139 |
|  | 2 | 16 (15-17.1) % | 739/4613 | 7.4 (5.1-9.9) % | 35/474 | 17 (15.8-18.2) % | 704/4139 |
|  | 3 | 29.1 (27.9-30.4) % | 1344/4613 | 14.3 (11.2-17.7) % | 68/474 | 30.8 (29.5-32.3) % | 1276/4139 |
|  | 4 | 21.7 (20.5-22.9) % | 999/4613 | 16.7 (13.5-20) % | 79/474 | 22.2 (21-23.5) % | 920/4139 |
|  | 5 | 12 (11.1-12.9) % | 553/4613 | 21.1 (17.5-24.7) % | 100/474 | 10.9 (10-11.9) % | 453/4139 |
|  | 6 | 7.3 (6.5-8) % | 336/4613 | 17.7 (14.3-21.3) % | 84/474 | 6.1 (5.4-6.8) % | 252/4139 |
|  | 7 | 4.3 (3.7-4.9) % | 198/4613 | 16.9 (13.7-20.3) % | 80/474 | 2.9 (2.3-3.4) % | 118/4139 |
|  | 8 | 0.5 (0.3-0.8) % | 25/4613 | 2.7 (1.5-4.2) % | 13/474 | 0.3 (0.1-0.5) % | 12/4139 |
|  | 9 |  |  |  |  |  |  |
| Multimorbidity (≥2 comorbidities) | Multimorbid | 66.8 (65.3-68.1) % | 2885/4322 | 79.9 (76.2-83.4) % | 366/458 | 65.2 (63.7-66.6) % | 2519/3864 |
| Number of comorbidities | Number of comorbidities | 2 (1-3) | 4322 | 3 (2-4) | 458 | 2 (1-3) | 3864 |
| Dementia | NA | 3.5 (3-4.1) % | 163/4650 | 21.2 (17.4-24.7) % | 101/477 | 1.5 (1.2-1.9) % | 62/4173 |
|  |  |  |  |  |  |  |  |
| Consent | Capacity | 95.3 (94.6-95.8) % | 4499/4723 | 70.6 (66.7-74.6) % | 337/477 | 98 (97.6-98.4) % | 4162/4246 |
| Consent | Consultee | 4.7 (4.1-5.4) % | 224/4723 | 29.4 (25.2-33.5) % | 140/477 | 2 (1.6-2.4) % | 84/4246 |
| Surgical specialty |  |  |  |  |  |  |  |
|  | Bariatric | 0.2 (0.1-0.3) % | 9/4601 | NA | NA | 0.2 (0.1-0.4) % | 9/4127 |
|  | Breast | 2.5 (2-2.9) % | 114/4601 | 0.2 (0-0.6) % | 1/474 | 2.7 (2.3-3.2) % | 113/4127 |
|  | Cardiology | 0 (0-0.1) % | 2/4601 | NA | NA | 0 (0-0.1) % | 2/4127 |
|  | Colorectal | 12.8 (11.8-13.7) % | 587/4601 | 9.5 (7-12) % | 45/474 | 13.1 (12.1-14.2) % | 542/4127 |
|  | Ear, nose & throat | 1.5 (1.2-1.9) % | 71/4601 | 0.4 (0-1.1) % | 2/474 | 1.7 (1.3-2) % | 69/4127 |
|  | Endocrine | 1.1 (0.8-1.4) % | 51/4601 | 0.2 (0-0.6) % | 1/474 | 1.2 (0.9-1.6) % | 50/4127 |
|  | Endoscopic | 0.3 (0.2-0.5) % | 14/4601 | 0.2 (0-0.6) % | 1/474 | 0.3 (0.1-0.5) % | 13/4127 |
|  | Gynaecology | 4.3 (3.7-4.9) % | 199/4601 | 0.8 (0.2-1.9) % | 4/474 | 4.7 (4.1-5.4) % | 195/4127 |
|  | Hepato-pancreato-biliary | 0.9 (0.7-1.2) % | 42/4601 | 1.9 (0.8-3.2) % | 9/474 | 0.8 (0.6-1.1) % | 33/4127 |
|  | Interventional radiology | 1.2 (0.9-1.5) % | 54/4601 | 1.9 (0.8-3.2) % | 9/474 | 1.1 (0.8-1.4) % | 45/4127 |
|  | Maxillo-facial and dental | 1.1 (0.8-1.4) % | 51/4601 | 0.8 (0.2-1.7) % | 4/474 | 1.1 (0.8-1.5) % | 47/4127 |
|  | Neurosurgery | 1.4 (1.1-1.7) % | 64/4601 | 2.5 (1.3-4) % | 12/474 | 1.3 (0.9-1.6) % | 52/4127 |
|  | Ophthalmic | 0.3 (0.1-0.4) % | 12/4601 | NA | NA | 0.3 (0.1-0.5) % | 12/4127 |
|  | Orthopaedics | 37 (35.7-38.5) % | 1703/4601 | 56.5 (51.9-61) % | 268/474 | 34.8 (33.3-36.2) % | 1435/4127 |
|  | Plastics | 2.7 (2.2-3.2) % | 124/4601 | 2.3 (1.1-3.8) % | 11/474 | 2.7 (2.3-3.2) % | 113/4127 |
|  | Spine | 2.8 (2.3-3.3) % | 129/4601 | 2.5 (1.3-4) % | 12/474 | 2.8 (2.3-3.3) % | 117/4127 |
|  | Thoracic | 6.1 (5.4-6.8) % | 281/4601 | 8.2 (5.7-10.8) % | 39/474 | 5.9 (5.2-6.6) % | 242/4127 |
|  | Transplant | 0.1 (0-0.2) % | 3/4601 | 0.2 (0-0.6) % | 1/474 | 0 (0-0.1) % | 2/4127 |
|  | Upper gastrointestinal | 5.3 (4.7-6) % | 244/4601 | 4.2 (2.5-6.1) % | 20/474 | 5.4 (4.7-6.1) % | 224/4127 |
|  | Urology | 15.3 (14.3-16.4) % | 705/4601 | 5.1 (3.4-7.2) % | 24/474 | 16.5 (15.3-17.6) % | 681/4127 |
|  | Vascular | 3 (2.6-3.5) % | 140/4601 | 2.3 (1.1-3.8) % | 11/474 | 3.1 (2.6-3.7) % | 129/4127 |
|  | Unknown | 0 (0-0.1) % | 2/4601 | NA | NA | 0 (0-0.1) % | 2/4127 |
| Surgical urgency |  |  |  |  |  |  |  |
|  | Emergency | 3.2 (2.7-3.7) % | 148/4650 | 6.3 (4.2-8.6) % | 30/477 | 2.8 (2.3-3.4) % | 118/4173 |
|  | Urgent | 20.2 (19.1-21.3) % | 941/4650 | 44.9 (40.5-49.3) % | 214/477 | 17.4 (16.3-18.6) % | 727/4173 |
|  | Expedited | 14.4 (13.4-15.5) % | 671/4650 | 20.8 (17.2-24.3) % | 99/477 | 13.7 (12.7-14.8) % | 572/4173 |
|  | Planned | 62.2 (60.8-63.6) % | 2890/4650 | 28.1 (24.1-32.3) % | 134/477 | 66 (64.6-67.6) % | 2756/4173 |
| Source of admission |  |  |  |  |  |  |  |
|  | Own home | 93.8 (93.1-94.6) % | 4361/4647 | 82.2 (78.6-85.5) % | 392/477 | 95.2 (94.5-95.8) % | 3969/4170 |
|  | Sheltered housing | 1.7 (1.3-2.1) % | 78/4647 | 1.9 (0.8-3.1) % | 9/477 | 1.7 (1.3-2) % | 69/4170 |
|  | Residential or nursing home | 2.1 (1.7-2.5) % | 97/4647 | 11.9 (9-14.9) % | 57/477 | 1 (0.7-1.3) % | 40/4170 |
|  | Rehabilitation facility | 0.2 (0.1-0.3) % | 8/4647 | 0.6 (0-1.5) % | 3/477 | 0.1 (0-0.2) % | 5/4170 |
|  | Another secondary care hospital | 0.9 (0.7-1.2) % | 44/4647 | 2.1 (0.8-3.4) % | 10/477 | 0.8 (0.6-1.1) % | 34/4170 |
|  | Other | 0.9 (0.7-1.2) % | 44/4647 | 1.3 (0.4-2.3) % | 6/477 | 0.9 (0.6-1.2) % | 38/4170 |
|  | Unknown | 0.3 (0.2-0.5) % | 15/4647 | NA | NA | 0.4 (0.2-0.6) % | 15/4170 |
| Independence |  |  |  |  |  |  |  |
|  | Independent | 67.7 (66.3-69.1) % | 3032/4479 | 38.7 (34-43.4) % | 164/424 | 70.7 (69.3-72.1) % | 2868/4055 |
|  | Assistance with instrumental ADLs | 28 (26.7-29.3) % | 1255/4479 | 46.7 (41.7-51.6) % | 198/424 | 26.1 (24.7-27.4) % | 1057/4055 |
|  | Assistance with basic ADLs | 4.3 (3.8-4.9) % | 192/4479 | 14.6 (11.3-17.9) % | 62/424 | 3.2 (2.7-3.7) % | 130/4055 |
| Ethnicity |  |  |  |  |  |  |  |
|  | Asian | 1.8 (1.4-2.2) % | 82/4597 | 1.9 (0.8-3.2) % | 9/471 | 1.8 (1.4-2.2) % | 73/4126 |
|  | Black | 1.3 (1-1.7) % | 62/4597 | 1.5 (0.4-2.8) % | 7/471 | 1.3 (1-1.7) % | 55/4126 |
|  | Mixed | 0.5 (0.3-0.7) % | 22/4597 | 0.2 (0-0.6) % | 1/471 | 0.5 (0.3-0.8) % | 21/4126 |
|  | White | 96.4 (95.8-96.9) % | 4431/4597 | 96.4 (94.7-97.9) % | 454/471 | 96.4 (95.8-96.9) % | 3977/4126 |
| Education |  |  |  |  |  |  |  |
|  | Degree level | 21.4 (20.2-22.6) % | 993/4645 | 15.8 (12.4-19.1) % | 75/476 | 22 (20.7-23.3) % | 918/4169 |
|  | A levels / NVQ 3 | 11.9 (11.1-12.9) % | 555/4645 | 9.7 (6.9-12.4) % | 46/476 | 12.2 (11.2-13.2) % | 509/4169 |
|  | Apprenticeship | 6.4 (5.7-7.1) % | 299/4645 | 6.5 (4.4-8.8) % | 31/476 | 6.4 (5.7-7.2) % | 268/4169 |
|  | GCSEs / NVQ 2 | 13.7 (12.7-14.7) % | 636/4645 | 11.1 (8.4-14.1) % | 53/476 | 14 (12.9-15.1) % | 583/4169 |
|  | O level / NVQ 1 | 12.2 (11.2-13.2) % | 567/4645 | 10.5 (7.8-13) % | 50/476 | 12.4 (11.5-13.4) % | 517/4169 |
|  | No formal qualifications | 23.9 (22.7-25.1) % | 1111/4645 | 26.9 (22.9-30.9) % | 128/476 | 23.6 (22.2-24.9) % | 983/4169 |
|  | Education not recorded | 10.4 (9.6-11.3) % | 484/4645 | 19.5 (16-23.1) % | 93/476 | 9.4 (8.5-10.3) % | 391/4169 |
| IMD deciles |  |  |  |  |  |  |  |
|  | 1 (most deprived) | 7 (6.4-7.8) % | 319/4526 | 8.6 (6.1-11.2) % | 39/456 | 6.9 (6.1-7.7) % | 280/4070 |
|  | 2 | 7.4 (6.6-8.2) % | 334/4526 | 7.2 (4.8-9.6) % | 33/456 | 7.4 (6.6-8.2) % | 301/4070 |
|  | 3 | 8.2 (7.4-9) % | 370/4526 | 7.9 (5.5-10.5) % | 36/456 | 8.2 (7.4-9) % | 334/4070 |
|  | 4 | 8.9 (8-9.7) % | 401/4526 | 9.4 (6.8-12.1) % | 43/456 | 8.8 (7.9-9.7) % | 358/4070 |
|  | 5 | 9.8 (9-10.7) % | 444/4526 | 8.8 (6.4-11.4) % | 40/456 | 9.9 (9-10.9) % | 404/4070 |
|  | 6 | 12.4 (11.5-13.4) % | 561/4526 | 12.9 (9.9-16) % | 59/456 | 12.3 (11.4-13.4) % | 502/4070 |
|  | 7 | 12 (11-12.9) % | 543/4526 | 12.1 (9-14.9) % | 55/456 | 12 (11-13) % | 488/4070 |
|  | 8 | 11.3 (10.4-12.2) % | 512/4526 | 12.3 (9.2-15.4) % | 56/456 | 11.2 (10.3-12.2) % | 456/4070 |
|  | 9 | 12.5 (11.5-13.5) % | 564/4526 | 11.8 (9-14.9) % | 54/456 | 12.5 (11.5-13.6) % | 510/4070 |
|  | 10 (least deprived) | 10.6 (9.6-11.5) % | 478/4526 | 9 (6.6-11.6) % | 41/456 | 10.7 (9.8-11.7) % | 437/4070 |
| Operative severity |  |  |  |  |  |  |  |
|  | Minor | 2.9 (2.5-3.4) % | 134/4600 | 2.1 (0.8-3.4) % | 10/474 | 3 (2.5-3.5) % | 124/4126 |
|  | Intermediate | 14 (13-15) % | 643/4600 | 8.9 (6.3-11.6) % | 42/474 | 14.6 (13.6-15.6) % | 601/4126 |
|  | Major | 32.5 (31.1-33.7) % | 1493/4600 | 34.6 (30.4-39) % | 164/474 | 32.2 (30.8-33.7) % | 1329/4126 |
|  | X Major | 32.8 (31.5-34.2) % | 1508/4600 | 35 (30.8-39.2) % | 166/474 | 32.5 (31.2-33.9) % | 1342/4126 |
|  | Complex | 17.8 (16.7-18.9) % | 820/4600 | 19.4 (16-23) % | 92/474 | 17.6 (16.5-18.8) % | 728/4126 |
|  | Unknown | 0 (0-0.1) % | 2/4600 | NA | NA | 0 (0-0.1) % | 2/4126 |
| Preop assessment |  |  |  |  |  |  |  |
|  | Nurse or AHP led clinic | 31.2 (29.9-32.5) % | 1393/4465 | 15.6 (12.4-19) % | 69/443 | 32.9 (31.5-34.4) % | 1324/4022 |
|  | Anaesthetist led clinic | 7.9 (7.1-8.7) % | 354/4465 | 5.2 (3.2-7.4) % | 23/443 | 8.2 (7.4-9.1) % | 331/4022 |
|  | Anaesthetist and geriatrician led clinics | 0.3 (0.1-0.4) % | 13/4465 | 0.2 (0-0.7) % | 1/443 | 0.3 (0.1-0.5) % | 12/4022 |
|  | Geriatrician led or MDT clinic | 1.1 (0.8-1.4) % | 49/4465 | 1.1 (0.2-2.3) % | 5/443 | 1.1 (0.8-1.4) % | 44/4022 |
|  | Physician (non geriatrician) led clinic | 1.1 (0.8-1.4) % | 48/4465 | 0.9 (0.2-2) % | 4/443 | 1.1 (0.8-1.4) % | 44/4022 |
|  | On the day assessment | 58.4 (56.9-59.9) % | 2608/4465 | 77 (72.7-80.8) % | 341/443 | 56.4 (54.8-57.9) % | 2267/4022 |
| Postop destination |  |  |  |  |  |  |  |
|  | Ward (level 0 or 1 care) | 80.9 (79.8-82) % | 3752/4637 | 73.5 (69.3-77.5) % | 350/476 | 81.8 (80.6-82.9) % | 3402/4161 |
|  | Unplanned admission to PACU or equivalent (level 1.5 care) | 0.7 (0.5-1) % | 34/4637 | 1.1 (0.2-2.1) % | 5/476 | 0.7 (0.5-1) % | 29/4161 |
|  | Planned admission to PACU or equivalent (level 1.5 care) | 7.1 (6.3-7.8) % | 327/4637 | 5.5 (3.6-7.6) % | 26/476 | 7.2 (6.5-8.1) % | 301/4161 |
|  | Unplanned admission to PACU or equivalent (level 2/3 care) | 0.3 (0.1-0.4) % | 12/4637 | 1.1 (0.2-2.1) % | 5/476 | 0.2 (0-0.3) % | 7/4161 |
|  | Planned admission to PACU or equivalent (level 2/3 care) | 2.2 (1.7-2.6) % | 100/4637 | 2.7 (1.3-4.4) % | 13/476 | 2.1 (1.7-2.5) % | 87/4161 |
|  | Unplanned critical care admission (level 2 or 3 care) | 1.1 (0.8-1.4) % | 51/4637 | 3.4 (1.9-5) % | 16/476 | 0.8 (0.6-1.1) % | 35/4161 |
|  | Planned critical care admission (level 2 or 3 care) | 7.6 (6.9-8.4) % | 354/4637 | 12.8 (9.9-16) % | 61/476 | 7 (6.3-7.9) % | 293/4161 |
| Urinary catheter |  |  |  |  |  |  |  |
|  | No catheter | 51 (49.5-52.5) % | 2368/4639 | 36.3 (32.1-40.7) % | 173/477 | 52.7 (51.2-54.3) % | 2195/4162 |
|  | Long-term/pre-admission catheter | 2.5 (2.1-3) % | 118/4639 | 5.7 (3.8-7.8) % | 27/477 | 2.2 (1.7-2.6) % | 91/4162 |
|  | Electively catheterised pre/intra-op | 41.9 (40.5-43.3) % | 1942/4639 | 52.8 (48.2-57.2) % | 252/477 | 40.6 (39.1-42.1) % | 1690/4162 |
|  | Catheterised post-op | 2.6 (2.2-3.1) % | 121/4639 | 3.8 (2.1-5.5) % | 18/477 | 2.5 (2-3) % | 103/4162 |

### Clinical characteristics: subgroups

Percentages have been rounded so may not total 100% exactly. Missing data are omitted from this table but reported in Supplementary table 4. Surgical urgency is defined using NCEPOD categorisations. The values here are proportions with 95% confidence intervals (95% CI) unless stated. 95% CI are calculated using the percentile method and 2000 bootstraps. SORT: Surgical Outcome Risk Tool; BMI: Body Mass Index (kg.m^-2^); COPD: Chronic obstructive pulmonary disease; WCC: white cell (leucocyte) count; eGFR: estimated glomerular filtration rate

#### Supplementary table 6A: Clinical characteristics of participants aged ≥85 in the SNAP-3 cohort, with and without delirium

|  |  | **All** | | **Delirium** | | **No delirium** | |
| --- | --- | --- | --- | --- | --- | --- | --- |
| **Characteristic** |  | **Summary** | **Number** | **Summary** | **Number** | **Summary** | **Number** |
| Number of comorbidities | Total comorbidities | 3 (2-4) | 694 | 3 (2-4) | 158 | 3 (2-4) | 536 |
| SORT predicted morbidity (%) | Predicted morbidity | 32 (30.8-33.3) | 740 | 32.6 (30.2-35.3) | 162 | 31.8 (30.4-33.4) | 578 |
| SORT predicted mortality (%) | Predicted mortality | 6.1 (5.5-6.7) | 745 | 10.4 (8.9-12.1) | 163 | 4.9 (4.3-5.5) | 582 |
| Polypharmacy |  | 61.8 (58.3-65.2) % | 455/736 | 68.6 (61-75.5) % | 109/159 | 60 (56-64) % | 346/577 |
| BMI category |  |  |  |  |  |  |  |
|  | Underweight | 5.3 (3.8-6.9) % | 39/738 | 10 (5-15) % | 16/160 | 4 (2.4-5.5) % | 23/578 |
|  | Healthy weight | 48.5 (44.9-52.2) % | 358/738 | 58.8 (51.2-66.9) % | 94/160 | 45.7 (41.7-49.7) % | 264/578 |
|  | Overweight | 31.6 (28.3-35) % | 233/738 | 22.5 (16.2-29.4) % | 36/160 | 34.1 (30.3-38.1) % | 197/578 |
|  | Obese class 1 | 14 (11.4-16.4) % | 103/738 | 8.8 (5-13.1) % | 14/160 | 15.4 (12.6-18.5) % | 89/578 |
|  | Obese class >=2 | 0.7 (0.1-1.4) % | 5/738 | NA | NA | 0.9 (0.2-1.7) % | 5/578 |
| Hearing impairment |  | 20.1 (17.3-23.2) % | 150/745 | 19 (12.9-25.2) % | 31/163 | 20.4 (17.2-23.9) % | 119/582 |
| Visual impairment |  | 6.2 (4.4-7.9) % | 46/745 | 9.8 (5.5-14.7) % | 16/163 | 5.2 (3.4-7) % | 30/582 |
| Dementia |  | 13.6 (11.3-16) % | 101/745 | 37.4 (30.1-45.4) % | 61/163 | 6.9 (4.8-8.9) % | 40/582 |
| Myocardial infarction |  | 14.1 (11.7-16.6) % | 105/745 | 12.3 (7.4-17.2) % | 20/163 | 14.6 (11.7-17.5) % | 85/582 |
| Heart failure |  | 12.5 (10.2-15) % | 93/745 | 17.8 (12.3-23.9) % | 29/163 | 11 (8.6-13.7) % | 64/582 |
| Atrial fibrillation |  | 27 (23.8-30.1) % | 201/745 | 32.5 (25.2-39.3) % | 53/163 | 25.4 (22.2-28.9) % | 148/582 |
| Valvular heart disease |  | 9.7 (7.5-11.9) % | 72/745 | 11 (6.7-16) % | 18/163 | 9.3 (7-11.7) % | 54/582 |
| Hypertension |  | 60 (56.4-63.6) % | 447/745 | 55.8 (48.5-63.8) % | 91/163 | 61.2 (57.2-65.3) % | 356/582 |
| Peripheral vascular disease |  | 4.2 (2.8-5.6) % | 31/745 | 3.7 (1.2-6.7) % | 6/163 | 4.3 (2.7-6.2) % | 25/582 |
| COPD |  | 10.5 (8.3-12.8) % | 78/745 | 8 (3.7-12.3) % | 13/163 | 11.2 (8.6-13.9) % | 65/582 |
| Chronic lung disease |  | 8.3 (6.4-10.3) % | 62/745 | 7.4 (3.7-11.7) % | 12/163 | 8.6 (6.4-10.8) % | 50/582 |
| OSA |  | 1.3 (0.5-2.3) % | 10/745 | NA | NA | 1.7 (0.7-2.9) % | 10/582 |
| Stroke |  | 13.8 (11.4-16.4) % | 103/745 | 16 (10.4-21.5) % | 26/163 | 13.2 (10.5-16) % | 77/582 |
| Hemiplegia |  | 0.8 (0.3-1.5) % | 6/745 | 0.6 (0-1.8) % | 1/163 | 0.9 (0.2-1.7) % | 5/582 |
| Anxiety / depression |  | 5 (3.5-6.6) % | 37/745 | 10.4 (6.1-15.3) % | 17/163 | 3.4 (2.1-5.2) % | 20/582 |
| Parkinson's disease |  | 1.2 (0.5-2) % | 9/745 | 3.7 (1.2-6.7) % | 6/163 | 0.5 (0-1.2) % | 3/582 |
| Diabetes |  |  |  |  |  |  |  |
|  | Present | 15.3 (12.9-18) % | 114/745 | 18.4 (12.9-24.5) % | 30/163 | 14.4 (11.5-17.4) % | 84/582 |
|  | Diabetes without chronic complications | 72.8 (64-80.7) % | 83/114 | 63.3 (46.7-80) % | 19/30 | 76.2 (67.9-85.7) % | 64/84 |
|  | Diabetes with chronic complications | 27.2 (19.3-36) % | 31/114 | 36.7 (20-56.7) % | 11/30 | 23.8 (15.5-33.3) % | 20/84 |
| Chronic kidney disease |  | 22.7 (19.7-25.9) % | 169/745 | 25.2 (18.4-31.9) % | 41/163 | 22 (18.7-25.6) % | 128/582 |
| Benign prostatic hyperplasia |  | 7.8 (5.9-9.8) % | 58/745 | 4.3 (1.2-8) % | 7/163 | 8.8 (6.5-11.2) % | 51/582 |
| Liver disease |  |  |  |  |  |  |  |
|  | Present | 0.8 (0.3-1.5) % | 6/745 | 1.8 (0-4.3) % | 3/163 | 0.5 (0-1.2) % | 3/582 |
|  | Mild liver disease | 0.8 (0.3-1.5) % | 6/745 | 1.8 (0-4.3) % | 3/163 | 0.5 (0-1.2) % | 3/582 |
| Peptic ulcer disease |  | 4.3 (3-5.8) % | 32/745 | 6.1 (3.1-10.4) % | 10/163 | 3.8 (2.2-5.5) % | 22/582 |
| Lymphoma |  | 0.7 (0.1-1.3) % | 5/745 | 0.6 (0-1.8) % | 1/163 | 0.7 (0.2-1.4) % | 4/582 |
| Leukaemia |  | 0.7 (0.1-1.3) % | 5/745 | 0.6 (0-1.8) % | 1/163 | 0.7 (0.2-1.4) % | 4/582 |
| Connective tissue disorders |  | 5 (3.5-6.6) % | 37/745 | 3.1 (0.6-6.1) % | 5/163 | 5.5 (3.6-7.4) % | 32/582 |
| Osteoarthritis |  | 36.4 (32.9-39.9) % | 271/745 | 32.5 (25.2-39.3) % | 53/163 | 37.5 (33.5-41.2) % | 218/582 |
| Malignancy |  |  |  |  |  |  |  |
|  | Present | 23 (19.9-26) % | 171/745 | 12.3 (7.4-17.8) % | 20/163 | 25.9 (22.3-29.6) % | 151/582 |
|  | Malignancy without metastasis | 18.9 (16.1-21.7) % | 141/745 | 9.2 (4.9-14.1) % | 15/163 | 21.6 (18.6-24.9) % | 126/582 |
|  | Malignancy with metastasis | 4 (2.7-5.4) % | 30/745 | 3.1 (0.6-6.1) % | 5/163 | 4.3 (2.7-6) % | 25/582 |
|  | Malignancy diagnosis ≤ 5 years | 14.6 (12.1-17.2) % | 109/745 | 7.4 (3.7-11.7) % | 12/163 | 16.7 (13.7-19.8) % | 97/582 |
|  | Malignancy diagnosis > 5 years | 8.6 (6.6-10.6) % | 64/745 | 4.9 (1.8-8.6) % | 8/163 | 9.6 (7.4-12.2) % | 56/582 |
| Haematology |  |  |  |  |  |  |  |
|  | Haemoglobin (g/l) | 121.8 (120.4-123.1) | 703 | 119.3 (116.3-122.2) | 163 | 122.5 (121-124) | 540 |
|  | WCC (x10^9/L) | 9.4 (9.1-9.8) | 703 | 10.7 (10.1-11.3) | 163 | 9.1 (8.7-9.5) | 540 |
|  | Neutrophil count (x10^9/L) | 7 (6.8-7.3) | 700 | 8.6 (8-9.2) | 161 | 6.6 (6.3-6.8) | 539 |
| Biochemistry |  |  |  |  |  |  |  |
|  | eGFR (ml/min) | 61 (45.5-76) | 694 | 61 (44-77) | 158 | 61 (46-75) | 536 |
|  | Sodium (mmol/l) | 138 (136-141) | 707 | 138 (135-140) | 162 | 139 (136-141) | 545 |
|  | Potassium (mmol/l) | 4.4 (4.3-4.4) | 705 | 4.3 (4.2-4.4) | 161 | 4.4 (4.3-4.4) | 544 |
|  | Creatinine (micromol/l) | 85 (68-110) | 706 | 82 (63-106.5) | 163 | 86 (69-111) | 543 |

#### Supplementary table 6B: Clinical characteristics of participants aged <85 in the SNAP-3 cohort, with and without delirium

|  |  | **All** | | **Delirium** | | **No delirium** | |
| --- | --- | --- | --- | --- | --- | --- | --- |
| **Characteristic** |  | **Summary** | **Number** | **Summary** | **Number** | **Summary** | **Number** |
| Number of comorbidities | Total comorbidities | 2 (1-3) | 5601 | 3 (2-4) | 302 | 2 (1-3) | 5299 |
| SORT predicted morbidity (%) | Predicted morbidity | 21.6 (21.2-22.1) | 6213 | 31.1 (29-33.4) | 313 | 21.1 (20.7-21.6) | 5900 |
| SORT predicted mortality (%) | Predicted mortality | 1.4 (1.3-1.4) | 6294 | 4.2 (3.6-4.9) | 315 | 1.2 (1.2-1.3) | 5979 |
| Polypharmacy |  | 45.2 (44-46.5) % | 2825/6247 | 63.8 (58.3-68.9) % | 199/312 | 44.2 (43-45.6) % | 2626/5935 |
| BMI category |  |  |  |  |  |  |  |
|  | Underweight | 1.6 (1.2-1.9) % | 97/6253 | 4.8 (2.6-7.4) % | 15/311 | 1.4 (1.1-1.7) % | 82/5942 |
|  | Healthy weight | 27.7 (26.6-28.9) % | 1733/6253 | 36 (30.9-40.8) % | 112/311 | 27.3 (26.1-28.4) % | 1621/5942 |
|  | Overweight | 36.5 (35.3-37.7) % | 2281/6253 | 33.1 (28.3-38.6) % | 103/311 | 36.7 (35.4-37.9) % | 2178/5942 |
|  | Obese class 1 | 30.3 (29.2-31.5) % | 1896/6253 | 24.1 (19.3-28.6) % | 75/311 | 30.6 (29.4-31.8) % | 1821/5942 |
|  | Obese class >=2 | 3.9 (3.5-4.4) % | 246/6253 | 1.9 (0.6-3.5) % | 6/311 | 4 (3.5-4.5) % | 240/5942 |
| Hearing impairment |  | 7.1 (6.5-7.7) % | 447/6306 | 11.1 (7.6-14.9) % | 35/315 | 6.9 (6.2-7.5) % | 412/5991 |
| Visual impairment |  | 2.2 (1.9-2.6) % | 140/6306 | 4.8 (2.5-7.3) % | 15/315 | 2.1 (1.7-2.5) % | 125/5991 |
| Dementia |  | 1.3 (1-1.6) % | 83/6306 | 12.7 (9.2-16.5) % | 40/315 | 0.7 (0.5-1) % | 43/5991 |
| Myocardial infarction |  | 9.2 (8.5-9.9) % | 577/6306 | 12.1 (8.6-15.9) % | 38/315 | 9 (8.3-9.7) % | 539/5991 |
| Heart failure |  | 4.1 (3.6-4.6) % | 259/6306 | 7.3 (4.8-10.2) % | 23/315 | 3.9 (3.4-4.5) % | 236/5991 |
| Atrial fibrillation |  | 9.1 (8.4-9.8) % | 574/6306 | 18.1 (13.7-22.2) % | 57/315 | 8.6 (7.9-9.4) % | 517/5991 |
| Valvular heart disease |  | 3.9 (3.4-4.3) % | 243/6306 | 7 (4.4-9.8) % | 22/315 | 3.7 (3.2-4.2) % | 221/5991 |
| Hypertension |  | 47.4 (46.1-48.6) % | 2988/6306 | 54.3 (48.6-60) % | 171/315 | 47 (45.8-48.3) % | 2817/5991 |
| Peripheral vascular disease |  | 4.7 (4.2-5.2) % | 295/6306 | 8.3 (5.4-11.1) % | 26/315 | 4.5 (4-5) % | 269/5991 |
| COPD |  | 9.4 (8.7-10.2) % | 595/6306 | 14 (10.2-17.8) % | 44/315 | 9.2 (8.5-9.9) % | 551/5991 |
| Chronic lung disease |  | 8.8 (8.1-9.5) % | 554/6306 | 9.8 (6.7-13.3) % | 31/315 | 8.7 (8-9.4) % | 523/5991 |
| OSA |  | 3.3 (2.8-3.7) % | 205/6306 | 1.6 (0.3-3.2) % | 5/315 | 3.3 (2.9-3.8) % | 200/5991 |
| Stroke |  | 7.4 (6.7-8) % | 464/6306 | 15.2 (11.4-19.7) % | 48/315 | 6.9 (6.3-7.6) % | 416/5991 |
| Hemiplegia |  | 0.7 (0.5-1) % | 47/6306 | 4.1 (2.2-6.3) % | 13/315 | 0.6 (0.4-0.8) % | 34/5991 |
| Anxiety / depression |  | 10.5 (9.7-11.2) % | 659/6306 | 17.5 (13.3-21.9) % | 55/315 | 10.1 (9.3-10.8) % | 604/5991 |
| Parkinson's disease |  | 0.9 (0.7-1.1) % | 56/6306 | 1.9 (0.6-3.5) % | 6/315 | 0.8 (0.6-1.1) % | 50/5991 |
| Diabetes |  |  |  |  |  |  |  |
|  | Present | 16.5 (15.6-17.4) % | 1039/6306 | 23.2 (18.4-27.6) % | 73/315 | 16.1 (15.2-17) % | 966/5991 |
|  | Diabetes without chronic complications | 70.7 (67.8-73.6) % | 734/1038 | 64.4 (53.4-75.3) % | 47/73 | 71.2 (68.2-74) % | 687/965 |
|  | Diabetes with chronic complications | 29.3 (26.7-32.2) % | 304/1038 | 35.6 (24.7-47.9) % | 26/73 | 28.8 (26-31.6) % | 278/965 |
| Chronic kidney disease |  | 8.4 (7.7-9.1) % | 527/6306 | 11.4 (8.3-14.9) % | 36/315 | 8.2 (7.5-8.9) % | 491/5991 |
| Benign prostatic hyperplasia |  | 6.8 (6.2-7.4) % | 428/6306 | 5.4 (2.9-7.9) % | 17/315 | 6.9 (6.3-7.5) % | 411/5991 |
| Liver disease |  |  |  |  |  |  |  |
|  | Present | 1.8 (1.5-2.2) % | 116/6306 | 1.6 (0.3-3.2) % | 5/315 | 1.9 (1.5-2.2) % | 111/5991 |
|  | Mild liver disease | 1.6 (1.3-1.9) % | 101/6306 | 1.3 (0.3-2.5) % | 4/315 | 1.6 (1.3-2) % | 97/5991 |
|  | Moderate or severe liver disease | 0.2 (0.1-0.3) % | 13/6306 | 0.3 (0-1) % | 1/315 | 0.2 (0.1-0.3) % | 12/5991 |
| Peptic ulcer disease |  | 3.4 (2.9-3.8) % | 212/6306 | 2.5 (1-4.4) % | 8/315 | 3.4 (3-3.9) % | 204/5991 |
| Lymphoma |  | 1 (0.8-1.3) % | 64/6306 | 1.9 (0.6-3.5) % | 6/315 | 1 (0.7-1.2) % | 58/5991 |
| Leukaemia |  | 0.6 (0.4-0.8) % | 36/6306 | 0.6 (0-1.6) % | 2/315 | 0.6 (0.4-0.8) % | 34/5991 |
| Connective tissue disorders |  | 4.8 (4.3-5.3) % | 303/6306 | 3.5 (1.6-5.7) % | 11/315 | 4.9 (4.3-5.4) % | 292/5991 |
| Osteoarthritis |  | 28.4 (27.3-29.5) % | 1792/6306 | 27.6 (22.5-32.7) % | 87/315 | 28.5 (27.3-29.6) % | 1705/5991 |
| Malignancy |  |  |  |  |  |  |  |
|  | Present | 20.8 (19.9-21.9) % | 1314/6306 | 19.7 (15.2-24.4) % | 62/315 | 20.9 (19.9-21.9) % | 1252/5991 |
|  | Malignancy without metastasis | 17.4 (16.5-18.4) % | 1100/6306 | 15.6 (11.7-19.7) % | 49/315 | 17.5 (16.6-18.5) % | 1051/5991 |
|  | Malignancy with metastasis | 3.5 (3-3.9) % | 220/6306 | 4.1 (2.2-6.3) % | 13/315 | 3.5 (3-3.9) % | 207/5991 |
|  | Malignancy diagnosis ≤ 5 years | 16.6 (15.7-17.5) % | 1046/6306 | 15.6 (11.7-19.7) % | 49/315 | 16.6 (15.7-17.5) % | 997/5991 |
|  | Malignancy diagnosis > 5 years | 4.4 (3.9-5) % | 280/6306 | 4.8 (2.5-7.3) % | 15/315 | 4.4 (3.9-5) % | 265/5991 |
| Haematology |  |  |  |  |  |  |  |
|  | Haemoglobin (g/l) | 133.3 (132.9-133.8) | 5680 | 124.5 (122.1-126.9) | 305 | 133.8 (133.4-134.3) | 5375 |
|  | WCC (x10^9/L) | 8 (7.9-8.1) | 5680 | 10 (9.4-10.6) | 305 | 7.9 (7.8-8) | 5375 |
|  | Neutrophil count (x10^9/L) | 5.3 (5.3-5.4) | 5644 | 7.4 (7-7.9) | 303 | 5.2 (5.1-5.3) | 5341 |
| Biochemistry |  | 76 (61-89) | 5601 | 73 (60-89) | 302 | 76 (61-89) | 5299 |
|  | eGFR (ml/min) |  |  |  |  |  |  |
|  | Sodium (mmol/l) | 139 (137-141) | 5709 | 139 (136-141) | 305 | 139 (137-141) | 5404 |
|  | Potassium (mmol/l) | 4.4 (4.4-4.4) | 5681 | 4.2 (4.2-4.3) | 303 | 4.4 (4.4-4.4) | 5378 |
|  | Creatinine (micromol/l) | 77 (64-92) | 5701 | 77 (62-94) | 304 | 76 (64-92) | 5397 |

#### Supplementary table 6C: Clinical characteristics of participants living with frailty in the SNAP-3 cohort, with and without delirium

|  |  | **All** | | **Delirium** | | **No delirium** | |
| --- | --- | --- | --- | --- | --- | --- | --- |
| **Characteristic** |  | **Summary** | **Number** | **Summary** | **Number** | **Summary** | **Number** |
| Number of comorbidities | Total comorbidities | 3 (2-4) | 1251 | 3 (2-4) | 267 | 3 (2-4) | 984 |
| SORT predicted morbidity (%) | Predicted morbidity | 27.8 (26.9-28.6) | 1348 | 31 (28.9-33.1) | 277 | 26.9 (25.9-27.9) | 1071 |
| SORT predicted mortality (%) | Predicted mortality | 4.3 (3.9-4.6) | 1362 | 7.9 (7-8.8) | 277 | 3.4 (3-3.7) | 1085 |
| Polypharmacy |  | 75.6 (73.3-77.9) % | 1014/1342 | 72.6 (67.5-77.7) % | 199/274 | 76.3 (73.5-78.8) % | 815/1068 |
| BMI category |  |  |  |  |  |  |  |
|  | Underweight | 4.8 (3.7-6) % | 65/1345 | 10.3 (7-14) % | 28/272 | 3.4 (2.4-4.7) % | 37/1073 |
|  | Healthy weight | 35.7 (33.2-38.2) % | 480/1345 | 44.9 (38.6-50.4) % | 122/272 | 33.4 (30.6-36.3) % | 358/1073 |
|  | Overweight | 26.4 (24.2-28.8) % | 355/1345 | 24.3 (19.1-29) % | 66/272 | 26.9 (24.2-29.6) % | 289/1073 |
|  | Obese class 1 | 27.9 (25.5-30.3) % | 375/1345 | 19.1 (14.7-24.3) % | 52/272 | 30.1 (27.3-33) % | 323/1073 |
|  | Obese class >=2 | 5.2 (4.1-6.4) % | 70/1345 | 1.5 (0.4-2.9) % | 4/272 | 6.2 (4.8-7.5) % | 66/1073 |
| Hearing impairment |  | 13 (11.4-14.9) % | 178/1364 | 14.7 (10.8-19.1) % | 41/278 | 12.6 (10.8-14.6) % | 137/1086 |
| Visual impairment |  | 5.1 (3.9-6.2) % | 69/1364 | 7.9 (5-11.2) % | 22/278 | 4.3 (3.1-5.5) % | 47/1086 |
| Dementia |  | 12.2 (10.6-14) % | 166/1364 | 36 (30.6-41.7) % | 100/278 | 6.1 (4.8-7.6) % | 66/1086 |
| Myocardial infarction |  | 14.4 (12.5-16.4) % | 197/1364 | 12.2 (8.3-16.2) % | 34/278 | 15 (13.1-17.1) % | 163/1086 |
| Heart failure |  | 11.4 (9.8-13.3) % | 156/1364 | 14.4 (10.4-18.7) % | 40/278 | 10.7 (8.8-12.6) % | 116/1086 |
| Atrial fibrillation |  | 19.5 (17.4-21.6) % | 266/1364 | 23.7 (18.7-29.1) % | 66/278 | 18.4 (16.2-20.7) % | 200/1086 |
| Valvular heart disease |  | 7.3 (6-8.7) % | 100/1364 | 7.9 (5-11.5) % | 22/278 | 7.2 (5.6-8.7) % | 78/1086 |
| Hypertension |  | 58.7 (56-61.3) % | 801/1364 | 58.6 (52.9-64.4) % | 163/278 | 58.7 (55.8-61.7) % | 638/1086 |
| Peripheral vascular disease |  | 9.3 (7.8-10.9) % | 127/1364 | 9.4 (5.8-12.9) % | 26/278 | 9.3 (7.5-11.2) % | 101/1086 |
| COPD |  | 16.9 (15-18.9) % | 230/1364 | 11.5 (7.9-15.1) % | 32/278 | 18.2 (16-20.6) % | 198/1086 |
| Chronic lung disease |  | 10.3 (8.8-12) % | 141/1364 | 8.3 (5-11.9) % | 23/278 | 10.9 (9.1-12.8) % | 118/1086 |
| OSA |  | 4.3 (3.3-5.4) % | 58/1364 | 1.1 (0-2.5) % | 3/278 | 5.1 (3.8-6.4) % | 55/1086 |
| Stroke |  | 15 (13.1-17) % | 204/1364 | 16.9 (12.6-21.6) % | 47/278 | 14.5 (12.3-16.6) % | 157/1086 |
| Hemiplegia |  | 2.9 (2-3.7) % | 39/1364 | 4 (1.8-6.5) % | 11/278 | 2.6 (1.7-3.6) % | 28/1086 |
| Anxiety / depression |  | 16.4 (14.4-18.3) % | 224/1364 | 14.4 (10.8-18.7) % | 40/278 | 16.9 (14.7-19.1) % | 184/1086 |
| Parkinson's disease |  | 2.6 (1.8-3.5) % | 36/1364 | 3.2 (1.4-5.4) % | 9/278 | 2.5 (1.6-3.5) % | 27/1086 |
| Diabetes |  |  |  |  |  |  |  |
|  | Present | 24.4 (22.1-26.8) % | 333/1364 | 24.5 (19.4-29.9) % | 68/278 | 24.4 (22-27) % | 265/1086 |
|  | Diabetes without chronic complications | 57.7 (52.3-63.1) % | 192/333 | 54.4 (41.2-66.2) % | 37/68 | 58.5 (52.5-64.2) % | 155/265 |
|  | Diabetes with chronic complications | 42.3 (36.9-47.7) % | 141/333 | 45.6 (33.8-57.4) % | 31/68 | 41.5 (35.5-47.5) % | 110/265 |
| Chronic kidney disease |  | 20.2 (18-22.4) % | 275/1364 | 22.7 (17.6-27.7) % | 63/278 | 19.5 (17.1-21.9) % | 212/1086 |
| Benign prostatic hyperplasia |  | 6.7 (5.4-8) % | 91/1364 | 5.4 (2.9-8.3) % | 15/278 | 7 (5.6-8.5) % | 76/1086 |
| Liver disease |  |  |  |  |  |  |  |
|  | Present | 3 (2.1-4) % | 41/1364 | 2.9 (1.1-5) % | 8/278 | 3 (2-4.1) % | 33/1086 |
|  | Mild liver disease | 2.7 (1.9-3.6) % | 37/1364 | 2.5 (0.7-4.3) % | 7/278 | 2.8 (1.8-3.8) % | 30/1086 |
|  | Moderate or severe liver disease | 0.3 (0.1-0.6) % | 4/1364 | 0.4 (0-1.1) % | 1/278 | 0.3 (0-0.6) % | 3/1086 |
| Peptic ulcer disease |  | 4.1 (3.1-5.2) % | 56/1364 | 3.6 (1.4-5.8) % | 10/278 | 4.2 (3-5.4) % | 46/1086 |
| Lymphoma |  | 1.1 (0.6-1.7) % | 15/1364 | 1.1 (0-2.5) % | 3/278 | 1.1 (0.6-1.8) % | 12/1086 |
| Leukaemia |  | 0.6 (0.2-1) % | 8/1364 | 1.1 (0-2.5) % | 3/278 | 0.5 (0.1-0.9) % | 5/1086 |
| Connective tissue disorders |  | 8.3 (7-9.8) % | 113/1364 | 3.2 (1.4-5.4) % | 9/278 | 9.6 (7.9-11.3) % | 104/1086 |
| Osteoarthritis |  | 40.3 (37.6-43) % | 550/1364 | 33.8 (28.1-39.6) % | 94/278 | 42 (39-44.9) % | 456/1086 |
| Malignancy |  |  |  |  |  |  |  |
|  | Present | 19.1 (16.9-21.2) % | 260/1364 | 15.8 (11.9-20.1) % | 44/278 | 19.9 (17.4-22.3) % | 216/1086 |
|  | Malignancy without metastasis | 15.3 (13.5-17.2) % | 209/1364 | 11.9 (8.3-15.8) % | 33/278 | 16.2 (14-18.5) % | 176/1086 |
|  | Malignancy with metastasis | 3.7 (2.7-4.8) % | 51/1364 | 4 (1.8-6.5) % | 11/278 | 3.7 (2.6-4.9) % | 40/1086 |
|  | Malignancy diagnosis ≤ 5 years | 13.3 (11.4-15.1) % | 181/1364 | 10.8 (7.2-14.7) % | 30/278 | 13.9 (11.9-16) % | 151/1086 |
|  | Malignancy diagnosis > 5 years | 5.9 (4.7-7.2) % | 80/1364 | 5.4 (2.9-8.3) % | 15/278 | 6 (4.6-7.5) % | 65/1086 |
| Haematology |  |  |  |  |  |  |  |
|  | Haemoglobin (g/l) | 123 (121.9-124.1) | 1290 | 118.9 (116.5-121.2) | 273 | 124.2 (123-125.3) | 1017 |
|  | WCC (x10^9/L) | 9.1 (8.9-9.4) | 1290 | 10.6 (10.1-11.2) | 273 | 8.8 (8.5-9) | 1017 |
|  | Neutrophil count (x10^9/L) | 6.7 (6.5-6.9) | 1282 | 8.3 (7.8-8.7) | 270 | 6.3 (6.1-6.5) | 1012 |
| Biochemistry |  |  |  |  |  |  |  |
|  | eGFR (ml/min) | 68 (53-86) | 1251 | 67 (54-83) | 267 | 69 (53-86) | 984 |
|  | Sodium (mmol/l) | 139 (136-141) | 1287 | 138 (136-141) | 274 | 139 (136-141) | 1013 |
|  | Potassium (mmol/l) | 4.4 (4.3-4.4) | 1282 | 4.3 (4.2-4.3) | 272 | 4.4 (4.3-4.4) | 1010 |
|  | Creatinine (micromol/l) | 78 (62-100) | 1284 | 78 (60-100) | 273 | 78 (63-100) | 1011 |

#### Supplementary table 6D: Clinical characteristics of participants not frail in the SNAP-3 cohort, with and without delirium

|  |  | **All** | | **Delirium** | | **No delirium** | |
| --- | --- | --- | --- | --- | --- | --- | --- |
| **Characteristic** |  | **Summary** | **Number** | **Summary** | **Number** | **Summary** | **Number** |
| Number of comorbidities | Total comorbidities | 2 (1-3) | 4992 | 2 (1-3) | 190 | 2 (1-3) | 4802 |
| SORT predicted morbidity (%) | Predicted morbidity | 21.6 (21.1-22) | 5544 | 32.2 (29.4-35.1) | 195 | 21.2 (20.7-21.6) | 5349 |
| SORT predicted mortality (%) | Predicted mortality | 1.3 (1.2-1.4) | 5615 | 4.2 (3.2-5.6) | 198 | 1.2 (1.1-1.3) | 5417 |
| Polypharmacy |  | 40.1 (38.8-41.5) % | 2241/5584 | 55.4 (48.2-62.1) % | 108/195 | 39.6 (38.3-40.9) % | 2133/5389 |
| BMI category |  |  |  |  |  |  |  |
|  | Underweight | 1.3 (1-1.5) % | 70/5591 | 1 (0-2.5) % | 2/197 | 1.3 (1-1.6) % | 68/5394 |
|  | Healthy weight | 28.7 (27.5-29.9) % | 1603/5591 | 42.6 (36-49.7) % | 84/197 | 28.2 (26.9-29.4) % | 1519/5394 |
|  | Overweight | 38.2 (36.8-39.3) % | 2133/5591 | 36.5 (29.9-43.6) % | 72/197 | 38.2 (37-39.5) % | 2061/5394 |
|  | Obese class 1 | 28.7 (27.6-29.9) % | 1606/5591 | 18.8 (13.7-24.4) % | 37/197 | 29.1 (27.9-30.3) % | 1569/5394 |
|  | Obese class >=2 | 3.2 (2.7-3.7) % | 179/5591 | 1 (0-2.5) % | 2/197 | 3.3 (2.8-3.8) % | 177/5394 |
| Hearing impairment |  | 7.4 (6.7-8.1) % | 416/5628 | 12.6 (8.1-17.7) % | 25/198 | 7.2 (6.5-7.9) % | 391/5430 |
| Visual impairment |  | 2.1 (1.7-2.4) % | 116/5628 | 4 (1.5-7.1) % | 8/198 | 2 (1.6-2.4) % | 108/5430 |
| Dementia |  | 0.3 (0.2-0.4) % | 17/5628 | 0.5 (0-1.5) % | 1/198 | 0.3 (0.1-0.5) % | 16/5430 |
| Myocardial infarction |  | 8.5 (7.8-9.3) % | 480/5628 | 12.1 (7.6-16.7) % | 24/198 | 8.4 (7.7-9.2) % | 456/5430 |
| Heart failure |  | 3.4 (2.9-3.9) % | 191/5628 | 5.1 (2-8.1) % | 10/198 | 3.3 (2.9-3.8) % | 181/5430 |
| Atrial fibrillation |  | 8.9 (8.2-9.7) % | 503/5628 | 22.2 (16.7-28.3) % | 44/198 | 8.5 (7.7-9.2) % | 459/5430 |
| Valvular heart disease |  | 3.8 (3.3-4.3) % | 213/5628 | 9.1 (5.6-13.1) % | 18/198 | 3.6 (3.1-4.1) % | 195/5430 |
| Hypertension |  | 46.2 (44.9-47.5) % | 2600/5628 | 50 (42.9-57.1) % | 99/198 | 46.1 (44.8-47.4) % | 2501/5430 |
| Peripheral vascular disease |  | 3.5 (3-4) % | 198/5628 | 3 (1-5.6) % | 6/198 | 3.5 (3.1-4) % | 192/5430 |
| COPD |  | 7.8 (7.1-8.5) % | 437/5628 | 12.6 (8.1-17.2) % | 25/198 | 7.6 (6.9-8.4) % | 412/5430 |
| Chronic lung disease |  | 8.4 (7.7-9.1) % | 470/5628 | 10.1 (6.1-14.6) % | 20/198 | 8.3 (7.5-9) % | 450/5430 |
| OSA |  | 2.8 (2.4-3.2) % | 156/5628 | 1 (0-2.5) % | 2/198 | 2.8 (2.4-3.3) % | 154/5430 |
| Stroke |  | 6.4 (5.8-7.1) % | 360/5628 | 12.6 (8.1-17.7) % | 25/198 | 6.2 (5.5-6.8) % | 335/5430 |
| Hemiplegia |  | 0.2 (0.1-0.4) % | 14/5628 | 1.5 (0-3.5) % | 3/198 | 0.2 (0.1-0.3) % | 11/5430 |
| Anxiety / depression |  | 8.3 (7.6-9.1) % | 469/5628 | 16.2 (11.1-21.7) % | 32/198 | 8 (7.3-8.8) % | 437/5430 |
| Parkinson's disease |  | 0.5 (0.3-0.7) % | 29/5628 | 1.5 (0-3.5) % | 3/198 | 0.5 (0.3-0.7) % | 26/5430 |
| Diabetes |  |  |  |  |  |  |  |
|  | Present | 14.4 (13.4-15.3) % | 808/5628 | 17.2 (12.1-22.2) % | 34/198 | 14.3 (13.3-15.2) % | 774/5430 |
|  | Diabetes without chronic complications | 76.6 (73.6-79.4) % | 618/807 | 85.3 (73.5-97.1) % | 29/34 | 76.2 (73.2-79.2) % | 589/773 |
|  | Diabetes with chronic complications | 23.4 (20.4-26.4) % | 189/807 | 14.7 (2.9-26.5) % | 5/34 | 23.8 (20.8-26.6) % | 184/773 |
| Chronic kidney disease |  | 7.4 (6.7-8.1) % | 415/5628 | 7.1 (4-10.6) % | 14/198 | 7.4 (6.7-8.1) % | 401/5430 |
| Benign prostatic hyperplasia |  | 7 (6.3-7.7) % | 394/5628 | 5.1 (2.5-8.1) % | 10/198 | 7.1 (6.4-7.8) % | 384/5430 |
| Liver disease |  |  |  |  |  |  |  |
|  | Present | 1.4 (1.1-1.7) % | 80/5628 | NA | NA | 1.5 (1.2-1.8) % | 80/5430 |
| Liver disease severity | Mild liver disease | 1.2 (1-1.5) % | 69/5628 | NA | NA | 1.3 (1-1.6) % | 69/5430 |
| Liver disease severity | Moderate or severe liver disease | 0.2 (0.1-0.3) % | 9/5628 | NA | NA | 0.2 (0.1-0.3) % | 9/5430 |
| Peptic ulcer disease |  | 3.2 (2.8-3.7) % | 182/5628 | 3.5 (1-6.1) % | 7/198 | 3.2 (2.8-3.7) % | 175/5430 |
| Lymphoma |  | 1 (0.7-1.2) % | 54/5628 | 2 (0.5-4.5) % | 4/198 | 0.9 (0.7-1.2) % | 50/5430 |
| Leukaemia |  | 0.6 (0.4-0.8) % | 33/5628 | NA | NA | 0.6 (0.4-0.8) % | 33/5430 |
| Connective tissue disorders |  | 4 (3.5-4.5) % | 226/5628 | 3.5 (1-6.1) % | 7/198 | 4 (3.5-4.6) % | 219/5430 |
| Osteoarthritis |  | 26.5 (25.4-27.7) % | 1494/5628 | 22.7 (17.2-28.8) % | 45/198 | 26.7 (25.5-27.9) % | 1449/5430 |
| Malignancy |  |  |  |  |  |  |  |
|  | Present | 21.7 (20.5-22.7) % | 1219/5628 | 18.7 (13.6-24.2) % | 37/198 | 21.8 (20.7-22.9) % | 1182/5430 |
|  | Malignancy without metastasis | 18.2 (17.3-19.2) % | 1027/5628 | 15.2 (10.1-20.2) % | 30/198 | 18.4 (17.3-19.4) % | 997/5430 |
|  | Malignancy with metastasis | 3.5 (3.1-4) % | 198/5628 | 3.5 (1-6.1) % | 7/198 | 3.5 (3-4) % | 191/5430 |
|  | Malignancy diagnosis ≤ 5 years | 17.2 (16.2-18.3) % | 969/5628 | 15.2 (10.6-20.2) % | 30/198 | 17.3 (16.3-18.3) % | 939/5430 |
|  | Malignancy diagnosis > 5 years | 4.7 (4.1-5.2) % | 263/5628 | 4 (1.5-7.1) % | 8/198 | 4.7 (4.2-5.3) % | 255/5430 |
| Haematology |  |  |  |  |  |  |  |
|  | Haemoglobin (g/l) | 134.4 (133.9-134.8) | 5042 | 127.9 (124.9-130.9) | 193 | 134.6 (134.2-135.1) | 4849 |
|  | WCC (x10^9/L) | 7.9 (7.8-8) | 5042 | 9.7 (9.1-10.4) | 193 | 7.8 (7.7-8) | 4849 |
|  | Neutrophil count (x10^9/L) | 5.2 (5.1-5.3) | 5011 | 7.3 (6.6-7.9) | 192 | 5.1 (5.1-5.2) | 4819 |
| Biochemistry |  |  |  |  |  |  |  |
|  | eGFR (ml/min) | 76 (61-88) | 4992 | 72 (57.2-86) | 190 | 76 (61-88) | 4802 |
|  | Sodium (mmol/l) | 139 (137-141) | 5077 | 138 (136-140) | 191 | 139 (138-141) | 4886 |
|  | Potassium (mmol/l) | 4.4 (4.4-4.4) | 5052 | 4.2 (4.2-4.3) | 190 | 4.4 (4.4-4.4) | 4862 |
|  | Creatinine (micromol/l) | 77 (65-93) | 5071 | 80 (65.8-98) | 192 | 77 (65-93) | 4879 |

#### Supplementary table 6E: Clinical characteristics of participants having elective surgery in the SNAP-3 cohort, with and without delirium

|  |  | **All** |  | **Delirium** |  | **No delirium** |  |
| --- | --- | --- | --- | --- | --- | --- | --- |
| **Characteristic** |  | **Summary** | **Number** | **Summary** | **Number** | **Summary** | **Number** |
| Number of comorbidities | Total comorbidities | 2 (1-3) | 4338 | 2.5 (1-4) | 128 | 2 (1-3) | 4210 |
| SORT predicted morbidity (%) | Predicted morbidity | 22.1 (21.6-22.6) | 4849 | 38.3 (34.6-41.8) | 134 | 21.7 (21.2-22.2) | 4715 |
| SORT predicted mortality (%) | Predicted mortality | 0.6 (0.6-0.7) | 4911 | 1.4 (1.1-1.6) | 134 | 0.6 (0.6-0.6) | 4777 |
| Polypharmacy |  | 45.2 (43.8-46.6) % | 2202/4875 | 61.4 (53-68.9) % | 81/132 | 44.7 (43.4-46.1) % | 2121/4743 |
| BMI category |  |  |  |  |  |  |  |
|  | Underweight | 1 (0.7-1.3) % | 49/4890 | 1.5 (0-3.7) % | 2/134 | 1 (0.7-1.3) % | 47/4756 |
|  | Healthy weight | 25.8 (24.6-26.9) % | 1260/4890 | 30.6 (22.4-38.8) % | 41/134 | 25.6 (24.4-27) % | 1219/4756 |
|  | Overweight | 37.8 (36.3-39.1) % | 1846/4890 | 35.1 (26.9-43.3) % | 47/134 | 37.8 (36.5-39.2) % | 1799/4756 |
|  | Obese class 1 | 31.5 (30.2-32.8) % | 1541/4890 | 32.8 (25.4-41) % | 44/134 | 31.5 (30.2-32.7) % | 1497/4756 |
|  | Obese class >=2 | 4 (3.4-4.5) % | 194/4890 | NA | NA | 4.1 (3.6-4.6) % | 194/4756 |
| Hearing impairment |  | 8 (7.3-8.8) % | 396/4921 | 14.2 (8.2-20.9) % | 19/134 | 7.9 (7.1-8.6) % | 377/4787 |
| Visual impairment |  | 2.2 (1.8-2.6) % | 107/4921 | 3.7 (0.7-7.5) % | 5/134 | 2.1 (1.7-2.6) % | 102/4787 |
| Dementia |  | 1 (0.8-1.3) % | 51/4921 | 6.7 (3-11.2) % | 9/134 | 0.9 (0.6-1.1) % | 42/4787 |
| Myocardial infarction |  | 8.7 (7.9-9.5) % | 426/4921 | 6.7 (3-11.2) % | 9/134 | 8.7 (7.9-9.5) % | 417/4787 |
| Heart failure |  | 4 (3.4-4.6) % | 197/4921 | 9.7 (5.2-14.9) % | 13/134 | 3.8 (3.3-4.4) % | 184/4787 |
| Atrial fibrillation |  | 9.7 (8.9-10.5) % | 477/4921 | 20.9 (14.2-28.4) % | 28/134 | 9.4 (8.6-10.2) % | 449/4787 |
| Valvular heart disease |  | 3.9 (3.4-4.5) % | 193/4921 | 11.2 (6-17.2) % | 15/134 | 3.7 (3.2-4.3) % | 178/4787 |
| Hypertension |  | 48.4 (47-49.8) % | 2380/4921 | 55.2 (47-63.4) % | 74/134 | 48.2 (46.7-49.5) % | 2306/4787 |
| Peripheral vascular disease |  | 3.6 (3.1-4.1) % | 177/4921 | 5.2 (1.5-9) % | 7/134 | 3.6 (3-4.1) % | 170/4787 |
| COPD |  | 8.9 (8.1-9.7) % | 436/4921 | 11.2 (6-17.2) % | 15/134 | 8.8 (8-9.6) % | 421/4787 |
| Chronic lung disease |  | 8.9 (8.1-9.7) % | 437/4921 | 11.2 (6-16.4) % | 15/134 | 8.8 (8-9.6) % | 422/4787 |
| OSA |  | 3.5 (3-4) % | 174/4921 | 2.2 (0-5.2) % | 3/134 | 3.6 (3.1-4.1) % | 171/4787 |
| Stroke |  | 7.2 (6.4-7.9) % | 353/4921 | 14.2 (9-20.9) % | 19/134 | 7 (6.2-7.7) % | 334/4787 |
| Hemiplegia |  | 0.5 (0.3-0.7) % | 25/4921 | 3.7 (0.7-7.5) % | 5/134 | 0.4 (0.3-0.6) % | 20/4787 |
| Anxiety / depression |  | 9.8 (8.9-10.6) % | 481/4921 | 17.2 (10.4-23.9) % | 23/134 | 9.6 (8.7-10.4) % | 458/4787 |
| Parkinson's disease |  | 0.7 (0.4-0.9) % | 32/4921 | 0.7 (0-2.2) % | 1/134 | 0.6 (0.4-0.9) % | 31/4787 |
| Diabetes |  |  |  |  |  |  |  |
|  | Present | 15.5 (14.5-16.6) % | 764/4921 | 20.9 (14.2-27.6) % | 28/134 | 15.4 (14.4-16.4) % | 736/4787 |
|  | Diabetes without chronic complications | 74.9 (71.7-77.9) % | 572/764 | 78.6 (60.7-92.9) % | 22/28 | 74.7 (71.5-77.8) % | 550/736 |
|  | Diabetes with chronic complications | 25.1 (22.1-28.3) % | 192/764 | 21.4 (7.1-35.7) % | 6/28 | 25.3 (22.3-28.4) % | 186/736 |
| Chronic kidney disease |  | 8.3 (7.5-9) % | 406/4921 | 7.5 (3-11.9) % | 10/134 | 8.3 (7.5-9) % | 396/4787 |
| Benign prostatic hyperplasia |  | 6.9 (6.2-7.6) % | 338/4921 | 4.5 (1.5-8.2) % | 6/134 | 6.9 (6.2-7.6) % | 332/4787 |
| Liver disease |  |  |  |  |  |  |  |
|  | Present | 1.6 (1.2-1.9) % | 78/4921 | 0.7 (0-2.2) % | 1/134 | 1.6 (1.3-2) % | 77/4787 |
|  | Mild liver disease | 1.4 (1.1-1.8) % | 71/4921 | 0.7 (0-2.2) % | 1/134 | 1.5 (1.1-1.8) % | 70/4787 |
|  | Moderate or severe liver disease | 0.1 (0-0.2) % | 6/4921 | NA | NA | 0.1 (0-0.2) % | 6/4787 |
| Peptic ulcer disease |  | 3.3 (2.8-3.8) % | 160/4921 | 5.2 (1.5-9) % | 7/134 | 3.2 (2.7-3.7) % | 153/4787 |
| Lymphoma |  | 0.9 (0.7-1.2) % | 45/4921 | 1.5 (0-3.7) % | 2/134 | 0.9 (0.6-1.2) % | 43/4787 |
| Leukaemia |  | 0.6 (0.4-0.8) % | 29/4921 | 0.7 (0-2.2) % | 1/134 | 0.6 (0.4-0.8) % | 28/4787 |
| Connective tissue disorders |  | 4.4 (3.8-5) % | 216/4921 | 1.5 (0-3.7) % | 2/134 | 4.5 (3.9-5.1) % | 214/4787 |
| Osteoarthritis |  | 30.2 (28.8-31.4) % | 1484/4921 | 32.8 (24.6-40.3) % | 44/134 | 30.1 (28.8-31.4) % | 1440/4787 |
| Malignancy |  |  |  |  |  |  |  |
|  | Present | 21.9 (20.7-23) % | 1078/4921 | 27.6 (20.1-35.1) % | 37/134 | 21.7 (20.6-22.9) % | 1041/4787 |
|  | Malignancy without metastasis | 18.5 (17.5-19.7) % | 912/4921 | 22.4 (14.9-29.1) % | 30/134 | 18.4 (17.3-19.6) % | 882/4787 |
|  | Malignancy with metastasis | 3.4 (2.9-4) % | 169/4921 | 5.2 (1.5-9) % | 7/134 | 3.4 (2.9-3.9) % | 162/4787 |
|  | Malignancy diagnosis ≤ 5 years | 17 (16-18.1) % | 838/4921 | 21.6 (14.9-28.4) % | 29/134 | 16.9 (15.8-17.9) % | 809/4787 |
|  | Malignancy diagnosis > 5 years | 5.1 (4.5-5.7) % | 249/4921 | 6.7 (3-11.2) % | 9/134 | 5 (4.4-5.6) % | 240/4787 |
| Haematology |  |  |  |  |  |  |  |
|  | Haemoglobin (g/l) | 135.1 (134.7-135.6) | 4351 | 131.4 (128.3-134.5) | 128 | 135.2 (134.8-135.7) | 4223 |
|  | WCC (x10^9/L) | 7.4 (7.3-7.5) | 4351 | 7.7 (7.2-8.1) | 128 | 7.4 (7.3-7.5) | 4223 |
|  | Neutrophil count (x10^9/L) | 4.7 (4.6-4.7) | 4320 | 5.2 (4.7-5.6) | 126 | 4.7 (4.6-4.7) | 4194 |
| Biochemistry |  |  |  |  |  |  |  |
|  | eGFR (ml/min) | 75 (61-88) | 4338 | 72 (56.8-84.2) | 128 | 75 (61-88) | 4210 |
|  | Sodium (mmol/l) | 140 (138-141) | 4392 | 139 (137-141) | 129 | 140 (138-141) | 4263 |
|  | Potassium (mmol/l) | 4.4 (4.4-4.4) | 4378 | 4.3 (4.3-4.4) | 129 | 4.4 (4.4-4.4) | 4249 |
|  | Creatinine (micromol/l) | 78 (65-94) | 4386 | 82 (68-98) | 129 | 78 (65-94) | 4257 |

#### Supplementary table 6F: Clinical characteristics of participants having non-elective surgery in the SNAP-3 cohort, with and without delirium

|  |  | **All** | | **Delirium** | | **No delirium** | |
| --- | --- | --- | --- | --- | --- | --- | --- |
| **Characteristic** |  | **Summary** | **Number** | **Summary** | **Number** | **Summary** | **Number** |
| Number of comorbidities | Total comorbidities | 2 (1-4) | 1960 | 3 (2-4) | 332 | 2 (1-3) | 1628 |
| SORT predicted morbidity (%) | Predicted morbidity | 24.2 (23.4-25) | 2106 | 29 (27.2-30.8) | 341 | 23.2 (22.4-24.1) | 1765 |
| SORT predicted mortality (%) | Predicted mortality | 4.7 (4.5-5) | 2130 | 8.3 (7.4-9.3) | 344 | 4 (3.8-4.3) | 1786 |
| Polypharmacy |  | 51.1 (49-53.1) % | 1080/2113 | 67.1 (61.8-72.1) % | 228/340 | 48.1 (45.6-50.5) % | 852/1773 |
| BMI category |  |  |  |  |  |  |  |
|  | Underweight | 4.1 (3.3-5) % | 87/2104 | 8.6 (5.6-11.9) % | 29/337 | 3.3 (2.5-4.1) % | 58/1767 |
|  | Healthy weight | 39.6 (37.5-41.7) % | 834/2104 | 49 (43.3-54.6) % | 165/337 | 37.9 (35.6-40.1) % | 669/1767 |
|  | Overweight | 31.8 (29.8-33.9) % | 669/2104 | 27.3 (22.6-32) % | 92/337 | 32.7 (30.6-34.9) % | 577/1767 |
|  | Obese class 1 | 21.7 (19.9-23.6) % | 457/2104 | 13.4 (9.5-16.9) % | 45/337 | 23.3 (21.4-25.2) % | 412/1767 |
|  | Obese class >=2 | 2.7 (2-3.4) % | 57/2104 | 1.8 (0.6-3.3) % | 6/337 | 2.9 (2.2-3.7) % | 51/1767 |
| Hearing impairment |  | 9.4 (8.3-10.7) % | 201/2134 | 13.6 (10.1-17.4) % | 47/345 | 8.6 (7.4-9.9) % | 154/1789 |
| Visual impairment |  | 3.7 (2.9-4.5) % | 79/2134 | 7.5 (4.9-10.4) % | 26/345 | 3 (2.2-3.7) % | 53/1789 |
| Dementia |  | 6.2 (5.2-7.3) % | 133/2134 | 26.7 (22-31) % | 92/345 | 2.3 (1.6-3) % | 41/1789 |
| Myocardial infarction |  | 12 (10.5-13.4) % | 256/2134 | 14.2 (10.4-18) % | 49/345 | 11.6 (10.1-13.1) % | 207/1789 |
| Heart failure |  | 7.3 (6.2-8.4) % | 155/2134 | 11.3 (8.1-14.8) % | 39/345 | 6.5 (5.4-7.7) % | 116/1789 |
| Atrial fibrillation |  | 14 (12.5-15.4) % | 298/2134 | 23.8 (19.1-28.4) % | 82/345 | 12.1 (10.6-13.6) % | 216/1789 |
| Valvular heart disease |  | 5.7 (4.7-6.7) % | 122/2134 | 7.2 (4.6-10.1) % | 25/345 | 5.4 (4.5-6.4) % | 97/1789 |
| Hypertension |  | 49.6 (47.3-51.6) % | 1058/2134 | 54.8 (49.6-60) % | 189/345 | 48.6 (46.2-50.9) % | 869/1789 |
| Peripheral vascular disease |  | 7 (5.9-8.1) % | 149/2134 | 7.2 (4.6-9.9) % | 25/345 | 6.9 (5.8-8.2) % | 124/1789 |
| COPD |  | 11.1 (9.8-12.5) % | 237/2134 | 12.2 (9-15.7) % | 42/345 | 10.9 (9.4-12.4) % | 195/1789 |
| Chronic lung disease |  | 8.4 (7.3-9.6) % | 179/2134 | 8.1 (5.2-11) % | 28/345 | 8.4 (7.2-9.8) % | 151/1789 |
| OSA |  | 1.9 (1.4-2.5) % | 41/2134 | 0.6 (0-1.4) % | 2/345 | 2.2 (1.5-2.9) % | 39/1789 |
| Stroke |  | 10.1 (8.8-11.3) % | 215/2134 | 15.9 (12.2-20) % | 55/345 | 8.9 (7.7-10.3) % | 160/1789 |
| Hemiplegia |  | 1.3 (0.8-1.8) % | 28/2134 | 2.6 (1.2-4.3) % | 9/345 | 1.1 (0.6-1.6) % | 19/1789 |
| Anxiety / depression |  | 10.2 (9-11.4) % | 218/2134 | 14.2 (10.7-18) % | 49/345 | 9.4 (8-10.8) % | 169/1789 |
| Parkinson's disease |  | 1.5 (1.1-2.1) % | 33/2134 | 3.2 (1.4-5.2) % | 11/345 | 1.2 (0.7-1.8) % | 22/1789 |
| Diabetes |  |  |  |  |  |  |  |
|  | Present | 18.2 (16.5-20) % | 389/2134 | 21.7 (17.4-26.1) % | 75/345 | 17.6 (15.7-19.5) % | 314/1789 |
|  | Diabetes without chronic complications | 63.1 (58.2-67.8) % | 245/388 | 58.7 (48-69.3) % | 44/75 | 64.2 (58.8-69.6) % | 201/313 |
|  | Diabetes with chronic complications | 36.9 (32.2-41.8) % | 143/388 | 41.3 (30.7-53.3) % | 31/75 | 35.8 (30.7-41.2) % | 112/313 |
| Chronic kidney disease |  | 13.6 (12.2-15) % | 291/2134 | 19.7 (15.7-24.1) % | 68/345 | 12.5 (10.9-14.1) % | 223/1789 |
| Benign prostatic hyperplasia |  | 7 (5.9-8.2) % | 150/2134 | 5.5 (3.2-8.1) % | 19/345 | 7.3 (6.2-8.6) % | 131/1789 |
| Liver disease |  |  |  |  |  |  |  |
|  | Present | 2.1 (1.5-2.7) % | 44/2134 | 2 (0.6-3.5) % | 7/345 | 2.1 (1.5-2.8) % | 37/1789 |
|  | Mild liver disease | 1.7 (1.2-2.2) % | 36/2134 | 1.7 (0.6-3.2) % | 6/345 | 1.7 (1.1-2.3) % | 30/1789 |
|  | Moderate or severe liver disease | 0.3 (0.1-0.6) % | 7/2134 | 0.3 (0-0.9) % | 1/345 | 0.3 (0.1-0.6) % | 6/1789 |
| Peptic ulcer disease |  | 3.9 (3.1-4.8) % | 84/2134 | 3.2 (1.4-5.2) % | 11/345 | 4.1 (3.2-5) % | 73/1789 |
| Lymphoma |  | 1.1 (0.7-1.6) % | 24/2134 | 1.4 (0.3-2.9) % | 5/345 | 1.1 (0.6-1.6) % | 19/1789 |
| Leukaemia |  | 0.6 (0.3-0.9) % | 12/2134 | 0.6 (0-1.4) % | 2/345 | 0.6 (0.3-0.9) % | 10/1789 |
| Connective tissue disorders |  | 5.8 (4.9-6.7) % | 124/2134 | 4.1 (2-6.4) % | 14/345 | 6.1 (5.1-7.3) % | 110/1789 |
| Osteoarthritis |  | 27.1 (25.3-29) % | 579/2134 | 27.8 (22.9-32.5) % | 96/345 | 27 (25-29.1) % | 483/1789 |
| Malignancy |  |  |  |  |  |  |  |
|  | Present | 19.2 (17.5-20.8) % | 409/2134 | 13 (9.9-16.8) % | 45/345 | 20.3 (18.6-22.2) % | 364/1789 |
|  | Malignancy without metastasis | 15.5 (14-17.1) % | 331/2134 | 9.9 (7-13) % | 34/345 | 16.6 (14.9-18.3) % | 297/1789 |
|  | Malignancy with metastasis | 3.8 (3-4.6) % | 81/2134 | 3.2 (1.4-5.2) % | 11/345 | 3.9 (3-4.9) % | 70/1789 |
|  | Malignancy diagnosis ≤ 5 years | 14.9 (13.4-16.4) % | 319/2134 | 9.3 (6.4-12.5) % | 32/345 | 16 (14.3-17.8) % | 287/1789 |
|  | Malignancy diagnosis > 5 years | 4.5 (3.6-5.3) % | 95/2134 | 4.1 (2-6.4) % | 14/345 | 4.5 (3.6-5.4) % | 81/1789 |
| Haematology |  |  |  |  |  |  |  |
|  | Haemoglobin (g/l) | 125.5 (124.5-126.3) | 2036 | 119.3 (117.2-121.4) | 341 | 126.7 (125.8-127.7) | 1695 |
|  | WCC (x10^9/L) | 9.8 (9.6-10.1) | 2036 | 11.2 (10.7-11.8) | 341 | 9.6 (9.4-9.8) | 1695 |
|  | Neutrophil count (x10^9/L) | 7.3 (7.1-7.5) | 2028 | 8.8 (8.4-9.3) | 339 | 7 (6.8-7.2) | 1689 |
| Biochemistry |  |  |  |  |  |  |  |
|  | eGFR (ml/min) | 74 (58.3-89) | 1960 | 68 (54-84) | 332 | 75 (60-90) | 1628 |
|  | Sodium (mmol/l) | 138 (136-140) | 2028 | 138 (136-140) | 339 | 138 (136-140) | 1689 |
|  | Potassium (mmol/l) | 4.3 (4.3-4.3) | 2012 | 4.2 (4.2-4.3) | 336 | 4.3 (4.3-4.3) | 1676 |
|  | Creatinine (micromol/l) | 75 (61-95) | 2025 | 77 (60-99.5) | 339 | 75 (61-94) | 1686 |

#### Supplementary table 6G: Clinical characteristics of participants having inpatient surgery in the SNAP-3 cohort, with and without delirium

|  |  | **All** | | **Delirium** | | **No delirium** | |
| --- | --- | --- | --- | --- | --- | --- | --- |
| **Characteristic** |  | **Summary** | **Number** | **Summary** | **Number** | **Summary** | **Number** |
| Number of comorbidities | Total comorbidities | 2 (1-3) | 4322 | 3 (2-4) | 458 | 2 (1-3) | 3864 |
| SORT predicted morbidity (%) | Predicted morbidity | 26.6 (26.1-27.2) | 4598 | 31.7 (30-33.4) | 473 | 26 (25.4-26.6) | 4125 |
| SORT predicted mortality (%) | Predicted mortality | 2.5 (2.4-2.7) | 4643 | 6.3 (5.6-7.1) | 476 | 2.1 (2-2.2) | 4167 |
| Polypharmacy |  | 52.1 (50.7-53.6) % | 2401/4605 | 65.3 (61.3-69.6) % | 307/470 | 50.6 (49.1-52.2) % | 2094/4135 |
| BMI category |  |  |  |  |  |  |  |
|  | Underweight | 2.4 (1.9-2.8) % | 109/4605 | 6.6 (4.5-9) % | 31/469 | 1.9 (1.5-2.3) % | 78/4136 |
|  | Healthy weight | 30.8 (29.5-32.2) % | 1418/4605 | 43.7 (39.2-48.2) % | 205/469 | 29.3 (28-30.8) % | 1213/4136 |
|  | Overweight | 34.6 (33.3-36) % | 1595/4605 | 29.6 (25.6-33.9) % | 139/469 | 35.2 (33.7-36.6) % | 1456/4136 |
|  | Obese class 1 | 28.8 (27.4-30.1) % | 1324/4605 | 18.8 (15.1-22.4) % | 88/469 | 29.9 (28.5-31.4) % | 1236/4136 |
|  | Obese class >=2 | 3.5 (2.9-4) % | 159/4605 | 1.3 (0.4-2.3) % | 6/469 | 3.7 (3.1-4.3) % | 153/4136 |
| Hearing impairment |  | 9 (8.2-9.9) % | 420/4650 | 13.8 (10.5-17) % | 66/477 | 8.5 (7.7-9.3) % | 354/4173 |
| Visual impairment |  | 3 (2.5-3.5) % | 139/4650 | 6.5 (4.4-8.8) % | 31/477 | 2.6 (2.1-3.1) % | 108/4173 |
| Dementia |  | 3.5 (3-4.1) % | 163/4650 | 21.2 (17.4-24.7) % | 101/477 | 1.5 (1.2-1.9) % | 62/4173 |
| Myocardial infarction |  | 10.6 (9.7-11.5) % | 495/4650 | 11.9 (9-14.9) % | 57/477 | 10.5 (9.6-11.4) % | 438/4173 |
| Heart failure |  | 6.1 (5.4-6.8) % | 285/4650 | 10.7 (8-13.6) % | 51/477 | 5.6 (4.9-6.4) % | 234/4173 |
| Atrial fibrillation |  | 13 (12-14) % | 604/4650 | 23.1 (19.3-26.8) % | 110/477 | 11.8 (10.8-12.8) % | 494/4173 |
| Valvular heart disease |  | 5.4 (4.8-6.1) % | 253/4650 | 8.4 (6.1-11.1) % | 40/477 | 5.1 (4.5-5.8) % | 213/4173 |
| Hypertension |  | 50.8 (49.4-52.3) % | 2363/4650 | 54.9 (50.5-59.3) % | 262/477 | 50.3 (48.9-51.9) % | 2101/4173 |
| Peripheral vascular disease |  | 5.7 (5-6.3) % | 264/4650 | 6.7 (4.6-9) % | 32/477 | 5.6 (4.8-6.3) % | 232/4173 |
| COPD |  | 9.9 (9-10.8) % | 461/4650 | 11.9 (9-14.9) % | 57/477 | 9.7 (8.7-10.6) % | 404/4173 |
| Chronic lung disease |  | 8.7 (7.9-9.5) % | 406/4650 | 9 (6.7-11.7) % | 43/477 | 8.7 (7.9-9.6) % | 363/4173 |
| OSA |  | 3.2 (2.7-3.7) % | 150/4650 | 1 (0.2-2.1) % | 5/477 | 3.5 (2.9-4) % | 145/4173 |
| Stroke |  | 9.2 (8.3-10) % | 426/4650 | 15.5 (12.4-18.9) % | 74/477 | 8.4 (7.6-9.3) % | 352/4173 |
| Hemiplegia |  | 1 (0.7-1.3) % | 45/4650 | 2.9 (1.5-4.4) % | 14/477 | 0.7 (0.5-1) % | 31/4173 |
| Anxiety / depression |  | 10.3 (9.5-11.2) % | 480/4650 | 15.1 (11.9-18.4) % | 72/477 | 9.8 (8.9-10.7) % | 408/4173 |
| Parkinson's disease |  | 1.1 (0.8-1.4) % | 49/4650 | 2.5 (1.3-4) % | 12/477 | 0.9 (0.6-1.2) % | 37/4173 |
| Diabetes |  |  |  |  |  |  |  |
|  | Present | 17.5 (16.5-18.6) % | 815/4650 | 21.6 (17.6-25.2) % | 103/477 | 17.1 (16-18.2) % | 712/4173 |
|  | Diabetes without chronic complications | 67.9 (64.9-71.1) % | 553/814 | 64.1 (54.4-72.8) % | 66/103 | 68.5 (65-71.9) % | 487/711 |
|  | Diabetes with chronic complications | 32.1 (28.8-35.4) % | 261/814 | 35.9 (27.2-45.6) % | 37/103 | 31.5 (28.1-34.9) % | 224/711 |
| Chronic kidney disease |  | 11.1 (10.2-12) % | 515/4650 | 16.4 (13-19.7) % | 78/477 | 10.5 (9.6-11.4) % | 437/4173 |
| Benign prostatic hyperplasia |  | 7.3 (6.6-8.1) % | 339/4650 | 5.2 (3.4-7.3) % | 25/477 | 7.5 (6.8-8.3) % | 314/4173 |
| Liver disease |  |  |  |  |  |  |  |
|  | Present | 1.8 (1.4-2.2) % | 84/4650 | 1.7 (0.6-2.9) % | 8/477 | 1.8 (1.4-2.2) % | 76/4173 |
| Liver disease severity | Mild liver disease | 1.6 (1.2-1.9) % | 74/4723 | 1.5 (0.6-2.7) % | 7/477 | 1.6 (1.2-2) % | 67/4246 |
| Liver disease severity | Moderate or severe liver disease | 0.2 (0.1-0.3) % | 9/4723 | 0.2 (0-0.6) % | 1/477 | 0.2 (0.1-0.3) % | 8/4246 |
| Peptic ulcer disease |  | 3.7 (3.1-4.2) % | 171/4650 | 3.8 (2.1-5.7) % | 18/477 | 3.7 (3.1-4.3) % | 153/4173 |
| Lymphoma |  | 1.1 (0.8-1.4) % | 49/4650 | 1.3 (0.4-2.3) % | 6/477 | 1 (0.7-1.3) % | 43/4173 |
| Leukaemia |  | 0.6 (0.4-0.9) % | 29/4650 | 0.6 (0-1.5) % | 3/477 | 0.6 (0.4-0.9) % | 26/4173 |
| Connective tissue disorders |  | 5 (4.4-5.6) % | 232/4650 | 3.4 (1.9-5) % | 16/477 | 5.2 (4.5-5.8) % | 216/4173 |
| Osteoarthritis |  | 32.2 (30.9-33.6) % | 1497/4650 | 29.1 (25.2-33.1) % | 139/477 | 32.5 (31.2-34) % | 1358/4173 |
| Malignancy |  |  |  |  |  |  |  |
|  | Present | 21.1 (19.9-22.3) % | 983/4650 | 17.2 (13.6-20.5) % | 82/477 | 21.6 (20.4-22.8) % | 901/4173 |
|  | Malignancy without metastasis | 17.3 (16.2-18.4) % | 803/4650 | 13.4 (10.3-16.8) % | 64/477 | 17.7 (16.6-18.9) % | 739/4173 |
|  | Malignancy with metastasis | 4 (3.4-4.5) % | 184/4650 | 3.8 (2.1-5.7) % | 18/477 | 4 (3.4-4.6) % | 166/4173 |
|  | Malignancy diagnosis ≤ 5 years | 16.7 (15.5-17.7) % | 775/4650 | 12.8 (9.9-15.9) % | 61/477 | 17.1 (16.1-18.3) % | 714/4173 |
|  | Malignancy diagnosis > 5 years | 4.7 (4.1-5.3) % | 217/4650 | 4.8 (2.9-6.7) % | 23/477 | 4.6 (4-5.3) % | 194/4173 |
| Haematology |  |  |  |  |  |  |  |
|  | Haemoglobin (g/l) | 130.1 (129.6-130.7) | 4432 | 122.6 (120.6-124.4) | 467 | 131 (130.5-131.6) | 3965 |
|  | WCC (x10^9/L) | 8.5 (8.4-8.6) | 4432 | 10.2 (9.8-10.7) | 467 | 8.3 (8.2-8.4) | 3965 |
|  | Neutrophil count (x10^9/L) | 5.9 (5.8-6) | 4404 | 7.8 (7.5-8.2) | 463 | 5.7 (5.6-5.8) | 3941 |
| Biochemistry |  |  |  |  |  |  |  |
|  | eGFR (ml/min) | 74 (60-88) | 4382 | 69.1 (55-84) | 458 | 75 (61-89) | 3924 |
|  | Sodium (mmol/l) | 139 (137-141) | 4416 | 138 (136-141) | 466 | 139 (137-141) | 3950 |
|  | Potassium (mmol/l) | 4.3 (4.3-4.4) | 4394 | 4.3 (4.2-4.3) | 463 | 4.4 (4.3-4.4) | 3931 |
|  | Creatinine (micromol/l) | 77 (64-94) | 4409 | 79 (62-99) | 466 | 77 (64-94) | 3943 |

### Outcomes: subgroups

LOS: length of stay; Postoperative morbidity is morbidity defined by the PostOperative Morbidity Survey (POMS)

#### Supplementary table 7A: Outcomes of participants aged ≥85 in the SNAP-3 cohort, with and without delirium

|  |  | **All** |  | **Delirium** |  | **No delirium** |  |
| --- | --- | --- | --- | --- | --- | --- | --- |
| **Characteristic** |  | **Summary** | **Number** | **Summary** | **Number** | **Summary** | **Number** |
| Postoperative LOS (days) | Median [IQR] | 5 [1-14] | 718 | 15.5 [7.8-28.2] | 152 | 2 [1-9] | 566 |
|  | 80th centile | 17 |  | 32 |  | 12 |  |
| Postoperative morbidity* |  | 49 (45.4-52.8) % | 365/745 | 89.6 (84.7-93.9) % | 146/163 | 37.6 (33.8-41.8) % | 219/582 |
| Mortality |  |  |  |  |  |  |  |
|  | 30-day | 3.8 (2.4-5.1) % | 28/739 | 8.7 (4.3-13) % | 14/161 | 2.4 (1.2-3.8) % | 14/578 |
|  | 120-day | 9.7 (7.7-11.9) % | 72/739 | 23.6 (17.4-30.4) % | 38/161 | 5.9 (4-7.8) % | 34/578 |
|  | 1-year | 18.1 (15.3-21) % | 134/739 | 39.8 (32.3-47.8) % | 64/161 | 12.1 (9.3-14.9) % | 70/578 |

#### Supplementary table 7B: Outcomes of participants aged <85 in the SNAP-3 cohort, with and without delirium

|  |  | **All** | | **Delirium** | | **No delirium** | |
| --- | --- | --- | --- | --- | --- | --- | --- |
| **Characteristic** |  | **Summary** | **Number** | **Summary** | **Number** | **Summary** | **Number** |
| Postoperative LOS (days) | Median [IQR] | 1 [0-4] | 6178 | 11 [6-20] | 302 | 1 [0-3] | 5876 |
|  | 80th centile | 5 |  | 22.8 |  | 4 |  |
| Postoperative morbidity* |  | 24.3 (23.2-25.4) % | 1533/6306 | 85.4 (81.6-89.2) % | 269/315 | 21.1 (20.1-22.1) % | 1264/5991 |
| Mortality |  |  |  |  |  |  |  |
|  | 30-day | 0.7 (0.5-1) % | 47/6285 | 4.1 (2.2-6.7) % | 13/315 | 0.6 (0.4-0.8) % | 34/5970 |
|  | 120-day | 2.2 (1.9-2.6) % | 140/6285 | 9.8 (6.7-13.3) % | 31/315 | 1.8 (1.5-2.2) % | 109/5970 |
|  | 1-year | 5.6 (5-6.1) % | 351/6285 | 20 (15.6-24.4) % | 63/315 | 4.8 (4.3-5.4) % | 288/5970 |

#### Supplementary table 7C: Outcomes of participants living with frailty in the SNAP-3 cohort, with and without delirium

|  |  | **All** | | **Delirium** | | **No delirium** | |
| --- | --- | --- | --- | --- | --- | --- | --- |
| **Characteristic** |  | **Summary** | **Number** | **Summary** | **Number** | **Summary** | **Number** |
| Postoperative LOS (days) | Median [IQR] | 4 [1-13] | 1322 | 14 [7-27] | 259 | 3 [1-9] | 1063 |
|  | 80th centile | 15 |  | 30 |  | 11.6 |  |
| Postoperative morbidity* |  | 49.3 (46.7-52) % | 673/1364 | 87.1 (83.1-90.6) % | 242/278 | 39.7 (36.7-42.6) % | 431/1086 |
| Mortality |  |  |  |  |  |  |  |
|  | 30-day | 3.5 (2.5-4.5) % | 47/1358 | 7.3 (4.4-10.5) % | 20/275 | 2.5 (1.6-3.5) % | 27/1083 |
|  | 120-day | 8.8 (7.4-10.4) % | 120/1358 | 18.9 (14.5-23.6) % | 52/275 | 6.3 (4.8-7.7) % | 68/1083 |
|  | 1-year | 16.3 (14.4-18.3) % | 222/1358 | 32 (26.5-37.5) % | 88/275 | 12.4 (10.3-14.4) % | 134/1083 |

#### Supplementary table 7D: Outcomes of participants not frail in the SNAP-3 cohort, with and without delirium

|  |  | **All** | | **Delirium** | | **No delirium** | |
| --- | --- | --- | --- | --- | --- | --- | --- |
| **Characteristic** |  | **Summary** | **Number** | **Summary** | **Number** | **Summary** | **Number** |
| Postoperative LOS (days) | Median [IQR] | 1 [0-3] | 5514 | 11 [6-16] | 192 | 1 [0-3] | 5322 |
|  | 80th centile | 5 |  | 18.8 |  | 4 |  |
| Postoperative morbidity* |  | 21.6 (20.5-22.6) % | 1215/5628 | 86.9 (82.3-91.9) % | 172/198 | 19.2 (18.2-20.3) % | 1043/5430 |
| Mortality |  |  |  |  |  |  |  |
|  | 30-day | 0.5 (0.3-0.7) % | 27/5605 | 3.5 (1-6.6) % | 7/198 | 0.4 (0.2-0.6) % | 20/5407 |
|  | 120-day | 1.6 (1.3-1.9) % | 90/5605 | 8.6 (5.1-12.6) % | 17/198 | 1.4 (1-1.7) % | 73/5407 |
|  | 1-year | 4.6 (4.1-5.2) % | 260/5605 | 19.7 (14.1-25.3) % | 39/198 | 4.1 (3.6-4.6) % | 221/5407 |

#### Supplementary table 7E: Outcomes of participants having elective surgery in the SNAP-3 cohort, with and without delirium

|  |  | **All** | | **Delirium** | | **No delirium** | |
| --- | --- | --- | --- | --- | --- | --- | --- |
| **Characteristic** |  | **Summary** | **Number** | **Summary** | **Number** | **Summary** | **Number** |
| Postoperative LOS (days) | Median [IQR] | 1 [0-3] | 4822 | 7 [5-12] | 130 | 1 [0-2] | 4692 |
|  | 80th centile | 3 |  | 13.2 |  | 3 |  |
| Postoperative morbidity* |  | 17.2 (16.2-18.3) % | 845/4921 | 79.1 (71.6-85.8) % | 106/134 | 15.4 (14.4-16.4) % | 739/4787 |
| Mortality |  |  |  |  |  |  |  |
|  | 30-day | 0.3 (0.1-0.4) % | 14/4905 | 1.5 (0-3.7) % | 2/134 | 0.3 (0.1-0.4) % | 12/4771 |
|  | 120-day | 1.1 (0.8-1.4) % | 54/4905 | 6.7 (3-11.2) % | 9/134 | 0.9 (0.7-1.2) % | 45/4771 |
|  | 1-year | 3.8 (3.2-4.4) % | 187/4905 | 14.2 (9-20.1) % | 19/134 | 3.5 (3-4.1) % | 168/4771 |

#### Supplementary table 7F: Outcomes of participants having non-elective surgery in the SNAP-3 cohort, with and without delirium

|  |  | **All** | | **Delirium** | | **No delirium** | |
| --- | --- | --- | --- | --- | --- | --- | --- |
| **Characteristic** |  | **Summary** | **Number** | **Summary** | **Number** | **Summary** | **Number** |
| Postoperative LOS (days) | Median [IQR] | 5 [1-13] | 2075 | 14 [9-27] | 324 | 3 [1-9] | 1751 |
|  | 80th centile | 15 |  | 30 |  | 12 |  |
| Postoperative morbidity* |  | 49.4 (47.3-51.5) % | 1055/2134 | 89.9 (86.7-93) % | 310/345 | 41.6 (39.4-43.9) % | 745/1789 |
| Mortality |  |  |  |  |  |  |  |
|  | 30-day | 2.9 (2.2-3.6) % | 61/2120 | 7.3 (4.7-10.2) % | 25/342 | 2 (1.3-2.7) % | 36/1778 |
|  | 120-day | 7.5 (6.4-8.6) % | 158/2120 | 17.5 (13.7-21.6) % | 60/342 | 5.5 (4.5-6.6) % | 98/1778 |
|  | 1-year | 14.1 (12.6-15.5) % | 298/2120 | 31.6 (26.6-36.3) % | 108/342 | 10.7 (9.3-12.1) % | 190/1778 |

#### Supplementary table 7G: Outcomes of participants having inpatient surgery in the SNAP-3 cohort, with and without delirium

|  |  | **All** | | **Delirium** | | **No delirium** | |
| --- | --- | --- | --- | --- | --- | --- | --- |
| **Characteristic** |  | **Summary** | **Number** | **Summary** | **Number** | **Summary** | **Number** |
| Postoperative LOS (days) | Median [IQR] | 3 [1-7] | 4535 | 12 [7-22] | 452 | 3 [1-6] | 4083 |
|  | 80th centile | 9 |  | 26 |  | 7 |  |
| Postoperative morbidity* |  | 40.1 (38.8-41.5) % | 1896/4722 | 86.8 (83.6-89.7) % | 414/477 | 34.9 (33.5-36.3) % | 1482/4245 |
| Mortality |  |  |  |  |  |  |  |
|  | 30-day | 1.5 (1.2-1.9) % | 70/4628 | 5.5 (3.6-7.8) % | 26/474 | 1.1 (0.8-1.4) % | 44/4154 |
|  | 120-day | 4.3 (3.7-4.9) % | 199/4628 | 14.3 (11.4-17.7) % | 68/474 | 3.2 (2.6-3.7) % | 131/4154 |
|  | 1-year | 9 (8.3-9.9) % | 418/4628 | 26.6 (22.4-30.6) % | 126/474 | 7 (6.3-7.8) % | 292/4154 |

### Supplementary results: main analysis

#### Supplementary table 8: The impact of delirium on predicted outcomes, using adjusted models and multiple imputation via chained equations

| **Outcomes** | **Predicted days for those without delirium** | **(95 % CI)** | | **Predicted days for those with delirium** | **(95 % CI)** | |
| --- | --- | --- | --- | --- | --- | --- |
| **Median postoperative length of stay** | -0.03 | (-0.10, | 0.04) | 5.14 | (3.82, | 6.46) |
| **80th percentile postoperative length of stay** | 0.40 | (0.18, | 0.62) | 9.95 | (7.02, | 12.89) |
|  | **Predicted probabilities for those without delirium (%)** | **(95 % CI)** | | **Predicted probabilities for those with delirium (%)** | **(95 % CI)** | |
| **Postoperative morbidity** | 2.45 | (1.55, | 3.84) | 20.32 | (12.79, | 30.72) |
| **30-day mortality** | 0.08 | (0.02, | 0.37) | 0.10 | (0.02, | 0.59) |
| **120-day mortality** | 0.30 | (0.11, | 0.79) | 0.53 | (0.19, | 1.53) |
| **1-year mortality** | 1.13 | (0.63, | 2.02) | 2.26 | (1.18, | 4.30) |

Notes: This table supports Figure 1.

Length of stay: Predicted days are calculated from quantile regression models (median, 80^th^ percentile), using multiple imputation using chained equations for missing data. Predictions are for the reference group: female, age 72 years, England, Index of Multiple Deprivation (IMD) quintile 5 (least deprived), Clinical Frailty Score (CFS) 1-3, no multimorbidity, no dementia, no malignancy, no postoperative perioperative medicine service, elective surgical urgency, minor operative severity, no preoperative assessment clinic, not morbid by day 7. Normal-based 95% confidence intervals were calculated using bootstrapped standard errors (1000 replications). Full results from the regression analyses are given in supplementary tables 9 and 10.

Postoperative morbidity: Conditional predicted probabilities are calculated from a multilevel logistic regression model using multiple imputation using chained equations for missing data. Predictions are for the reference group: female, age 72 years, England, IMD quintile 5 (least deprived), CFS 1-3, no multimorbidity, no dementia, no malignancy, no visual or hearing impairments, no polypharmacy, no postoperative perioperative medicine service, elective surgical urgency, minor operative severity, no preoperative assessment clinic, not morbid by day 7, at a ‘typical’ hospital. Full results from the regression analysis are given in supplementary table 11.

Postoperative mortality: Conditional predicted probabilities are calculated from a multilevel logistic regression model using multiple imputation using chained equations for missing data. Predictions are for the reference group: female, age 72 years, England, IMD quintile 5 (least deprived), CFS 1-3, no multimorbidity, no dementia, no malignancy, no visual or hearing impairments, no polypharmacy, no postoperative perioperative medicine service, elective surgical urgency, minor operative severity, no preoperative assessment clinic, at a ‘typical’ hospital. Full results from the regression analyses are given in supplementary tables 12, 13, and 14.

#### *Supplementary table 9: Adjusted effect estimates from quantile regression with delirium as main exposure and median length of stay as outcome*

| **Variable** | **Adjusted difference in days (95% CI)** |
| --- | --- |
| Intercept | -0.03 (-0.10,0.04) |
| Delirium | 5.17 (3.84,6.49) |
| Clinical Frailty Scale (CFS)  (ref: CFS 1-3, no multimorbidity) |  |
| CFS 4, no multimorbidity | 0.14 (-0.17,0.46) |
| CFS 5, no multimorbidity | 0.21 (-0.31,0.73) |
| CFS 6, no multimorbidity | 0.28 (-0.51,1.07) |
| CFS 7-8, no multimorbidity | 1.34 (-1.81,4.48) |
| CFS 4, with multimorbidity | -0.04 (-0.32,0.23) |
| CFS 5, with multimorbidity | 0.16 (-0.52,0.84) |
| CFS 6, with multimorbidity | 0.33 (-0.64,1.30) |
| CFS 7-8, with multimorbidity | 0.67 (-2.89,4.22) |
| Multimorbidity (with CFS1-3) | 0.01 (-0.03,0.05) |
| Age (years) | 0.01 (-0.00,1.00) |
| Male | 0.00 (-0.04,0.03) |
| IMD/nation; ref: England, IMD quintile 5 (least deprived) |  |
| England, IMD1 (most deprived) | 0.01 (-0.06,0.08) |
| England, IMD2 | 0.04 (-0.04,0.12) |
| England, IMD3 | 0.02 (-0.05,0.08) |
| England, IMD4 | 0.03 (-0.03,0.09) |
| Northern Ireland | -0.20 (-0.70,0.30) |
| Scotland | -0.08 (-0.24,0.08) |
| Wales | -0.02 (-0.12,0.09) |
| Dementia | 0.39 (-0.68,1.46) |
| Malignancy | 0.02 (-0.03,0.06) |
| Surgical urgency; ref: Elective |  |
| Emergency | 2.10 (0.29,3.90) |
| Urgent | 1.36 (0.81,1.91) |
| Expedited | 0.32 (-0.10,0.75) |
| Surgical severity; ref: minor |  |
| Intermediate | 0.02 (-0.02,0.07) |
| Major | 0.73 (0.34,1.11) |
| X Major | 1.54 (0.89,2.18) |
| Complex | 1.65 (1.08,2.22) |
| Pre-op clinic; ref: none | 0.09 (-0.05,0.23) |
| Postop care of the elderly; ref: none | 0.02 (-0.03,0.06) |
| POMS (excl. delirium) | 5.26 (4.74,5.77) |

Notes: CI: confidence interval. IMD: Index of multiple deprivation (for England), divided into quintiles (IMD1 = most deprived quintile, IMD5 = least deprived quintile). Estimates are given as adjusted quantile regression coefficients for median length of stay after multiple imputation using chained equations. All estimates are adjusted for age, sex, area social deprivation/nation, frailty, multimorbidity, the interaction between frailty and multimorbidity, dementia, malignancy, operative severity, surgical urgency, preoperative assessment clinic, the existence of a postoperative perioperative medicine service, and postoperative morbidity. Normal-based 95% confidence intervals were calculated using bootstrapped standard errors (1000 replications).

#### *Supplementary table 10: Adjusted effect estimates from quantile regression with delirium as main exposure and 80^th^ percentile length of stay as outcome*

| **Variable** | **Adjusted difference in days (95% CI)** |
| --- | --- |
| Intercept | 0.40 (0.18,0.62) |
| Delirium | 9.56 (6.64,12.47) |
| Clinical Frailty Scale (CFS); ref: CFS 1-3, no multimorbidity |  |
| CFS 4, no multimorbidity | 0.23 (-0.20,0.65) |
| CFS 5, no multimorbidity | 0.15 (-1.21,1.51) |
| CFS 6, no multimorbidity | 1.08 (-2.20,4.35) |
| CFS 7-8, no multimorbidity | 11.27 (0.30,22.23) |
| CFS 4, with multimorbidity | 0.11 (-0.38,0.60) |
| CFS 5, with multimorbidity | 0.67 (-0.80,2.14) |
| CFS 6, with multimorbidity | 0.14 (-3.61,3.89) |
| CFS 7-8, with multimorbidity | -5.36 (-17.22,6.49) |
| Multimorbidity (with CFS1-3) | 0.07 (-0.16,0.29) |
| Age (years) | 0.04 (0.03,2.27) |
| Male | -0.05 (-0.16,0.06) |
| IMD/nation; ref: England, IMD quintile 5 (least deprived) |  |
| England, IMD1 (most deprived) | 0.09 (-0.14,0.31) |
| England, IMD2 | 0.13 (-0.08,0.34) |
| England, IMD3 | 0.08 (-0.09,0.25) |
| England, IMD4 | 0.08 (-0.07,0.24) |
| Northern Ireland | -0.25 (-1.64,1.13) |
| Scotland | -0.08 (-0.25,0.09) |
| Wales | 0.22 (-0.18,0.62) |
| Dementia | 0.24 (-4.02,4.50) |
| Malignancy | 0.08 (-0.12,0.27) |
| Surgical urgency; ref: Elective |  |
| Emergency | 7.19 (1.53,12.84) |
| Urgent | 4.65 (3.39,5.91) |
| Expedited | 1.45 (0.94,1.95) |
| Surgical severity; ref: minor |  |
| Intermediate | 0.03 (-0.09,0.15) |
| Major | 0.93 (0.77,1.09) |
| X Major | 2.15 (1.90,2.40) |
| Complex | 2.72 (2.09,3.35) |
| Pre-op clinic; ref: none | 0.10 (-0.09,0.29) |
| Postop care of the elderly; ref: none | 0.02 (-0.13,0.16) |
| POMS (excl. delirium) | 9.85 (8.86,10.83) |

Notes: CI: confidence interval. IMD: Index of multiple deprivation (for England), divided into quintiles (IMD1 = most deprived quintile, IMD5 = least deprived quintile). Estimates are given as adjusted quantile regression coefficients for 80^th^ percentile length of stay after multiple imputation using chained equations. All estimates are adjusted for age, sex, area social deprivation/nation, frailty, multimorbidity, the interaction between frailty and multimorbidity, dementia, malignancy, operative severity, surgical urgency, preoperative assessment clinic, the existence of a postoperative perioperative medicine service, and postoperative morbidity. Normal-based 95% confidence intervals were calculated using bootstrapped standard errors (1000 replications).

#### Supplementary table 11: Adjusted odds ratios from mixed effects logistic regression *with delirium as main exposure and postoperative morbidity as outcome*

| **Variable** | **Adjusted Odds Ratio (95% CI)** |
| --- | --- |
| Intercept | 0.03 (0.02,0.04) |
| Delirium | 10.16 (7.44,13.88) |
| Clinical Frailty Scale (CFS)  (ref: CFS 1-3, no multimorbidity) |  |
| CFS 4, no multimorbidity | 1.64 (1.21,2.22) |
| CFS 5, no multimorbidity | 2.35 (1.51,3.65) |
| CFS 6, no multimorbidity | 2.72 (1.55,4.78) |
| CFS 7-8, no multimorbidity | 3.88 (1.93,7.78) |
| CFS 4, with multimorbidity | 1.00 (0.69,1.45) |
| CFS 5, with multimorbidity | 0.95 (0.58,1.57) |
| CFS 6, with multimorbidity | 0.91 (0.48,1.72) |
| CFS 7-8, with multimorbidity | 1.07 (0.48,2.38) |
| Multimorbidity (with CFS1-3) | 1.36 (1.13,1.64) |
| Age (years) | 1.00 (1.00,1.00) |
| Male | 0.88 (0.76,1.00) |
| IMD/nation; ref: England, IMD quintile 5 (least deprived) |  |
| England, IMD1 (most deprived) | 0.88 (0.69,1.14) |
| England, IMD2 | 0.85 (0.67,1.08) |
| England, IMD3 | 1.08 (0.88,1.34) |
| England, IMD4 | 0.92 (0.75,1.14) |
| Northern Ireland | 1.81 (0.73,4.50) |
| Scotland | 0.99 (0.66,1.48) |
| Wales | 1.25 (0.84,1.86) |
| Dementia | 0.48 (0.30,0.78) |
| Malignancy | 1.10 (0.94,1.30) |
| Surgical urgency; ref: Elective |  |
| Emergency | 7.93 (5.31,11.85) |
| Urgent | 6.15 (5.11,7.40) |
| Expedited | 3.03 (2.50,3.66) |
| Surgical severity; ref: minor |  |
| Intermediate | 1.19 (0.80,1.78) |
| Major | 2.70 (1.84,3.95) |
| X Major | 7.22 (4.91,10.60) |
| Complex | 15.65 (10.50,23.34) |
| Pre-op clinic; ref: none | 1.37 (1.15,1.62) |
| Postop care of the elderly; ref: none | 1.07 (0.82,1.40) |

Notes: CI: confidence interval, IMD: Index of multiple deprivation (for England), divided into quintiles (IMD1 = most deprived quintile, IMD5 = least deprived quintile). Estimates are given as adjusted odds ratios from mixed effects logistic regression for postoperative morbidity (excluding delirium) within seven days of surgery, including a random intercept for hospital and after multiple imputation using chained equations. All estimates are adjusted for age, sex, area social deprivation/nation, frailty, multimorbidity, the interaction between frailty and multimorbidity, dementia, malignancy, operative severity, surgical urgency, preoperative assessment clinic, and the existence of a postoperative perioperative medicine service.

#### Supplementary table 12: Adjusted odds ratios from mixed effects logistic regression with delirium as main exposure and 30-day mortality as outcome

| **Variable** | **Adjusted Odds Ratio (95% CI)** |
| --- | --- |
| Intercept | 0.00 (0.00,0.00) |
| Delirium | 1.39 (0.78,2.48) |
| Clinical Frailty Scale (CFS); ref: CFS 1-3, no multi morbidity) |  |
| CFS 4-6, no multimorbidity | 2.32 (0.62,8.71) |
| CFS 7-8, no multimorbidity | 11.12 (2.43,50.84) |
| CFS 4-6, with multimorbidity | 0.92 (0.20,4.32) |
| CFS 7-8, with multimorbidity | 1.07 (0.20,5.77) |
| Multimorbidity (with CFS1-3) | 1.22 (0.34,4.34) |
| Age (years) | 1.00 (1.00,1.00) |
| Male | 1.24 (0.75,2.07) |
| IMD/nation; ref: England, IMD quintile 5 (least deprived) |  |
| England, IMD1 (most deprived) | 1.62 (0.67,3.91) |
| England, IMD2 | 1.20 (0.47,3.08) |
| England, IMD3 | 1.22 (0.53,2.81) |
| England, IMD4 | 1.25 (0.53,2.93) |
| Northern Ireland | 0.51 (0.06,4.79) |
| Scotland | 1.52 (0.52,4.46) |
| Wales | 1.34 (0.43,4.18) |
| Dementia | 0.71 (0.32,1.59) |
| Malignancy | 2.15 (1.23,3.76) |
| Visual impairment | 0.72 (0.23,2.23) |
| Hearing impairment | 1.00 (0.48,2.07) |
| Polypharmacy | 1.47 (0.80,2.70) |
| Surgical urgency; ref: Elective |  |
| Emergency | 2.02 (0.52,7.88) |
| Urgent | 3.51 (1.65,7.44) |
| Expedited | 3.63 (1.71,7.69) |
| Surgical severity; ref: minor |  |
| Intermediate | 0.39 (0.13,1.18) |
| Major | 0.34 (0.12,0.94) |
| X Major | 0.45 (0.16,1.27) |
| Complex | 0.41 (0.13,1.31) |
| Pre-op clinic; ref: none | 1.58 (0.79,3.16) |
| Postop care of the elderly; ref: none | 0.95 (0.50,1.83) |
| POMS (excl. delirium) | 4.58 (2.25,9.34) |

Notes: CI: confidence interval, IMD: Index of multiple deprivation (for England), divided into quintiles (IMD1 = most deprived quintile, IMD5 = least deprived quintile). Estimates are given as adjusted odds ratios from mixed effects logistic regression for mortality within 30 days of surgery, including a random intercept for hospital and after multiple imputation using chained equations. All estimates are adjusted for age, sex, area social deprivation/nation, frailty, multimorbidity, the interaction between frailty and multimorbidity, dementia, malignancy, visual impairments, hearing impairments, polypharmacy, operative severity, surgical urgency, preoperative assessment clinic, the existence of a postoperative perioperative medicine service, and postoperative morbidity. Note that frailty was regrouped into three categories due to small numbers.

#### Supplementary table 13: Adjusted odds ratios from mixed effects logistic regression with delirium as main exposure and 120-day mortality as outcome

| **Variable** | **Adjusted Odds Ratio (95% CI)** |
| --- | --- |
| Intercept | 0.00 (0.00,0.01) |
| Delirium | 1.78 (1.21,2.61) |
| Clinical Frailty Scale (CFS); ref: CFS 1-3, no multimorbidity |  |
| CFS 4, no multimorbidity | 3.37 (1.62,7.00) |
| CFS 5, no multimorbidity | 2.82 (1.09,7.32) |
| CFS 6, no multimorbidity | 3.20 (1.14,8.96) |
| CFS 7-8, no multimorbidity | 5.30 (1.86,15.13) |
| CFS 4, with multimorbidity | 0.05 (0.20,1.26) |
| CFS 5, with multimorbidity | 0.72 (0.24,2.12) |
| CFS 6, with multimorbidity | 0.90 (0.28,2.86) |
| CFS 7-8, with multimorbidity | 1.20 (0.38,3.79) |
| Multimorbidity (with CFS1-3) | 1.39 (0.74,2.63) |
| Age (years) | 1.00 (1.00,1.00) |
| Male | 1.47 (1.08,2.01) |
| IMD/Nation; ref: England, IMD quintile 5 (least deprived) |  |
| England, IMD1 (most deprived) | 0.93 (0.54,1.59) |
| England, IMD2 | 0.77 (0.44,1.33) |
| England, IMD3 | 0.91 (0.56,1.48) |
| England, IMD4 | 0.85 (0.52,1.40) |
| Northern Ireland | 0.47 (0.11,2.06) |
| Scotland | 1.22 (0.62,2.40) |
| Wales | 1.00 (0.49,2.06) |
| Dementia | 0.89 (0.51,1.56) |
| Malignancy | 2.46 (1.76,3.44) |
| Visual impairment | 0.64 (0.29,1.41) |
| Hearing impairment | 1.00 (0.63,1.59) |
| Polypharmacy | 1.20 (0.83,1.72) |
| Surgical urgency; ref: Elective |  |
| Emergency | 2.63 (1.18,5.86) |
| Urgent | 3.85 (2.53,5.86) |
| Expedited | 2.99 (1.94,4.62) |
| Surgical severity; ref: minor |  |
| Intermediate | 0.94 (0.45,1.95) |
| Major | 0.86 (0.42,1.74) |
| X Major | 0.77 (0.37,1.59) |
| Complex | 0.65 (0.29,1.46) |
| Pre-op clinic; ref: none | 1.52 (1.01,2.28) |
| Postop care of the elderly; ref: none | 0.80 (0.49,1.31) |
| POMS (excl. delirium) | 2.54 (1.74,3.69) |

Notes: CI: confidence interval, IMD: Index of multiple deprivation (for England), divided into quintiles (IMD1 = most deprived quintile, IMD5 = least deprived quintile). Estimates are given as adjusted odds ratios from mixed effects logistic regression for mortality within 120 days of surgery, including a random intercept for hospital and after multiple imputation using chained equations. All estimates are adjusted for age, sex, area social deprivation/nation, frailty, multimorbidity, the interaction between frailty and multimorbidity, dementia, malignancy, visual impairments, hearing impairments, polypharmacy, operative severity, surgical urgency, preoperative assessment clinic, the existence of a postoperative perioperative medicine service, and postoperative morbidity.

#### Supplementary table 14: Adjusted odds ratios from mixed effects logistic regression with delirium as main exposure and one-year mortality as outcome

| **Variable** | **Adjusted Odds Ratio (95% CI)** |
| --- | --- |
| Intercept | 0.01 (0.01,0.02) |
| Delirium | 2.02 (1.51,2.70) |
| Clinical Frailty Scale (CFS); ref: CFS 1-3, no multimorbidity |  |
| CFS 4, no multimorbidity | 2.68 (1.70,4.24) |
| CFS 5, no multimorbidity | 1.58 (0.78,3.22) |
| CFS 6, no multimorbidity | 1.48 (0.65,3.34) |
| CFS 7-8, no multimorbidity | 5.06 (2.47,10.39) |
| CFS 4, with multimorbidity | 0.62 (0.35,1.11) |
| CFS 5, with multimorbidity | 1.02 (0.46,2.26) |
| CFS 6, with multimorbidity | 1.80 (0.74,4.40) |
| CFS 7-8, with multimorbidity | 1.04 (0.47,2.33) |
| Multimorbidity (with CFS1-3) | 1.22 (0.84,1.76) |
| Age (years) | 1.00 (1.00,1.00) |
| Male | 1.47 (1.19,1.81) |
| IMD/Nation; ref: England, IMD quintile 5 (least deprived) |  |
| England, IMD1 | 1.02 (0.70,1.47) |
| England, IMD2 | 0.87 (0.60,1.25) |
| England, IMD3 | 1.11 (0.80,1.53) |
| England, IMD4 | 0.88 (0.63,1.23) |
| Northern Ireland | 0.23 (0.06,0.82) |
| Scotland | 1.24 (0.80,1.93) |
| Wales | 1.06 (0.67,1.67) |
| Dementia | 1.00 (0.64,1.58) |
| Malignancy | 2.97 (2.38,3.71) |
| Visual impairment | 1.24 (0.76,2.03) |
| Hearing impairment | 0.80 (0.57,1.12) |
| Polypharmacy | 1.34 (1.05,1.72) |
| Surgical urgency; ref: Elective |  |
| Emergency | 2.09 (1.21,3.62) |
| Urgent | 2.55 (1.94,3.36) |
| Expedited | 2.07 (1.56,2.74) |
| Surgical severity; ref: minor |  |
| Intermediate | 1.00 (0.63,1.61) |
| Major | 0.88 (0.56,1.39) |
| X Major | 0.68 (0.42,1.10) |
| Complex | 0.72 (0.43,1.22) |
| Pre-op clinic; ref: none | 1.25 (0.96,1.63) |
| Postop care of the elderly; ref: none | 0.96 (0.73,1.26) |
| POMS (excl. delirium) | 2.03 (1.58,2.60) |

Notes: CI: confidence interval, IMD: Index of multiple deprivation (for England), divided into quintiles (IMD1 = most deprived quintile, IMD5 = least deprived quintile). Estimates are given as adjusted odds ratios from mixed effects logistic regression for mortality within one year of surgery, including a random intercept for hospital and after multiple imputation using chained equations. All estimates are adjusted for age, sex, area social deprivation/nation, frailty, multimorbidity, the interaction between frailty and multimorbidity, dementia, malignancy, visual impairments, hearing impairments, polypharmacy, operative severity, surgical urgency, preoperative assessment clinic, the existence of a postoperative perioperative medicine service, and postoperative morbidity.

### Supplementary results: sensitivity analyses

#### Supplementary table 15: Effect estimates comparing the effect of postoperative delirium within 7 days versus no delirium, complete cases only.

| **Outcome** | **Sample size** | **Estimate (95 % CI)** |
| --- | --- | --- |
| Median length of stay (difference in days) | 4578 | 4.7 (3.3, 6.1) |
| 80th percentile length of stay (difference in days) | 4578 | 9.4 (6.1, 12.7) |
| Postoperative morbidity (OR) | 4630 | 8.5 (6.0, 12.1) |
| 30-day mortality (OR) | 4581 | 1.3 (0.6, 2.6) |
| 120-day mortality (OR) | 4581 | 1.6 (1.0, 2.5) |
| 1-year mortality (OR) | 4581 | 1.9 (1.4, 2.7) |

*Note: OR: odds ratio. CI: confidence interval. Sample sizes differ due to missing data. All models adjusted for age, sex, area social deprivation/nation, frailty, multimorbidity, the interaction between frailty and multimorbidity, dementia, malignancy, operative severity, surgical urgency, preoperative assessment clinic, and the existence of a postoperative perioperative medicine service. Length of stay models also adjusted for postoperative morbidity. Mortality models also adjusted for visual impairments, hearing impairments, polypharmacy, and postoperative morbidity. For length of stay models, bootstrapping with 1000 samples was used to estimate standard errors in the presence of clustering by hospitals. The morbidity and mortality models included a random intercept for hospitals.*

#### Supplementary table 16: Effect estimates of comparing the effect of postoperative delirium within 7 days versus no delirium, inpatient cases only.

| **Outcome** | **Sample size** | **Estimate (95 % CI)** |
| --- | --- | --- |
| Median length of stay (difference in days) | 4535 | 4.4 (3.1, 5.6) |
| 80th percentile length of stay (difference in days) | 4535 | 8.5 (5.6, 11.4) |
| Postoperative morbidity (OR) | 4722 | 6.3 (4.5, 8.9) |
| 30-day mortality (OR) | 4628 | 1.3 (0.6, 2.5) |
| 120-day mortality (OR) | 4628 | 1.5 (1.0, 2.4) |
| 1-year mortality (OR) | 4628 | 1.9 (1.4, 2.7) |

*Note: OR: odds ratio. CI: confidence interval. Sample sizes differ due to missing outcome data, which were not imputed. All models adjusted for age, sex, area social deprivation/nation, frailty, multimorbidity, the interaction between frailty and multimorbidity, dementia, malignancy, operative severity, surgical urgency, preoperative assessment clinic, and the existence of a postoperative perioperative medicine service. Length of stay models also adjusted for postoperative morbidity. Mortality models also adjusted for visual impairments, hearing impairments, polypharmacy, and postoperative morbidity. For length of stay models, bootstrapping with 1000 samples was used to estimate standard errors in the presence of clustering by hospitals. The morbidity and mortality models included a random intercept for hospitals.*

### SNAP-3 Collaborators

#### Supplementary table 17: SNAP-3 collaborators (in separate file)

### Supplementary figures

#### Supplementary figure 1: participant flow diagram

Initially recruited

7821

Did not meet inclusion criteria

71

Surgery postponed

425

No reason given for withdrawal

4

Eligible participants

7321

CFS 9

5

Withdrawal on patient wishes

187

No covariate data

1

Analysed participants

7128

#### Supplementary figure 2: directed acyclic graph for the relationship between delirium with postoperative length of stay


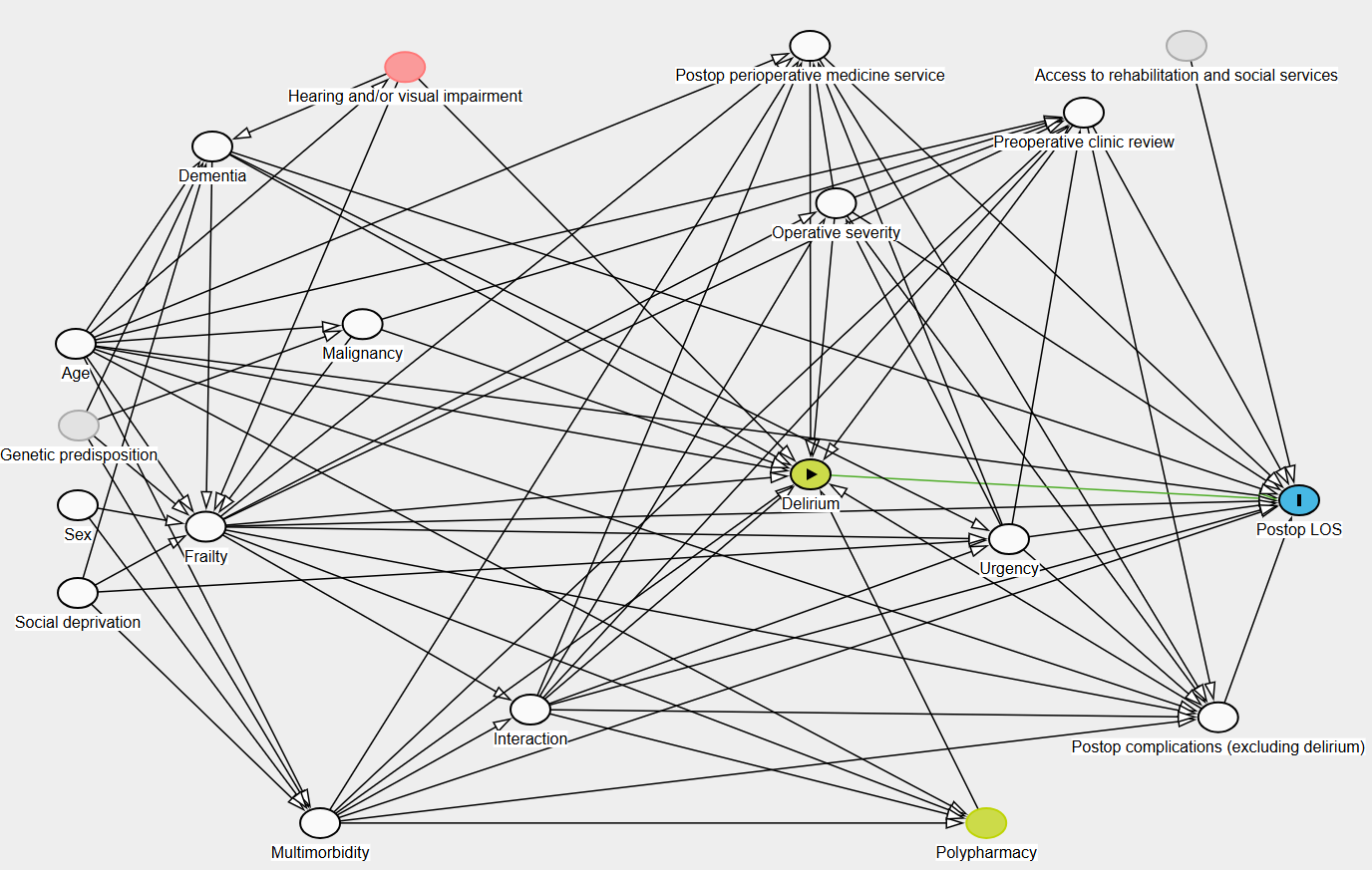


This Directed Acyclic Graph (DAG) illustrates the assumptions made about the exposure-outcome relationship between delirium (exposure) and postoperative length of stay (LOS, outcome) as shown in DAGitty. The exposure, delirium, is represented by a green node with a filled black triangle symbol, whilst the outcome, postoperative length of stay (LOS) is shown as a blue node with a thick black vertical line. Other variables are represented as follows: blue nodes depict ancestors of the outcome; red nodes represent ancestors of both the exposure and outcome (turning white if adjusted for); green nodes indicate ancestors of the exposure; pale grey nodes signify unobserved or latent variables; and darker grey nodes represent other variables. In this example, all ancestors of the outcome have been adjusted for, turning them white. Frailty is defined by a Clinical Frailty Scale score ≥5. Multimorbidity is defined as ≥2 comorbidities excluding dementia, malignancy, hearing and visual impairment. Postoperative perioperative medicine service is defined at hospital level where there is a proactive or reactive perioperative medicine service outside of orthogeriatrics.

#### Supplementary figure 3: directed acyclic graph for the relationship between delirium with postoperative morbidity


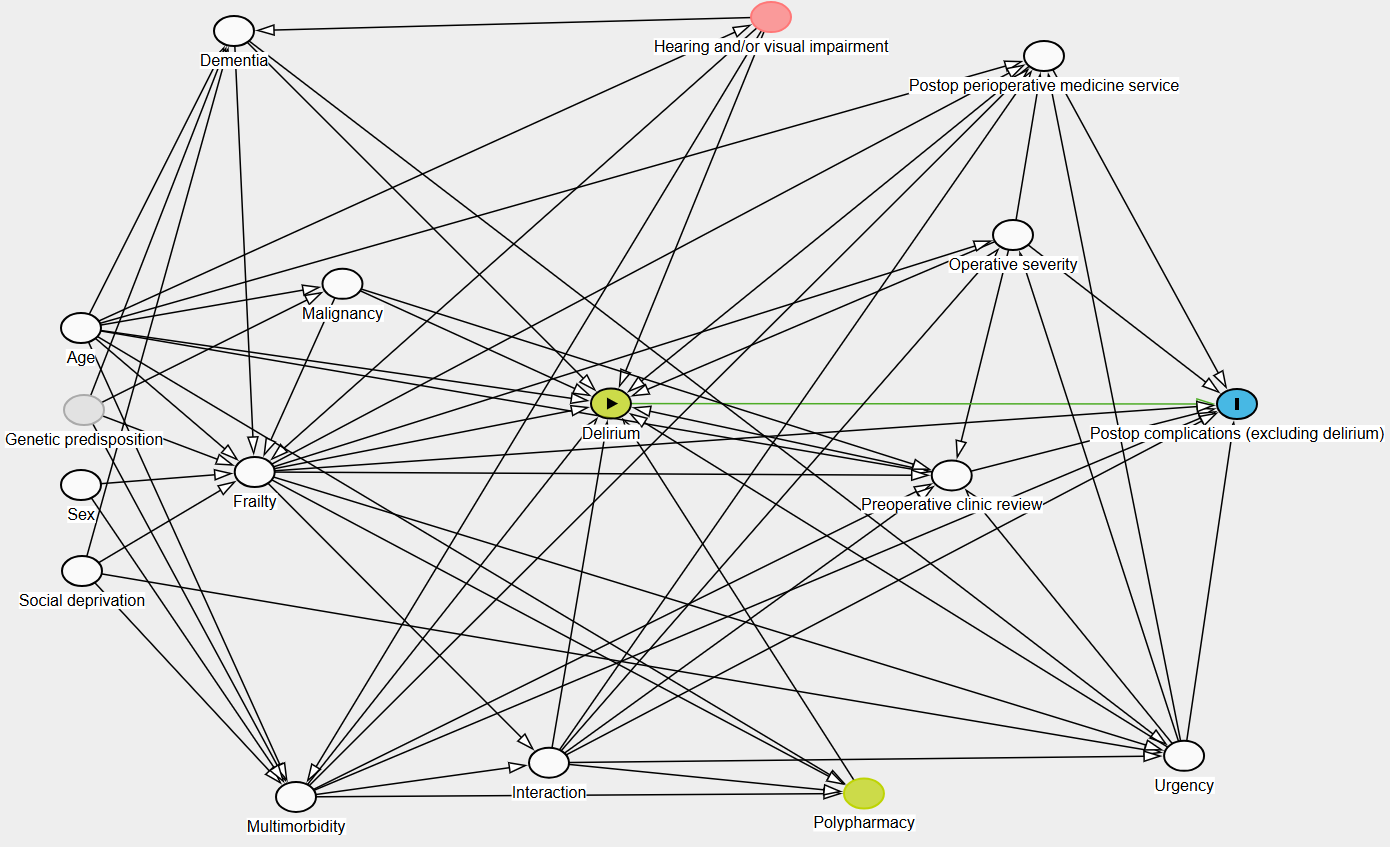


This Directed Acyclic Graph (DAG) illustrates the assumptions made about the exposure-outcome relationship between delirium (exposure) and postoperative morbidity (outcome) as shown in DAGitty. The exposure, delirium, is represented by a green node with a filled black triangle symbol. The outcome, postoperative morbidity is shown as a blue node with a thick black vertical line. Other variables are represented as follows: blue nodes depict ancestors of the outcome; red nodes represent ancestors of both the exposure and outcome (turning white if adjusted for); green nodes indicate ancestors of the exposure; pale grey nodes signify unobserved or latent variables; and darker grey nodes represent other variables. Frailty is defined by a Clinical Frailty Scale score ≥5. Multimorbidity is defined as ≥2 comorbidities excluding dementia, malignancy, hearing and visual impairment. Postoperative perioperative medicine service is defined at hospital level where there is a proactive or reactive perioperative medicine service outside of orthogeriatrics.

#### Supplementary figure 4: directed acyclic graph for the relationship between delirium with postoperative mortality


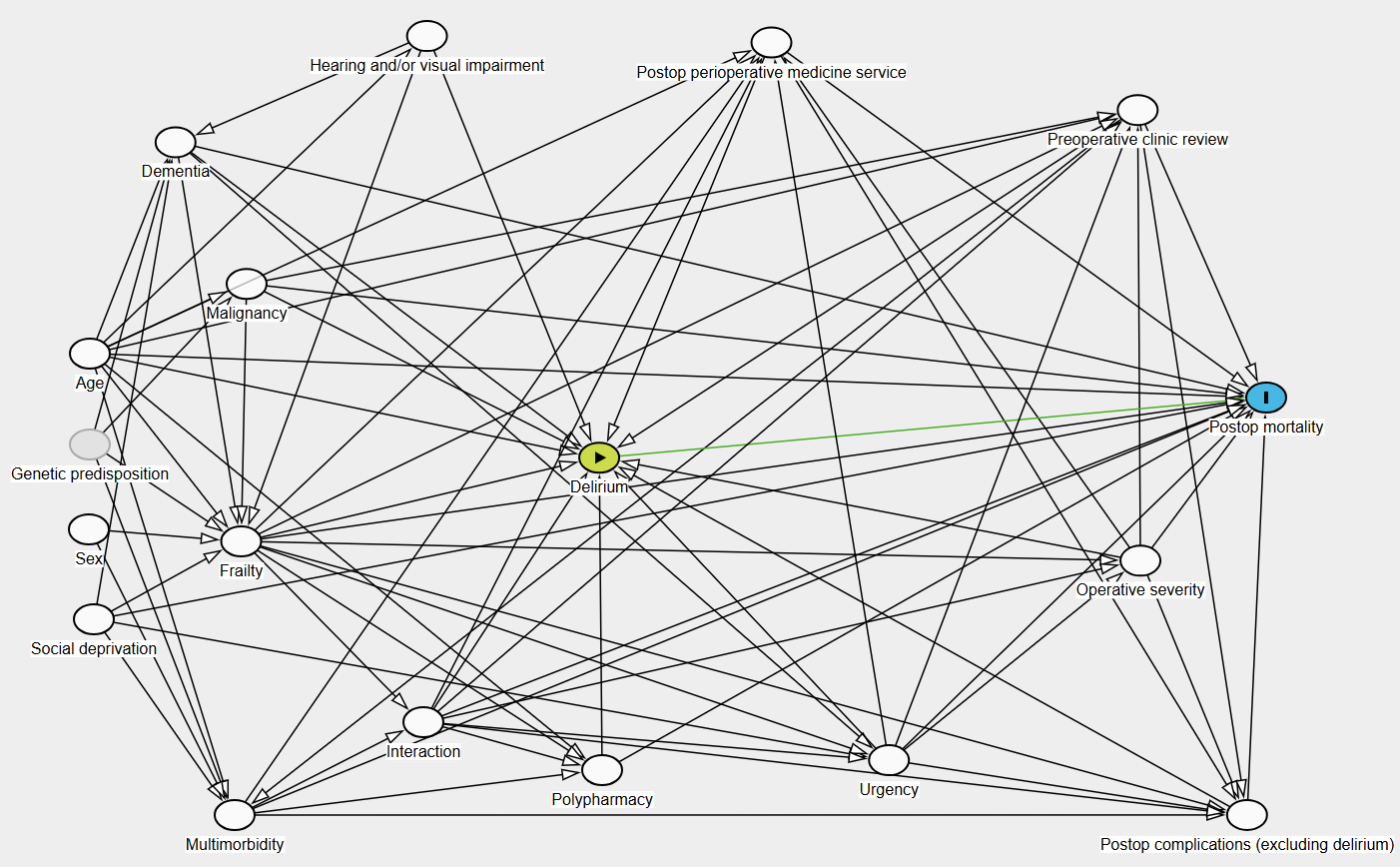


This Directed Acyclic Graph (DAG) illustrates the assumptions made about the exposure-outcome relationship between delirium (exposure) and postoperative mortality (outcome) as shown in DAGitty. The exposure, delirium, is represented by a green node with a filled black triangle symbol. The outcome, postoperative mortality is shown as a blue node with a thick black vertical line. Other variables are represented as follows: blue nodes depict ancestors of the outcome; red nodes represent ancestors of both the exposure and outcome (turning white if adjusted for); green nodes indicate ancestors of the exposure; pale grey nodes signify unobserved or latent variables; and darker grey nodes represent other variables. Frailty is defined by a Clinical Frailty Scale score ≥5. Multimorbidity is defined as ≥2 comorbidities excluding dementia, malignancy, hearing and visual impairment. Postoperative perioperative medicine service is defined at hospital level where there is a proactive or reactive perioperative medicine service outside of orthogeriatrics.

1 National Confidential Enquiry into Patient Outcome and Death. *The NCEPOD Classification of Intervention.* Abbey House, 74-76 John Street, London, EC1M 4DZ, 2004
